# Cooperative chalcogen bonding interactions in confined sites activate aziridines

**DOI:** 10.1038/s41467-022-31293-5

**Published:** 2022-06-22

**Authors:** Haofu Zhu, Pan-Pan Zhou, Yao Wang

**Affiliations:** 1 School of Chemistry and Chemical Engineering, Key Laboratory of the Colloid and Interface Chemistry, Ministry of Education, Shandong University, Jinan, 250100 China; 2 College of Chemistry and Chemical Engineering, Key Laboratory of Special Function Materials and Structure Design of Ministry of Education, Lanzhou University, Lanzhou, 730000 China

**Keywords:** Organic chemistry, Supramolecular chemistry

## Abstract

The activation of aziridines typically involves the use of strong Lewis acids or transition metals, and methods relying on weak interactions are rare. Herein, we report that cooperative chalcogen bonding interactions in confined sites can activate sulfonyl-protected aziridines. Among the several possible distinct bonding modes, our experiments and computational studies suggest that an activation mode involving the cooperative Se···O and Se···N interactions is in operation. The catalytic reactions between weakly bonded supramolecular species and nonactivated alkenes are considered as unfavorable approaches. However, here we show that the activation of aziridines by cooperative Se···O and Se···N interactions enables the cycloaddition of weakly bonded aziridine-selenide complex with nonactivated alkenes in a catalytic manner. Thus, weak interactions can indeed enable these transformations and are an alternative to methods relying on strong Lewis acids.

## Abstract (web summary)

The activation of aziridines is typically achieved via reaction with strong Lewis acids or transition metals. Here, the authors report that cooperative Se ∙ ∙∙O and Se ∙ ∙∙N noncovalent interactions can activate sulfonyl-protected aziridines, which enables their use in cycloaddition reactions with nonactivated alkenes.

## Introduction

**Fig. 1:**
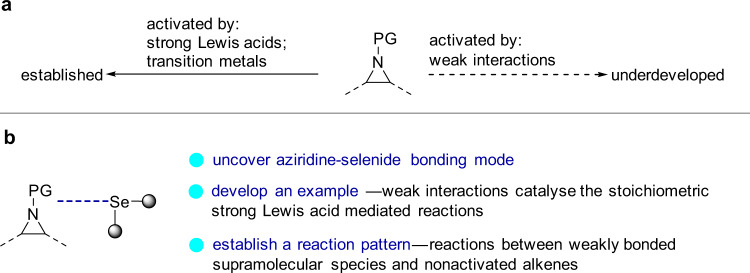
Distinct approaches to the activation of aziridines. **a** Activation of aziridines: the state of the art. **b** This work: chalcogen bonding activation and catalysis approach.

Weak interactions are among the significant evolutionary forces that are smartly exploited by nature to modulate the conformation of proteins and to drive cellular reactions. The simulation of this biomimetic strategy in supramolecular catalysis has gained fruitful achievements in promoting chemical reactions^1–10^. Amongst these interactions, catalysis with hydrogen bond plays a dominant role while halogen^2–4^, as well as chalcogen bonding catalysis^5–9^, has lately attracted ever-increasing research interest. Since the weak interactions restrict both the reactivity and the concentration of equilibrating supramolecular complex, this catalysis discipline has its constraint boundary considering the limitations of the activation targets and reaction patterns^10^. Even though weak interactions can activate a range of molecules such as carbonyl compounds, imines, nitro olefins, etc., it is a challenging task for these interactions to activate target molecules like aziridines which were conventionally handled by strong Lewis acids or transition metals. (Fig. 1a)^10^. Furthermore, the reactants suitable for trapping these weakly bonded supramolecular species have specific requirements, thus partly restricting the reaction patterns. For instance, the reactions between weakly bonded supramolecular species and nonactivated alkenes are considered unfavorable approaches in supramolecular catalysis^10^. To develop this field, research toward expanding the activation targets and establishing distinct reaction patterns is of vital importance.

Chalcogen bonding^11^, the noncovalent interaction between an electron donor and a chalcogen atom incorporated in a specific molecular entity, has recently found application in drug design^12^, material chemistry^13^, intramolecularly conformational control^14–18^, anion recognition and transport processes^19–26^. The theoretical investigation suggests that charge transfer, disperse force, and electrostatic potential are significant contributors to the formation of chalcogen bonding interactions^6^. As the chalcogen bond, in general, is weak^27^, catalysis with chalcogen bonding interactions based on divalent chalcoether is thus a rarely explored concept and a limited number of examples were introduced^28–37^. Herein, we report the chalcogen bonding mode of the aziridine-selenide complex and the catalytic reactions between these supramolecular complexes and nonactivated alkenes. We show that weak interactions enable catalytic transformations, in contrast with methods relying on stoichiometric strong Lewis acid-mediated organic transformations (Fig. 1b).

## Results and discussion

### Bonding property

**Fig. 2:**
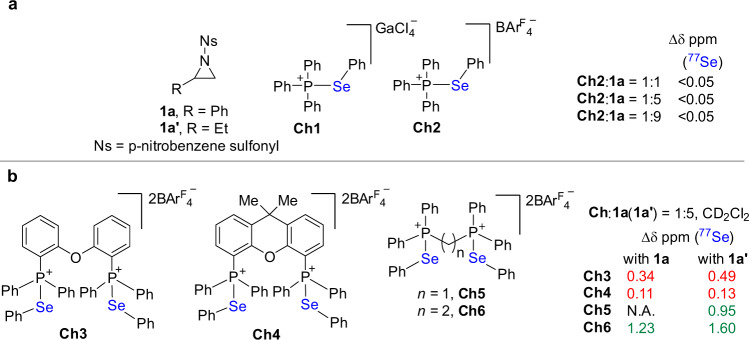
Chalcogen bonding interactions between Ch1-6 and aziridines (^77^Se NMR in CD_2_Cl_2_, 76 MHz, and 298 K). **a** Chalcogen bonding between monodentate catalysts **Ch1**-**2** and aziridine **1a**. **b** Chalcogen bonding between bidentate catalysts **Ch3**-**6** and aziridine **1a/1a**′.

We recently developed a class of phosphonium selenide-based chalcogen bonding catalysts, which showed catalytic activity in the activation of carbonyl groups and vinylindoles^28–31^. Since there are multiple Lewis basic sites in sulfonyl-protected aziridine like **1a**/**1a**′ (Fig. 2a), the binding modes for activating aziridines with such phosphonium selenides are much more complicated and the interactions with proper sites are crucial to the generation of catalytic capability. To understand the distinct bonding behavior of monodentate and bidentate chalcogen bonding donors **Ch1**-**6** with sulfonyl-protected aziridine, ^77^Se NMR analysis of the bonding complexes was carried out (Fig. 2). Upon using monodentate chalcogen bonding donors **Ch1-2** associated with ${{{{{\mathrm{GaCl}}}}}}_{4}^{-}$ or a less coordinating counteranion, i.e. ${{{{{\mathrm{BAr}}}}}}^{{{{{\mathrm{F}}}}}}_{{4}^{-}}$ (tetrakis(3,5-bis(trifluoromethyl)phenyl)borate), analysis of the mixture of **Ch1**/**2** and aziridine **1a** by ^77^Se NMR showed that the chemical shift of the ^77^Se signal almost remains unchanged even in the presence of 9.0 equivalent of aziridine **1a** (Fig. 2a). These observations suggest that a single chalcogen bonding interaction between **Ch1**/**2** and aziridine gives short-life supramolecular species with very low concentration.

Upon forming intermolecular interaction with **1a**/**1a**′, the distinct structure of **Ch3-6** leads to the variation of the ^77^Se signal in a different extent (Fig. 2b). Analysis of the mixture of **Ch3** and **1a** by ^77^Se NMR reveals that the chemical shift of the ^77^Se signal varies by 0.34 ppm while the ^31^P signal almost remains unchanged (<0.05 ppm), indicating that the interaction between selenium and aziridine induces the change of the ^77^Se signal. The interaction of **Ch4** with **1a** also resulted in an observable variation of the ^77^Se signal (0.11 ppm). In sharp contrast, an as much as 1.23 ppm down-filed shift of the ^77^Se signal was observed in the presence of **Ch6**. Since **1a** was not stable enough in the presence of **Ch5**, the variation of ^77^Se signal, in this case, is invalid. Further investigation using aziridine **1b** shows a similar trend of perturbation and the interaction of **Ch6** and **1b** gave the most dramatic change in the ^77^Se signal. These observations pose a confusing phenomenon that the variation of the ^77^Se signals of **Ch5** and **Ch6** is much more dramatic than chalcogen bonding donors **Ch3** and **Ch4**.

**Fig. 3:**
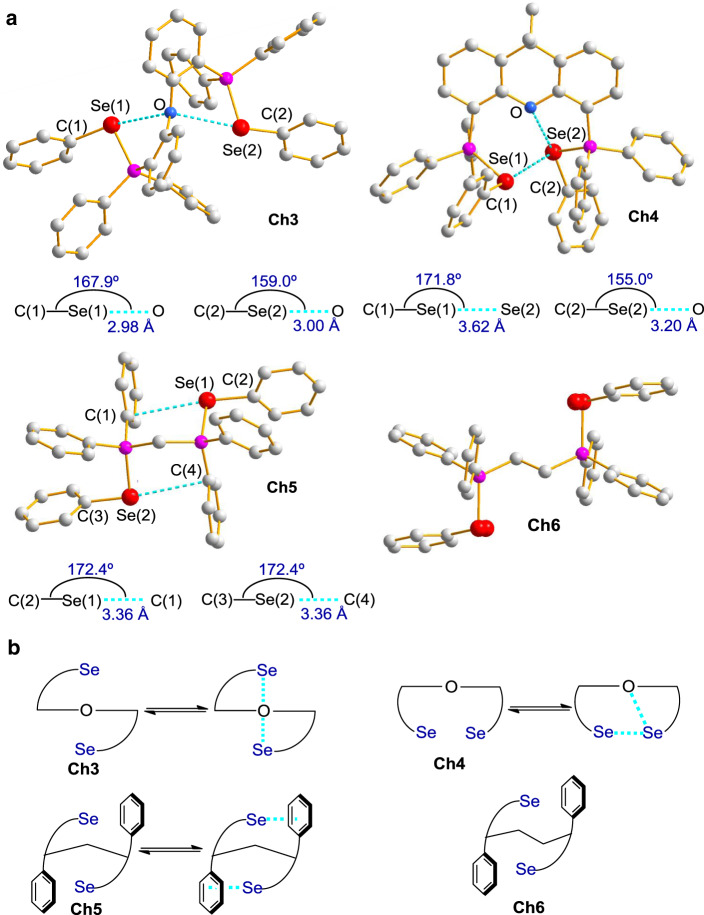
Relationship between bonding and structure. **a** X-ray crystal structures of **Ch3**-**6** (hydrogens and counteranions were omitted for clarity; the selenium atoms are disordered and no chalcogen bonding interaction in **Ch6**). **b** Equilibrium between intramolecular chalcogen bonding and non-bonding status of catalysts **Ch3**-**5** and catalyst **Ch6**.

To explain the aforementioned observations, the relationship between bonding and structure was investigated (Fig. 3a). To this end, the crystal structures of catalysts **Ch3**, **Ch5**, and **Ch6** associated with a ${{{{{\mathrm{BAr}}}}}}^{{{{{\mathrm{F}}}}}}_{{4}^{-}}$ counteranion were obtained. The crystal data for **Ch4** associated with ${{{{{\mathrm{BAr}}}}}}^{{{{{\mathrm{F}}}}}}_{{4}^{-}}$ was obtained previously^31^ and it would provide insightful information to elucidate its structure herein. The intramolecular chalcogen bonding interactions were observed in the crystal structures **Ch3**-**5** (Fig. 3a). These weak interactions are in agreement with the properties of chalcogen bonding since the separation between the two contact atoms is shorter than the sum of their van der Waals radii while the electron donor approaches the selenium atom approximately along the direction of the axis of C‒Se covalent bond^10^. The crystal structure of **Ch3** shows two Se···O bonding interactions while its rigid counterpart **Ch4** shows the presence of a ‘like-like’ Se···Se interaction and a Se···O interaction between the two bonding units. The bonding behavior of **Ch3** is similar to the previously observed structure of **Ch4** associated with a TfO^‒^ counteranion^29^, which also shows two intramolecular Se···O bonding interactions. These observations indicate the counteranion can pronouncedly affect the bonding interactions. The crystal structure of **Ch5** exhibits the Se···π bonding interactions between the selenium in one unit and the phenyl ring in the other unit. In contrast, no intramolecular chalcogen bonding interaction was observed in the crystal structure of **Ch6**. While X-ray crystallographic data of **Ch5** and **Ch6** indicate solvent in the crystals, powder X-ray diffraction (PXRD) data of **Ch3**-**6** are in agreement with the simulated results from corresponding single crystal diffraction data (see Supplementary Figs. 28–31).

Owing to the presence of the intramolecular Se···O interaction in **Ch3** as well as both Se···O and Se···Se interactions in **Ch4** (Fig. 3b), the addition of aziridine **1a**/**1a**′ competitively generates the intermolecular Se···O and Se···N interactions which are the similar type relative to the intramolecular interactions, thus resulting in less variation of the ^77^Se signals as shown in Fig. 2. In contrast, in the case of **Ch5** or **Ch6** which does not have an intramolecular Se···O or Se···Se interaction, the intermolecular Se···O and Se···N interactions with **1a**/**1a**′ would substantially change the bonding status of **Ch5** and **Ch6**, thus inducing more marked variation of the ^77^Se signals.

### Bonding mode

**Fig. 4:**
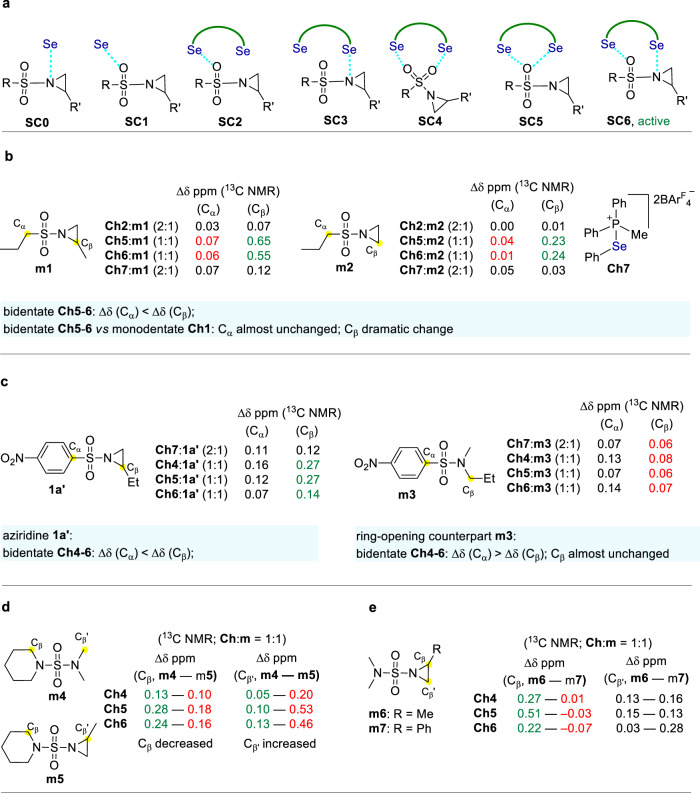
Probing binding modes (^13^C NMR in CD_2_Cl_2_, 100 MHz, 298 K). **a** Distinct binding modes. **b** Comparable chalcogen bonding experiments between monodentate and bidentate catalysts. **c** Comparable chalcogen bonding experiments between aziridine and its ring-opening counterpart. **d** Comparable chalcogen bonding experiments between catalysts **Ch4**-**6** and **m4**-**5**. **e** Substitution group effect on chalcogen bonding between aziridines **m6**-**7** and catalysts **Ch4**-**6**.

To investigate the active bonding modes (Fig. 4a), a range of molecular control experiments were carried out as depicted in Fig. 4b–e (see Supplementary Figs. 7–22). Using aziridine **m1** a model Lewis base, the control ^13^C NMR experiments reveal that the chemical shift of C_α_ (attached to S) almost remains unchanged while the chemical shift of the more remote C_β_ (two bonds from S) varies dramatically when comparing the bonding performance of monodentate catalyst **Ch2** (**Ch2**:**m1** 2:1) and their counterparts, bidentate catalysts **Ch5** and **Ch6** (**Ch**:**m1** 1:1) (Fig. 4b). For a bidentate catalyst, it is unlikely that the shifting from a single interaction mode (i.e., **SC0** or **SC1**) to a mode of double interaction with oxygen (i.e., **SC4** or **SC5)** would lead to almost no perturbation of the chemical shift of C_α_ while resulting in a marked variation of C_β_. Instead, the dramatic change of C_β_ attached to nitrogen indicates that a double interaction mode involving the direct interaction with nitrogen (i.e., **SC6**) is a reasonable binding mode to explain the experimental results. For a much sharper contrast, the formation of the complexes between **Ch5**/**6** and **m1** led to a negligible variation of C_α_ (0.07 and 0.06 ppm) while resulting in a much more pronounced change of C_β_ (0.65 and 0.55 ppm), which also suggests the direct interaction with nitrogen (i.e., **SC6**) to be the active complex. To give a more direct comparison, twofold **Ch7** (**Ch7**:**m1** 2:1) was used, i.e., monodentate version of **Ch5** and **Ch6**, and similar results were obtained. Further using **m2** as a model Lewis base gave similar observations.

The subsequent molecular control experiments further support **SC6** as the active binding species. As shown in Fig. 4c, in contrast to aziridine **1a**′, its ring-opening counterpart **m3** almost gave no change in the chemical shift of C_β_ regardless of in the presence of a monodentate or a bidentate catalyst. These observations indicate the structure of the nitrogen part is critically important to the chalcogen bonding interactions. The distinct perturbation of the chemical shift of C_β_ between aziridine **1a**′ and its ring-opening counterpart **m3** suggests a direct interaction with nitrogen (i.e., **SC6**) is in operation rather than interaction with the relatively more remote oxygen (**SC4** or **SC5**).

The results of the molecular balance experiments in Fig. 4d are further against a double interaction mode with oxygen (i.e., **SC4** and **SC5**). For C_β_ in the piperidine ring, the variation of the chemical shift decreased from **m4** to **m5**. However, a reverse variation of C_β′_ relative to C_β_ was observed, that is, a decreased variation of the chemical shift of C_β_ leads to an increased variation of the chemical shift of C_β′_. These molecular balance experiments indicate the equilibrating interaction with one of the two nitrogen parts is more favorable, thus suggesting binding modes **SC6** is operative rather than **SC4** or **SC5**. Further investigation reveals that the substitution group in the aziridine ring has a pronounced effect on the variation of the chemical shift of the ring carbons attached to nitrogen (Fig. 4e). It was found that the ring carbon C_β_ in **m6** with an aliphatic substitution (i.e., Me) resulted in a marked variation of the chemical shift while the ring carbon C_β_ in **m7** with an aromatic substitution (i.e., Ph) almost remains unchanged (**Ch4**) or even shifted to a reverse direction (**Ch5**, **Ch6**). These experiments indicate the substitution group in the aziridine ring can largely affect the interactions, thus pointing out a mode involving the direct interaction with the nitrogen atom (i.e., **SC6**). Therefore, the molecular control experiments depicted in Fig. 4b–e suggest **SC6** is the active binding mode of bidentate chalcogen bonding donors **Ch4**-**6**.

**Fig. 5:**
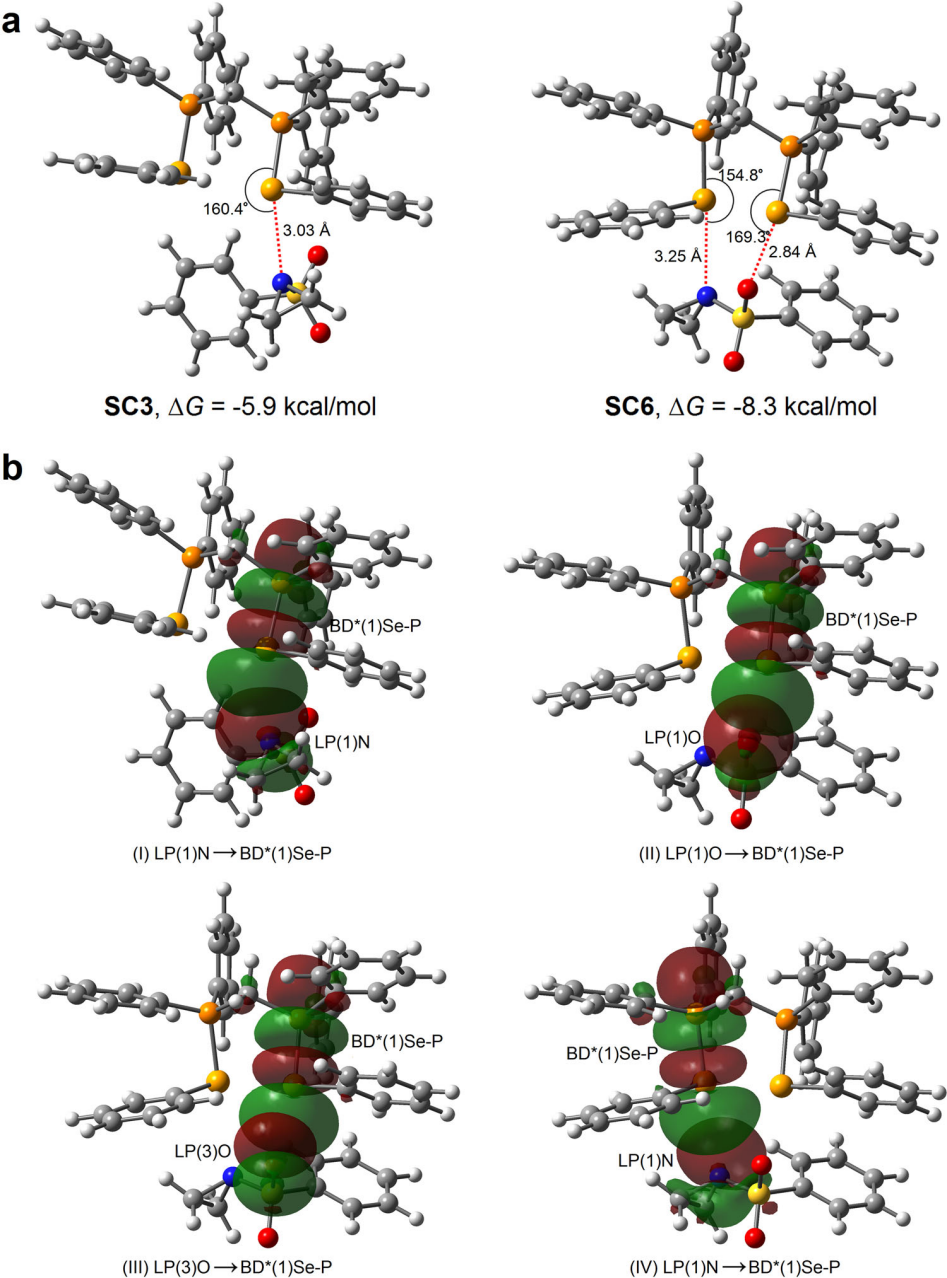
DFT calculations. **a** The optimized structures of the complexes **SC3** and **SC6** between **Ch5** and 1-(phenylsulfonyl)aziridine. **b** The key intermolecular donor-acceptor orbital interactions between **Ch5** and 1-(phenylsulfonyl)aziridine in **SC3** (I) and **SC6** (II-IV); BD*(1) denotes the σ* antibonding orbital; LP(1) and LP(3) denote the first and third lone-pair orbitals, respectively.

The binding mode of **SC6** between **Ch5** and 1-(phenylsulfonyl)aziridine were further corroborated by DFT calculations (see Supplementary Tables 5–7). The complexes were optimized at the M06-2X/6-31 g(d,p) level of theory corrected with Grimme’s dispersion (D3)^38^. The optimized structure of **SC6** (Fig. 5a) shows the simultaneously cooperative Se···O and Se···N interactions confirmed by QTAIM analyses with interaction energy of −23.7 kcal/mol, while the complex **SC3** with a single Se···N interaction has weaker interaction energy of −20.6 kcal/mol. The relative Gibbs free energy for **SC6** is −8.3 kcal/mol which is lower than that of **SC3** (−5.9 kcal/mol), suggesting that the formation of **SC6** is more favorable and feasible. These calculated energies are in a reasonable range considering the literature precedents^32,39^. Natural bond orbital (NBO) analyses of **SC3** and **SC6** (Fig. 5b) further suggest that the Se···N interaction between **Ch5** and aziridine in **SC3** plays a significant role. In contrast, both the Se···O and Se···N interactions between **Ch5** and aziridine in **SC6** play important roles.

### Chalcogen bonding catalysis

**Fig. 6:**
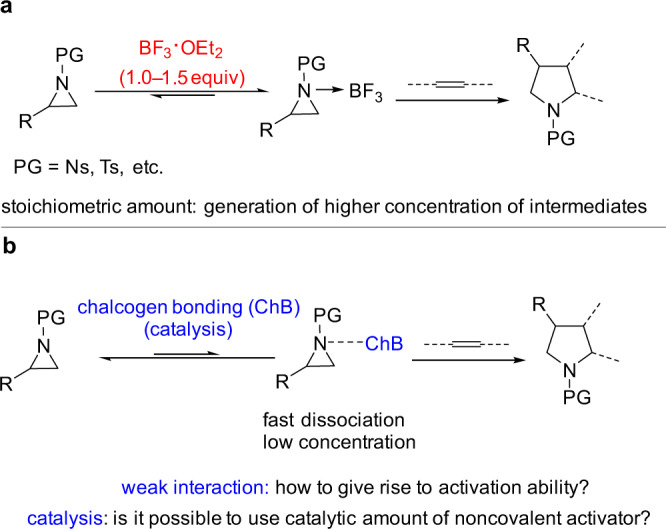
Shifting stoichiometric strong Lewis acid approach to chalcogen bonding catalysis. **a** Stoichiometric strong Lewis acid approach. **b** A challenging proposal: catalysis by weak interactions.

The reactions between weakly bonded supramolecular species and nonactivated alkenes are considered unfavorable transformations^1–10^. Therefore, to apply the bonding property of the aziridine-selenide complex, the cycloaddition of aziridines with nonactivated alkenes was selected as a target reaction as literature reports indicate that it would be a tough challenge for a weak interaction to promote this reaction (Fig. 6)^40–43^. To generate reactivity, the reported precedent used strong Lewis acid BF_3_·OEt_2_ to promote this reaction (Fig. 6a)^40,41^. Moreover, in order to generate a proper concentration of intermediate suitable for trapping with nonactivated alkenes, a stoichiometric amount of BF_3_·OEt_2_ was used. Otherwise, the reaction has to be carried out under a thermal-driven condition (100 °C) to give reactivity in the presence of a transition-metal catalyst^42,43^. To achieve this reaction by a weak interaction, there are two challenging problems (Fig. 6b). As the weak interaction provides much less activation than the strong Lewis acid counterpart, one problem is thus how a weak interaction with aziridine can give rise to activation ability. Furthermore, in contrast to the strong Lewis acid counterpart, the weak interaction always results in the generation of an unstable supramolecular complex with low concentration. Therefore, the other glaring problem is how to increase the concentration of aziridine-mediated supramolecular species to a reactive level by only using a catalytic amount of noncovalent activator. In this context, the chalcogen bonding approach was applied to address these problems.

**Fig. 7:**
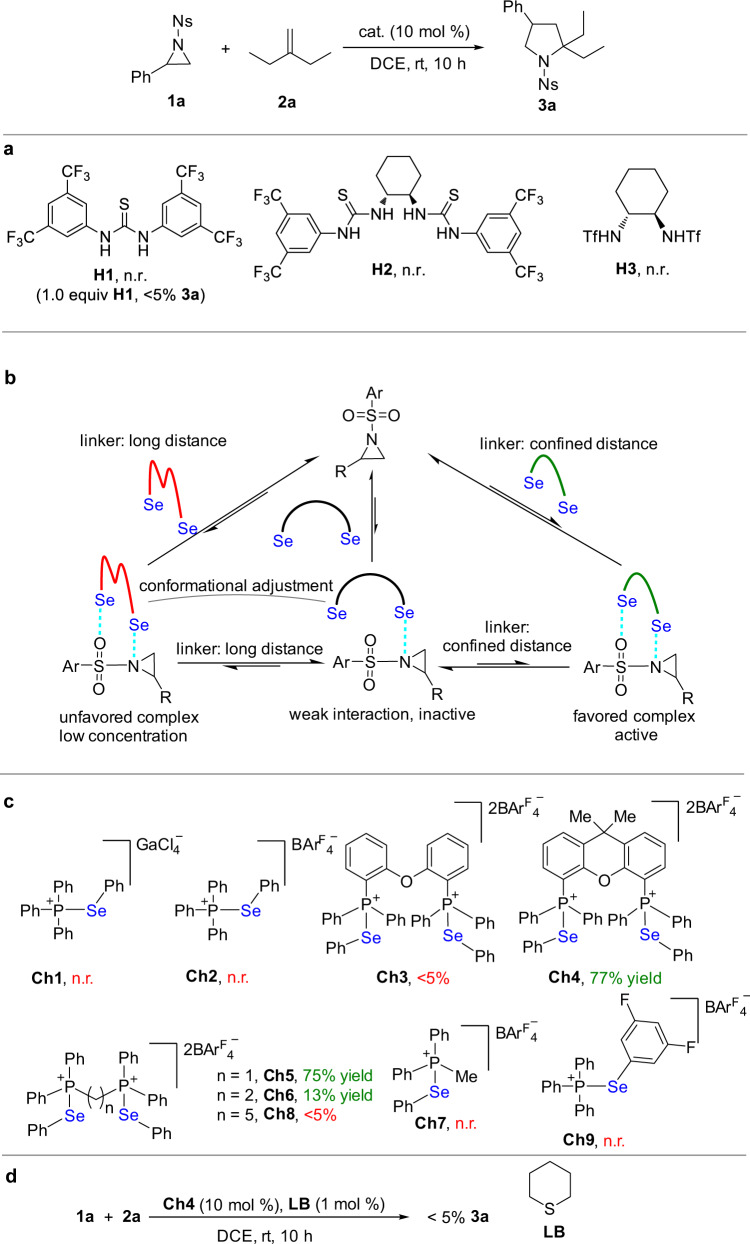
Noncovalent catalysis approach to cycloaddition of aziridine 1a with nonactivated alkene 2a. **a** Catalysis with typical hydrogen-bonding donors. **b** The interactions between bidentate chalcogen bonding donors and phenylsulfonyl protected aziridines. **c** Chalcogen bonding catalysis approach to cycloaddition of aziridines with nonactivated alkenes. **d** Inhibition experiment. DCE 1, 2-dichloroethane.

Initially, to evaluate the reactivity of this cycloaddition process towards activation by noncovalent interactions, the well-established hydrogen-bonding donors **H1**-**3** were employed as references (Fig. 7a). There was no background reaction even after 24 h in the absence of a catalyst. In the presence of 10 mol % of a typical hydrogen-bonding donor (**H1**-**3**), the reaction did not work. Furthermore, only a trace amount of product **3a** (<5%) was obtained using a stoichiometric amount of a hydrogen-bonding catalyst **H1** (1.0 equiv), indicating inertness of this reaction under activation by weak interactions. Considering the poor reactivity of this cycloaddition reaction, we envisioned that cooperative chalcogen–bonding interactions with aziridine would enhance the activation ability and provide more stabilization of the supramolecular species, thus improving the reactivity. However, to match the confined Lewis basic binding sites in phenylsulfonyl protected aziridines, the judicious use of bidentate chalcogen bonding donors that are capable of precisely recognizing the proper Lewis basic sites of aziridine is critically important to give catalytic activity (Fig. 7b).

In consistence with the results in Figs. 2, 4, upon using monodentate chalcogen bonding donors **Ch1**-**2** as catalysts, the reaction did not work. Nonetheless, these experiments revealed that neither the phosphonium unit nor the BAr^F^_4_ˉ counterion is catalytically active (Fig. 7c). To implement the idea as depicted in Fig. 7b, two sets of bidentate catalysts were investigated. One set is catalyst **Ch3** with a free rotation linker and its rigidified counterpart **Ch4**. The other set is catalysts **Ch5**-**7** with different lengths of linkers. The comprehensive control experiments (see Supplementary Figs. 15–22) indicate **Ch3** bearing a long linker with free rotation between two chalcogen bonding sites operates like a monodentate catalyst. In consistence with these observations, the experimental results revealed that catalyst **Ch3** with a long and free rotation linker gave no catalytic activity. Then the free rotation linker of catalyst **Ch3** was rigidified. Upon changing catalyst **Ch3** to catalyst **Ch4** with a rigid backbone, the reaction began to work and catalyst **Ch4** showed high catalytic activity to give a 77% yield of product **3a** at room temperature. Analysis of the mixture of **Ch4** and olefin **2a** by ^77^Se and ^31^P NMR indicated that there was no detectable variation of the ^77^Se and ^31^P signals, indicating there is no competitive bonding between catalysts **Ch4** and **2a** (see Supplementary Fig. 23).

Further investigation of the catalytic performance of the bidentate catalysts **Ch5**-**6** suggests the distance between the two binding sites is critically important. Upon linking the two phosphonium units with one carbon, catalyst **Ch5** exhibited good catalytic activity to give a 75% yield of **3a**, indicating this distance between the two binding sites can well interact with the proper Lewis basic sites of aziridine. In contrast to **Ch5**, even only increasing the length of the linker by one carbon, catalyst **Ch6** showed poor catalytic activity and only a 13% yield of **3a** was obtained. In contrast, monodentate catalyst **Ch7** had no catalytic activity. Similar to the case of **Ch3**, upon linking the two phosphonium units with five carbons, catalyst **Ch8** with improperly positioned two binding sites completely lost catalytic activity. Catalyst **Ch9** with electron-withdrawing substituents on the aryl ring did not show catalytic activity. For the same selenide, the strength of chalcogen bonding interaction is determined by the electron-donating ability of aziridines. Therefore, toluenesulfonyl-protected aziridine in principle generates stronger chalcogen bonding interactions than nitrobenzenesulfonyl-protected aziridine. In line with this notion, analysis of a mixture of *N*-(*p*-toluenesulfonyl)aziridine (**1b**, i.e., Ns in **1a** was replaced by Ts) and 10 mol% selenide **Ch5** in DCE by TLC revealed that **1b** was fully decomposed after only 80 s at room temperature, which was further supported by ^1^H NMR analysis of this mixture. In sharp contrast, for a reaction mixture of *N*-(*p*-nitrobenzenesulfonyl)aziridine **1a** and 10 mol% selenide **Ch5** in DCE, 76% **1a** remained unchanged after 80 s. To corroborate these observations, further analysis of a 1:1 competitive mixture of **1a** and **1b** in the presence of 10 mol% selenide **Ch5** in CD_2_Cl_2_ indicated that **1b** was fully decomposed while 81% of **1a** remained unchanged in this reaction mixture after 80 s. Therefore, in order to suppress side reaction pathways, nitrobenzenesulfonyl protecting group is an optimal choice.

Since the weak nature of chalcogen bonding interactions restricts both the reactivity and the concentration of equilibrating aziridine-selenide complex, the control experiment (Fig. 7d) demonstrate that only the addition of 1 mol% tetrahydrothiopyran almost resulted in complete inhibition of the reaction even in the presence of 10 mol% catalyst **Ch4** (<5% **3a**). In line with the analysis as depicted in Fig. 6b, this experiment suggests that only a very low concentration of complex **SC6** was generated and it is susceptible to completive bonding.

**Fig. 8:**
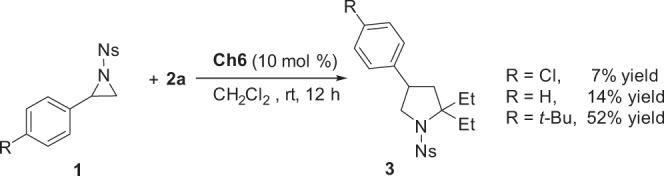
Control experiments on the catalytic performance of Ch6 by using aziridines with different electronic properties. Reaction conditions: the reactions were carried out by using aziridine **1** (0.1 mmol), **2a** (0.3 mmol, 25.2 mg), **Ch6** (0.01 mmol, 10 mol %, 24.4 mg) in CH_2_Cl_2_ (1.5 mL) at room temperature under argon for 12 h.

While the experiments in Fig. 4 indicate catalyst **Ch6** could act as an effective bidentate chalcogen bonding donor to form complex **SC6**, however, **Ch6** is less efficient to activate electron-deficient Ns-protected aziridines in contrast to its counterpart **Ch4** or **Ch5**. On the other hand, comparable results on the interactions of more electron-rich aziridines **m1**-**2** with **Ch5** and **Ch6** were observed (Fig. 4b), indicating the interaction of **Ch6** and aziridine is affected by the electronic effect of aziridines. Based on these observations, the contrast experiments on the catalytic performance of **Ch6** in CH_2_Cl_2_ using aziridines with different electronic properties were carried out (Fig. 8). The control experiments using *p*-*t*-BuPh, *p*-ClPh, and Ph substituted aziridines as substrates showed distinct results (yield: 52% for *p*-*t*-BuPh; 14% for Ph; 7% for *p*-ClPh).

**Fig. 9:**
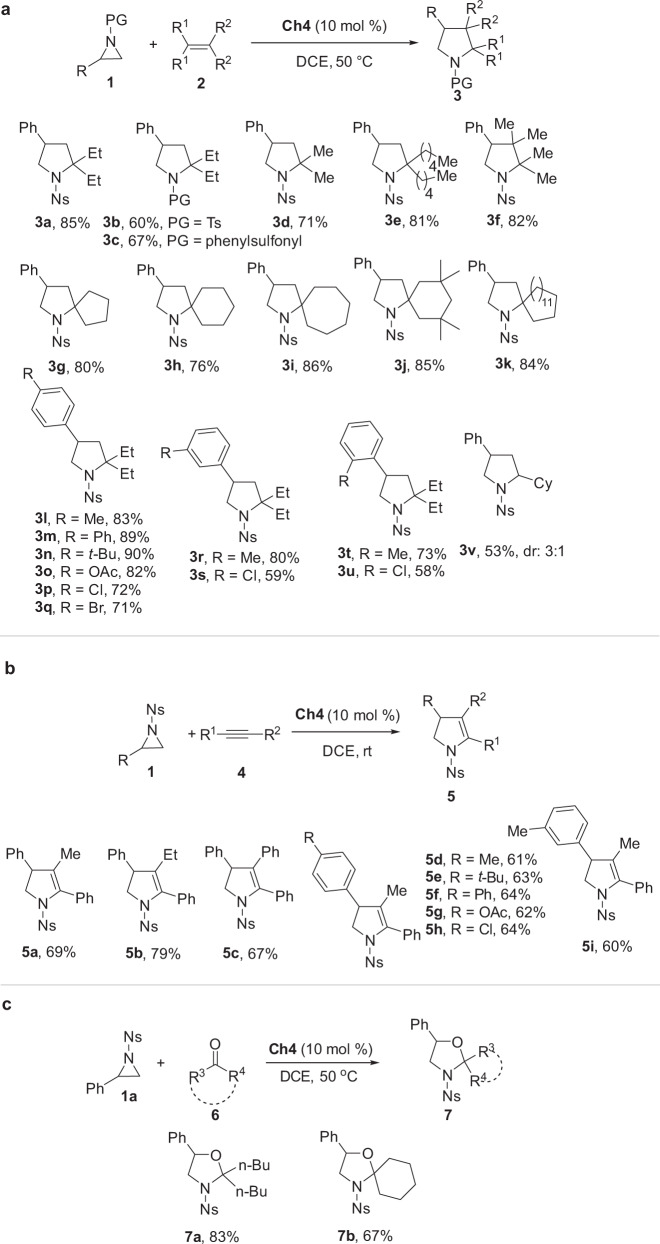
Reaction scope. **a** Reactions between aziridines and alkenes. **b** Reactions between aziridines and alkynes. **c** Reactions between aziridines and ketones.

As shown in Fig. 9a, the reaction time can be shortened from 10 to 1 h while the yield of product **3a** was improved from 77 to 85% upon conducting the reaction at 50 °C. Tracing the reaction system by ^31^P NMR reveals that catalyst **Ch4** is stable in this reaction process and only the ^31^P signal of catalyst **Ch4** was observed (see Supplementary Fig. 2). Different protecting groups can be tolerated in this reaction to give products **3b** and **3c** in 60 and 67% yields, respectively. Terminal olefins with different chain length showed similar reactivity. A tetrasubstituted olefin was highly reactive to give pyrrolidine **3f** in an 82% yield. Cycloalkyl-substituted olefins were used to give different spiro-pyrrolidines **3g**–**k** in good yields. Regardless of the electronic characteristic of the substituents on the aromatic ring as well as the positions where the substitution groups are located, aziridines bearing different aromatic rings were well tolerated to give pyrrolidines **3l**–**u** with reasonable yields. Mono-substituted nonactivated alkene was proven to be an effective reactant, and product **3v** was obtained in 53% yield (dr: 3:1). Furthermore, alkynes were also effective substrates^44^, and products **5a**-**5i** were obtained in 60–79% yields (Fig. 9b). Upon using ketones as substrates^44^, products **7a** and **7b** were obtained in 83 and 67% yields, respectively (Fig. 9c).

In summary, this manuscript reports the chalcogen bonding of aziridine-selenide complexes and establishes a type of reaction between weakly bonded supramolecular species and nonactivated alkenes, thus shifting the stoichiometric strong Lewis acid-mediated approach to a noncovalent catalysis manner. Experimental results revealed that the bidentate catalysts with confined binding distance enable the simultaneous formation of Se∙∙∙O and Se∙∙∙N interactions with aziridines, which enables their use in catalysis. This research opens up opportunities for addressing problems relative to activation targets and reaction patterns in supramolecular catalysis.

## Methods

### General procedure for the preparation of bidentate chalcogen bonding catalysts Ch3-6 and Ch8

To a red solution of PhSeCl (383.0 mg, 2.0 mmol) in dry Et_2_O (6.0 mL) at 0 °C under argon was added TMSOTf (444.5 mg, 2.0 mmol). The reaction mixture was allowed to warm up to room temperature and stirred for 40 min to give a dark orange solution. Then 1.0 mmol phosphine ([oxybis(2,1-phenylene)]bis(diphenylphosphine) for **Ch3**; (9,9-dimethyl-9H-xanthene-4,5-diyl)bis(diphenylphosphine) for **Ch4**; bis(diphenylphosphino)methane for **Ch5**; 1,2-bis(diphenylphosphino)ethane for **Ch6**; 1,5-bis(diphenylphosphino)pentane for **Ch8**) dissolved in dry CH_2_Cl_2_ (4.0 mL) was added over 5 min at 0 °C. The reaction mixture was allowed to warm up to room temperature and stand for 1 h. The white solid suspension was filtered and washed by anhydrous diethyl ether. Then sodium tetrakis[3,5-bis(trifluoromethyl)phenyl]borate (1772.4 mg, 2.0 mmol) was added to a solution of the above white solid (1.0 mmol) in dry CH_2_Cl_2_ (10.0 mL) under argon and the reaction mixture was stirred at room temperature for 24 h. Then the reaction mixture was filtered and the filtrate was concentrated to give a saturated solution under reduced pressure and then 10.0 mL *n*-hexane was slowly added. The two-phase solution was placed at room temperature under argon and the desired product precipitated out as a white solid. Then the precipitated white solid was collected by filtration and recrystallized twice from CH_2_Cl_2_ (or ether) and *n*-hexane to afford the pure catalyst.

### General procedure for the preparation of monodentate chalcogen bonding catalysts Ch2, Ch7, and Ch9

To a red solution of PhSeCl (191.5 mg, 1.0 mmol for catalysts **Ch2** and **Ch7**) or 3,5-F_2_C_6_H_3_SeCl (227.9 mg, 1.0 mmol for catalyst **Ch9**) in dry Et_2_O (6.0 mL) at 0 °C under argon was added TMSOTf (222.3 mg, 1.0 mmol). The reaction mixture was allowed to warm up to room temperature and stirred for 40 min to give a dark orange solution. Then 1.0 mmol phosphine (triphenylphosphine for **Ch2** and **Ch9**; methyldiphenylphosphine for **Ch7**) dissolved in dry CH_2_Cl_2_ (4.0 mL) was added over 5 min at 0 °C. The reaction mixture was allowed to warm up to room temperature and stand for 1 h. Then sodium tetrakis[3,5-bis(trifluoromethyl)phenyl]borate (886.2 mg, 1.0 mmol) was added to the above reaction system and stirred at room temperature for 24 h. Then the reaction mixture was filtered and the filtrate was concentrated to give a saturated solution under reduced pressure and then 10.0 mL *n*-hexane was slowly added. The two-phase solution was placed at room temperature under argon and the desirable product precipitated out as a white solid. Then the precipitated white solid was collected by filtration and recrystallized twice from CH_2_Cl_2_ and *n*-hexane to afford the pure catalyst.

### General procedures for chalcogen bonding catalysis of cycloaddition reactions

To a reaction mixture of aziridine **1** (0.2 mmol) and catalyst **Ch4** (10 mol %, 52.4 mg, 0.02 mmol) in a 10-mL-Schlenk tube was added DCE (1.0 mL) under argon atmosphere. Then the corresponding reactant alkene **2** or alkyne **4** or ketone **6** (0.6 mmol, 3.0 equiv) was added to the above reaction mixture. The reaction was stirred at indicated temperature (50 °C for the reactions to generate products **3** and **7**; room temperature for the reactions to generate product **5**) until the completion of the reaction as judged by TLC analysis. Then the solvent was removed under reduced pressure and the residue was purified by flash chromatography on silica gel using petroleum ether/ethyl acetate (v/v = 30:1 to 10:1) as eluent to give the desired products.

## Supplementary information

Supplementary Information

## Data Availability

All the data supporting the findings of this study are available within the article and its Supplementary Information file. The X-ray crystallographic data for structures reported in this study have been deposited in the Cambridge Crystallographic Data Centre under deposition numbers 2069229 (**Ch3**), 2069224 (**Ch5**), and 2069228 (**Ch6**). These data can be obtained free of charge from The Cambridge Crystallographic Data Centre.
