# Supplementary material for: Cooperative chalcogen bonding interactions in confined sites activate aziridines
Source: Nat Commun. 2022 Jun 22;13:3563. doi: 10.1038/s41467-022-31293-5 (PMC9217929; doi:10.1038/s41467-022-31293-5)
Supplement: Supplementary file 1 — Supplementary Information [file 41467_2022_31293_MOESM1_ESM.pdf]

Supplementary Information for

Cooperative Chalcogen Bonding Interactions in Confined Sites Activate Aziridines

Haofu Zhu[1], Pan-Pan Zhou[2*], Yao Wang[1*]

[1]School of Chemistry and Chemical Engineering, Key Laboratory of the Colloid and Interface Chemistry, Ministry of Education, Shandong University, Jinan, 250100, China

[2]Key Laboratory of Advanced Catalysis of Gansu Province, College of Chemistry and Chemical Engineering, Lanzhou University, Lanzhou, 730000, China

*Corresponding author. Email: zhoupp@lzu.edu.cn; yaowang@sdu.edu.cn

Table of Contents:

1. General Information S2

2. Preparation of Catalysts S2

3. Optimization of Reaction Condition S9

4. Optimized Procedure......S10

5. Analytical Data for Products S11

6. Mechanistic Study......S21

7. DFT Calculations S45

8. X-ray Crystallographic Data S54

9. Powder X-ray Diffraction (PXRD) S57

10. Copies of NMR Spectra S59

11. Supplementary References......S112

1. General Information

All the chemicals were either purchased from commercial suppliers or purified by standard procedures as specified in *Purification of Laboratory Chemicals*, 7th Ed (Armarego, W. L. F.; Chai, C. L. L. Butterworth Heinemann: 2013). All reactions were carried out under argon atmosphere. Analytical thin-layer chromatography (TLC) was performed on silica gel plates and analyzed by UV light or by potassium permanganate stains followed by heating. Flash chromatography was carried out utilizing silica gel (200-300 mesh). NMR spectra were recorded in $CDCl_3$ or CD_2Cl_2 at 298 K on a Bruker AM-400 spectrometer. The chemical shifts are reported in ppm relative to either the residual solvent peak (^{13}C) (δ = 77.00 ppm for $CDCl_3$; δ = 53.84 ppm for CD_2Cl_2), (^1H) (δ = 5.32 ppm for CD_2Cl_2, δ = 0 ppm for TMS) as an internal standard or using PhSeSePh as a reference compound (^{77}Se) (δ = 461.57 ppm for PhSeSePh) as an external standard. Data for ^1H NMR are reported as follows: chemical shift (δ ppm), multiplicity (s = singlet, d = doublet, t = triplet, m = multiplet, dd = doublet doublet), coupling constant (Hz), integration. Data for ^{13}C NMR are reported as chemical shift. HRMS were performed on a Bruker Apex II mass instrument (ESI). Powder X-ray diffraction (PXRD) patterns were obtained on a SmartL ab 9KW, using Cu Kα (λ = 1.5418 Å) radiation.

2. Preparation of Catalysts

Catalysts **Ch1-9** as depicted below were evaluated in this work. Catalyst **Ch1** was prepared according to the literature procedure [1]; Catalysts **Ch2-9** were prepared using the general procedure.

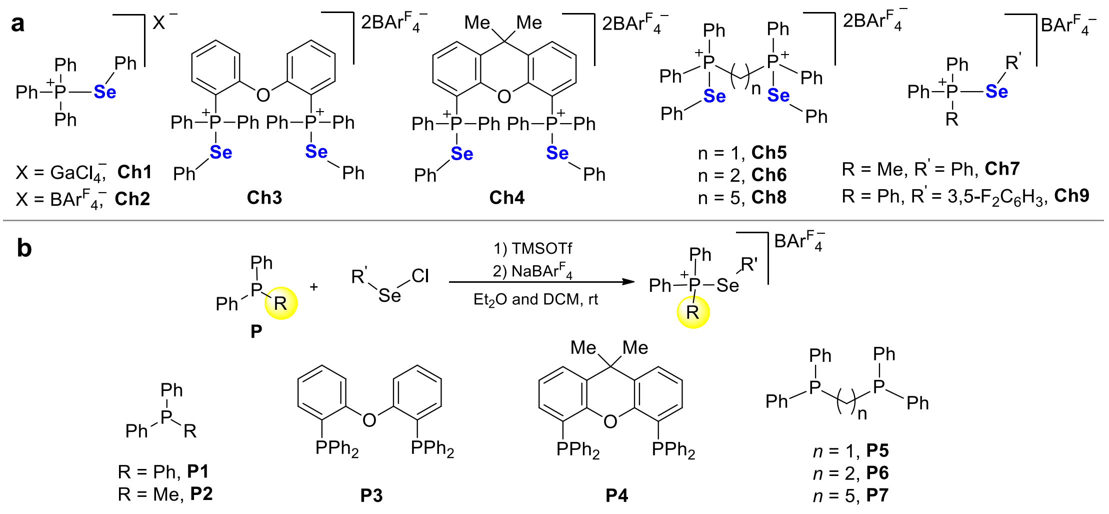

Supplementary Figure 1. Preparation of catalysts. a Chalcogen bonding catalysts. **b** General procedure for catalyst synthesis.

Experiment and analytical data for catalysts

Preparation of Ch1: PhSeCl (191.52 mg, 1.0 mmol) was added to a solution of $GaCl_3$ (176.08 mg, 1.0 mmol) in dry DCM (6.0 mL) and the reaction mixture was stirred for 5 minutes to give a dark red solution. Then triphenylphosphine (**P1**, 262.29 mg, 1.0 mmol) was added to the above reaction mixture and the reaction was run for 1 h to generate a colourless solution. Subsequently, the reaction mixture was concentrated to give a saturated solution (~3 mL DCM) under reduced pressure and then 10.0 mL n-hexane was slowly added. The two-phase solution was then placed for 2 h at room temperature under argon to give white solid. The precipitate was filtered off and washed by anhydrous diethyl ether to afford pure **Ch1** as a white solid (91% yield, 571.3 mg). **Ch1** is a known compound and the spectroscopic data are consistent with those reported in the literature [1].

Preparation of Ch2: To a red solution of PhSeCl (191.52 mg, 1.0 mmol) in dry DCM (6.0 mL) at 0 °C under argon was added TMSOTf (222.26 mg, 1.0 mmol). The reaction mixture was allowed to room temperature and stirred for 40 minutes to give a dark orange solution. Then triphenylphosphine (**P1**, 262.29 mg, 1.0 mmol) in dry DCM (4.0 mL) was added over 5 minutes at 0 °C. The reaction mixture was allowed to room temperature and stirred for 1 h. Then sodium tetrakis[3,5-bis(trifluoromethyl)phenyl]borate (886.21 mg, 1.0 mmol) was added to the above reaction system and stirred at room temperature for 24 h. Then the reaction mixture was filtered and the filtrate was concentrated to give a saturated solution under reduced pressure and then 10.0 mL *n*-hexane was slowly added. The two-phase solution was then placed at room temperature under argon and the desirable product precipitates out as a white solid. Then the precipitated white solid was collected by filtration and recrystallized twice from DCM and n-hexane to afford pure catalyst **Ch2** (80% yield, 1028.2 mg).

Ch2: White solid. ^{1}H NMR (400 MHz, 298K, CD_2Cl_2) δ 7.80–7.78 (m, 2H), 7.76–7.75 (m, 9H), 7.63–7.58 (m, 6H), 7.56–7.54 (m, 6H), 7.52–7.48 (m, 4H), 7.47–7.43 (m, 1H), 7.26–7.20 (m, 4H); ^{13}C NMR (100 MHz, 298K, CD_2Cl_2) δ 162.20 (q, J = 49.5 Hz), 138.13 (d, J = 3.3 Hz), 136.39 (d, J =3.3 Hz), 135.23 (bs), 134.22 (d, J = 10.7 Hz), 132.50 (d, J = 3.4 Hz), 131.26 (d, J = 2.8 Hz), 130.91 (d, J = 13.5 Hz), 129.33 (qq, J = 31.3, 2.9 Hz), 125.01 (q, J = 270.8 Hz), 119.21 (d, J = 7.2 Hz), 118.65 (d, J = 77.5 Hz), 118.01–117.82 (m); ^{31}P NMR (162 MHz, 298K, CD_2Cl_2) δ 37.53; ^{77}Se NMR (76 MHz, 298K, CD_2Cl_2) δ 313.01 (d, $J_{\text{Se-P}}$ = 454.32 Hz); ^{19}F NMR (376 MHz, 298K, CD_2Cl_2) δ -62.78; HRMS (ESI+) exact mass calculated for $[M]^+$ ($C_{24}H_{20}PSe$) requires m/z 419.0462, found m/z 419.0458.

Preparation of Ch3. To a red solution of PhSeCl (383.04 mg, 2.0 mmol) in dry Et_2O (6.0 mL) at 0 °C under argon was added TMSOTf (444.52 mg, 2.0 mmol). The reaction mixture was allowed to room temperature and stirred for

40 minutes to give a dark orange solution. Then [oxybis(2,1-phenylene)]bis(diphenylphosphine) (**P3**, 538.57 mg, 1.0 mmol) in dry DCM (4.0 mL) was added over 5 minutes at 0 °C. The reaction mixture was allowed to room temperature and stirred for 1 h. The white solid suspension was filtered and washed by anhydrous diethyl ether. Then sodium tetrakis[3,5-bis(trifluoromethyl)phenyl]borate (1772.42 mg, 2.0 mmol) was added to a solution of the above white solid (1149.97 mg, 1.0 mmol) in dry DCM (10.0 mL) under argon and the reaction mixture was stirred at room temperature for 24 h. Then the reaction mixture was filtered and the filtrate was concentrated to give a saturated solution under reduced pressure and then 10.0 mL *n*-hexane was slowly added. The two-phase solution was then placed at room temperature under argon and the desirable product precipitates out as a white solid. Then the precipitated white solid was collected by filtration and recrystallized twice from DCM and n-hexane to afford pure catalyst **Ch3** (79% yield, 2036.9 mg).

Ch3: White solid. ^{1}H NMR (400 MHz, 298K, CD_2Cl_2) δ 7.80 (s, 16H), 7.75–7.65 (m, 8H), 7.59 (s, 8H), 7.51–7.44 (m, 10H), 7.42–7.31 (m, 6H), 7.23 (dd, *J* = 14.4, 8.2 Hz, 4H), 7.13–7.10 (m, 8H), 6.68–6.64 (m, 2H); ^{13}C NMR (100 MHz, 298K, CD_2Cl_2) δ 162.29 (q, *J* = 49.5 Hz), 158.91 (d, *J* = 2.3 Hz), 139.08 (d, *J* = 2.3 Hz), 137.91 (d, *J* = 3.7 Hz), 136.57 (dd, *J* = 23.8, 3.3 Hz), 135.60 (d, *J* = 7.0 Hz), 135.31 (bs), 134.22 (dd, *J* = 35.9, 10.9 Hz) , 132.50 (d, *J* = 3.6 Hz), 131.17 (d, *J* = 3.2 Hz), 131.02 (d, *J* = 1.4 Hz), 130.89, 129.42 (qq, *J* = 31.3, 2.8 Hz), 127.72 (d, *J* = 12.0 Hz), 125.09 (q, *J* = 270.8 Hz), 121.86 (d, *J* = 6.6 Hz), 119.87 (d, *J* = 7.7 Hz), 118.05–117.94 (m), 116.86 (d, *J* = 78.7 Hz), 113.02 (d, *J* = 78.9 Hz); ^{31}P NMR (162 MHz, 298K, CD_2Cl_2) δ 32.81; ^{77}Se NMR (76 MHz, 298K, CD_2Cl_2) δ 335.14 (d, $J_{Se\text{-}P}$ = 475.54 Hz); ^{19}F NMR (376 MHz, 298K, CD_2Cl_2) δ -62.71; HRMS (ESI+) exact mass calculated for $[M]^{2+}$ ($C_{48}H_{38}OP_2Se_2$) requires m/z 426.0359, found m/z 426.0364.

Preparation of Ch4. To a red solution of PhSeCl (383.04 mg, 2.0 mmol) in dry Et_2O (6.0 mL) at 0 °C under argon was added TMSOTf (444.52 mg, 2.0 mmol). The reaction mixture was allowed to room temperature and stirred for 40 minutes to give a dark orange solution. Then 4,5-bis(diphenylphosphino)-9,9-dimethylxanthene (**P4**, 578.63 mg, 1.0 mmol) in dry DCM (4.0 mL) was added over 5 minutes at 0 °C. The reaction mixture was allowed to room temperature and stirred for 1 h. The white solid suspension was filtered and washed by anhydrous diethyl ether. Then sodium tetrakis[3,5-bis(trifluoromethyl)phenyl]borate (1772.42 mg, 2.0 mmol) was added to a solution of the above white solid (1190.01 mg, 1.0 mmol) in dry DCM (10.0 mL) under argon and the reaction mixture was stirred at room temperature for 24 h. Then the reaction mixture was filtered and the filtrate was concentrated to give a saturated solution under reduced pressure and then 10.0 mL *n*-hexane was slowly added. The two-phase solution was then placed at room temperature under argon and the desirable product precipitates out as a white solid. Then the

precipitated white solid was collected by filtration and recrystallized twice from Et_2O and n-hexane to afford pure catalyst **Ch4** (81% yield, 2126.9 mg).

Ch4: White solid. ^{1}H NMR (400 MHz, 298K, CD_2Cl_2) δ 8.12 (d, *J* = 7.9 Hz, 2H), 7.88–7.81 (m, 16H), 7.63 (s, 9H), 7.60–7.46 (m, 3H), 7.51–7.46 (m, 8H), 7.43–7.38 (m, 12H), 7.19 (t, *J* = 7.8 Hz, 6H), 7.08–7.06 (m, 4H), 1.90 (s, 6H); ^{13}C NMR (100 MHz, 298K, CD_2Cl_2) δ 162.25 (q, *J* = 49.5 Hz), 152.90 (d, *J* = 2.5 Hz), 137.61 (d, *J* = 3.6 Hz), 137.17 (d, *J* = 8.0 Hz), 136.83 (d, *J* = 2.6 Hz), 136.70 (d, *J* = 3.3 Hz), 135.29 (bs), 134.40 (d, *J* = 10.7 Hz), 133.73 (d, *J* = 6.7 Hz), 132.76 (d, *J* = 3.5 Hz), 131.30 (d, *J* = 2.9 Hz), 131.13, 130.99, 129.41 (qq, *J* = 31.3, 2.8 Hz), 126.63 (d, *J* = 13.2 Hz), 125.06 (q, *J* = 270.7 Hz), 120.68 (d, *J* = 8.3 Hz), 119.60 (d, *J* = 78.4 Hz), 118.02–117.91 (m), 105.35 (d, *J* = 77.5 Hz), 35.25, 33.74; ^{31}P NMR (162 MHz, 298K, CD_2Cl_2) δ 30.99; ^{77}Se NMR (76 MHz, 298K, $CDCl_3$) δ 342.73 (d, $J_{Se\text{-}P}$ = 480.55 Hz); ^{19}F NMR (376 MHz, 298K, $CDCl_3$) δ -60.80; HRMS (ESI+) exact mass calculated for $[M]^{2+}$ ($C_{51}H_{42}OP_2Se_2$) requires m/z 446.0515, found m/z 446.0520.

Preparation of Ch5. To a red solution of PhSeCl (383.04 mg, 2.0 mmol) in dry Et_2O (6.0 mL) at 0 °C under argon was added TMSOTf (444.52 mg, 2.0 mmol). The reaction mixture was allowed to room temperature and stirred for 40 minutes to give a dark orange solution. Then bis(diphenylphosphino)methane (**P5**, 384.40 mg, 1.0 mmol) in dry DCM (4.0 mL) was added over 5 minutes at 0 °C. The reaction mixture was allowed to room temperature and stirred for 1 h. The white solid suspension was filtered and washed by anhydrous diethyl ether. Then sodium tetrakis[3,5-bis(trifluoromethyl)phenyl]borate (1772.42 mg, 2.0 mmol) was added to a solution of the above white solid (995.94 mg, 1.0 mmol) in dry DCM (10.0 mL) under argon and the reaction mixture was stirred at room temperature for 24 h. Then the reaction mixture was filtered and the filtrate was concentrated to give a saturated solution under reduced pressure and then 10.0 mL *n*-hexane was slowly added. The two-phase solution was then placed at room temperature under argon and the desirable product precipitates out as a white solid. Then the precipitated white solid was collected by filtration and recrystallized twice from DCM and n-hexane to afford pure catalyst **Ch5** (65% yield, 1575.8 mg).

Ch5: White solid. ^{1}H NMR (400 MHz, 298K, CD2Cl2) δ 7.84–7.82 (m, 3H), 7.78 (s, 19H), 7.58 (s, 16H), 7.49–7.44 (m, 8H), 7.34 (t, J = 7.7 Hz, 2H), 7.09 (t, J = 7.8 Hz, 4H), 6.98 (d, J = 7.6 Hz, 3H), 4.58 (t, J = 13.5 Hz, 2H); ^{13}C NMR (100 MHz, 298K, CD_2Cl_2) δ 162.24 (q, *J* = 49.5 Hz), 138.49, 137.58, 135.27 (bs), 133.71 (d, *J* = 5 Hz), 133.63, 133.31, 132.04, 131.98, 131.89 (d, *J* = 16.9 Hz), 129.36 (qq, *J* = 31.3, 2.9 Hz), 125.06 (q, *J* = 270.8 Hz), 118.02–117.95 (m), 114.77 (d, *J* = 76.4 Hz), 28.66 (t, *J* = 37.3 Hz); ^{31}P NMR (162 MHz, 298K, CD_2Cl_2) δ 30.27; ^{77}Se NMR (76 MHz, 298K, CD_2Cl_2) δ 312.64 (d, $J_{Se\text{-}P}$ = 487.41 Hz); ^{19}F NMR (376 MHz, 298K, CD_2Cl_2) δ -62.66;

HRMS (ESI+) exact mass calculated for $[M]^{2+}$ ($C_{37}H_{32}P_2Se_2$) requires m/z 349.0149, found m/z 349.0153.

Preparation of Ch6. To a red solution of PhSeCl (383.04 mg, 2.0 mmol) in dry Et_2O (6.0 mL) at 0 °C under argon was added TMSOTf (444.52 mg, 2.0 mmol). The reaction mixture was allowed to room temperature and stirred for 40 minutes to give a dark orange solution. Then 1,2-bis(diphenylphosphino)ethane (**P6**, 384.4 mg, 1.0 mmol) in dry DCM (4.0 mL) was added over 5 minutes at 0 °C. The reaction mixture was allowed to room temperature and stirred for 1 h. The white solid suspension was filtered and washed by anhydrous diethyl ether. Then sodium tetrakis[3,5-bis(trifluoromethyl)phenyl]borate (1772.42 mg, 2.0 mmol) was added to a solution of the above white solid (995.94 mg, 1.0 mmol) in dry DCM (10.0 mL) under argon and the reaction mixture was stirred at room temperature for 24 h. Then the reaction mixture was filtered and the filtrate was concentrated to give a saturated solution under reduced pressure and then 10.0 mL *n*-hexane was slowly added. The two-phase solution was then placed at room temperature under argon and the desirable product precipitates out as a white solid. Then the precipitated white solid was collected by filtration and recrystallized twice from DCM and n-hexane to afford pure catalyst **Ch6** (76% yield, 1853.2 mg).

Ch6: White solid. ^{1}H NMR (400 MHz, 298K, CD_2Cl_2) δ 7.82 (t, *J* = 7.6 Hz, 4H), 7.77–7.76 (m, 16H), 7.63–7.58 (m, 8H), 7.56 (s, 8H), 7.45–7.36 (m, 10H), 7.14 (t, *J* = 7.8 Hz, 4H), 6.95 (d, *J* = 7.5 Hz, 4H), 2.79 (d, *J* = 3.7 Hz, 4H); ^{13}C NMR (100 MHz, 298K, CD_2Cl_2) δ 162.25 (q, *J* = 49.5 Hz), 137.91, 137.45, 135.23 (bs), 133.18 (d, *J* = 3.2 Hz), 133.09 (d, *J* = 5.3 Hz), 131.88, 131.78 (d, *J* = 6.7 Hz), 129.32 (qq, *J* = 31.3, 2.8 Hz), 125.00 (q, *J* = 270.8 Hz), 118.03–117.88 (m), 116.43, 115.37 (d, *J* = 74.9 Hz), 20.42 (d, *J* = 21.0 Hz); ^{31}P NMR (162 MHz, 298K, CD_2Cl_2) δ 40.00; ^{77}Se NMR (76 MHz, 298K, CD_2Cl_2) δ 275.20 (d, $J_{Se\text{-}P}$ = 468.92 Hz); ^{19}F NMR (376 MHz, 298K, CD_2Cl_2) δ -62.66; HRMS (ESI+) exact mass calculated for $[M]^{2+}$ ($C_{38}H_{34}P_2Se_2$) requires m/z 356.0228 found m/z 356.0232.

Preparation of Ch7. To a red solution of PhSeCl (191.52 mg, 1.0 mmol) in dry DCM (6.0 mL) at 0 °C under argon was added TMSOTf (222.26 mg, 1.0 mmol). The reaction mixture was allowed to room temperature and stirred for 40 minutes to give a dark orange solution. Then methyldiphenylphosphine (**P2**, 200.22 mg, 1.0 mmol) in dry DCM (4.0 mL) was added over 5 minutes at 0 °C. The reaction mixture was allowed to room temperature and stirred for 1 h. Then sodium tetrakis[3,5-bis(trifluoromethyl)phenyl]borate (886.21 mg, 1.0 mmol) was added to the above reaction system and stirred at room temperature for 24 h. Then the reaction mixture was filtered and the filtrate was concentrated to give a saturated solution under reduced pressure and then 10.0 mL *n*-hexane was slowly added. The two-phase solution was then placed at room temperature under argon and the desirable product precipitates out as a white solid. Then the precipitated white solid was collected by filtration and recrystallized twice from DCM and

n-hexane to afford pure catalyst **Ch7** (87% yield, 1061.2 mg).

Ch7: White solid. ^{1}H NMR (400 MHz, 298K, CD_2Cl_2) δ 7.82–7.77 (m, 10H), 7.66–7.60 (m, 12H), 7.51–7.46 (m, 1H), 7.36–7.28 (m, 4H), 2.51 (d, *J* = 13.3 Hz, 3H); ^{13}C NMR (100 MHz, 298K, CD_2Cl_2) δ 162.27 (q, *J* = 50.0 Hz), 137.82 (d, *J* = 3.4 Hz), 136.54 (d, *J* = 3.3 Hz), 135.30 (bs), 132.70 (dd, *J* = 20.2, 7.1 Hz), 131.45 (d, *J* = 2.9 Hz), 131.08 (d, *J* = 13.5 Hz), 129.40 (qq, *J* = 31.0, 3.0 Hz), 125.08 (q, *J* = 270.8 Hz), 119.57, 118.80, 118.40 (d, *J* = 7.1 Hz), 118.07–117.92 (m), 13.14 (d, *J* = 49.6 Hz); ^{31}P NMR (162 MHz, 298K, CD_2Cl_2) δ 33.08; ^{77}Se NMR (76 MHz, 298K, CD_2Cl_2) δ 307.70 (d, J Se-P = 443.06 Hz); ^{19}F NMR (376 MHz, 298K, CD_2Cl_2) δ -62.74; HRMS (ESI+) exact mass calculated for $[M]^+$ ($C_{19}H_{18}PSe$) requires m/z 357.0306, found m/z 357.0302.

Preparation of Ch8. To a red solution of PhSeCl (383.04 mg, 2.0 mmol) in dry Et_2O (6.0 mL) at 0 °C under argon was added TMSOTf (444.52 mg, 2.0 mmol). The reaction mixture was allowed to room temperature and stirred for 40 minutes to give a dark orange solution. Then 1,5-Bis(diphenylphosphino)pentane (**P7**, 440.51 mg, 1.0 mmol) in dry DCM (4.0 mL) was added over 5 minutes at 0 °C. The reaction mixture was allowed to room temperature and stirred for 1 h. The white solid suspension was filtered and washed by anhydrous diethyl ether. Then sodium tetrakis[3,5-bis(trifluoromethyl)phenyl]borate (1772.42 mg, 2.0 mmol) was added to a solution of the above white solid (1051.99 mg, 1.0 mmol) in dry DCM (10.0 mL) under argon and the reaction mixture was stirred at room temperature for 24 h. Then the reaction mixture was filtered and the filtrate was concentrated to give a saturated solution under reduced pressure and then 10.0 mL *n*-hexane was slowly added. The two-phase solution was then placed at room temperature under argon and the desirable product precipitates out as a white solid. Then the precipitated white solid was collected by filtration and recrystallized twice from DCM and n-hexane to afford pure catalyst **Ch8** (68% yield, 1686.7 mg).

Ch8: White solid. ^{1}H NMR (400 MHz, 298K, CD_2Cl_2) δ 7.79 (s, 16H), 7.73–7.70 (m, 4H), 7.58 (s, 9H), 7.56–7.51 (m, 10H), 7.48 (t, *J* = 6.8 Hz, 5H), 7.40 (t, *J* = 7.2 Hz, 2H), 7.20–7.12 (m, 8H), 2.72–2.69 (m, 4H), 1.60–1.57 (m, 6H); ^{13}C NMR (100 MHz, 298K, CD_2Cl_2) δ 162.25 (q, *J* = 49.5 Hz), 137.75, 136.64 (d, *J* = 3.2 Hz), 135.32 (bs), 133.27 (d, *J* = 10.3 Hz), 132.54, 131.37, 131.07 (d, *J* = 13.3 Hz), 129.42 (qq, *J* = 31.3, 2.9 Hz), 125.08 (q, *J* = 270.8 Hz), 118.06–117.98 (m), 117.68 (d, *J* = 6.6 Hz), 117.56 (d, *J* = 74.3 Hz), 31.66 (t, *J* = 18.3 Hz), 26.34, 25.91, 22.75–22.73 (m); ^{31}P NMR (162 MHz, 298K, CD_2Cl_2) δ 40.25; ^{77}Se NMR (76 MHz, 298K, CD_2Cl_2) δ 279.37 (d, $J_{Se\text{-}P}$ = 445.18 Hz); ^{19}F NMR (376 MHz, 298K, CD_2Cl_2) δ -62.67; HRMS (ESI+) exact mass calculated for $[M]^{2+}$ ($C_{41}H_{40}P_2Se_2$) requires m/z 377.0462, found m/z 377.0457.

Preparation of Ch9. To a red solution of 3,5-$F_2C_6H_3SeCl$ (227.91 mg, 1.0 mmol) in dry DCM (6.0 mL) at 0 °C under argon was added TMSOTf (222.26 mg, 1.0 mmol). The reaction mixture was allowed to room temperature and stirred for 40 minutes to give a dark orange solution. Then triphenylphosphine (**P1**, 262.29 mg, 1.0 mmol) in dry DCM (4.0 mL) was added over 5 minutes at 0 °C. The reaction mixture was allowed to room temperature and stirred for 1 h. Then sodium tetrakis[3,5-bis(trifluoromethyl)phenyl]borate (886.21 mg, 1.0 mmol) was added to the above reaction system and stirred at room temperature for 24 h. Then the reaction mixture was filtered and the filtrate was concentrated to give a saturated solution under reduced pressure and then 10.0 mL *n*-hexane was slowly added. The two-phase solution was then placed at room temperature under argon and the desirable product precipitates out as a white solid. Then the precipitated white solid was collected by filtration and recrystallized twice from DCM and n-hexane to afford pure catalyst **Ch9** (63% yield, 830.5 mg).

Ch9: White solid. ^{1}H NMR (400 MHz, 298K, CD_2Cl_2) δ 7.85 – 7.79 (m, 3H), 7.78 – 7.73 (m, 8H), 7.65 (td, *J* = 7.9, 4.3 Hz, 6H), 7.60 – 7.53 (m, 10H), 6.94 (tq, *J* = 8.6, 2.1 Hz, 1H), 6.84 – 6.77 (m, 2H); ^{13}C NMR (100 MHz, 298K, CD_2Cl_2) δ 163.55 (ddd, *J* = 255.7, 12.2, 3.3 Hz), 162.24 (q, *J* = 49.5 Hz), 136.91 (d, *J* = 3.3 Hz), 135.26 (bs), 134.24 (d, *J* = 10.8 Hz), 131.20 (d, *J* = 13.7 Hz), 129.36 (qq, *J* = 31.3, 2.9 Hz), 125.03 (q, *J* = 272.7 Hz), 121.34 (ddd, *J* = 27.1, 7.7, 3.3 Hz), 120.95 (dd, *J* = 13.3, 6.9 Hz), 118.32 – 117.90 (m), 117.88 (d, *J* = 77.7 Hz), 108.67 (td, *J* = 24.7, 3.2 Hz); ^{31}P NMR (162 MHz, 298K, CD_2Cl_2) δ 39.46; ^{77}Se NMR (76 MHz, 298K, CD_2Cl_2) δ 325.19 (d, $J_{\text{Se-P}}$ = 435.74 Hz); ^{19}F NMR (376 MHz, 298K, CD_2Cl_2) δ -62.77, -105.01 – -105.05 (m). HRMS (ESI+) exact mass calculated for $[M]^+$ ($C_{24}H_{18}F_2PSe$) requires m/z 455.0274, found m/z 455.0274.

3. Optimization of Reaction Condition

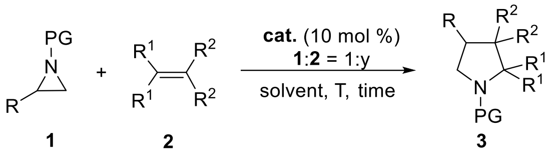

Supplementary Table 1. Evaluation of catalysts.

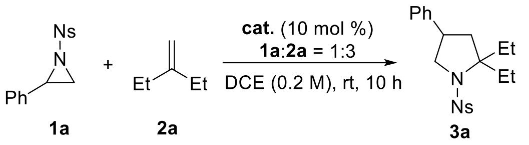

entry	cat.	yield (%)
1	vacant	n.r.
2	**Ch1**	n.r.
3	**Ch2**	n.r.
4	**Ch3**	n.r.
5	**Ch4**	77
6	**Ch5**	75
7	**Ch6**	13
8	**Ch7**	n.r.
9	**Ch8**	n.r.

Supplementary Table 2. Evaluation of PG.

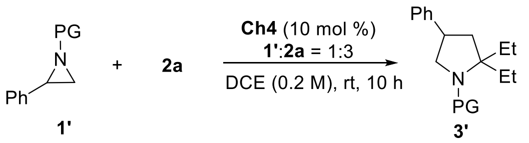

entry	PG	conversion (%)	yield (%)
1	Ts	>95	48
2	Ns	>95	77
3	$-S(O_2)Ph$	>95	52

Supplementary Table 3. Evaluation of solvent.

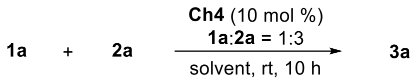

entry	solvent (0.2 M)	yield (%)
1	DCM	75
2	DCE	77
3	$CHCl_3$	63
4	toluene	n.r.

Supplementary Table 4. Evaluation of reaction temperature and the molar ratio of the substrate.

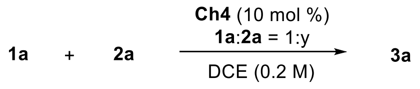

entry	T (°C)	time (h)	1a:2a	yield (%)
1	rt	10	1:3	77
2	50	1	1:3	85
3	50	1	1:1	47
4	50	1	1:5	86

General procedure for optimization: To a reaction mixture of catalyst **Ch** (0.02 mmol, 10 mol %) and **1** (0.2 mmol) in a 10 mL-Schlenk tube was added a specific solvent under argon atmosphere. Then **2a** (y equiv) was added to the above reaction mixture. The reaction was stirred at room temperature or 50 ºC for the indicated reaction time. Then the solvent was removed under reduced pressure and the residue was purified by flash chromatography on silica gel using petroleum ether/ethyl acetate as eluent to give the desired products.

4. Optimized Procedure

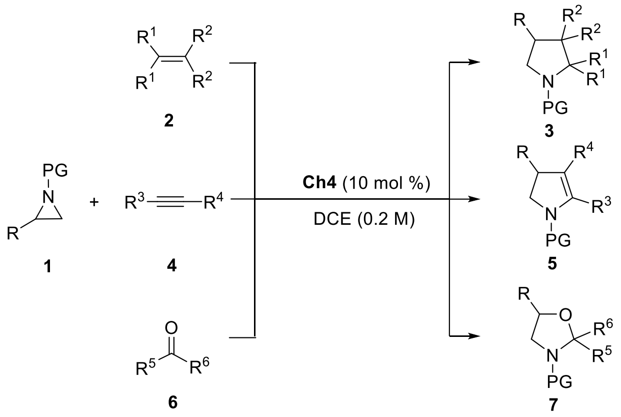

General procedure: To a reaction mixture of catalyst **Ch4** (52.36 mg, 0.02 mmol) and aziridines **1** (0.2 mmol) in a 10 mL-Schlenk tube was added DCE (1.0 mL) under argon atmosphere. Then alkenes **2**, alkynes **4** or ketone **6** (0.6 mmol, 3 equiv) was added to the above reaction mixture. The reaction was stirred at 50 °C (rt for **5a-5i**) until the completion of the reaction as judged by TLC analysis. Then the solvent was removed under reduced pressure and the residue was purified by flash chromatography on silica gel using petroleum ether/ethyl acetate as eluent to give the desired products.

Typical procedure for synthesis of product 3a: To a reaction mixture of catalyst **Ch4** (52.36 mg, 0.02 mmol) and **1a** (60.81 mg, 0.2 mmol) in a 10 mL-Schlenk tube was added DCE (1.0 mL) under argon atmosphere. Then **2a** (50.45 mg, 0.6 mmol) was added to the above reaction mixture. The reaction was stirred at 50 °C for 1 h. Then the solvent was removed under reduced pressure and the residue was purified by flash chromatography on silica gel using petroleum ether/ethyl acetate (v/v = 30:1 to 10:1) as eluent to give the desired product **3a** (85% yield, 65.9 mg).

5. Analytical Data for Products

2,2-diethyl-1-[(4-nitrophenyl)sulfonyl]-4-phenylpyrrolidine (**3a**)

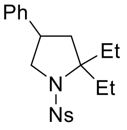

3a

Compound **3a** was synthesized according to the general procedure as a solid (85% yield, 65.9 mg); ^{1}H NMR (400 MHz, 298K, $CDCl_3$) δ 8.35 (d, *J* = 8.9 Hz, 2H), 8.05 (d, *J* = 8.9 Hz, 2H), 7.34–7.28 (m, 2H), 7.27–7.21 (m, 1H), 7.21–7.14 (m, 2H), 3.87–3.91 (m, 1H), 3.23–3.33 (m, 2H), 2.24–2.15 (m, 1H), 2.11–1.98 (m, 2H), 1.97–1.81 (m, 3H), 0.98–0.93 (m, 6H); ^{13}C NMR (100 MHz, 298K, $CDCl_3$) δ 149.59, 146.85, 139.60, 128.72, 128.22, 127.25, 126.98, 124.18, 74.40, 56.09, 42.13, 41.37, 32.58, 32.33; HRMS (ESI+) exact mass calculated for $[M+H]^+$ ($C_{20}H_{25}N_2O_4S$) requires m/z 389.1530, found m/z 389.1526.

2,2-diethyl-4-phenyl-1-tosylpyrrolidine (**3b**)

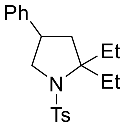

3b

Compound **3b** was synthesized according to the general procedure as a solid (60% yield, 42.8 mg); ^{1}H NMR (400 MHz, 298K, $CDCl_3$) δ 7.76 (d, *J* = 8.3 Hz, 2H), 7.32–7.28 (m, 4H), 7.25–7.21 (m, 1H), 7.19–7.17 (m, 2H), 3.85–3.81 (m, 1H), 3.35–3.20 (m, 2H), 2.42 (s, 3H), 2.19–1.82 (m, 6H), 0.97 (m, 6H); ^{13}C NMR (100 MHz, 298K, $CDCl_3$) δ 142.60, 140.33, 138.46, 129.35, 128.57, 127.10, 127.03, 126.95, 73.44, 55.70, 42.28, 41.31, 32.41, 32.16, 21.44, 9.65, 8.95; HRMS (ESI+) exact mass calculated for $[M+H]^+$ ($C_{21}H_{28}NO_2S$) requires m/z 358.1835, found m/z 358.1833.

2,2-diethyl-4-phenyl-1-(phenylsulfonyl)pyrrolidine (**3c**)

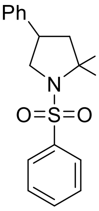

3c

Compound **3c** was synthesized according to the general procedure as a solid (67% yield, 45.9 mg); ^{1}H NMR (400 MHz, 298K, $CDCl_3$) δ 7.91–7.87 (m, 2H), 7.57–7.48 (m, 3H), 7.33–7.29 (m, 2H), 7.25–7.21 (m, 1H), 7.19–7.17 (m, 2H), 3.88–3.84 (m, 1H), 3.36–3.22 (m, 2H), 2.20–1.82 (m, 6H), 0.97 (t, *J* = 7.3 Hz, 3H), 0.95 (t, *J* = 7.4 Hz, 3H); ^{13}C NMR (100 MHz, 298K, $CDCl_3$) δ 141.32, 140.23, 131.99, 128.78, 128.59, 127.02, 126.99, 73.57, 55.78, 42.24, 41.31, 32.44, 32.22, 9.64, 8.92; HRMS (ESI+) exact mass calculated for $[M+H]^+$ ($C_{20}H_{26}NO_2S$) requires m/z 344.1679, found m/z 344.1676.

2,2-dimethyl-1-[(4-nitrophenyl)sulfonyl]-4-phenylpyrrolidine (**3d**)

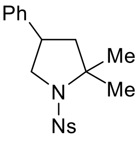

3d

Compound **3d** was synthesized according to the general procedure as a solid (71% yield, 51.1 mg); ^{1}H NMR (400 MHz, 298K, $CDCl_3$) δ 8.34 (d, *J* = 8.9 Hz, 2H), 8.04 (d, *J* = 8.9 Hz, 2H), 7.34–7.28 (m, 2H), 7.27–7.22 (m, 1H), 7.19–7.14 (m, 2H), 4.03–3.95 (m, 1H), 3.55–3.44 (m, 1H), 3.22 (dd,

J = 10.8, 9.2 Hz, 1H), 2.19 (ddd, J = 12.3, 6.2, 0.8 Hz, 1H), 2.00 (t, J = 12.4 Hz, 1H), 1.59 (s, 3H), 1.55 (s, 3H); ^{13}C NMR (100 MHz, 298K, $CDCl_3$) δ 149.64, 146.85, 139.16, 128.71, 128.21, 127.25, 126.95, 124.21, 66.53, 55.56, 49.53, 40.67, 29.15, 28.31; HRMS (ESI+) exact mass calculated for $[M+H]^+$ ($C_{18}H_{21}N_2O_4S$) requires m/z 361.1217, found m/z 361.1212.

1-[(4-nitrophenyl)sulfonyl]-2,2-dipentyl-4-phenylpyrrolidine (3e)

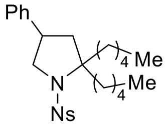

3e

Compound **3e** was synthesized according to the general procedure as a solid (81% yield, 76.5 mg); ^{1}H NMR (400 MHz, 298K, $CDCl_3$) δ 8.35 (d, J = 8.8 Hz, 2H), 8.03 (d, J = 8.8 Hz, 2H), 7.33–7.30 (m, 2H), 7.26–7.23 (m, 1H), 7.18–7.16 (m, 2H), 3.89 (t, J = 7.5 Hz, 1H), 3.37–3.22 (m, 2H), 2.21 (dd, J = 12.8, 6.6 Hz, 1H), 2.10–2.04(m, 1H), 1.98–1.91 (m, 1H), 1.89–1.76 (m, 3H), 1.41–1.26 (m, 12H), 0.91–0.86 (m, 6H); ^{13}C NMR (100 MHz, 298K, $CDCl_3$) δ 149.56, 146.99, 139.59, 128.71, 128.16, 127.23, 126.99, 124.13, 73.58, 55.91, 43.28, 41.36, 40.67, 40.21, 32.22, 32.17, 24.90, 24.21, 22.67, 22.61, 14.09, 14.05; HRMS (ESI+) exact mass calculated for $[M+H]^+$ ($C_{26}H_{37}N_2O_4S$) requires m/z 473.2469, found m/z 473.2464.

2,2,3,3-tetramethyl-1-[(4-nitrophenyl)sulfonyl]-4-phenylpyrrolidine (3f)

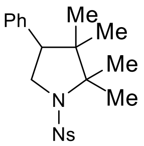

3f

Compound **3f** was synthesized according to the general procedure as a solid (82% yield, 63.6 mg); ^{1}H NMR (400 MHz, 298K, $CDCl_3$) δ 8.37 (d, J = 8.9 Hz, 2H), 8.10 (d, J = 8.9 Hz, 2H), 7.35–7.26 (m, 3H), 7.16–7.10 (m, 2H), 3.88–3.82 (m, 1H), 3.66 (dd, J = 11.6, 9.6 Hz, 1H), 3.32 (dd, J = 11.6, 8.4 Hz, 1H), 1.44 (s, 3H), 1.38 (s, 3H), 0.79 (s, 3H), 0.51 (s, 3H); ^{13}C NMR (100 MHz, 298K, $CDCl_3$) δ 149.63, 147.26, 135.84, 129.08, 128.15, 128.08, 127.40, 124.26, 71.89, 50.12, 49.78, 47.70, 25.96, 22.55, 20.93, 18.49; HRMS (ESI+) exact mass calculated for $[M+H]^+$ ($C_{20}H_{25}N_2O_4S$) requires m/z 389.1530, found m/z 389.1530.

1-[(4-nitrophenyl)sulfonyl]-3-phenyl-1-azaspiro[4.4]nonane (3g)

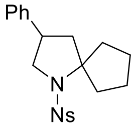

3g

Compound **3g** was synthesized according to the general procedure as a solid (80% yield, 61.7 mg); ^{1}H NMR (400 MHz, 298K, $CDCl_3$) δ 8.38–8.31 (m, 2H), 8.07–8.01 (m, 2H), 7.34–7.28 (m, 2H), 7.27–7.21 (m, 1H), 7.21–7.14 (m, 2H), 4.03–3.99 (m, 1H), 3.50–3.38 (m, 1H), 3.21 (m, 1H), 2.65–2.54 (m, 1H), 2.38–2.23 (m, 2H), 1.93–1.78 (m, 4H), 1.61–1.44 (m, 3H); ^{13}C NMR (100 MHz, 298K, $CDCl_3$) δ 149.64, 146.92, 139.08, 128.69, 128.09, 126.98, 124.26, 75.44, 55.53, 48.40, 41.06, 38.63, 36.87, 23.46, 22.83; HRMS (ESI+) exact mass calculated for $[M+H]^+$ ($C_{20}H_{23}N_2O_4S$) requires m/z 387.1373, found m/z 387.1373.

1-[(4-nitrophenyl)sulfonyl)-3-phenyl-1-azaspiro[4.5]decane (3h)

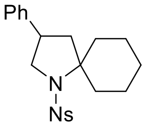

3h

Compound **3h** was synthesized according to the general procedure as a solid (76% yield, 60.8 mg); ^{1}H NMR (400 MHz, 298K, $CDCl_3$) δ 8.37–8.30 (m, 2H), 8.08–8.02 (m, 2H), 7.34–7.28 (m, 2H), 7.27–7.22 (m, 1H), 7.21–7.17 (m, 2H), 4.01–3.97 (m, 1H), 3.43–3.34 (m, 1H), 3.21 (dd, *J* = 11.0, 9.1 Hz, 1H), 2.59 (dd, *J* = 12.5, 6.1 Hz, 1H), 2.48 (td, *J* = 12.7, 3.8 Hz, 1H), 2.34–2.27 (m, 1H), 1.83–1.61 (m, 5H), 1.49 (d, *J* = 12.3 Hz, 1H), 1.38–1.28 (m, 2H), 1.23–1.21 (m, 1H); ^{13}C NMR (100 MHz, 298K, $CDCl_3$) δ 149.55, 147.28, 139.23, 128.68, 128.18, 127.24, 126.98, 124.17, 71.18, 55.21, 43.52, 40.90, 37.88, 36.00, 24.86, 24.81, 24.19; HRMS (ESI+) exact mass calculated for $[M+H]^+$ ($C_{21}H_{25}N_2O_4S$) requires m/z 401.1530, found m/z 401.1528.

1-[(4-nitrophenyl)sulfonyl]-3-phenyl-1-azaspiro[4.6]undecane (3i)

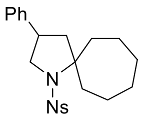

3i

Compound **3i** was synthesized according to the general procedure as a solid (86% yield, 71.2 mg); ^{1}H NMR (400 MHz, 298K, $CDCl_3$) δ 8.34 (d, *J* = 8.8 Hz, 2H), 8.04 (d, *J* = 8.8 Hz, 2H), 7.33–7.30 (m, 2H), 7.27–7.23 (m, 1H), 7.20–7.18 (m, 2H), 3.96 (t, *J* = 8.1 Hz, 1H), 3.47–3.38 (m, 1H), 3.17 (dd, *J* = 10.9, 9.1 Hz, 1H), 2.56–2.40 (m, 3H), 1.99 (dd, *J* = 13.9, 6.3 Hz, 1H), 1.87–1.56 (m, 7H), 1.53–1.45 (m, 1H), 1.442– 1.24 (m, 2H); ^{13}C NMR (100 MHz, 298K, $CDCl_3$) δ 147.15, 139.21, 128.69, 128.16, 127.25, 127.01, 124.19, 74.42, 55.22, 45.72, 41.08, 40.63, 39.50, 27.65, 27.49, 23.66, 22.58; HRMS (ESI+) exact mass calculated for $[M+H]^+$ ($C_{22}H_{26}N_2O_4S$) requires m/z 415.1686, found m/z 415.1691.

7,7,9,9-tetramethyl-1-[(4-nitrophenyl)sulfonyl]-3-phenyl-1-azaspiro[4.5]decane (3j)

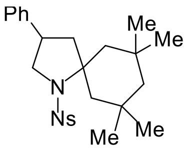

3j

Compound **3j** was synthesized according to the general procedure as a solid (85% yield, 77.5 mg); ^{1}H NMR (400 MHz, 298K, $CDCl_3$) δ 8.35 (d, *J* = 8.9 Hz, 2H), 8.03 (d, *J* = 8.9 Hz, 2H), 7.34–7.31 (m, 2H), 7.28–7.24 (m, 1H), 7.20–7.18 (m, 2H), 3.94 (t, *J* = 8.1 Hz, 1H), 3.47–3.38 (m, 1H), 3.07 (dd, *J* = 11.0, 9.1 Hz, 1H), 2.76 (dd, *J* = 12.2, 5.9 Hz, 1H), 2.56 (d, *J* = 12.8 Hz, 1H), 2.26 (d, *J* = 13.7 Hz, 1H), 1.87 (t, *J* = 12.1 Hz, 1H), 1.75 (d, *J* = 13.7 Hz, 1H), 1.35–1.25 (m, 3H), 1.11 (s, 3H), 1.02 (s, 6H), 0.98 (s, 3H); ^{13}C NMR (100 MHz, 298K, $CDCl_3$) δ 149.53`, 147.34, 139.10, 128.73, 128.17, 127.33, 127.06, 124.18, 77.32, 77.00, 76.68, 73.03, 54.02, 50.18, 48.55, 47.49, 47.16, 41.52, 36.96, 36.20, 32.85, 32.76, 29.12, 27.83; HRMS (ESI+) exact mass calculated for $[M+H]^+$ ($C_{25}H_{33}N_2O_4S$) requires m/z 457.2156, found m/z 457.2153.

1-[(4-nitrophenyl)sulfonyl]-3-phenyl-1-azaspiro[4.14]nonadecane (3k)

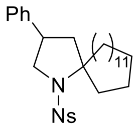

3k

Compound **3k** was synthesized according to the general procedure as a solid (84% yield, 88.4 mg); ^{1}H NMR (400 MHz, 298K, $CDCl_3$) δ 8.34 (d, *J* = 8.9 Hz, 2H), 8.04 (d, *J* = 8.9 Hz, 2H), 7.32–7.28 (m, 2H), 7.26–7.22 (m, 1H), 7.16–7.14 (m, 2H), 3.92 (t, *J* = 8.3 Hz, 1H), 3.44–3.35 (m, 1H), 3.19 (dd, *J* = 10.8, 9.4 Hz, 1H), 2.32b–2.26 (m, 2H), 2.07–2.03 (m, 1H), 1.92 (t, *J* = 12.6 Hz, 1H), 1.87–1.81 (m, 1H), 1.68–1.61 (m, 1H), 1.50–1.28 (m, 24H); ^{13}C NMR (100 MHz, 298K, $CDCl_3$) δ 149.57, 147.03, 139.56, 128.70, 128.32, 127.23, 126.94, 124.15, 73.91, 55.59, 44.22, 40.89, 39.29, 39.10, 27.70, 27.68, 26.85, 26.71, 26.62, 26.55, 26.48, 26.19, 26.10, 23.49, 23.44; HRMS (ESI+) exact mass calculated for $[M+H]^+$ ($C_{30}H_{43}N_2O_4S$) requires m/z 527.2938, found m/z 527.2935.

2,2-diethyl-1-[(4-nitrophenyl)sulfonyl]-4-(p-tolyl)pyrrolidine (3l)

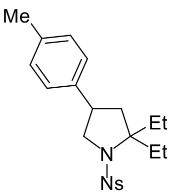

3l

Compound **3l** was synthesized according to the general procedure as a solid (83% yield, 66.7 mg); ^{1}H NMR (400 MHz, 298K, $CDCl_3$) δ 8.34 (d, *J* = 8.8 Hz, 2H), 8.04 (d, *J* = 8.8 Hz, 2H), 7.12 (d, *J* = 8.0 Hz, 2H), 7.06 (d, *J* = 8.1 Hz, 2H), 3.86 (t, *J* = 7.5 Hz, 1H), 3.34–3.19 (m, 2H), 2.32 (s, 3H), 2.17 (dd, *J* = 13.6, 6.8 Hz, 1H), 2.03 (dd, *J* = 16.3, 9.7 Hz, 2H), 1.94–1.81 (m, 3H), 0.98–0.92 (m, 6H); ^{13}C NMR (100 MHz, 298K, $CDCl_3$) δ 149.56, 146.87, 136.91, 136.51, 129.36, 128.20, 126.83, 124.15, 74.38, 56.17, 42.18, 41.00, 32.56, 32.32, 20.95, 9.67, 8.93; HRMS (ESI+) exact mass calculated for $[M+H]^+$ ($C_{21}H_{27}N_2O_4S$) requires m/z 403.1686, found m/z 403.1689.

4-[(1,1'-biphenyl)-4-yl]-2,2-diethyl-1-[(4-nitrophenyl)sulfonyl]pyrrolidine (3m)

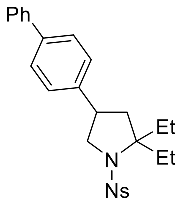

3m

Compound **3m** was synthesized according to the general procedure as a solid (89% yield, 82.8 mg); ^{1}H NMR (400 MHz, 298K, $CDCl_3$) δ 8.35 (d, *J* = 8.9 Hz, 2H), 8.05 (d, *J* = 8.9 Hz, 2H), 7.58–7.51 (m, 4H), 7.43 (m, 2H), 7.34 (m, 1H), 7.26–7.24 (m, 2H), 3.92 (m, 1H), 3.34 (m, 2H), 2.25–2.20 (m, 1H), 2.13–2.00 (m, 2H), 1.98–1.84 (m, 3H), 1.00–0.94 (m, 6H).^{13}C NMR (100 MHz, 298K, $CDCl_3$) δ 149.58, 146.83, 140.43, 140.22, 138.60, 128.78, 128.22, 127.42, 127.38, 124.18, 74.42, 56.07, 42.15, 41.08, 32.58, 32.33, 9.69, 8.96.HRMS (ESI+) exact mass calculated for $[M+H]^+$ ($C_{26}H_{29}N_2O_4S$) requires m/z 465.1843, found m/z 465.1843.

4-[4-(tert-butyl)phenyl]-2,2-diethyl-1-[(4-nitrophenyl)sulfonyl]pyrrolidine (3n)

Compound **3n** was synthesized according to the general procedure as a solid (90% yield, 79.9 mg); ^{1}H NMR (400 MHz, 298K, $CDCl_3$) δ 8.35 (d, *J* = 8.9 Hz, 2H), 8.04 (d, *J* = 8.9 Hz, 2H), 7.34 (d, *J* = 8.4 Hz, 2H), 7.12 (d, *J* = 8.3

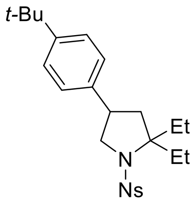

3n

Hz, 2H), 3.90–3.88 (m, 1H), 3.3–3.22 (m, 2H), 2.20–2.15 (m, 1H), 2.09–1.99 m, 2H), 1.95–1.82 (m, 3H), 1.30 (s, 9H), 0.97 (t, J = 7.5 Hz, 3H), 0.94 (t, J = 7.4 Hz, 3H); ^{13}C NMR (100 MHz, 298K, $CDCl_3$) δ 150.23, 149.55, 146.87, 136.51, 128.20, 126.66, 125.58, 124.14, 74.39, 56.18, 42.13, 40.92, 34.41, 32.56, 32.32, 31.24, 9.67, 8.93; HRMS (ESI+) exact mass calculated for $[M+H]^+$ ($C_{24}H_{33}N_2O_4S$) requires m/z 445.2156, found m/z 445.2158.

4-[5,5-diethyl-1-[(4-nitrophenyl)sulfonyl]pyrrolidin-3-yl]phenyl acetate (**3o**)

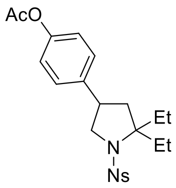

3o

Compound **3o** was synthesized according to the general procedure as a solid (82% yield, 73.1 mg); ^{1}H NMR (400 MHz, 298K, $CDCl_3$) δ 8.34 (d, J = 8.9 Hz, 2H), 8.03 (d, J = 8.9 Hz, 2H), 7.17 (d, J = 8.5 Hz, 2H), 7.02 (d, J = 8.6 Hz, 2H), 3.89–3.85 (m, 1H), 3.32–3.29 (m, 1H), 3.24–3.19 (m, 1H), 2.28 (s, 3H), 2.20–2.15 (m, 1H), 2.07–1.98 (m, 2H), 1.94–1.81 (m, 3H), 0.95 (t, J = 7.5 Hz, 3H), 0.93 (t, J = 7.4 Hz, 3H); ^{13}C NMR (100 MHz, 298K, $CDCl_3$) δ 169.47, 149.63, 149.59, 146.75, 137.13, 128.20, 127.98, 124.18, 121.83, 74.36, 56.05, 42.16, 40.83, 32.52, 32.30, 21.03, 9.64, 8.92; HRMS (ESI+) exact mass calculated for $[M+H]^+$ ($C_{22}H_{26}N_2O_4S$) requires m/z 447.1584, found m/z 447.1579.

4-(4-chlorophenyl)-2,2-diethyl-1-[(4-nitrophenyl)sulfonyl]pyrrolidine (**3p**)

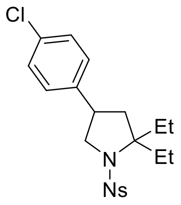

3p

Compound **3p** was synthesized according to the general procedure as a solid (72% yield, 60.7 mg); ^{1}H NMR (400 MHz, 298K, $CDCl_3$) δ 8.34 (d, J = 8.9 Hz, 2H), 8.04 (d, J = 8.9 Hz, 2H), 7.28–7.26 (m, 2H), 7.10 (d, J = 8.4 Hz, 2H), 3.88–3.84 (m, 1H), 3.34–3.19 (m, 2H), 2.21–2.16 (m, 1H), 2.07–1.98 (m, 2H), 1.96–1.82 (m, 3H), 0.95 (t, J = 7.4 Hz, 3H), 0.94 (t, J = 7.3 Hz, 3H); ^{13}C NMR (100 MHz, 298K, $CDCl_3$) δ 149.61, 146.73, 138.06, 132.95, 128.82, 128.32, 128.21, 124.20, 74.36, 55.89, 42.07, 40.77, 32.54, 32.28, 9.65, 8.91; HRMS (ESI+) exact mass calculated for $[M+H]^+$ ($C_{20}H_{24}ClN_2O_4S$) requires m/z 423.1140, found m/z 423.1135.

4-(4-bromophenyl)-2,2-diethyl-1-[(4-nitrophenyl)sulfonyl]pyrrolidine (**3q**)

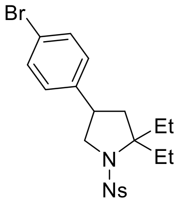

3q

Compound **3q** was synthesized according to the general procedure as a solid (71% yield, 66.1 mg); ^{1}H NMR (400 MHz, 298K, $CDCl_3$) δ 8.34 (d, J = 8.9 Hz, 2H), 8.04 (d, J = 8.9 Hz, 2H), 7.41 (d, J = 8.5 Hz, 2H), 7.04 (d, J = 8.4 Hz, 2H), 3.88–3.84 (m , 1H), 3.32–3.18 (m, 2H), 2.20–2.15 (m, 1H), 2.06–1.97 (m, 2H), 1.95–1.81 (m, 3H), 0.94 (t, J = 7.4 Hz, 3H) , 0.93 (t, J = 7.3 Hz, 3H); ^{13}C NMR (100 MHz, 298K, $CDCl_3$) δ 149.59, 146.71, 138.59, 131.76, 128.69, 128.20, 124.20, 120.96, 74.34, 55.80, 42.01, 40.82, 32.53, 32.27, 9.64, 8.91; HRMS (ESI+) exact mass calculated

for [M+H]+ ($C_{20}H_{24}BrN_2O_4S$) requires m/z 467.0635, found m/z 467.0636.

2,2-diethyl-1-[(4-nitrophenyl)sulfonyl]-4-(m-tolyl)pyrrolidine (3r)

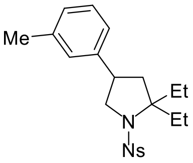

3r

Compound **3r** was synthesized according to the general procedure as a solid (80% yield, 64.5 mg); ^{1}H NMR (400 MHz, 298K, $CDCl_3$) δ 8.35 (d, J = 8.9 Hz, 2H), 8.05 (d, J = 8.9 Hz, 2H), 7.23–7.17 (m, 1H), 7.06 (d, J = 7.4 Hz, 1H), 6.98 (d, J = 6.8 Hz, 2H), 3.91-3.84 (m, 1H), 3.35–3.23 (m, 2H), 2.33 (s, 3H), 2.22–2.14 (m, 1H), 2.10–1.98 (m, 2H), 1.97–1.81 (m, 3H), 0.97 (t, J = 7.4 Hz, 3H) , 0.96 (t, J = 7.4 Hz, 3H); ^{13}C NMR (100 MHz, 298K, $CDCl_3$) δ 149.53, 146.85, 139.50, 138.33, 128.56, 128.18, 127.91, 127.74, 124.13, 123.90, 74.33, 56.02, 42.03, 41.25, 32.55, 32.27, 21.33, 9.64, 8.91; HRMS (ESI+) exact mass calculated for [M+H]+ ($C_{21}H_{27}N_2O_4S$) requires m/z 403.1686, found m/z 403.1687.

4-(3-chlorophenyl)-2,2-diethyl-1-[(4-nitrophenyl)sulfonyl]pyrrolidine (3s)

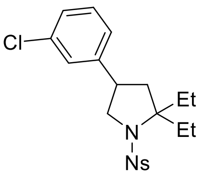

3s

Compound **3s** was synthesized according to the general procedure as a solid (59% yield, 49.8 mg); ^{1}H NMR (400 MHz, 298K, $CDCl_3$) δ 8.35 (d, J = 8.9 Hz, 2H), 8.04 (d, J = 8.9 Hz, 2H), 7.26–7.19 (m, 2H), 7.14 (m, 1H), 7.05 (dt, J = 6.8, 1.7 Hz, 1H), 3.89–3.85 (m, 1H), 3.34–3.20 (m, 2H), 2.21–2.16 (m, 1H), 2.07–1.98 (m, 2H), 1.95–1.82 (m, 3H), 0.95 (t, J = 7.4 Hz, 3H), 0.94 (t, J = 7.3 Hz, 3H); ^{13}C NMR (100 MHz, 298K, $CDCl_3$) δ 149.61, 146.70, 141.68, 134.51, 129.96, 128.21, 127.38, 127.16, 125.20, 124.22, 74.33, 55.68, 41.88, 40.97, 32.54, 32.25, 9.62, 8.92; HRMS (ESI+) exact mass calculated for [M+H]+ ($C_{20}H_{24}ClN_2O_4S$) requires m/z 423.1140, found m/z 423.1142.

2,2-diethyl-1-[(4-nitrophenyl)sulfonyl]-4-(o-tolyl)pyrrolidine (3t)

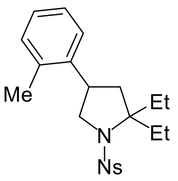

3t

Compound **3t** was synthesized according to the general procedure as a solid (73% yield, 58.7 mg); ^{1}H NMR (400 MHz, 298K, $CDCl_3$) δ 8.35 (d, J = 8.9 Hz, 2H), 8.05 (d, J = 8.9 Hz, 2H), 7.19–7.11 (m, 4H), 3.87–3.81 (m, 1H), 3.56-3.47 (m, 1H), 3.30 (dd, J = 11.2, 9.3 Hz, 1H), 2.32 (s, 3H), 2.17–1.98 (m, 3H), 1.98–1.81 (m, 3H), 0.99 (t, J = 7.4 Hz, 3H), 0.94 (t, J = 7.4 Hz, 3H); ^{13}C NMR (100 MHz, 298K, $CDCl_3$) δ 149.58, 146.86, 137.53, 136.24, 130.68, 128.18, 126.92, 126.45, 124.86, 124.17, 74.13, 55.11, 41.93, 37.42, 32.69, 32.28, 19.66, 9.63, 8.99; HRMS (ESI+) exact mass calculated for [M+H]+ ($C_{21}H_{27}N_2O_4S$) requires m/z 403.1686, found m/z 403.1683.

4-(2-chlorophenyl)-2, 2-diethyl-1-[(4-nitrophenyl)sulfonyl]pyrrolidine (3u)

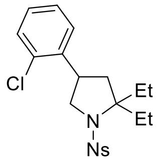

3u

Compound **3u** was synthesized according to the general procedure as a solid (58% yield, 48.9 mg); ^{1}H NMR (400 MHz, 298K, $CDCl_3$) δ 8.36 (d, *J* = 8.9 Hz, 2H), 8.06 (d, *J* = 8.9 Hz, 2H), 7.36 (d, *J* = 7.5 Hz, 1H), 7.24–7.16 (m, 3H), 3.91–3.87 (m, 1H), 3.77–3.68 (m, 1H), 3.33 (dd, *J* = 11.1, 9.4 Hz, 1H), 2.23 (dd, *J* = 12.8, 6.9 Hz, 1H), 2.07–1.80 (m, 5H), 0.96 (t, *J* = 7.3 Hz, 3H), 0.95 (t, *J* = 7.4 Hz, 3H); ^{13}C NMR (100 MHz, 298K, $CDCl_3$) δ 149.60, 146.91, 136.88, 134.33, 129.92, 128.31, 128.18, 127.23, 126.62, 124.20, 74.11, 54.24, 40.82, 37.95, 32.70, 32.16, 9.57, 8.91; HRMS (ESI+) exact mass calculated for $[M+H]^+$ ($C_{20}H_{24}ClN_2O_4S$) requires m/z 423.1140, found m/z 423.1144.

2-cyclohexyl-1-[(4-nitrophenyl)sulfonyl]-4-phenylpyrrolidine (3v)

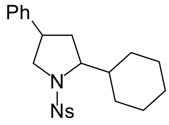

3v

Compound **3v** was synthesized according to the general procedure as a solid (mixture 3:1) (53% yield, 43.9 mg); ^{1}H NMR (400 MHz, 298K, $CDCl_3$) δ 8.45 – 8.28 (m, 2H), 8.11 – 7.95 (m, 2H), 7.38 – 6.92 (m, 5H), 4.33 – 2.82 (m, 3H), 2.40 – 2.08 (m, 2H), 1.94 – 1.51 (m, 7H), 1.47 – 0.94 (m, 6H); ^{13}C NMR (100 MHz, 298K, CDCl3) δ 150.17, 149.99, 149.50, 143.23, 142.37, 128.66, 128.38, 127.92, 127.24, 126.62, 124.30, 65.94, 55.61, 55.37, 48.42, 43.51, 43.10, 42.53, 42.17, 36.69, 33.93, 31.59, 30.59, 30.30, 27.80, 26.50, 26.41, 26.26, 26.22, 26.06 , 25.25, 22.56; HRMS (ESI+) exact mass calculated for $[M+H]^+$ ($C_{22}H_{27}N_2O_4S$) requires m/z 415.5275, found m/z 415.5273.

4-methyl-1-[(4-nitrophenyl)sulfonyl]-3,5-diphenyl-2,3-dihydro-1H-pyrrole (5a)

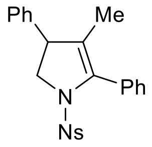

5a

Compound **5a** was synthesized according to the general procedure as a solid (69% yield, 57.9 mg); ^{1}H NMR (400 MHz, 298K, $CDCl_3$) δ 8.22–8.19 (m, 2H), 7.69–7.66 (m, 2H), 7.43–7.41 (m, 5H), 7.19–7.14 (m, 3H), 6.83–6.80 (m, 2H), 4.43 (dd, *J* = 12.3, 10.3 Hz, 1H), 3.86 (dd, *J* = 12.3, 6.1 Hz, 1H), 3.72 (dd, *J* = 9.8, 6.2 Hz, 1H), 1.48 (d, *J* = 1.0 Hz, 3H); ^{13}C NMR (100 MHz, 298K, $CDCl_3$) δ 150.12, 142.85, 141.54, 137.31, 131.56, 129.88, 129.12, 128.74, 127.90, 127.25, 127.0, 126.47, 123.89, 57.75, 51.16, 12.83; HRMS (ESI+) exact mass calculated for $[M+H]^+$ ($C_{23}H_{21}N_2O_4S$) requires m/z 421.1217, found m/z 421.1219.

4-ethyl-1-[(4-nitrophenyl)sulfonyl]-3,5-diphenyl-2,3-dihydro-1H-pyrrole (5b)

Compound **5b** was synthesized according to the general procedure as a solid (79% yield, 68.6 mg); ^{1}H NMR (400 MHz, 298K, $CDCl_3$) δ 8.25–8.22 (m, 2H), 7.73–7.70 (m, 2H), 7.41 (s, 5H), 7.20–7.15 (m, 3H), 6.89-6.85 (m, 2H),

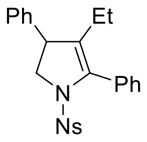

5b

4.46–4.38 (m, 1H), 3.90–3.82 (m, 2H), 2.15–2.05 (m, 1H), 1.71–1.62 (m, 1H), 0.71 (t, *J* = 7.5 Hz, 3H); ^{13}C NMR (100 MHz, 298K, CDCl$_3$) δ 150.07, 143.06, 141.39, 136.78, 132.34, 131.59, 129.74, 129.10, 128.73, 128.72, 127.92, 127.42, 127.13, 123.83, 57.91, 48.29, 19.33, 12.36; HRMS (ESI+) exact mass calculated for [M+H]$^+$ ($C_{24}H_{23}N_2O_4S$) requires m/z 435.1373, found m/z 435.1372.

1-[(4-nitrophenyl)sulfonyl]-3,4,5-triphenyl-2,3-dihydro-1H-pyrrole (5c)

Compound **5c** was synthesized according to the general procedure as a solid (67% yield, 64.6 mg); ^{1}H NMR (400 MHz, 298K, CDCl$_3$) δ 8.14 (d, *J* = 8.8 Hz, 2H), 7.63 (d, *J* = 8.8 Hz, 2H), 7.42–7.32 (m, 5H), 7.14–7.08 (m, 3H), 7.00–6.91 (m, 5H), 6.70–6.67 (m, 2H), 4.58 (dd, *J* = 11.8, 9.4 Hz, 1H), 4.18-4.07 (m, 2H); ^{13}C NMR (100 MHz, 298K, CDCl$_3$) δ 150.06, 144.07, 142.16, 138.85, 133.85, 131.36, 130.72, 129.36, 128.86, 128.71, 128.24, 127.96, 127.88, 127.83, 127.40, 126.99, 126.82, 123.91, 58.83, 50.10; HRMS (ESI+) exact mass calculated for [M+H]$^+$ ($C_{28}H_{23}N_2O_4S$) requires m/z 483.1373, found m/z 483.1369.

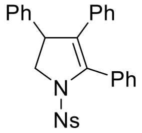

5c

4-methyl-1-[(4-nitrophenyl)sulfonyl]-5-phenyl-3-(p-tolyl)-2,3-dihydro-1H-pyrrole (5d)

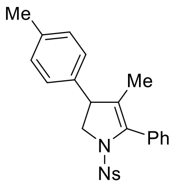

5d

Compound **5d** was synthesized according to the general procedure as a solid (61% yield, 52.9 mg); ^{1}H NMR (400 MHz, 298K, CDCl$_3$) δ 8.20 (d, *J* = 8.5 Hz, 2H), 7.67 (d, *J* = 8.5 Hz, 2H), 7.45–7.39 (m, 5H), 6.95 (d, *J* = 7.8 Hz, 2H), 6.68 (d, *J* = 7.8 Hz, 2H), 4.41 (dd, *J* = 11.9, 10.6 Hz, 1H), 3.83 (dd, *J* = 12.3, 6.1 Hz, 1H), 3.67 (dd, *J* = 9.9, 6.2 Hz, 1H), 2.29 (s, 3H), 1.49 (s, 3H); ^{13}C NMR (100 MHz, 298K, CDCl$_3$) δ 150.06, 142.89, 138.54, 137.17, 136.85, 131.67, 129.86, 129.40, 129.10, 128.69, 127.89, 127.10, 126.72, 123.83, 77.32, 77.00, 76.68, 57.91, 50.79, 20.91, 12.88; HRMS (ESI+) exact mass calculated for [M+H]$^+$ ($C_{24}H_{23}N_2O_4S$) requires m/z 435.1373, found m/z 435.1374.

3-[4-(tert-butyl)phenyl]-4-methyl-1-[(4-nitrophenyl)sulfonyl]-5-phenyl-2,3-dihydro-1H-pyrrole (5e)

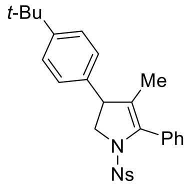

5e

Compound **5e** was synthesized according to the general procedure as a solid (63% yield, 60.0 mg); ^{1}H NMR (400 MHz, 298K, CDCl$_3$) δ 8.22 (d, *J* = 8.8 Hz, 2H), 7.69 (d, *J* = 8.7 Hz, 2H), 7.44–7.40 (m, 5H), 7.16 (d, *J* = 8.2 Hz, 2H), 6.70 (d, *J* = 8.2 Hz, 2H), 4.41 (dd, *J* = 12.2, 10.4 Hz, 1H), 3.83 (dd, *J* = 12.3, 6.1 Hz, 1H), 3.70 (dd, *J* = 10.0, 6.1 Hz, 1H), 1.49 (s, 3H), 1.28 (s, 9H); ^{13}C NMR (100 MHz, 298K, CDCl$_3$) δ 150.10, 150.07, 138.29, 137.05, 131.64, 129.87, 129.19, 128.66, 127.86, 126.83, 126.70, 125.56, 123.86, 57.67, 50.70, 34.35, 31.20, 12.88; HRMS (ESI+) exact mass calculated for [M+H]$^+$ ($C_{27}H_{29}N_2O_4S$) requires m/z 477.1844, found m/z 477.1838.

3-[(1,1'-biphenyl)-4-yl]-4-methyl-1-[(4-nitrophenyl)sulfonyl]-5-phenyl-2,3-dihydro-1H-pyrrole (5f)

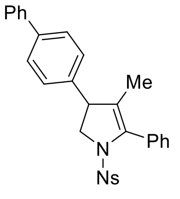

5f

Compound **5f** was synthesized according to the general procedure as a solid (64% yield, 63.6 mg); ^{1}H NMR (400 MHz, 298K, $CDCl_3$) δ 8.25 (dt, J = 2.2, 0.5 Hz, 2H), 7.72 (dt, J = 2.2, 0.5 Hz, 2H), 7.54 (dt, J = 3.0, 1.9 Hz, 2H), 7.47–7.40 (m, 9H), 7.38–7.34 (m, 1H), 6.92 (d, J = 8.2 Hz, 2H), 4.45 (dd, J = 12.2, 10.2 Hz, 1H), 3.90 (dd, J = 12.3, 6.5 Hz, 1H), 3.77 (dd, J = 9.5, 6.9 Hz, 1H), 1.51 (d, J = 1.1 Hz, 3H); ^{13}C NMR (100 MHz, 298K, $CDCl_3$) δ 150.09, 142.97, 140.40, 140.19, 140.14, 137.28, 131.50, 129.89, 129.20, 128.82, 128.72, 127.89, 127.77, 127.44, 127.39, 126.85, 126.52, 123.89, 57.65, 50.93, 12.75; HRMS (ESI+) exact mass calculated for $[M+H]^+$ ($C_{29}H_{25}N_2O_5S$) requires m/z 497.1530, found m/z 497.1525.

4-[4-methyl-1-[(4-nitrophenyl)sulfonyl]-5-phenyl-2,3-dihydro-1H-pyrrol-3-yl]phenyl acetate (5g)

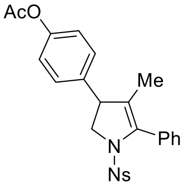

5g

Compound **5g** was synthesized according to the general procedure as a solid (62% yield, 59.3 mg); ^{1}H NMR (400 MHz, 298K, $CDCl_3$) δ 8.20 (d, J = 8.9 Hz, 2H), 7.66 (d, J = 8.8 Hz, 2H), 7.45–7.37 (m, 5H), 6.87 (d, J = 8.6 Hz, 2H), 6.82 (d, J = 8.6 Hz, 2H), 4.41 (dd, J = 12.3, 10.2 Hz, 1H), 3.85 (dd, J = 12.3, 5.9 Hz, 1H), 3.72 (dd, J = 9.8, 6.0 Hz, 1H), 2.30 (s, 3H), 1.49 (d, J = 0.8 Hz, 3H); ^{13}C NMR (100 MHz, 298K, $CDCl_3$) δ 169.32, 150.14, 149.54, 142.87, 139.01, 137.49, 131.39, 129.84, 129.05, 128.74, 128.11, 127.87, 126.08, 123.9, 121.86, 57.69, 50.50, 21.01, 12.80; HRMS (ESI+) exact mass calculated for $[M+H]^+$ ($C_{25}H_{23}N_2O_6S$) requires m/z 479.1271, found m/z 479.1272.

3-(4-chlorophenyl)-4-methyl-1-[(4-nitrophenyl)sulfonyl)-5-phenyl-2,3-dihydro-1H-pyrrole (5h)

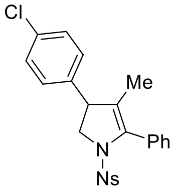

5h

Compound **5h** was synthesized according to the general procedure as a solid (64% yield, 58.1 mg); ^{1}H NMR (400 MHz, 298K, $CDCl_3$) δ 8.24 (d, J = 8.6 Hz, 2H), 7.68 (d, J = 8.6 Hz, 2H), 7.43–7.36 (m, 5H), 7.17 (d, J = 8.3 Hz, 2H), 6.84 (d, J = 8.4 Hz, 2H), 4.40 (dd, J = 12.1, 10.2 Hz, 1H), 3.84 (dd, J = 12.2, 6.4 Hz, 1H), 3.71 (dd, J = 9.7, 6.6 Hz, 1H), 1.45 (s, 3H); ^{13}C NMR (100 MHz, 298K, $CDCl_3$) δ 150.12, 143.05, 139.94, 137.59, 133.12, 131.26, 129.89, 129.16, 128.98, 128.84, 128.71, 127.94, 126.00, 123.88, 57.50, 50.65, 12.60; HRMS (ESI+) exact mass calculated for $[M+H]^+$ ($C_{23}H_{20}ClN_2O_4S$) requires m/z 455.0827, found m/z 455.0828.

4-methyl-1-[(4-nitrophenyl)sulfonyl]-5-phenyl-3-(m-tolyl)-2,3-dihydro-1H-pyrrole (5i)

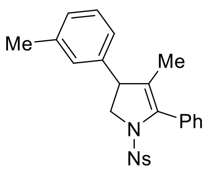

5i

Compound **5i** was synthesized according to the general procedure as a solid (60% yield, 52.1 mg); ^{1}H NMR (400 MHz, 298K, $CDCl_3$) δ 8.23 (d, *J* = 8.8 Hz, 2H), 7.70 (d, *J* = 8.8 Hz, 2H), 7.44–7.40 (m, 5H), 7.06– 6.99 (m, 2H), 6.65 (s, 1H), 6.58 (d, *J* = 7.2 Hz, 1H), 4.42 (dd, *J* = 12.3, 10.3 Hz, 1H), 3.84 (dd, *J* = 12.3, 6.5 Hz, 1H), 3.68 (dd, *J* = 9.8, 6.9 Hz, 1H), 2.23 (s, 3H), 1.47 (d, *J* = 0.9 Hz, 3H); ^{13}C NMR (100 MHz, 298K, $CDCl_3$) δ 150.07, 142.88, 141.47, 138.46, 137.12, 131.63, 129.87, 129.19, 128.69, 128.62, 128.11, 127.88, 126.69, 124.28, 123.83, 57.75, 51.17, 21.32, 12.82; HRMS (ESI+) exact mass calculated for $[M+H]^+$ ($C_{24}H_{23}N_2O_4S$) requires m/z 435.1373, found m/z 435.1372.

2,2-dibutyl-3-[(4-nitrophenyl)sulfonyl]-5-phenyloxazolidine (7a)

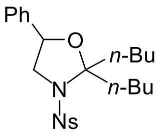

7a

Compound **7a** was synthesized according to the general procedure as a solid (83% yield, 74.1 mg); ^{1}H NMR (400 MHz, 298K, $CDCl_3$) δ 8.41 – 8.32 (m, 2H), 8.09 – 8.02 (m, 2H), 7.40 – 7.29 (m, 5H), 5.09 (dd, *J* = 9.9, 5.8 Hz, 1H), 3.92 (dd, *J* = 8.7, 5.9 Hz, 1H), 3.17 (dd, *J* = 9.7, 9.0 Hz, 1H), 2.18 – 1.93 (m, 4H), 1.46 – 1.22 (m, 7H), 1.08 (1.13 – 1.02, 1H), 0.90 (t, *J* = 7.2 Hz, 3H), 0.84 (t, *J* = 7.3 Hz, 3H); ^{13}C NMR (100 MHz, 298K, $CDCl_3$) δ 146.18, 136.96, 128.77, 128.73, 128.18, 126.14, 124.30, 103.08, 76.90, 54.51, 39.28, 37.77, 26.46, 25.61, 22.88, 22.59, 14.06, 14.04; HRMS (ESI+) exact mass calculated for $[M+H]^+$ ($C_{23}H_{31}N_2O_5S$) requires m/z 447.1948, found m/z 447.1945.

4-[(4-nitrophenyl)sulfonyl]-2-phenyl-1-oxa-4-azaspiro[4.5]decane (7b)

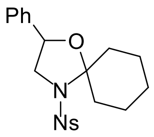

7b

Compound **7b** was synthesized according to the general procedure as a solid (67% yield, 53.8 mg), 00mg); ^{1}H NMR (400 MHz, 298K, $CDCl_3$) δ 8.38 – 8.32 (m, 2H), 8.06 – 8.01 (m, 2H), 7.38 – 7.29 (m, 5H), 5.11 (dd, *J* = 9.5, 5.7 Hz, 1H), 3.99 (dd, *J* = 8.7, 5.7 Hz, 1H), 3.14 (t, *J* = 8.9, 1H), 2.36 – 2.26 (m, 1H), 2.19 (td, *J* = 13.3, 4.4 Hz, 1H), 2.05 – 1.98 (m, 1H), 1.77 – 1.56 (m, 6H), 1.35 – 1.22 (m, 1H); ^{13}C NMR (100 MHz, 298K, $CDCl_3$) δ 149.84, 146.39, 137.12, 128.65, 128.34, 126.01, 124.32, 99.68, 75.86, 54.10, 36.22, 35.46, 24.46, 23.55, 23.40; HRMS (ESI+) exact mass calculated for $[M+H]^+$ ($C_{20}H_{23}N_2O_5S$) requires m/z 403.1322, found m/z 403.1317.

6. Mechanistic Study

(1) Standard condition A

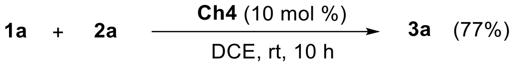

To a reaction mixture of catalyst **Ch4** (52.36 mg, 0.02 mmol) and **1a** (60.81 mg, 0.2 mmol) in a 10 mL-Schlenk tube was added DCE (1.0 mL) under argon atmosphere. Then **2a** (50.45 mg, 0.6 mmol) was added to the above reaction mixture and the reaction was stirred at rt for 10 h. The solvent was removed under reduced pressure and the residue was purified by flash chromatography on silica gel to give the **3a** in 77% yield.

Addition of tetrahydrothiopyran to the standard condition A

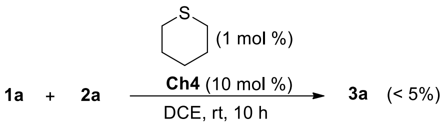

To a reaction mixture of catalyst **Ch4** (52.36 mg, 0.02 mmol) and tetrahydrothiopyran (0.20 mg, 0.002 mmol) in a 10 mL-Schlenk tube was added DCE (1.0 mL) under argon atmosphere. The above reaction mixture was stirred at room temperature for 10 minutes. Then **1a** (60.81 mg, 0.2 mmol) and **2a** (50.45 mg, 0.6 mmol) was added to the above reaction mixture. Then the reaction was stirred at rt for 10 h. Only trace amount of product **3a** was obtained (< 5% yield).

Hydrogen-bonding catalysis

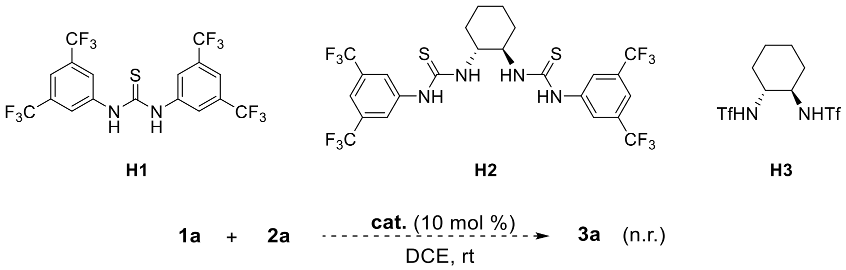

To a reaction mixture of catalyst **H1-3** (0.02 mmol, 10 mol %) and **1a** (60.81 mg, 0.2 mmol) in a 10 mL-Schlenk tube was added DCE (1.0mL) under argon atmosphere. Then **2a** (50.45 mg, 0.6 mmol) was added to the above reaction mixture. The reaction mixture was stirred at rt for 10 h. No reaction took place.

(2) Standard condition B

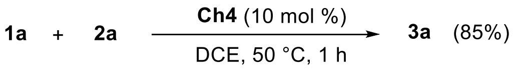

To a reaction mixture of catalyst **Ch4** (52.36 mg, 0.02 mmol) and **1a** (60.81 mg, 0.2 mmol) in a 10 mL-Schlenk tube was added DCE (1.0 mL) under argon atmosphere. Then **2a** (50.45 mg, 0.6 mmol) was added to the above reaction mixture and the reaction was stirred at 50 °C for 1 h. The solvent was removed under reduced pressure and the residue was purified by flash chromatography on silica gel to give the **3a** in 85% yield.

Addition of tetrahydrothiopyran to the standard condition B

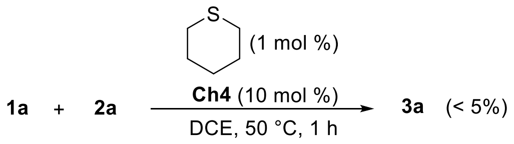

To a reaction mixture of catalyst **Ch4** (52.36 mg, 0.02 mmol) and tetrahydrothiopyran (0.20 mg, 0.002 mmol) in a 10 mL-Schlenk tube was added DCE (1.0 mL) under argon atmosphere. The above reaction mixture was stirred at room temperature for 10 minutes. Then **1a** (60.81 mg, 0.2 mmol) and **2a** (50.45 mg, 0.6 mmol) was added to the above reaction mixture. Then the reaction was stirred at 50 °C for 1 h. Only trace amount of product **3a** was obtained (< 5% yield).

(3) Tracing the reaction process using ^{31}P NMR

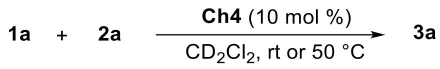

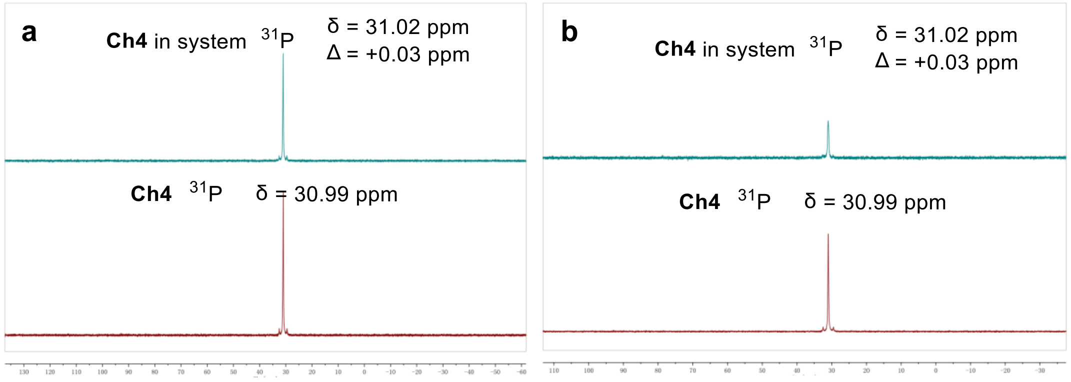

Supplementary Figure 2. Tracing the reaction process using ^{31}P NMR (CD_2Cl_2, 162 MHz, 298K). a The ^{31}P NMR spectrum of **Ch4** in reaction system at room temperature. **b** The ^{31}P NMR spectrum of **Ch4** in reaction system at 50 °C.

Two parallel experiments were performed:

To a reaction mixture of catalyst **Ch4** (52.36 mg, 0.02 mmol) and **1a** (60.81 mg, 0.2 mmol) in a 10 mL-Schlenk tube was added CD_2Cl_2 (1.0 mL) under argon atmosphere. Then **2a** (50.45 mg, 0.6 mmol) was added to the above reaction mixture and the reaction was stirred at room temperature. The reaction process was traced by ^{31}P NMR when the reaction was run for 8 h (**3a**, 63% isolated yield). NMR experiments reveal that only one ^{31}P signal (δ 31.02 ppm)

assigned to catalyst **Ch4** was observed.

Upon conducting the reaction at 50 °C, the reaction process was traced by ^{31}P NMR when the reaction was run for 40 min (**3a**, 76% isolated yield). NMR experiments reveal that only one ^{31}P signal (δ 31.02 ppm) assigned to catalyst **Ch4** was observed.

(4) The electronic effect of the protecting groups of aziridines

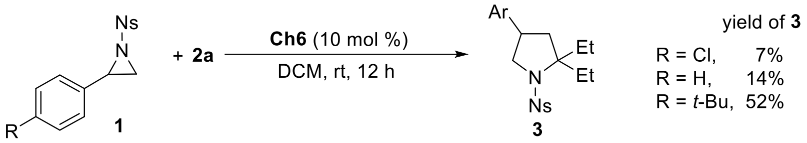

To a reaction mixture of catalyst **Ch6** (24.25 mg, 0.01 mmol) and **1** (0.1 mmol) in a 10 mL-Schlenk tube was added DCM (1.5 mL) under argon atmosphere. Then **2a** (25.23 mg, 0.3 mmol) was added to the above reaction mixture and the reaction was stirred at room temperature for 12 h. The solvent was removed under reduced pressure and the residue was purified by flash chromatography on silica gel to give the distinct yield (52% for *p*-*t*-BuPh; 14% for Ph; 7% for *p*-ClPh).

(5) ^{77}Se NMR studies on the interactions between catalysts and **1a**

To a mixture of catalyst **Ch** (0.05 mmol) and **1a** in an NMR tube was added CD_2Cl_2 (0.5 mL) and then analysis of the reaction mixture by ^{77}Se NMR experiments.

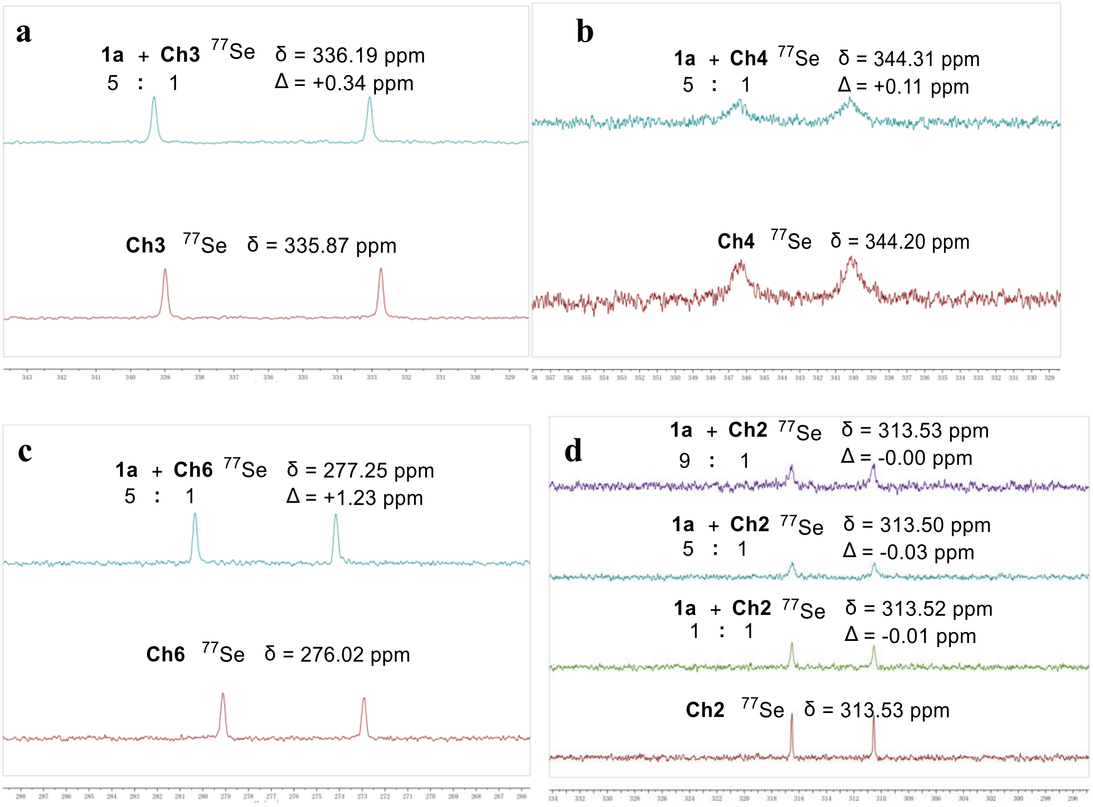

Supplementary Figure 3. The ^{77}Se NMR spectrum of catalyst upon addition of 1a (^{77}Se NMR in CD_2Cl_2, 76 MHz, 298K). a The Δδ ^{77}Se NMR spectrum of catalyst **Ch3** upon addition of **1a**. **b** The Δδ ^{77}Se NMR spectrum of catalyst **Ch4** upon addition of **1a**. **c** The Δδ ^{77}Se NMR spectrum of catalyst **Ch6** upon addition of **1a**. **d** The Δδ ^{77}Se NMR spectrum of catalyst **Ch2** upon addition of **1a**.

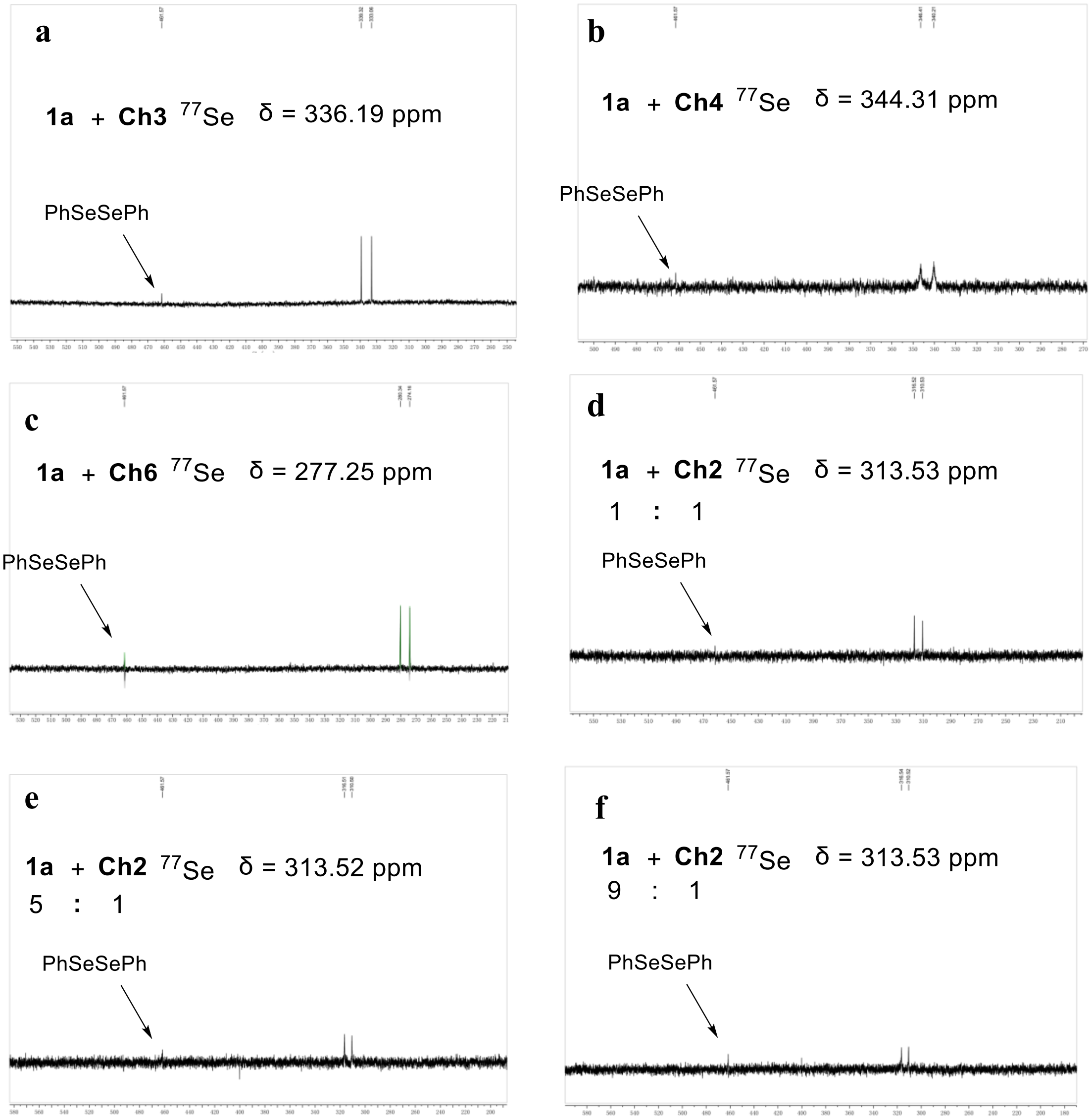

Supplementary Figure 4. The ^{77}Se NMR spectrum of catalyst upon addition of 1a (^{77}Se NMR in CD_2Cl_2, 76 MHz, 298K). a The ^{77}Se NMR spectrum of the mixture of **Ch3** and **1a** (1:5). **b** The ^{77}Se NMR spectrum of the mixture of **Ch4** and **1a** (1:5). **c** ^{77}Se NMR spectrum of the mixture of **Ch6** and **1a** (1:5). **d** The ^{77}Se NMR spectrum of the mixture of **Ch2** and **1a** (1:1). **e** The ^{77}Se NMR spectrum of the mixture of **Ch2** and **1a** (1:5). **f** The ^{77}Se NMR spectrum of the mixture of **Ch2** and **1a** (1:9).

(6) The ^{77}Se NMR studies of catalyst and **1a’**

To a mixture of catalyst **Ch** (0.05 mmol) and **1a’** (0.25 mmol, 64.0 mg) in an NMR tube was added CD_2Cl_2 (0.5 mL) and then analysis of the reaction mixture by ^{77}Se NMR experiments.

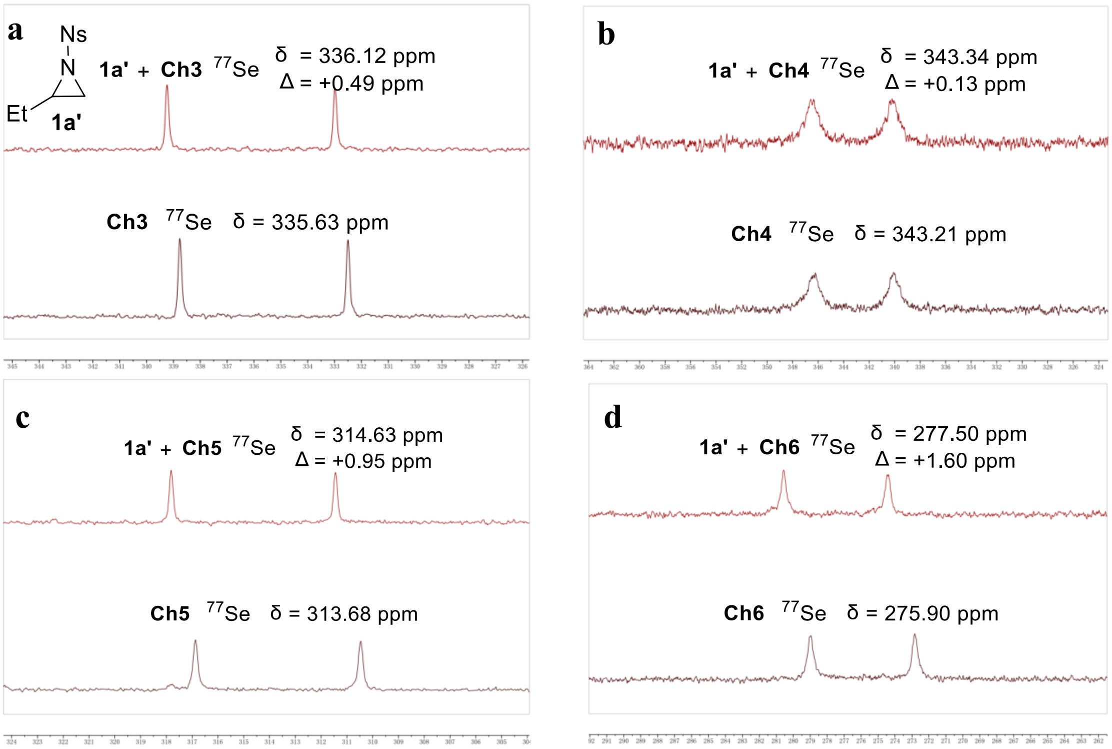

Supplementary Figure 5. The ^{77}Se NMR spectrum of catalyst upon addition of 1a’ (^{77}Se NMR in CD_2Cl_2, 76 MHz, 298K). a The Δδ ^{77}Se NMR spectrum of catalyst **Ch3** upon addition of **1a’**. **b** The Δδ ^{77}Se NMR spectrum of catalyst **Ch4** upon addition of **1a’**. **c** The Δδ ^{77}Se NMR spectrum of catalyst **Ch5** upon addition of **1a’**. **d** The Δδ ^{77}Se NMR spectrum of catalyst **Ch6** upon addition of **1a’**.

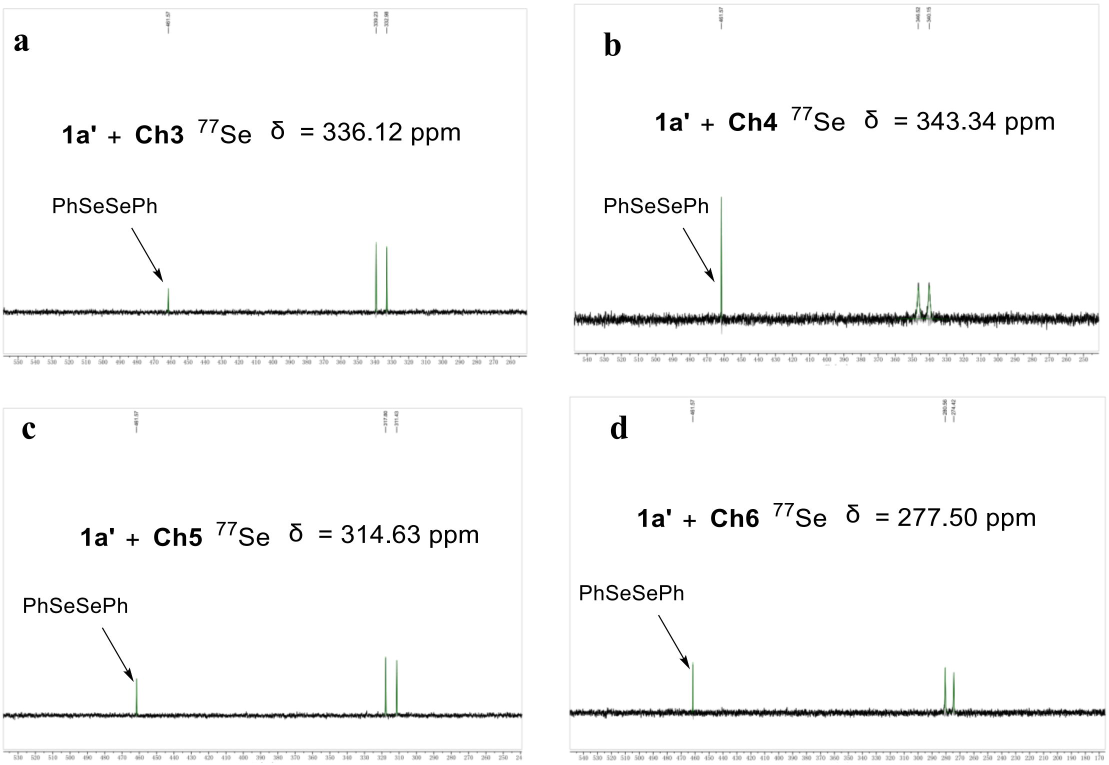

Supplementary Figure 6. The ^{77}Se NMR spectrum of catalyst upon addition of 1a' (^{77}Se NMR in CD_2Cl_2, 76 MHz, 298K). a The ^{77}Se NMR spectrum of the mixture of **Ch3** and **1a'** (1:5). **b** The ^{77}Se NMR spectrum of the mixture of **Ch4** and **1a'** (1:5). **c** The ^{77}Se NMR spectrum of the mixture of **Ch5** and **1a'** (1:5). **d** The ^{77}Se NMR spectrum of the mixture of **Ch6** and **1a'** (1:5).

(7) The interaction between **Ch** and 2-methyl-1-(propylsulfonyl)aziridine (**m1**)

To a mixture of catalyst **Ch** (0.03 mmol for bidentate **Ch5** and **Ch6**; 0.06 mmol for monodentate **Ch2** and **Ch7**) and **m1** (0.03 mmol, 4.89 mg) in an NMR tube was added CD_2Cl_2 (0.5 mL) and then analysis of the reaction mixture by ^{13}C NMR experiments.

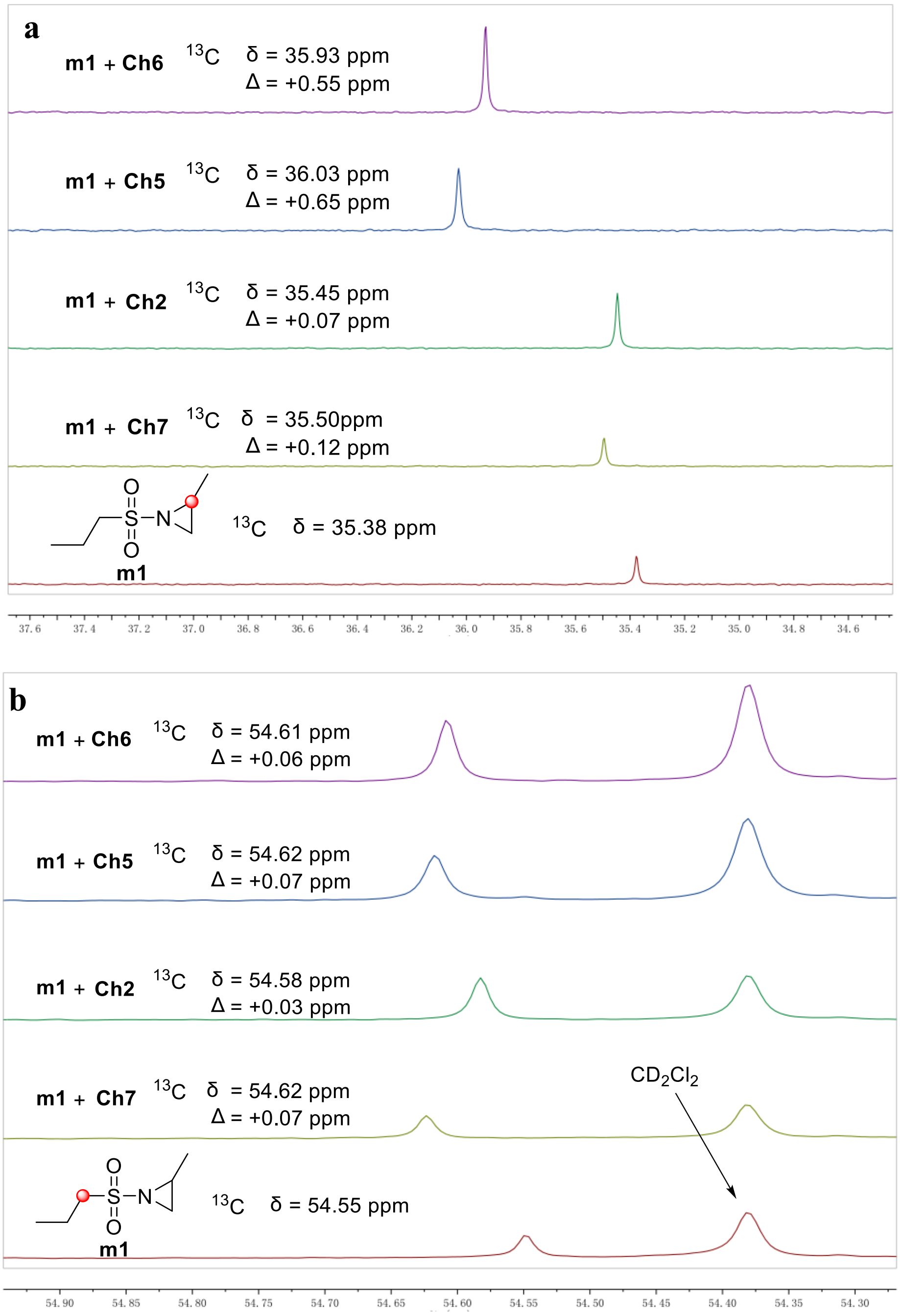

Supplementary Figure 7. The interaction between Ch and m1. a-b The Δδ ^{13}C NMR spectrum of **m1** upon addition of **Ch**. (^{13}C NMR in CD_2Cl_2, 100 MHz, 298K).

(8) The interaction between **Ch** and 1-(propylsulfonyl)aziridine (**m2**)

To a mixture of catalyst **Ch** (0.03 mmol for bidentate **Ch5** and **Ch6**; 0.06 mmol for monodentate **Ch2** and **Ch7**) and **m2** (0.03 mmol, 4.47 mg) in an NMR tube was added CD_2Cl_2 (0.5 mL) and then analysis of the reaction mixture by ^{13}C NMR experiments.

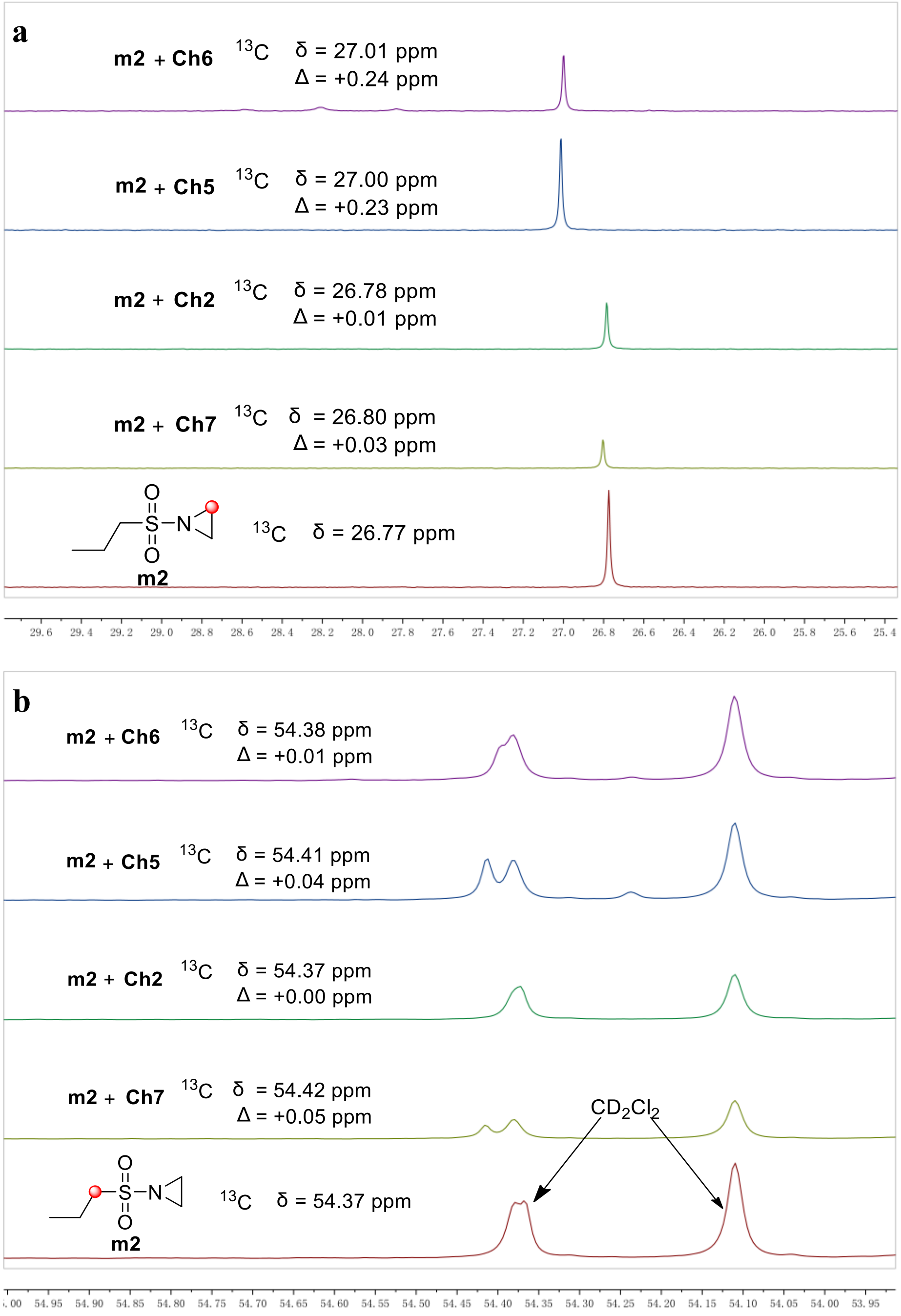

Supplementary Figure 8. The interaction between Ch and m2. a-b The Δδ ^{13}C NMR spectrum of **m2** upon addition of **Ch** (^{13}C NMR in CD_2Cl_2, 100 MHz, 298K).

(9) The interaction between **Ch** and 2-ethyl-1-[(4-nitrophenyl)sulfonyl]aziridine (**1a'**)

To a mixture of catalyst **Ch** (0.03 mmol for bidentate **Ch3-6**; 0.06 mmol for monodentate **Ch7**) and **1a'** (0.03 mmol, 7.68 mg) in an NMR tube was added CD_2Cl_2 (0.5 mL) and then analysis of the reaction mixture by ^{13}C NMR experiments.

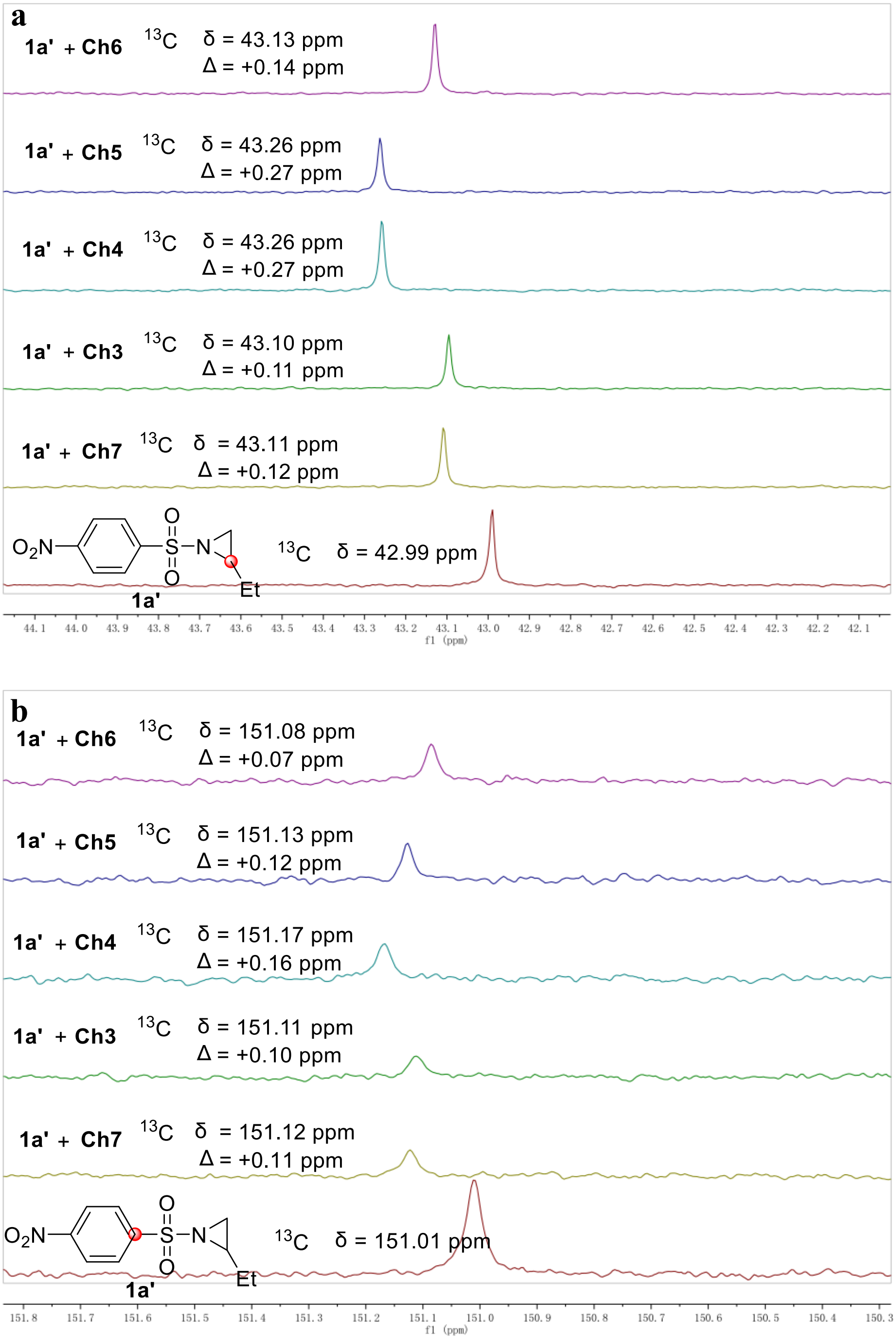

Supplementary Figure 9. The interaction between Ch and 1a'. a-b The Δδ ^{13}C NMR spectrum of **1a'** upon addition of **Ch3-7** (^{13}C NMR in CD_2Cl_2, 100 MHz, 298K).

(10) The interaction between **Ch** and N-methyl-4-nitro-N-propylbenzenesulfonamide (**m3**)

To a mixture of catalyst **Ch** (0.03 mmol for bidentate **Ch3-6**, 0.06 mmol for monodentate **Ch7**) and **m3** (0.03 mmol, 9.12 mg) in an NMR tube was added CD_2Cl_2 (0.5 mL) and then analysis of the reaction mixture by ^{13}C NMR experiments.

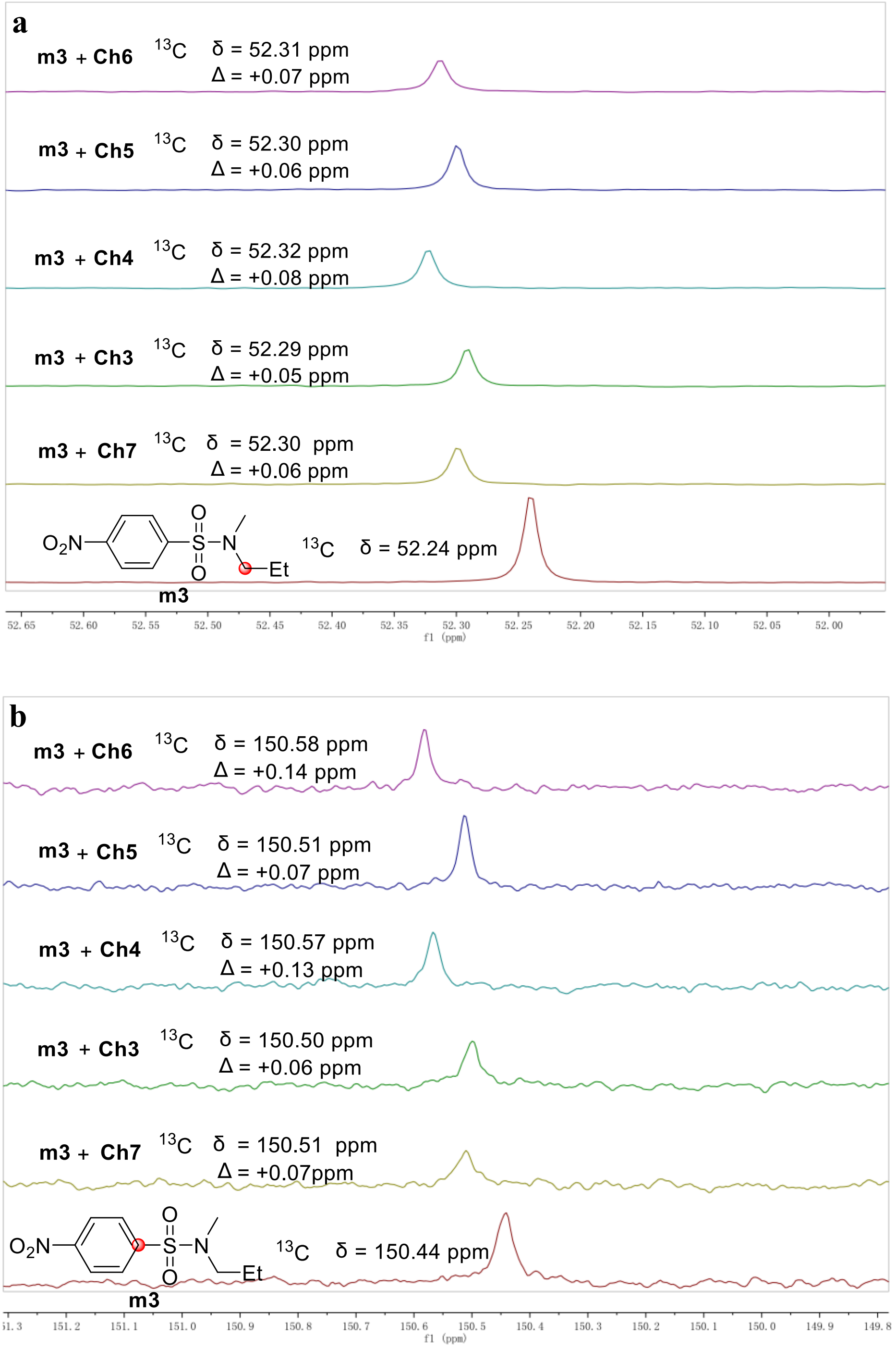

Supplementary Figure 10. The interaction between Ch and m3. a-b The Δδ ^{13}C NMR spectrum of **m3** upon addition of **Ch3-7** (^{13}C NMR in CD_2Cl_2, 100 MHz, 298K).

(11) The interaction between **Ch** and N,N-dimethylpiperidine-1-sulfonamide (**m4**)

To a mixture of catalyst **Ch** (0.03 mmol) and **m4** (0.03 mmol, 5.76 mg) in an NMR tube was added CD_2Cl_2 (0.5 mL) and then analysis of the reaction mixture by ^{13}C NMR experiments.

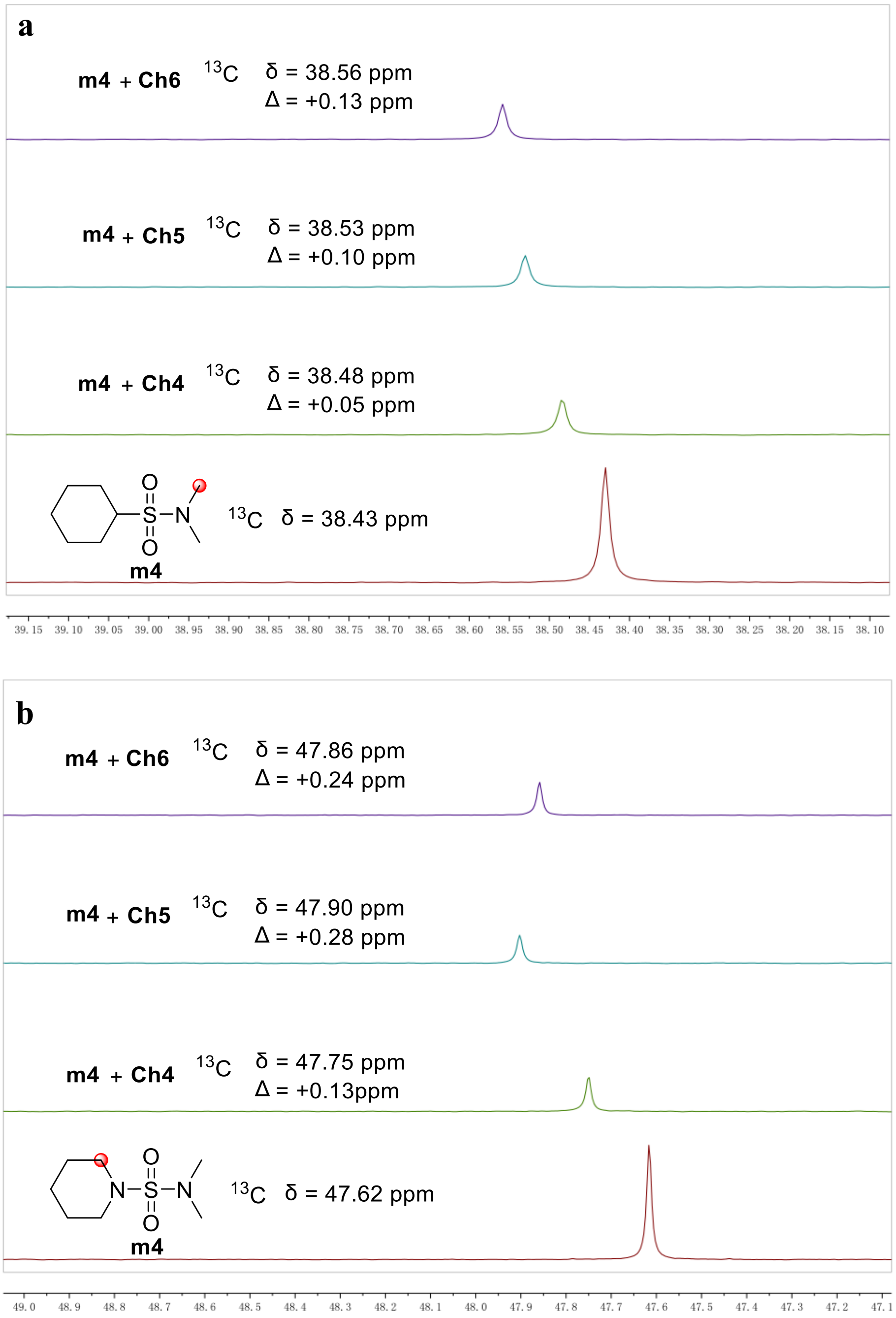

Supplementary Figure 11. The interaction between Ch and m4. a-b The Δδ ^{13}C NMR spectrum of **m4** upon addition of **Ch4-6** (^{13}C NMR in CD_2Cl_2, 100 MHz, 298K).

(12) The interaction between **Ch** and 1-[(2-methylaziridin-1-yl)sulfonyl]piperidine (**m5**)

To a mixture of catalyst **Ch** (0.03 mmol) and **m5** (0.03 mmol, 6.12 mg) in an NMR tube was added CD_2Cl_2 (0.5 mL) and then analysis of the reaction mixture by ^{13}C NMR experiments.

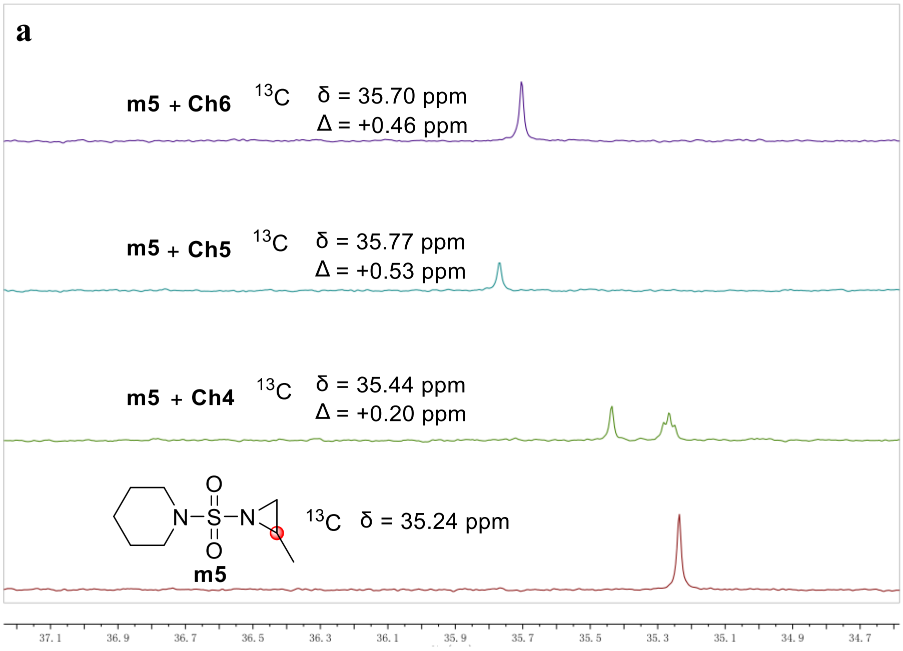

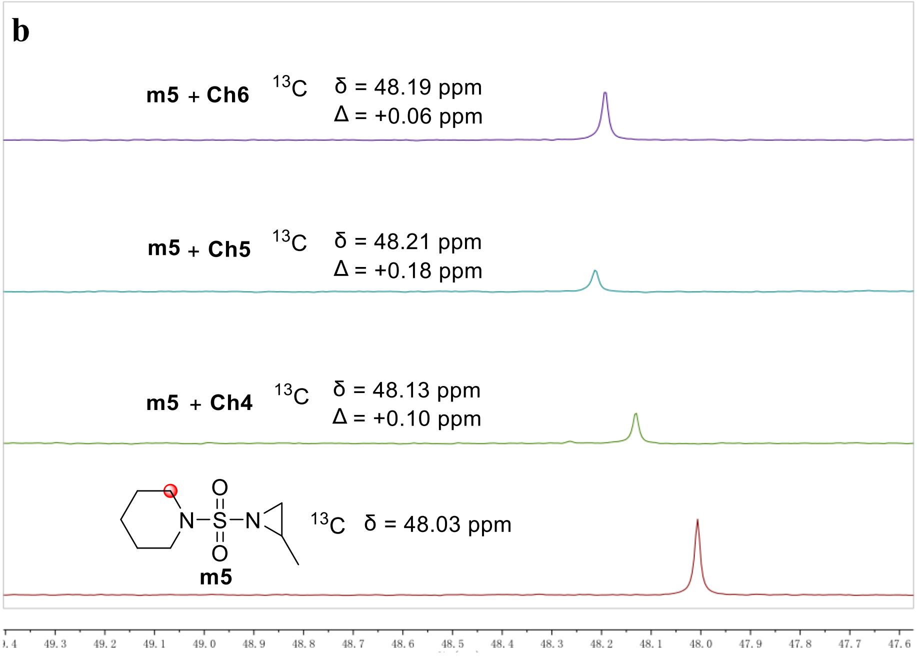

Supplementary Figure 12. The interaction between Ch and m5. a-b The Δδ ^{13}C NMR spectrum of **m5** upon addition of **Ch4-6** (^{13}C NMR in CD_2Cl_2, 100 MHz, 298K).

(13) The interaction between **Ch** and N,N,2-trimethylaziridine-1-sulfonamide (**m6**)

To a mixture of catalyst **Ch** (0.03 mmol) and **m6** (0.03 mmol, 4.92 mg) in an NMR tube was added CD_2Cl_2 (0.5 mL) and then analysis of the reaction mixture by ^{13}C NMR experiments.

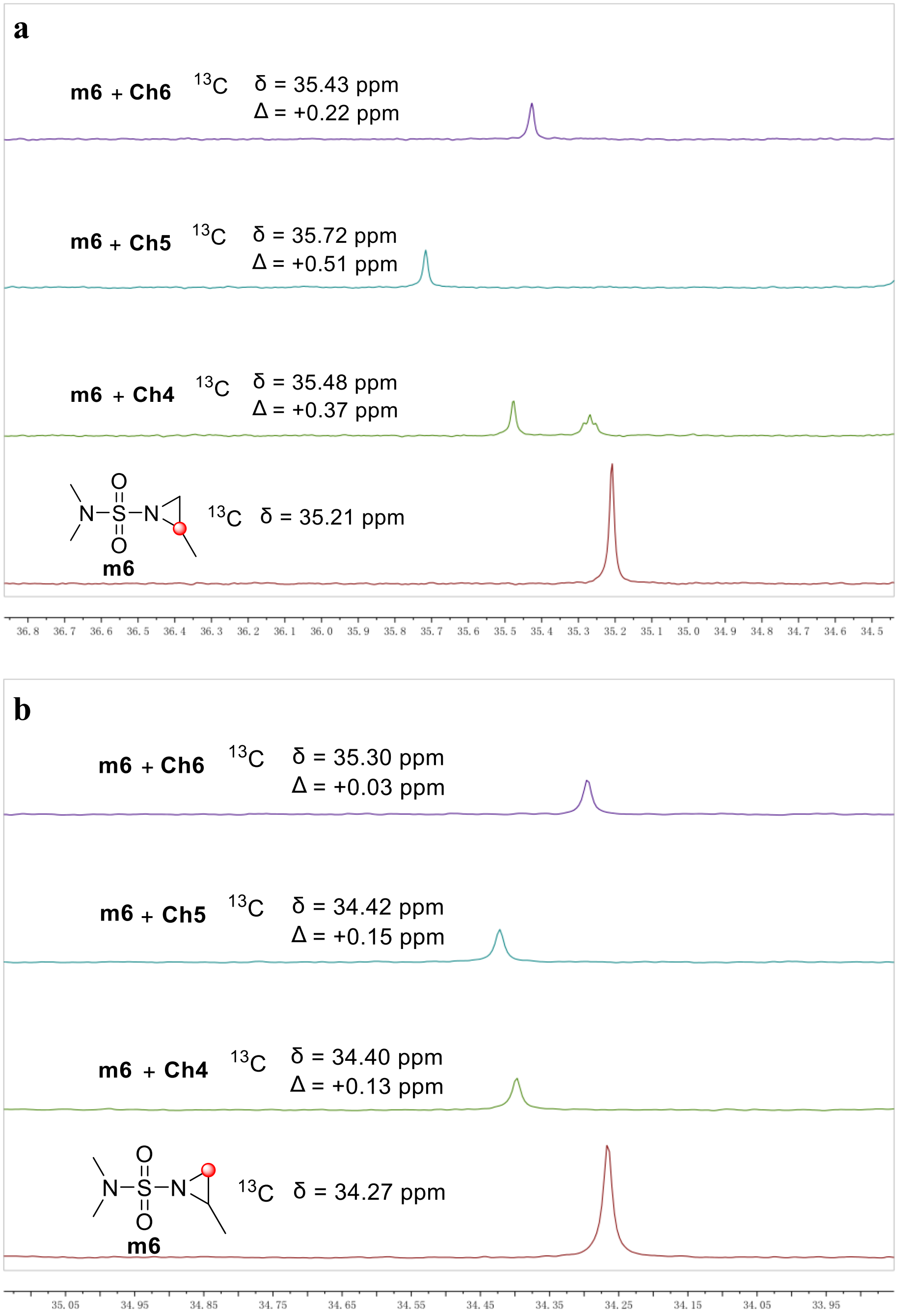

Supplementary Figure 13. The interaction between Ch and m6. a-b The Δδ ^{13}C NMR spectrum of **m6** upon addition of **Ch4-6** (^{13}C NMR in CD_2Cl_2, 100 MHz, 298K).

(14) The interaction between **Ch** and N,N-dimethyl-2-phenylaziridine-1-sulfonamide (**m7**)

To a mixture of catalyst **Ch** (0.03 mmol) and **m7** (0.03 mmol, 6.78 mg) in an NMR tube was added CD_2Cl_2 (0.5 mL) and then analysis of the reaction mixture by ^{13}C NMR experiments.

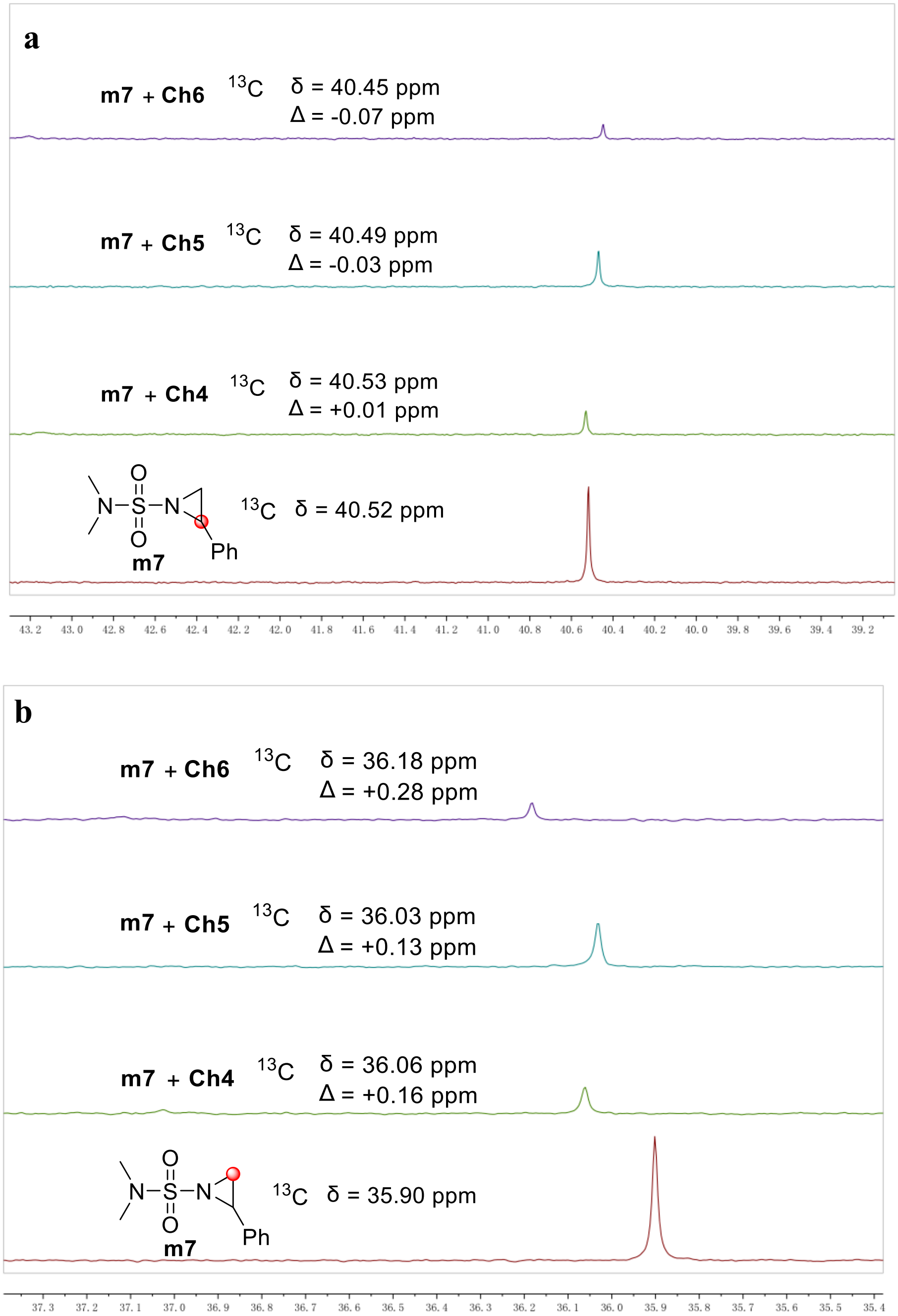

Supplementary Figure 14. The interaction between Ch and m7. a-b The Δδ ^{13}C NMR spectrum of **m7** upon addition of **Ch4-6** (^{13}C NMR in CD_2Cl_2, 100 MHz, 298K).

(15) The interaction between **Ch3/Ch7** and acetone (**m8**)

To a mixture of catalyst **Ch** (0.03 mmol for bidentate **Ch3**; 0.06 mmol for monodentate **Ch7**) and **m8** (0.03 mmol, 1.74 mg) in an NMR tube was added CD_2Cl_2 (0.5 mL) and then analysis of the reaction mixture by ^{13}C NMR experiments.

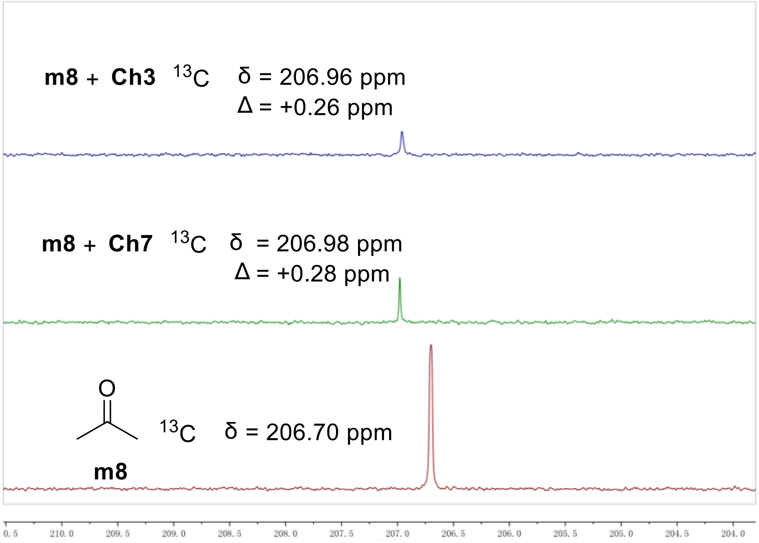

Supplementary Figure 15. The interaction between Ch3/Ch7 and acetone. The variation of ^{13}C NMR of **m8** upon addition of **Ch3/Ch7** (^{13}C NMR in CD_2Cl_2, 100 MHz, 298K).

(16) The interaction between **Ch3/Ch7** and N-ethyl-N-methylpropane-1-sulfonamide (**m9**)

To a mixture of catalyst **Ch** (0.03 mmol for bidentate **Ch3**; 0.06 mmol for monodentate **Ch7**) and **m9** (0.03 mmol, 4.95 mg) in an NMR tube was added CD_2Cl_2 (0.5 mL) and then analysis of the reaction mixture by ^{13}C NMR experiments.

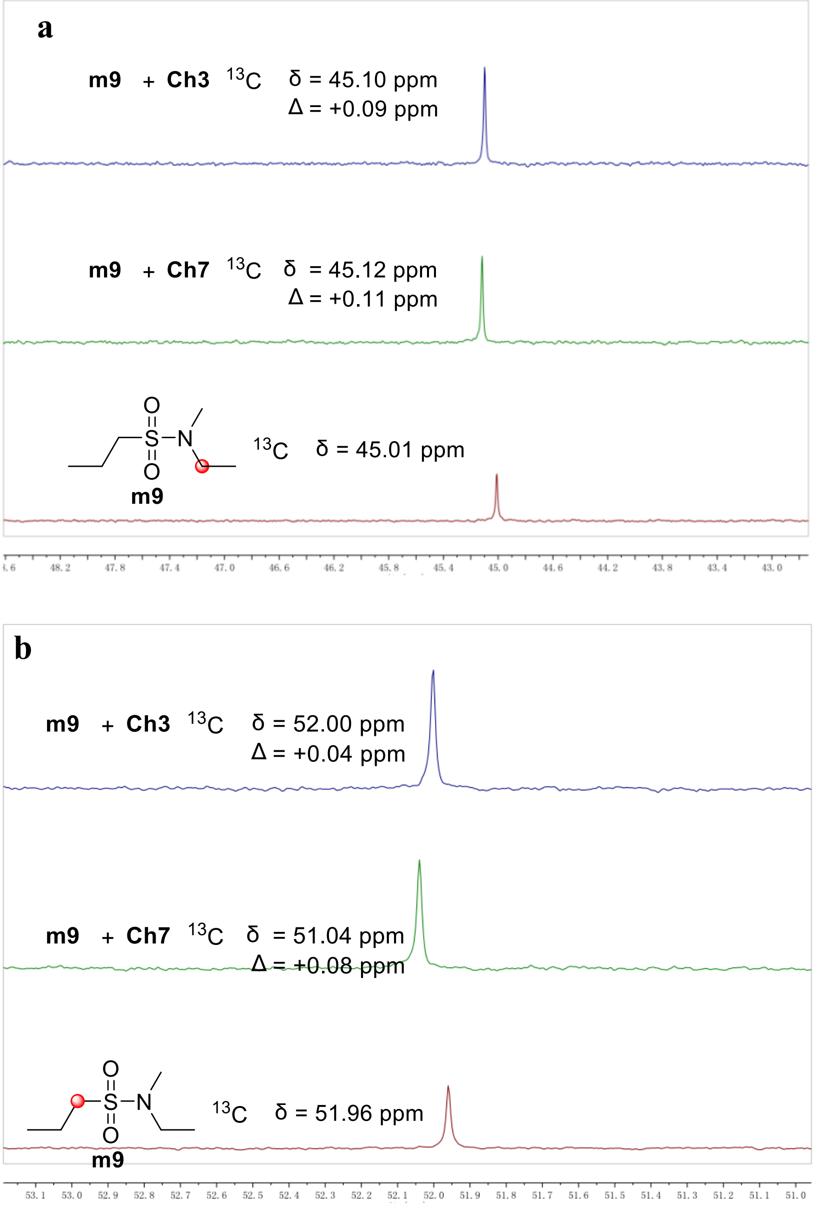

Supplementary Figure 16. The interaction between Ch and m9. a-b The Δδ ^{13}C NMR spectrum of **m9** upon addition of **Ch3/Ch7** (^{13}C NMR in CD_2Cl_2, 100 MHz, 298K).

(17) The interaction between **Ch3/Ch7** and 2-methyl-1-[(4-nitrophenyl)sulfonyl]aziridine (**m10**)

To a mixture of catalyst **Ch** (0.03 mmol for bidentate **Ch3**; 0.06 mmol for monodentate **Ch7**) and **m10** (0.03 mmol, 7.26 mg) in an NMR tube was added CD_2Cl_2 (0.5 mL) and then analysis of the reaction mixture by ^{13}C NMR experiments.

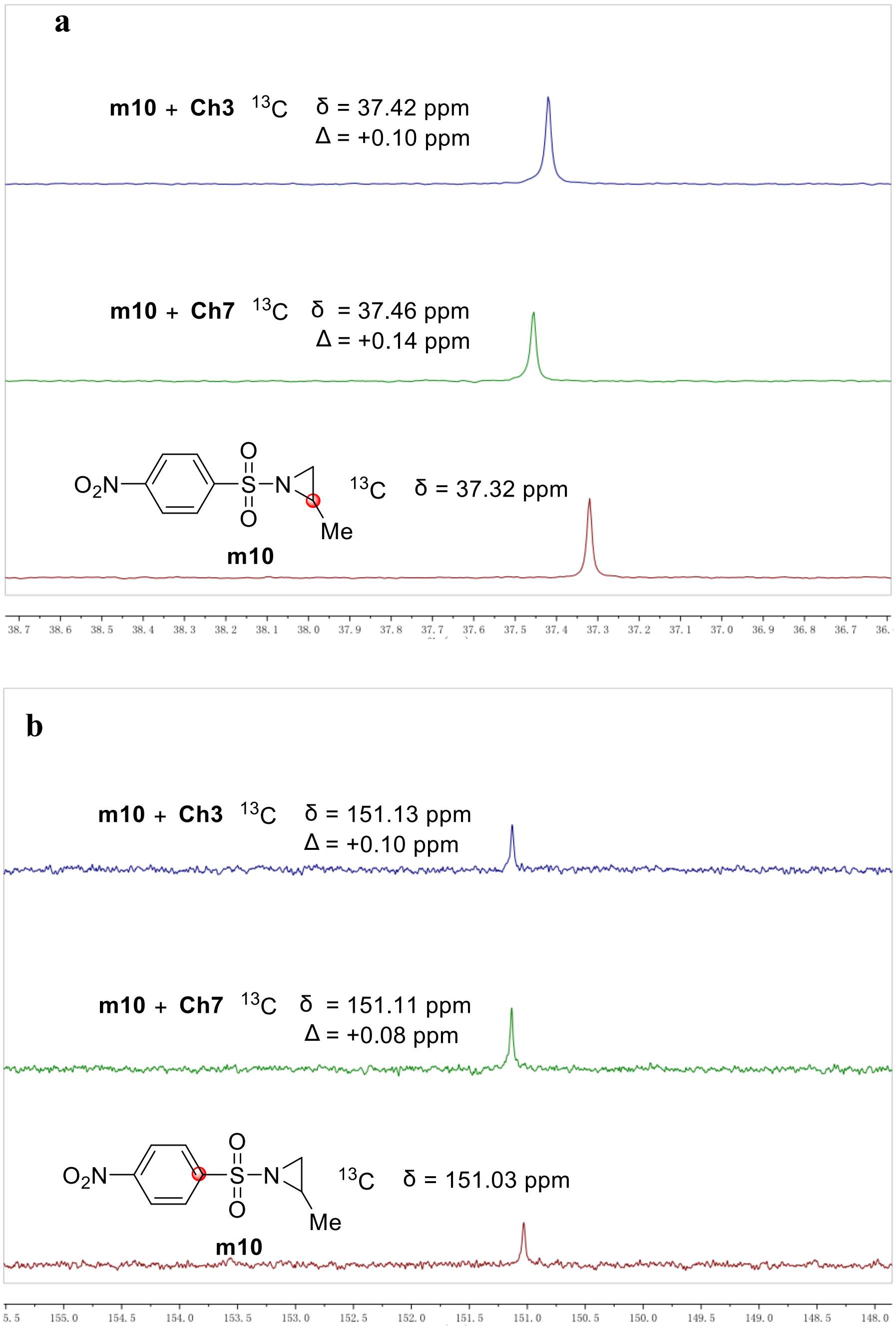

Supplementary Figure 17. The interaction between Ch and m10. a-b The Δδ ^{13}C NMR spectrum of **m10** upon addition of **Ch3/Ch7** (^{13}C NMR in CD_2Cl_2, 100 MHz, 298K).

(18) The interaction between **Ch3/Ch7** and N-ethyl-N-methyl-4-nitrobenzenesulfonamide (**m11**)

To a mixture of catalyst **Ch** (0.03 mmol for bidentate **Ch3**; 0.06 mmol for monodentate **Ch7**) and **m11** (0.03 mmol, 6.72 mg) in an NMR tube was added CD_2Cl_2 (0.5 mL) and then analysis of the reaction mixture by ^{13}C NMR experiments.

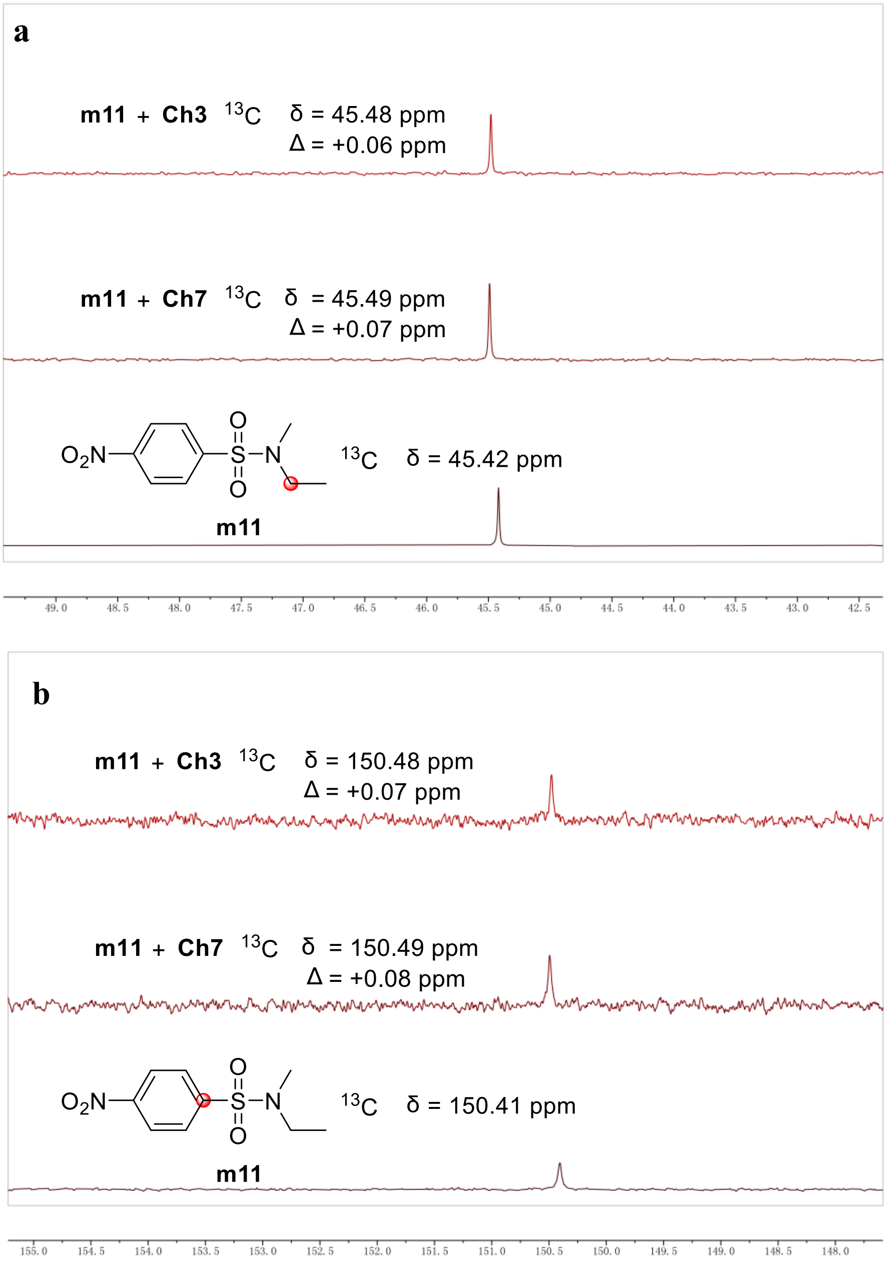

Supplementary Figure 18. The interaction between Ch and m11. a-b The Δδ ^{13}C NMR spectrum of **m11** upon addition of **Ch3/Ch7** (^{13}C NMR in CD_2Cl_2, 100 MHz, 298K).

(19) The interaction between **Ch3/Ch7** and N,N-dimethylpiperidine-1-sulfonamide (**m4**)

To a mixture of catalyst **Ch** (0.03 mmol for bidentate **Ch3**; 0.06 mmol for monodentate **Ch7**) and **m4** (0.03 mmol, 5.76 mg) in an NMR tube was added CD_2Cl_2 (0.5 mL) and then analysis of the reaction mixture by ^{13}C NMR experiments.

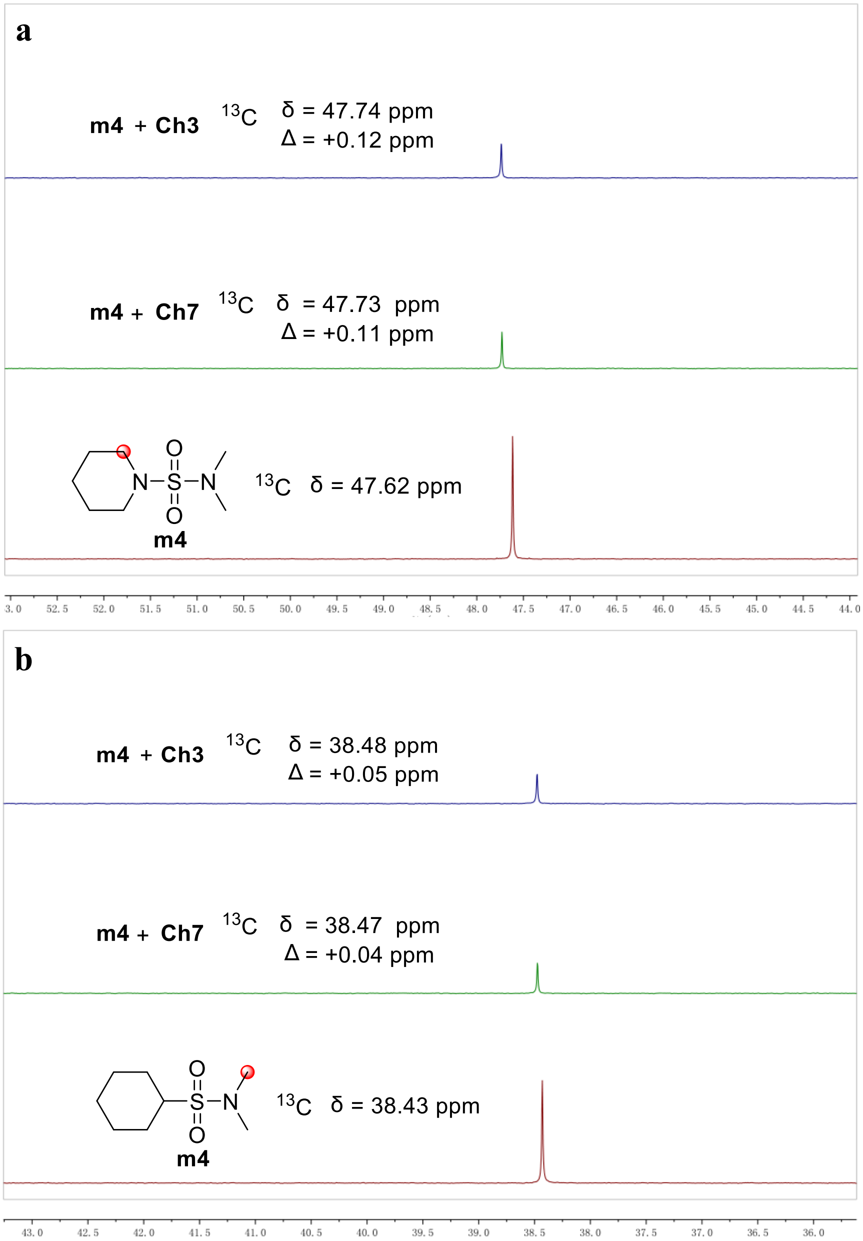

Supplementary Figure 19. The interaction between Ch and m4. a-b The Δδ ^{13}C NMR spectrum of **m4** upon addition of **Ch3/Ch7** (^{13}C NMR in CD_2Cl_2, 100 MHz, 298K).

(20) The interaction between **Ch3/Ch7** and 1-[(2-methylaziridin-1-yl)sulfonyl]piperidine (**m5**)

To a mixture of catalyst **Ch3/Ch7** (0.03 mmol for bidentate **Ch3**; 0.06 mmol for monodentate **Ch7**) and **m5** (0.03 mmol, 6.12 mg) in an NMR tube was added CD_2Cl_2 (0.5 mL) and then analysis of the reaction mixture by ^{13}C NMR experiments.

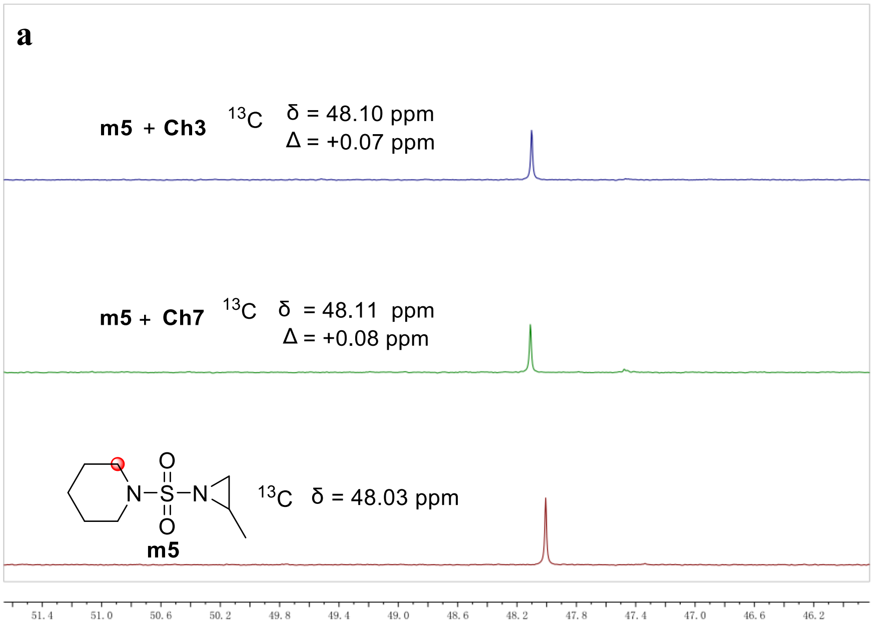

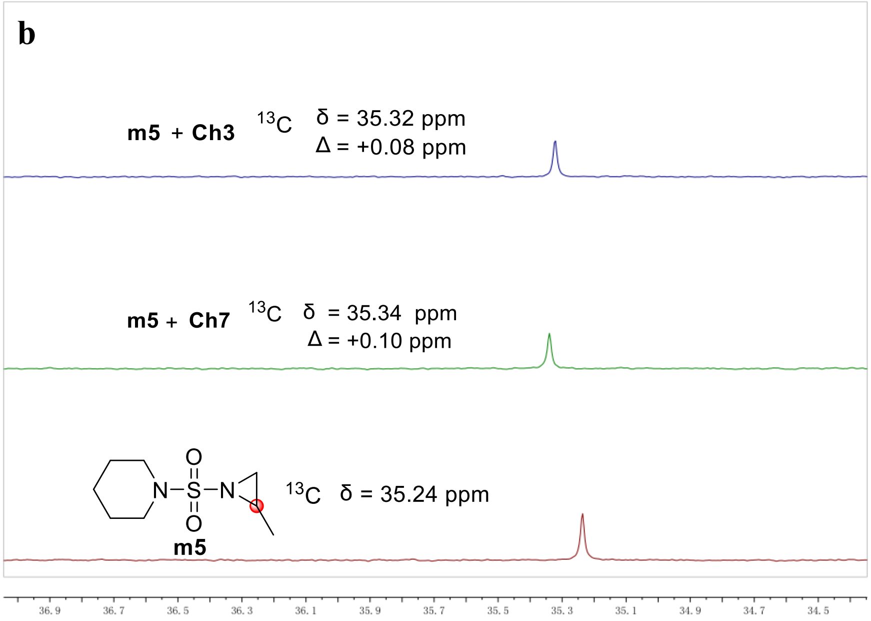

Supplementary Figure 20. The interaction between Ch and m5. a-b The Δδ ^{13}C NMR spectrum of **m5** upon addition of **Ch3/Ch7** (^{13}C NMR in CD_2Cl_2, 100 MHz, 298K).

(21) The interaction between **Ch3/Ch7** and N,N,2-trimethylaziridine-1-sulfonamide (**m6**)

To a mixture of catalyst **Ch** (0.03 mmol for bidentate **Ch3**; 0.06 mmol for monodentate **Ch7**) and **m6** (0.03 mmol, 4.92 mg) in an NMR tube was added CD_2Cl_2 (0.5 mL) and then analysis of the reaction mixture by ^{13}C NMR experiments.

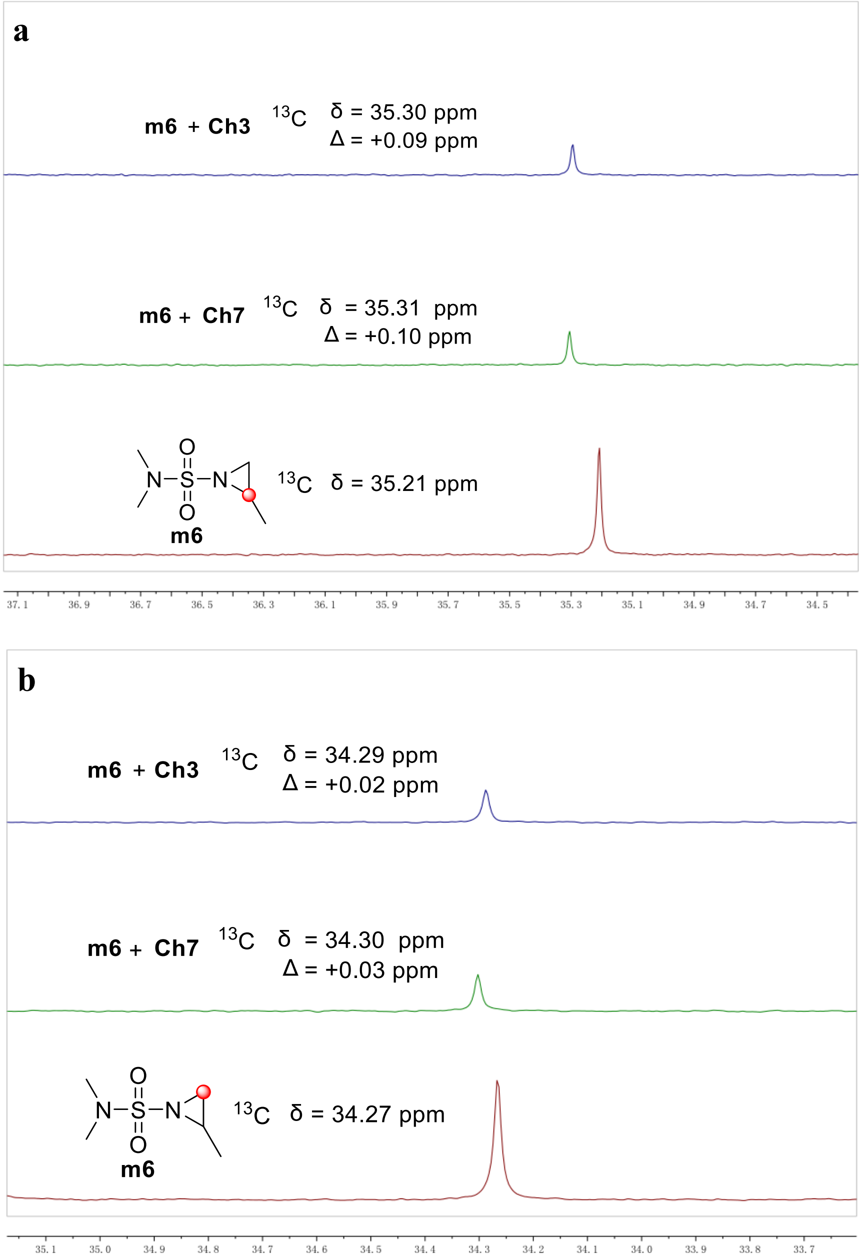

Supplementary Figure 21. The interaction between Ch and m6. a-b The Δδ ^{13}C NMR spectrum of **m6** upon addition of **Ch3/Ch7** (^{13}C NMR in CD_2Cl_2, 100 MHz, 298K).

(22) The interaction between **Ch3/Ch7** and N,N-dimethyl-2-phenylaziridine-1-sulfonamide (**m7**)

To a mixture of catalyst **Ch** (0.03 mmol for bidentate **Ch3**; 0.06 mmol for monodentate **Ch7**) and **m7** (0.03 mmol, 6.78 mg) in an NMR tube was added CD_2Cl_2 (0.5 mL) and then analysis of the reaction mixture by ^{13}C NMR experiments.

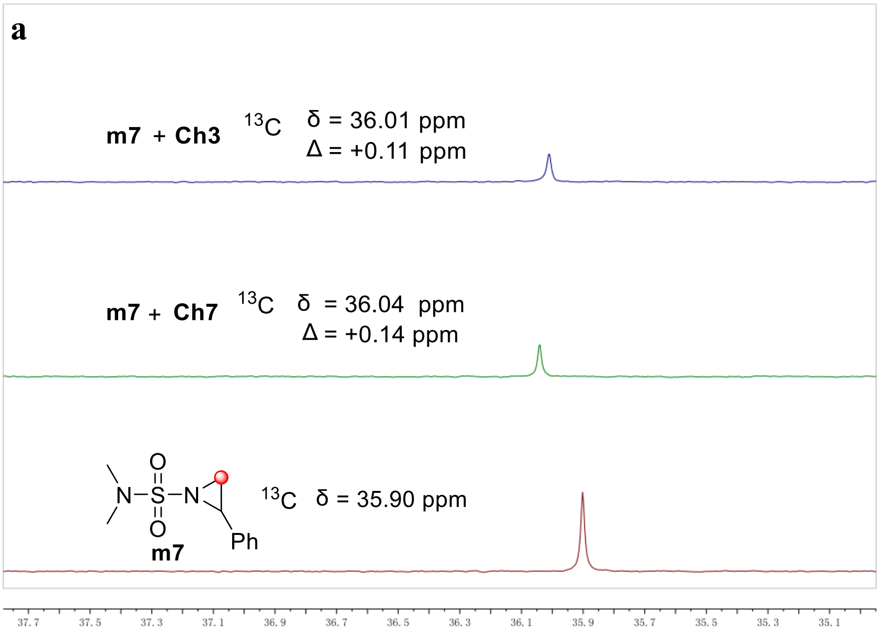

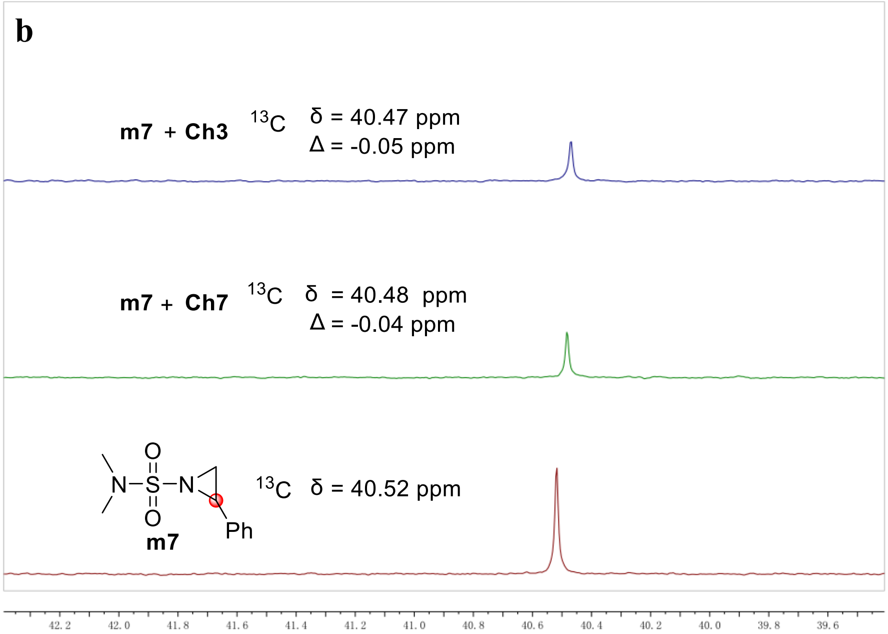

Supplementary Figure 22. The interaction between Ch and m7. a-b The Δδ ^{13}C NMR spectrum of **m7** upon addition of **Ch3/Ch7** (^{13}C NMR in CD_2Cl_2, 100 MHz, 298K).

(23) The interaction between **Ch4** and **2a**

To a mixture of catalyst **Ch4** (0.05 mmol, 130.92 mg) and **2a** (0.05 mmol, 4.20 mg) in an NMR tube was added CD_2Cl_2 (0.5 mL) and then analysis of the reaction mixture by ^{77}Se and ^{31}P NMR experiments.

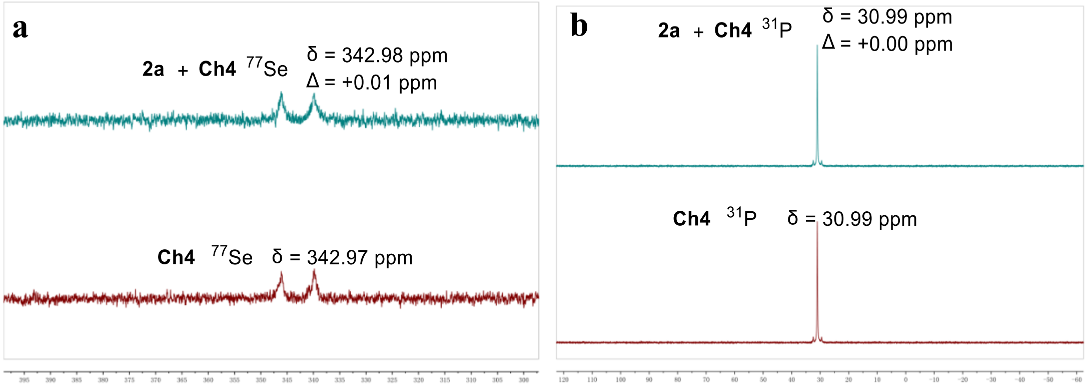

Supplementary Figure 23. The Δδ NMR spectrum of the mixture of Ch4 and 2a. a The Δδ ^{77}Se NMR spectrum of **Ch4** upon addition of **2a**. **b** The Δδ ^{31}P NMR spectrum of **Ch4** upon addition of **2a**.

7. DFT Calculations

Computational details

The binding modes between **Ch5** and aziridine were further assessed via DFT calculations. Two complexes including **SC3** and **SC6** were constructed. The geometries of **Ch5**, aziridine and the two complexes **SC3** and **SC6** were optimized at the M06-2X[2]/6-31g(d,p) level of theory corrected with the Grimme's dispersion (D3)[3] using the Gaussian 09 program[4], and vibrational frequency calculations were also performed at this level. The integral equation formalism model (IEFPCM)[5] in which aniline was chosen as the solvent to simulate solvation effect. Quantum theory of atoms in molecules (QTAIM)[6] analyses were employed to achieve the topology properties at the bond critical points (BCPs) of intermolecular interactions using AIMAll (Version 08.11.06) program.[7] The intermolecular BCPs were plotted by Multiwfn (Version 3.5) software[8]. Natural bond orbital (NBO)[9,10] analysis was carried out to obtain the intermolecular donor-acceptor orbital interaction. The interaction energy (ΔE_{int}) as well as the Gibbs free energy (ΔG) for the complex is calculated as the energy difference between the total energy of the complex and the sum of energies of its components.

As revealed by QTAIM analyses of **SC3** and **SC6** shown in **Supplementary Figure 24** and **Supplementary Table 5**, the intermolecular Se1···O1 and Se1···N1 interactions between **Ch5** and aziridine are observed in **SC3**, while the intermolecular Se1···O1 and Se2···N1 interactions between **Ch5** and aziridine are observed in **SC6**, confirming the existences of intermolecular interactions in the proposed interaction models. NBO analysis of the complex **SC3** (**Fig. 5** in manuscript) shows that the intermolecular donor-acceptor orbital interaction between the first (or third) lone pair orbital of O1 of aziridine and σ*antibonding orbital of Se1-P bond of **Ch5** is weaker which has a stabilization energy less than 0.1 kcal/mol, while the intermolecular donor-acceptor orbital interaction between the lone pair orbital of N1 of aziridine and σ*antibonding orbital of Se1-P bond of **Ch5** is very strong which has a stabilization energy of 3.4 kcal/mol, suggesting the Se1···N1 interactions between **Ch5** and aziridine in **SC3** plays more important role. For **SC6**, the intermolecular donor-acceptor orbital interaction between the first (or third) lone pair orbital of O1 of aziridine and σ*antibonding orbital of Se1-P bond of **Ch5** is very strong which has a stabilization energy of 1.9 (or 4.2) kcal/mol, while the intermolecular donor-acceptor orbital interaction between the lone pair orbital of N1 of aziridine and σ*antibonding orbital of Se2-P bond of **Ch5** has a stabilization energy of 1.5 kcal/mol, indicating that both the intermolecular Se1···O1 and Se2···N1 interactions between **Ch5** and aziridine in **SC6** plays more important role. The interaction energy between **Ch5** and aziridine in **SC6** is stronger than that between **Ch5** and aziridine in **SC3** by 3.1 kcal/mol (**Supplementary Table 6**), and the relative Gibbs free energy for **SC6** is lower

than that for **SC3** by 2.4 kcal/mol, indicating that **SC6** is more favorable complex which forms in the reaction between **Ch5** and aziridine.

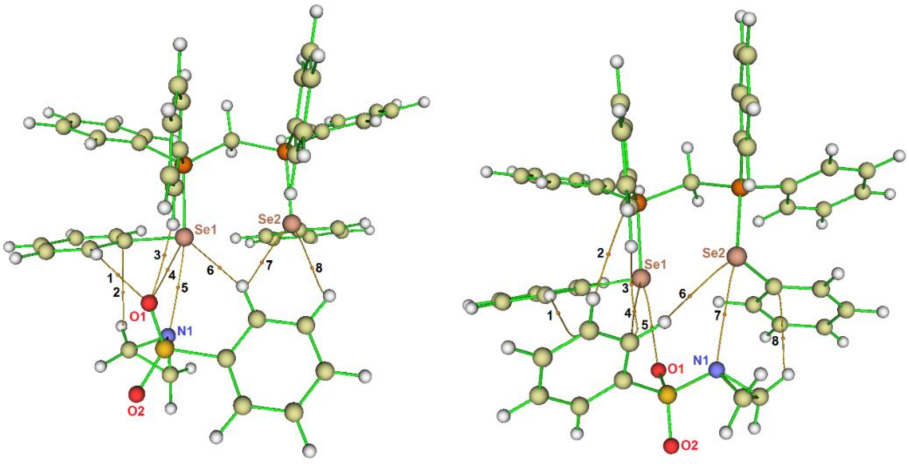

Supplementary Figure 24. DFT calculations of the complex between aziridine and selenide. The intermolecular BCPs for **SC3** and **SC6**.

Supplementary Table 5. Numbers of bond critical points (BCPs No.) and the corresponding distances between atoms (*d*, in Å), classifications of the intermolecular interactions, electron density (*ρ*, in a.u.) and its Laplacian value ($\nabla^2\rho$, in a.u.), the electronic kinetic energy density (*G*, in a.u.), the electronic potential energy density (*V*, in a.u.) and the electronic energy density (*H*, in a.u.) at the BCPs of SC3 and SC6.

	BCPs No.	Classification	d	ρ	$\nabla^2\rho$	G	V	H
**SC3**	1	O···H	2.29	0.0142	0.0474	0.0114	-0.0109	0.0005
	2	C···H	2.79	0.0074	0.0260	0.0051	-0.0038	0.0013
	3	O···H	2.26	0.0133	0.0439	0.0107	-0.0105	0.0002
	4	Se···O	3.29	0.0102	0.0306	0.0071	-0.0065	0.0006
	5	Se···N	3.03	0.0155	0.0441	0.0103	-0.0097	0.0006
	6	Se···H	2.77	0.0103	0.0309	0.0066	-0.0055	0.0011
	7	Se···H	2.73	0.0371	0.0152	0.0078	-0.0062	0.0016
	8	Se···H	2.91	0.0081	0.0273	0.0053	-0.0038	0.0015
**SC6**	1	C···H	2.84	0.0066	0.0199	0.0041	-0.0033	0.0008
	2	H···H	2.45	0.0041	0.0159	0.0029	-0.0019	0.0010
	3	C···H	2.61	0.0087	0.0306	0.0063	-0.0049	0.0014
	4	Se···C	3.39	0.0097	0.0275	0.0057	-0.0046	0.0011
	5	Se···O	2.84	0.0190	0.0593	0.0144	-0.0139	0.0005
	6	Se···H	3.05	0.0058	0.0184	0.0036	-0.0026	0.0010
	7	Se···N	3.25	0.0107	0.0312	0.0071	-0.0064	0.0007
	8	C···H	2.64	0.0095	0.0335	0.0069	-0.0054	0.0015

Supplementary Table 6. The interaction energy (ΔE_{int}, kcal/mol) and the relative Gibbs free energies (ΔG, kcal/mol) for the complexes SC3 and SC6 between Ch5 and aziridine.

Complex	ΔE_{int}	ΔG
**SC3**	-20.6	-5.9
**SC6**	-23.7	-8.3

Supplementary Table 7. Cartesian Coordinates. a Coordinates of aziridine. **b** Coordinates of **Ch5**. **c** Coordinates of Complex **SC3**. **d** Coordinates of Complex **SC6**.

**a**			
S	-0.955484	-0.528679	-0.455408
O	-1.183441	-1.967702	-0.410727
C	0.757925	-0.186365	-0.172418
C	1.472355	-1.020832	0.683996
C	1.338526	0.912171	-0.798747
C	2.812611	-0.736156	0.920567
H	0.986566	-1.874736	1.144361
C	2.681847	1.183064	-0.552391
H	0.748712	1.527498	-1.469603
C	3.412128	0.362945	0.305124
H	3.390796	-1.372806	1.581084
H	3.157121	2.031340	-1.032373
H	4.458648	0.578458	0.493610
C	-3.026545	0.578001	0.805374
C	-1.914687	1.546166	0.944432
H	-3.659835	0.337567	1.650682
H	-3.477921	0.496414	-0.178976
N	-1.642185	0.103457	0.950686
H	-1.719213	2.028060	1.894995
O	-1.399889	0.223796	-1.629143
H	-1.640153	2.100154	0.051276

**b**			
**Se**	1.554500	-1.361300	-1.088300
P	1.354600	0.334300	0.342600
C	3.430300	-1.621400	-0.823500
C	4.329700	-0.952300	-1.651300
H	3.969900	-0.301400	-2.441800
C	2.738400	0.335400	1.468500
C	3.901200	1.019000	1.094500
H	3.923000	1.624600	0.193300
C	2.702300	-0.450400	2.626500
H	1.809100	-0.999400	2.907700

C	3.869300	-2.462700	0.196800
H	3.152100	-2.979200	0.826400
C	5.695500	-1.113200	-1.435200
H	6.405400	-0.593800	-2.069600
C	5.235900	-2.606200	0.412700
H	5.589600	-3.245500	1.214500
C	5.004000	0.131300	3.046100
H	5.892600	0.050300	3.662900
C	6.145100	-1.930500	-0.400400
H	7.210000	-2.044900	-0.227500
C	3.840700	-0.541300	3.417800
H	3.820900	-1.142100	4.319800
C	5.035800	0.907700	1.888800
H	5.942200	1.430200	1.604800
Se	-1.550700	-0.317500	-1.592500
P	-1.736700	0.486300	0.470600
C	-2.214300	2.874100	-0.868700
C	-2.469600	-1.966500	-1.261700
C	-3.849900	-1.989900	-1.449300
H	-4.373200	-1.099900	-1.782900
C	-1.991000	2.260200	0.366600
C	1.193000	3.084000	0.145100
C	-2.420200	4.249900	-0.913900
C	-3.078200	-0.312800	1.340100
C	-2.917100	-1.589600	1.892800
H	-1.958200	-2.095800	1.883400
C	-1.765600	-3.088300	-0.829400
H	-0.693900	-3.035000	-0.665200
C	-4.541700	-3.167700	-1.185000
H	-5.617500	-3.200300	-1.318800
C	0.995500	4.272800	-0.544500
C	-4.329800	0.315100	1.331600
H	-4.451800	1.300900	0.893500
C	-1.980400	3.007900	1.553600
H	-1.825100	2.531800	2.517800
C	0.866800	3.057500	-2.635800
H	0.749700	3.052400	-3.713600
C	-3.852900	-4.298600	-0.748300
H	-4.397000	-5.214000	-0.541800
C	-2.470800	-4.261000	-0.574100
H	-1.938500	-5.142200	-0.232900
C	-2.402900	4.998500	0.258900
H	-2.562200	6.070600	0.216900

C	0.828500	4.257000	-1.930700
H	0.667800	5.189500	-2.461200
C	1.071500	1.857600	-1.961400
H	1.133700	0.918700	-2.502700
C	-5.266100	-1.614700	2.433400
H	-6.122600	-2.126000	2.859500
C	-2.186100	4.379500	1.490900
H	-2.181200	4.964600	2.403400
C	-4.018000	-2.236300	2.438100
H	-3.900600	-3.226500	2.863500
C	-5.421800	-0.342900	1.885800
H	-6.393100	0.138500	1.887200
C	1.224300	1.878100	-0.570800
H	-2.589200	4.732000	-1.870300
H	0.966600	5.210600	-0.001000
C	-0.169200	0.121000	1.354200
H	-0.090500	0.769600	2.234300
H	-0.196200	-0.918300	1.695300
H	1.322200	3.097700	1.224500
H	-2.233200	2.291200	-1.783800

**c**			
**S**	-3.868849	-2.217139	0.447574
O	-3.461977	-1.134047	1.341499
C	-2.734642	-3.561748	0.626129
C	-1.373575	-3.266667	0.604497
C	-3.218786	-4.849202	0.818360
C	-0.466242	-4.303130	0.782436
H	-1.056006	-2.243180	0.436792
C	-2.295919	-5.878602	0.997950
H	-4.287714	-5.032423	0.831465
C	-0.930727	-5.605179	0.980793
H	0.600680	-4.102443	0.764001
H	-2.646702	-6.892771	1.152638
H	-0.218765	-6.411115	1.122408
C	-4.650704	-1.097332	-1.849411
C	-4.141261	-2.437549	-2.213530
H	-4.383644	-0.224475	-2.435220
H	-5.603267	-1.059408	-1.329290
N	-3.512764	-1.681992	-1.117962
H	-3.494435	-2.565139	-3.073199
O	-5.236203	-2.723283	0.526856
Se	-1.267546	0.292966	-0.654630

P	0.011442	1.822985	0.340443
C	-2.717493	1.497118	-1.007188
C	-3.736677	1.650915	-0.070430
H	-3.719580	1.083949	0.853567
C	-0.490801	3.468502	-0.163753
C	-1.498611	4.108946	0.565812
H	-1.884127	3.668043	1.480438
C	0.004494	4.028064	-1.347392
H	0.768943	3.530203	-1.934904
C	-2.723625	2.188723	-2.216972
H	-1.925453	2.046554	-2.938816
C	-4.777116	2.533373	-0.350397
H	-5.576829	2.664847	0.370462
C	-3.763391	3.076631	-2.478514
H	-3.776390	3.631907	-3.410276
C	-1.519631	5.875862	-1.076128
H	-1.924940	6.815387	-1.436118
C	-4.786019	3.248489	-1.546087
H	-5.594617	3.940919	-1.755136
C	-0.508219	5.239559	-1.793776
H	-0.123330	5.680527	-2.706300
C	-2.014500	5.312983	0.098359
H	-2.801794	5.810277	0.653615
Se	1.469594	-1.565002	-0.362854
P	2.684631	0.162115	0.300473
C	2.071637	-0.819840	2.822690
C	1.969850	-1.473859	-2.214808
C	3.105439	-2.178558	-2.611061
H	3.677308	-2.747670	-1.885351
C	2.873466	0.050460	2.076979
C	0.628717	2.399065	2.979877
C	2.205500	-0.845361	4.205256
C	4.270569	0.183508	-0.524978
C	4.428807	0.784202	-1.780037
H	3.623073	1.332580	-2.256674
C	1.205833	-0.737063	-3.117773
H	0.309855	-0.223085	-2.786650
C	3.499444	-2.119495	-3.943738
H	4.384549	-2.657320	-4.265464
C	0.506802	2.220719	4.350831
C	5.312196	-0.555623	0.053060
H	5.183862	-1.030955	1.020634
C	3.813742	0.881401	2.699688

H	4.447288	1.542906	2.115442
C	-1.160665	0.501099	3.996075
H	-1.859183	-0.229104	4.389509
C	2.758714	-1.370820	-4.857220
H	3.071000	-1.325879	-5.895050
C	1.615824	-0.686749	-4.447890
H	1.035022	-0.115481	-5.163778
C	3.139121	-0.021744	4.831137
H	3.242610	-0.050666	5.910538
C	-0.390650	1.276395	4.856608
H	-0.483907	1.147777	5.929966
C	-1.035681	0.652609	2.617708
H	-1.633483	0.047886	1.943581
C	6.680831	-0.073065	-1.872410
H	7.623354	-0.174116	-2.399564
C	3.942714	0.837150	4.082928
H	4.672598	1.470922	4.573605
C	5.641397	0.655424	-2.446755
H	5.768892	1.118566	-3.418444
C	6.517552	-0.676822	-0.626200
H	7.327950	-1.243019	-0.181583
C	-0.144583	1.607469	2.118267
H	1.580501	-1.509602	4.791335
H	1.109225	2.818657	5.025715
C	1.774377	1.678062	-0.168178
H	2.313588	2.548844	0.227295
H	1.789932	1.737490	-1.260821
H	1.318650	3.142233	2.589378
H	1.341902	-1.463924	2.340339
H	-4.756020	-3.289074	-1.936527

**d**			
**S**	-2.304500	-3.422400	0.354900
O	-2.286300	-2.798200	-0.969400
C	-3.062500	-2.339300	1.538300
C	-2.311900	-1.487900	2.345600
C	-4.454700	-2.356700	1.576600
C	-2.986200	-0.651700	3.231300
H	-1.227300	-1.490800	2.293200
C	-5.113600	-1.499100	2.451400
H	-5.012000	-3.039700	0.943500
C	-4.379900	-0.652100	3.279800
H	-2.424200	0.001500	3.890100

H	-6.196900	-1.501200	2.491900
H	-4.893800	0.006100	3.972100
C	-0.033900	-4.751000	0.661500
C	-0.427900	-4.348500	2.027900
H	0.994300	-4.659900	0.331000
H	-0.627000	-5.531200	0.194300
N	-0.704400	-3.455900	0.887500
H	0.310000	-3.943200	2.711600
O	-2.933100	-4.735100	0.470500
H	-1.274000	-4.864700	2.472500
Se	-1.338000	-0.149600	-0.610000
P	-0.313400	1.834600	-0.620900
C	-3.046000	0.498700	-1.166700
C	-3.985500	0.821600	-0.190300
H	-3.740500	0.715600	0.863200
C	-1.178400	2.960700	-1.702800
C	-2.230900	3.713100	-1.168000
H	-2.424100	3.714900	-0.099000
C	-0.931900	2.939600	-3.080100
H	-0.131000	2.336900	-3.497400
C	-3.339600	0.603400	-2.524000
H	-2.595900	0.332300	-3.266400
C	-5.234600	1.293600	-0.584000
H	-5.974600	1.550300	0.166700
C	-4.584300	1.091800	-2.907100
H	-4.820500	1.197700	-3.960600
C	-2.794600	4.436200	-3.398300
H	-3.430000	5.012300	-4.062400
C	-5.527100	1.436900	-1.938800
H	-6.497300	1.814300	-2.244500
C	-1.740700	3.690700	-3.924400
H	-1.552600	3.687200	-4.992000
C	-3.040000	4.448200	-2.026300
H	-3.861000	5.030100	-1.622700
Se	1.388300	-0.972300	0.822800
P	2.526500	0.688900	-0.150000
C	3.028900	1.469500	2.471600
C	1.930900	-2.314300	-0.430400
C	3.074700	-3.044700	-0.118500
H	3.630900	-2.829600	0.787800
C	3.149200	1.797400	1.119200
C	0.443000	3.738400	1.247100
C	3.548100	2.338300	3.427600

C	3.870100	0.023900	-1.126600
C	3.647200	-0.505800	-2.404000
H	2.677000	-0.445500	-2.885000
C	1.182600	-2.562600	-1.579900
H	0.301800	-1.969200	-1.807100
C	3.492500	-4.044400	-0.993200
H	4.385900	-4.616700	-0.768700
C	0.683100	4.171400	2.544700
C	5.120900	-0.097400	-0.507700
H	5.286900	0.305400	0.486800
C	3.792900	2.976600	0.716000
H	3.908500	3.222200	-0.336200
C	-0.256700	2.118200	3.421900
H	-0.523800	1.499500	4.271700
C	2.756800	-4.309100	-2.147100
H	3.080100	-5.092800	-2.823800
C	1.607200	-3.573600	-2.437500
H	1.037800	-3.785400	-3.336000
C	4.180200	3.513800	3.034800
H	4.582700	4.186600	3.784300
C	0.338700	3.359600	3.627300
H	0.538900	3.700200	4.637800
C	-0.508100	1.674000	2.128000
H	-1.006300	0.729700	1.939100
C	5.932300	-1.275500	-2.448000
H	6.737100	-1.787100	-2.964700
C	4.305600	3.831900	1.681600
H	4.807400	4.743800	1.378300
C	4.685100	-1.154800	-3.060100
H	4.517500	-1.571000	-4.047100
C	6.151400	-0.746200	-1.178300
H	7.123200	-0.839600	-0.707100
C	-0.150700	2.484500	1.047700
H	3.453200	2.092100	4.479400
H	1.146300	5.137400	2.712000
C	1.390100	1.600900	-1.276400
H	1.820400	2.586300	-1.492200
H	1.312700	1.048500	-2.216600
H	0.716300	4.366300	0.402800
H	2.543100	0.548400	2.778700

8. X-Ray Crystallographic Data

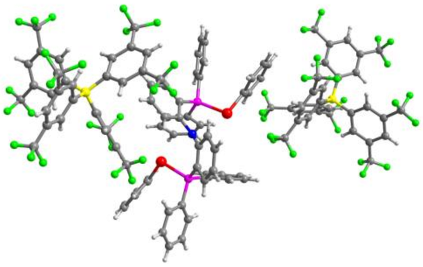

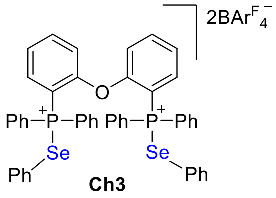

Supplementary Figure 25. X-ray crystallographic structure of Ch3 (CCDC 2069229).

Supplementary Table 8. Crystal data refinement and structure refinement for Ch3.

Identification code	**Ch3**
Empirical formula	$C_{112}H_{62}B_2F_{48}OP_2Se_2$
Formula weight	2577.09
Temperature/K	173(2)
Crystal system	orthorhombic
Space group	Pbca
a/Å	23.0472(3)
b/Å	23.8379(2)
c/Å	39.8684(4)
α/°	90
β/°	90
γ/°	90
Volume/Å^3	21903.5(4)
Z	8
ρ_{calc}mg/mm^3	1.563
μ/mm^{-1}	2.307
F(000)	10256.0
Crystal size/mm^3	0.05 × 0.05 × 0.02
Radiation	CuKα (λ = 1.54184)
2Θ range for data collection	5.776 to 134.16
Index ranges	$-21 \leq h \leq 27$, $-28 \leq k \leq 23$, $-47 \leq l \leq 46$
Reflections collected	109931
Independent reflections	19419 [R_{int} = 0.0649, R_{sigma} = 0.0447]
Data/restraints/parameters	19419/456/1558
Goodness-of-fit on F^2	1.048
Final R indexes [I>=2σ (I)]	R_1 = 0.0892, wR_2 = 0.2337
Final R indexes [all data]	R_1 = 0.1035, wR_2 = 0.2437
Largest diff. peak/hole / e Å^{-3}	2.06/-1.24

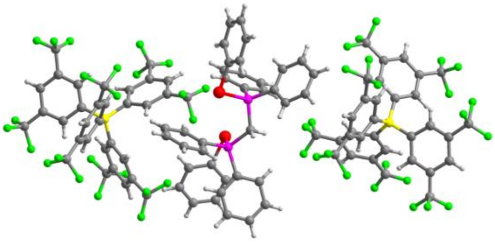

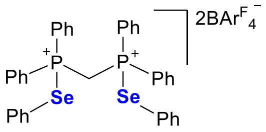

Ch5

Supplementary Figure 26. X-ray crystallographic structure of Ch5 (CCDC 2069224).

Supplementary Table 9. Crystal data refinement and structure refinement for Ch5.

Identification code	**Ch5**
Empirical formula	$C_{101}H_{56}B_2F_{48}P_2Se_2$
Formula weight	2422.93
Temperature/K	173(2)
Crystal system	orthorhombic
Space group	Pbcn
a/Å	19.5049(3)
b/Å	26.1210(5)
c/Å	23.7178(3)
α/°	90
β/°	90
γ/°	90
Volume/Å^3	12083.9(3)
Z	4
ρ_{calc}g/cm^3	1.332
μ/mm^{-1}	2.048
F(000)	4808.0
Crystal size/mm^3	0.05 × 0.04 × 0.03
Radiation	CuKα (λ = 1.54184)
2Θ range for data collection/°	6.768 to 134.158
Index ranges	$-23 \leq h \leq 22$, $-31 \leq k \leq 24$, $-28 \leq l \leq 28$
Reflections collected	39898
Independent reflections	10660 [R_{int} = 0.0791, R_{sigma} = 0.0501]
Data/restraints/parameters	10660/558/888
Goodness-of-fit on F^2	1.067
Final R indexes [I>=2σ (I)]	R_1 = 0.1213, wR_2 = 0.3116
Final R indexes [all data]	R_1 = 0.1351, wR_2 = 0.3227
Largest diff. peak/hole / e Å^{-3}	1.41/-1.50

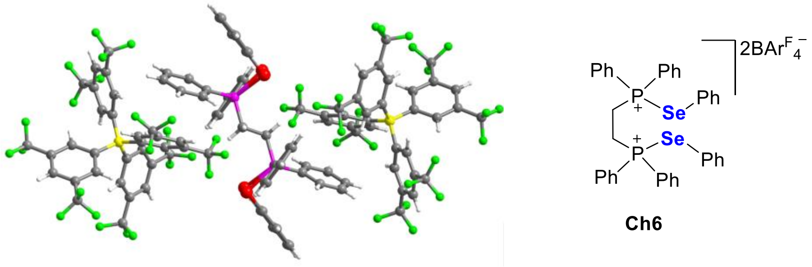

Ch6

Supplementary Figure 27. X-ray crystallographic structure of Ch6 (CCDC 2069228).

Supplementary Table 10. Crystal data refinement and structure refinement for Ch6.

Identification code	**Ch6**
Empirical formula	$C_{102}H_{58}B_2F_{48}P_2Se_2$
Formula weight	2436.96
Temperature/K	173.00(10)
Crystal system	triclinic
Space group	P-1
a/Å	11.9262(3)
b/Å	13.9272(3)
c/Å	17.9429(4)
α/°	109.921(2)
β/°	98.479(2)
γ/°	105.834(2)
Volume/Å^3	2598.67(11)
Z	1
ρ_{calc}g/cm^3	1.557
μ/mm^{-1}	2.384
F(000)	1210.0
Crystal size/mm^3	0.06 × 0.05 × 0.05
Radiation	CuKα (λ = 1.54184)
2Θ range for data collection/°	7.2 to 134.16
Index ranges	$-14 \leq h \leq 14$, $-16 \leq k \leq 15$, $-20 \leq l \leq 21$
Reflections collected	27168
Independent reflections	9153 [R_{int} = 0.0366, R_{sigma} = 0.0356]
Data/restraints/parameters	9153/144/688
Goodness-of-fit on F^2	1.045
Final R indexes [I>=2σ (I)]	R_1 = 0.0931, wR_2 = 0.2318
Final R indexes [all data]	R_1 = 0.1022, wR_2 = 0.2380
Largest diff. peak/hole / e Å^{-3}	1.62/-1.10

9. Powder X-ray Diffraction (PXRD)

Black (Sim.): simulated pattern derived from the single-crystal structure data
Red (Exp.): PXRD pattern

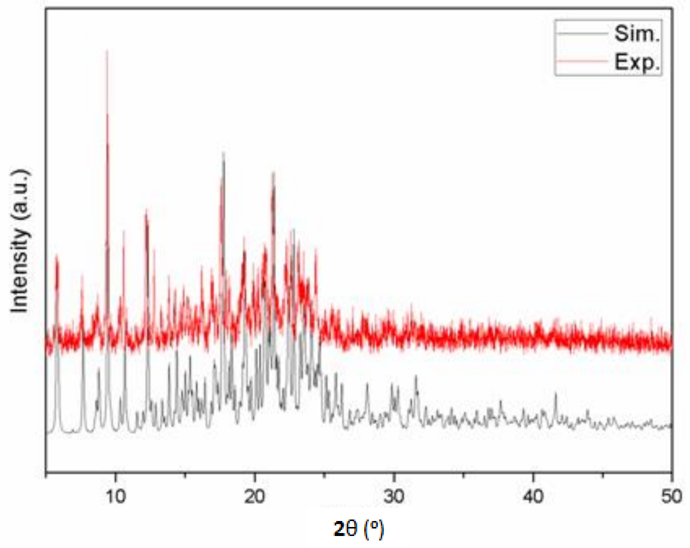

Supplementary Figure 28. PXRD pattern of Ch3 (The data are in agreement with the simulated pattern derived from the corresponding single-crystal X-ray diffraction data).

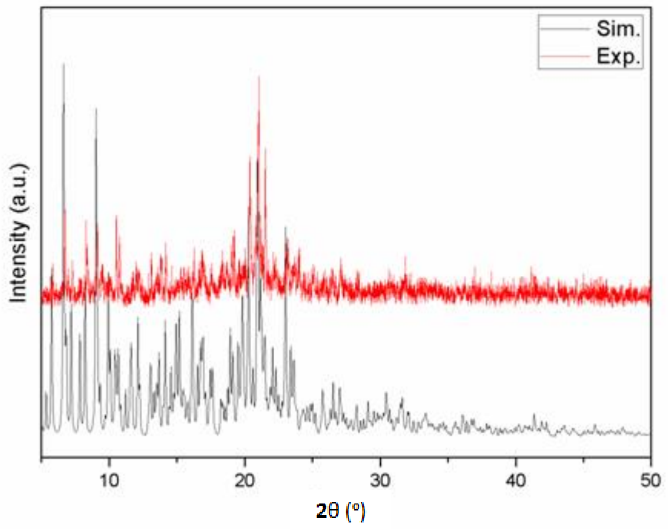

Supplementary Figure 29. PXRD pattern of Ch4 (The data are in agreement with the simulated pattern derived from the corresponding single-crystal X-ray diffraction data).

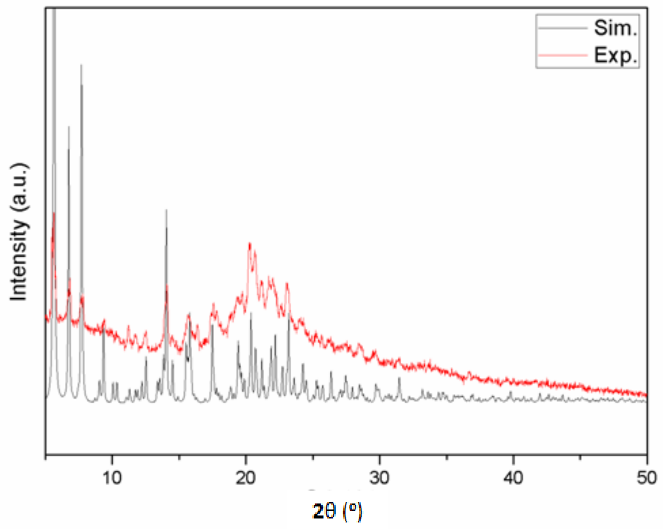

Supplementary Figure 30. PXRD pattern of Ch5 (The data are in agreement with the simulated pattern derived from the corresponding single-crystal X-ray diffraction data).

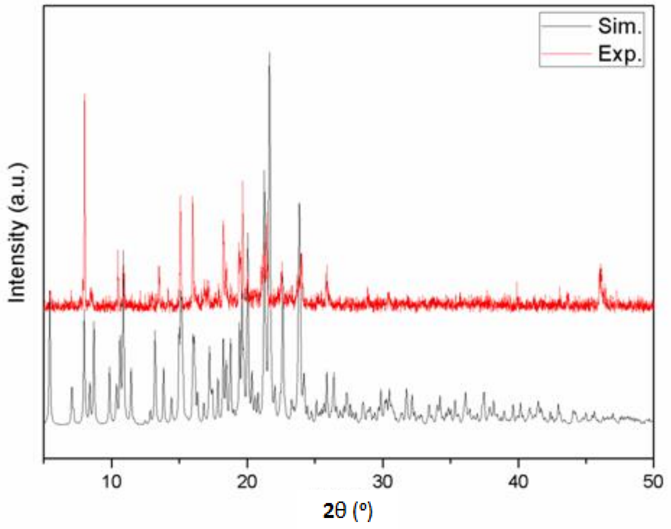

Supplementary Figure 31. PXRD pattern of Ch6 (The data are in agreement with the simulated pattern derived from the corresponding single-crystal X-ray diffraction data).

10. Copies of NMR Spectra

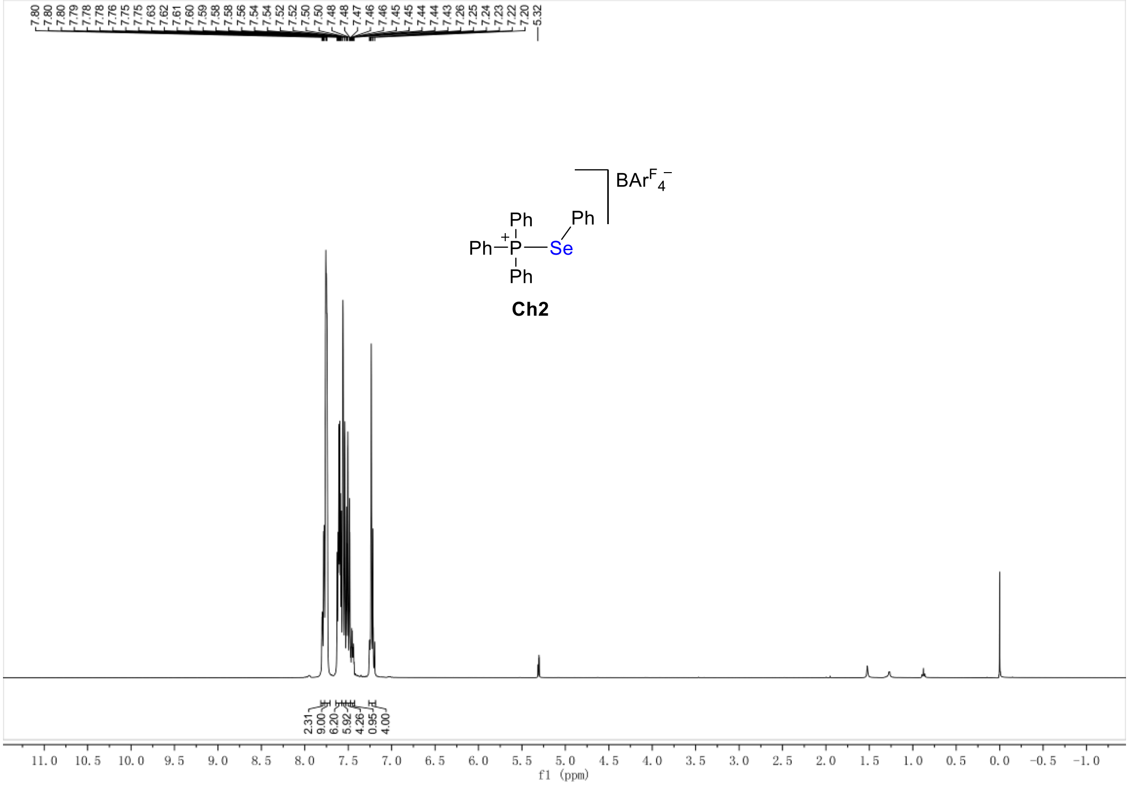

Supplementary Figure 32. ^{1}H NMR spectrum of compound Ch2 ($CDCl_3$, 400 MHz, 298K)

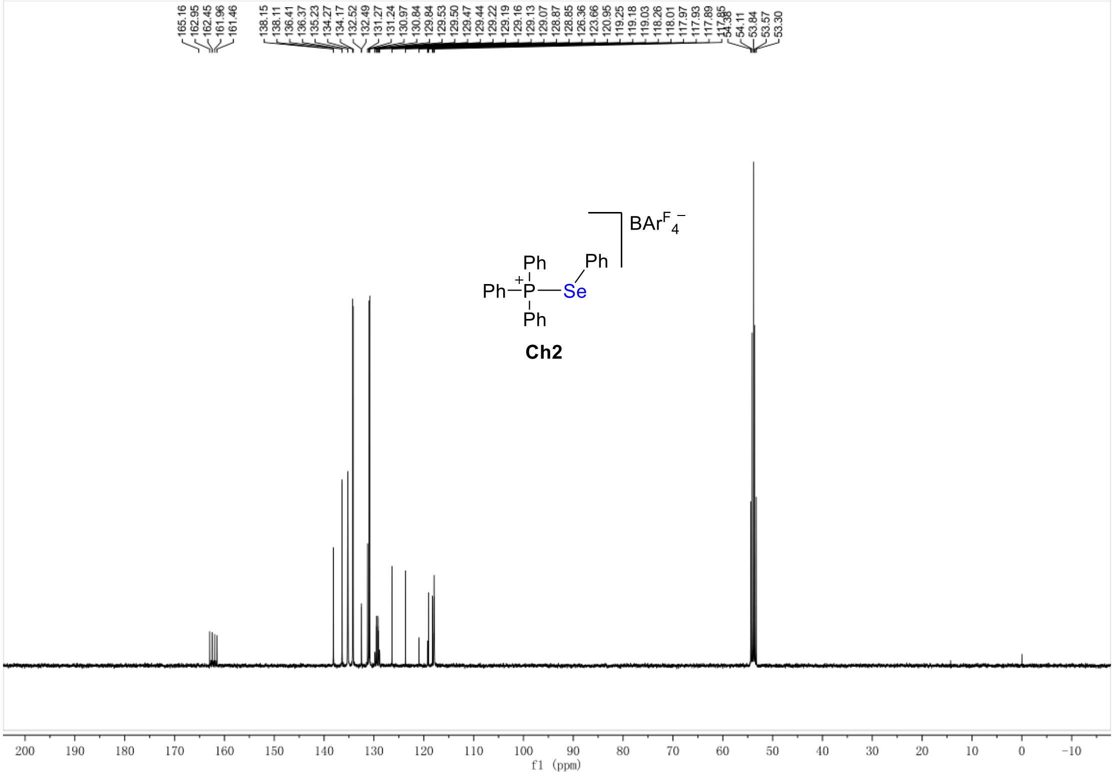

Supplementary Figure 33. ^{13}C NMR spectrum of compound Ch2 ($CDCl_3$, 100 MHz, 298K)

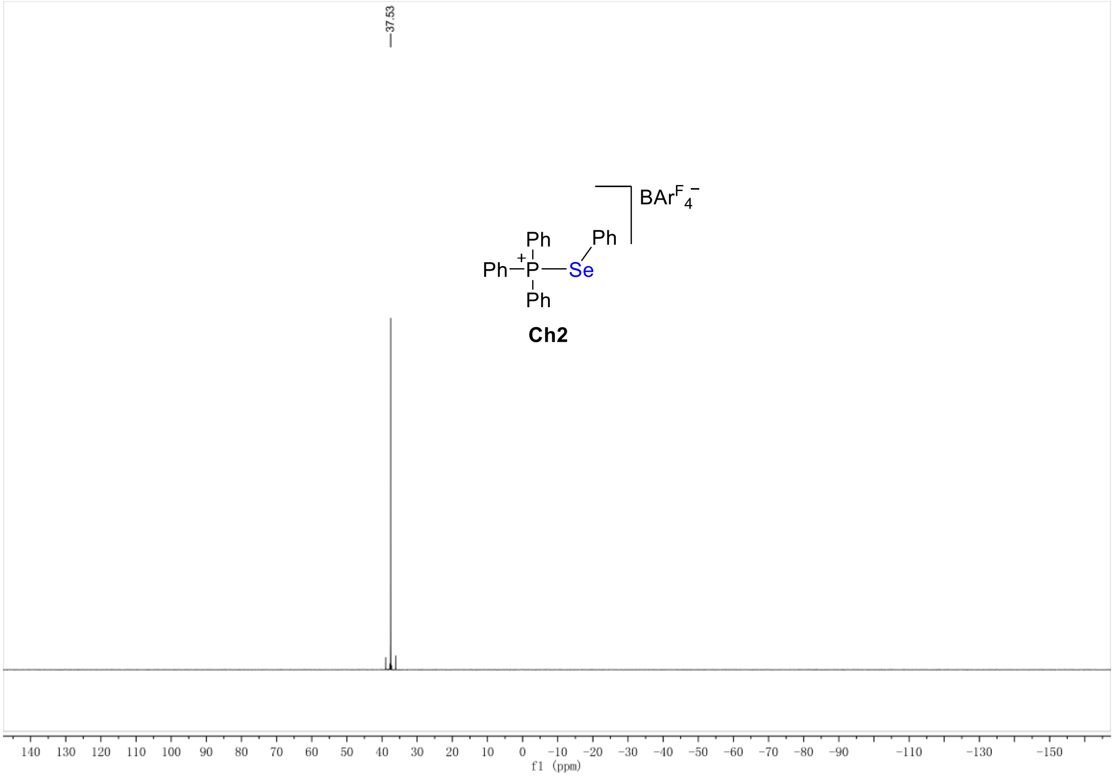

Supplementary Figure 34. ^{31}**P NMR spectrum of compound Ch2 (CD$_2$Cl$_2$, 162 MHz, 298K)**

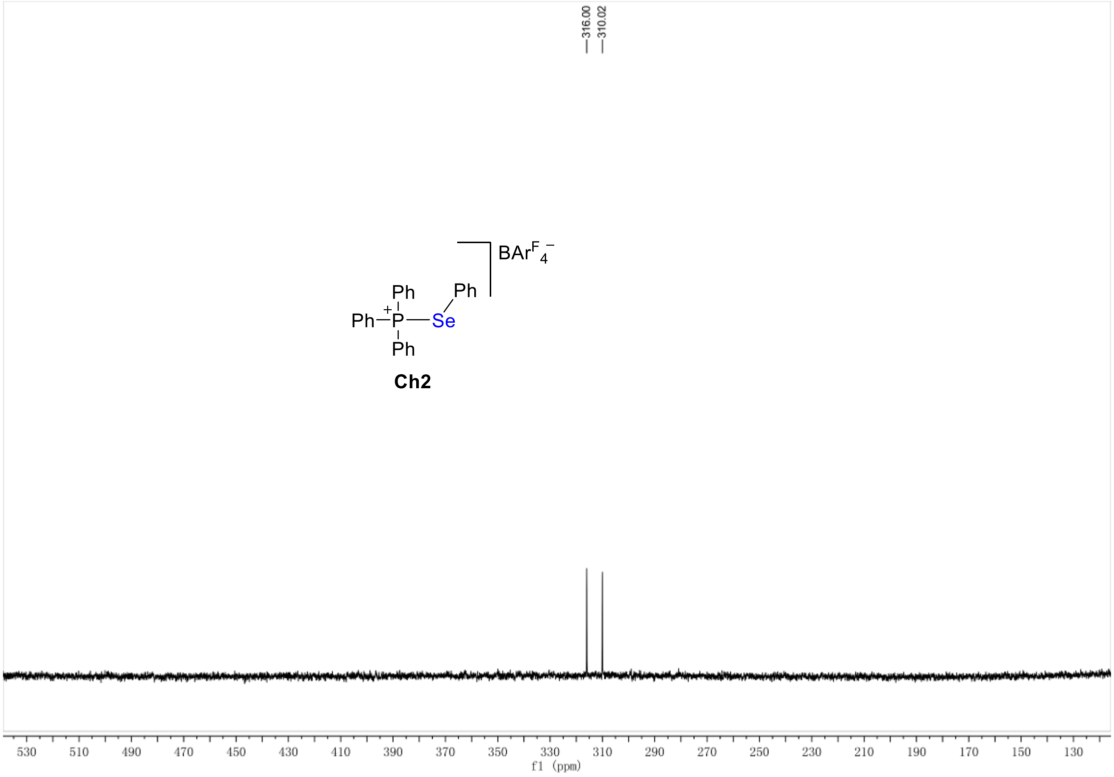

Supplementary Figure 35. ^{77}**Se NMR spectrum of compound Ch2 (CD$_2$Cl$_2$, 76 MHz, 298K)**

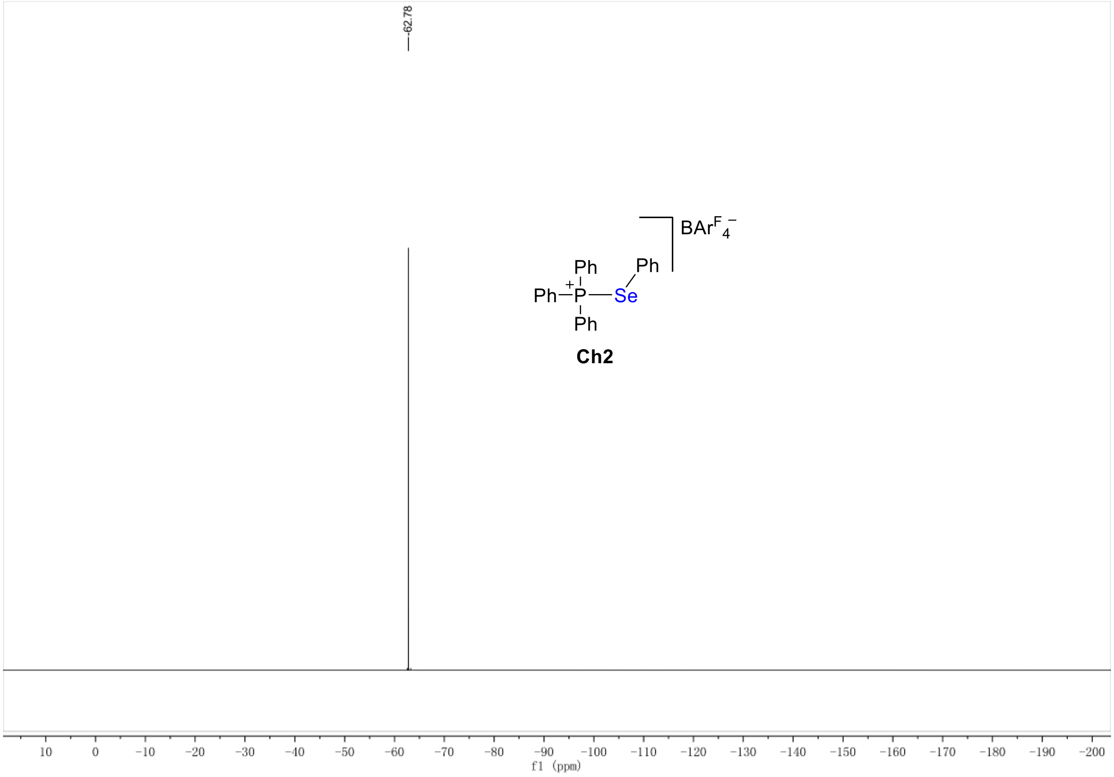

Supplementary Figure 36. **^{19}F NMR spectrum of compound Ch2 (CD_2Cl_2, 376 MHz, 298K)**

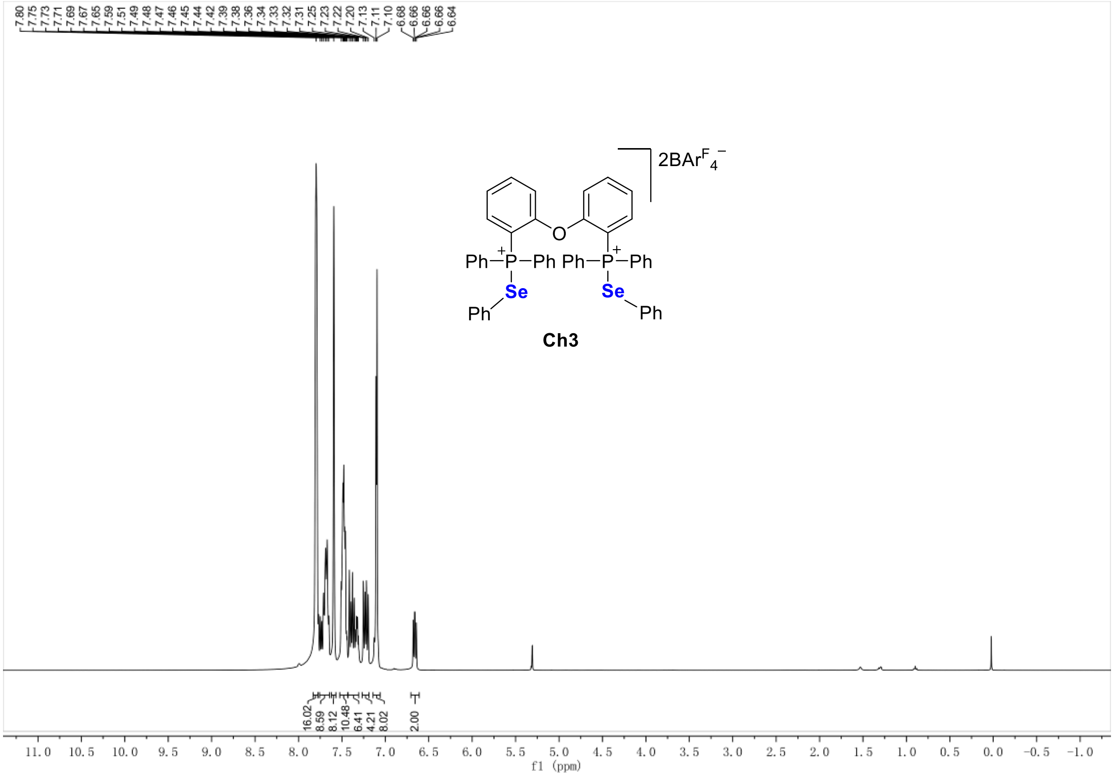

Supplementary Figure 37. **^{1}H NMR spectrum of compound Ch3 (CD_2Cl_2, 400 MHz, 298K)**

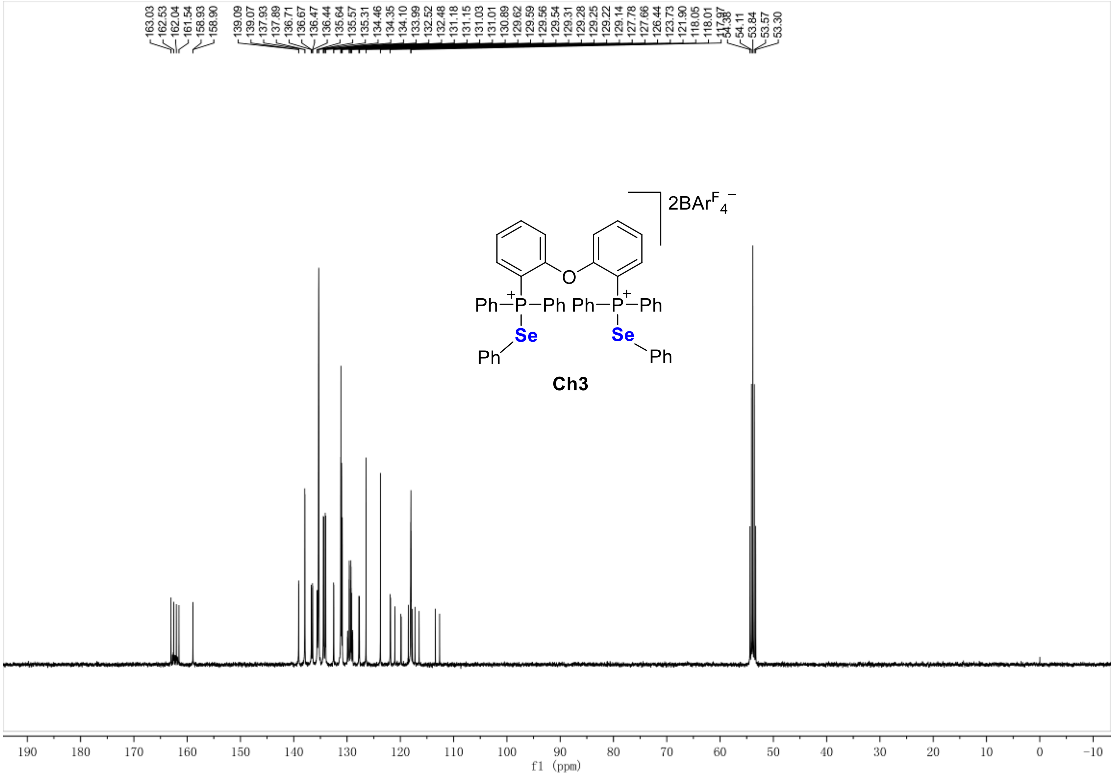

Supplementary Figure 38. ^{13}C NMR spectrum of compound Ch3 (CD_2Cl_2, 100 MHz, 298K)

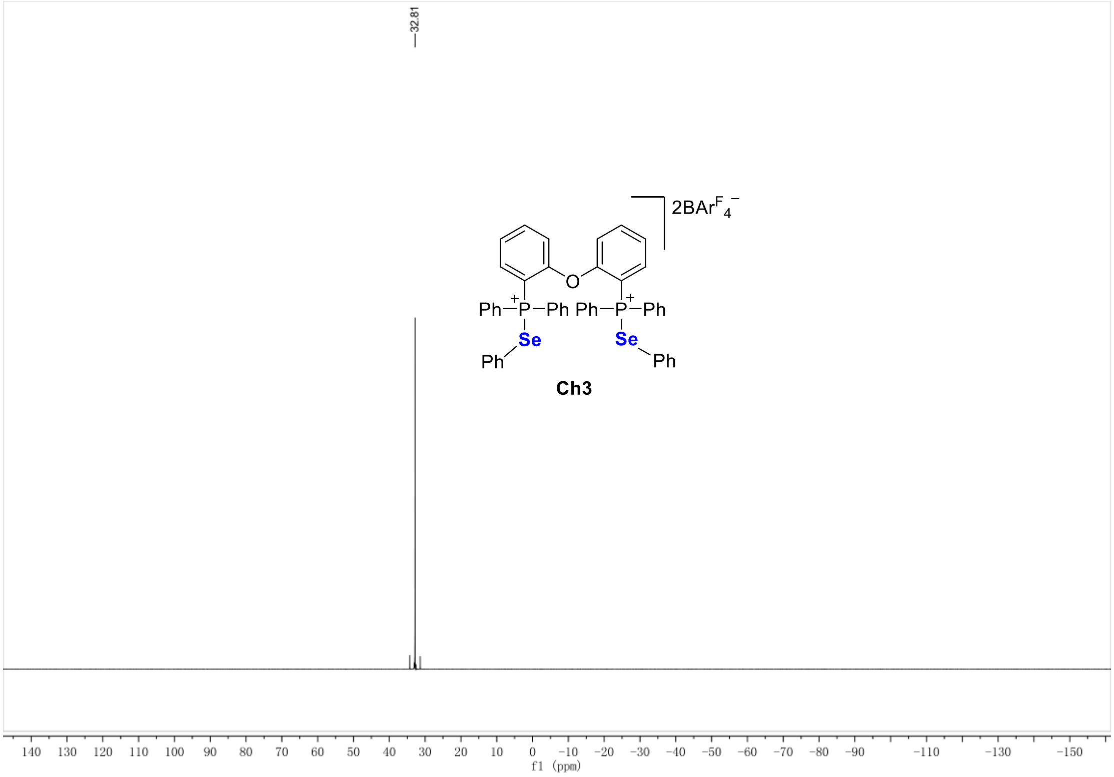

Supplementary Figure 39. ^{31}P NMR spectrum of compound Ch3 (CD_2Cl_2, 162 MHz, 298K)

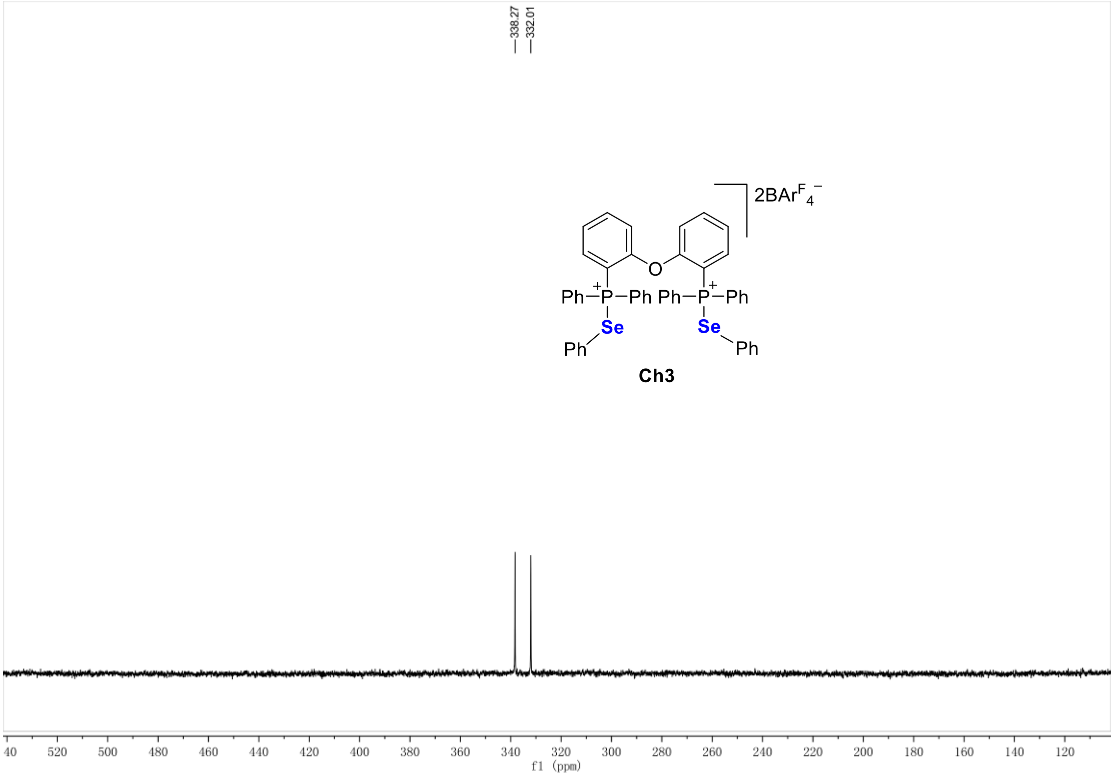

Supplementary Figure 40. ^{77}Se **NMR spectrum of compound Ch3 (**CD_2Cl_2**, 76 MHz, 298K)**

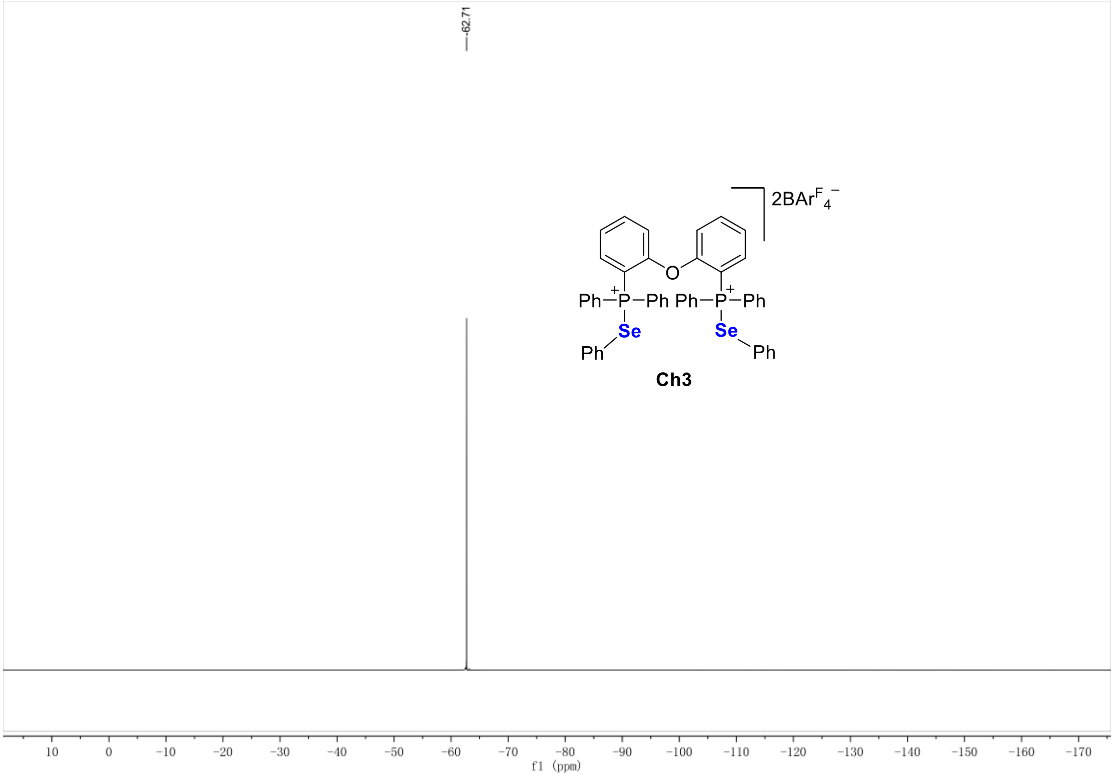

Supplementary Figure 41. ^{19}F **NMR spectrum of compound Ch3 (**CD_2Cl_2**, 376 MHz, 298K)**

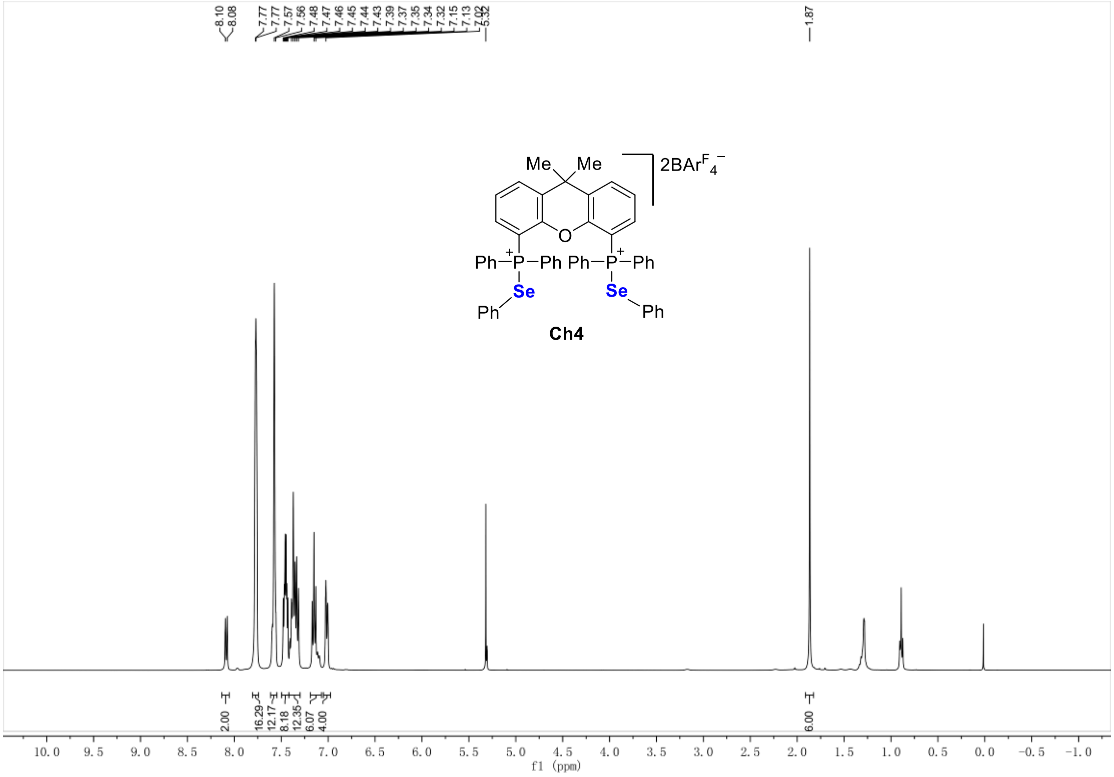

Supplementary Figure 42. ^{1}H NMR spectrum of compound Ch4 (CD_2Cl_2, 400 MHz, 298K)

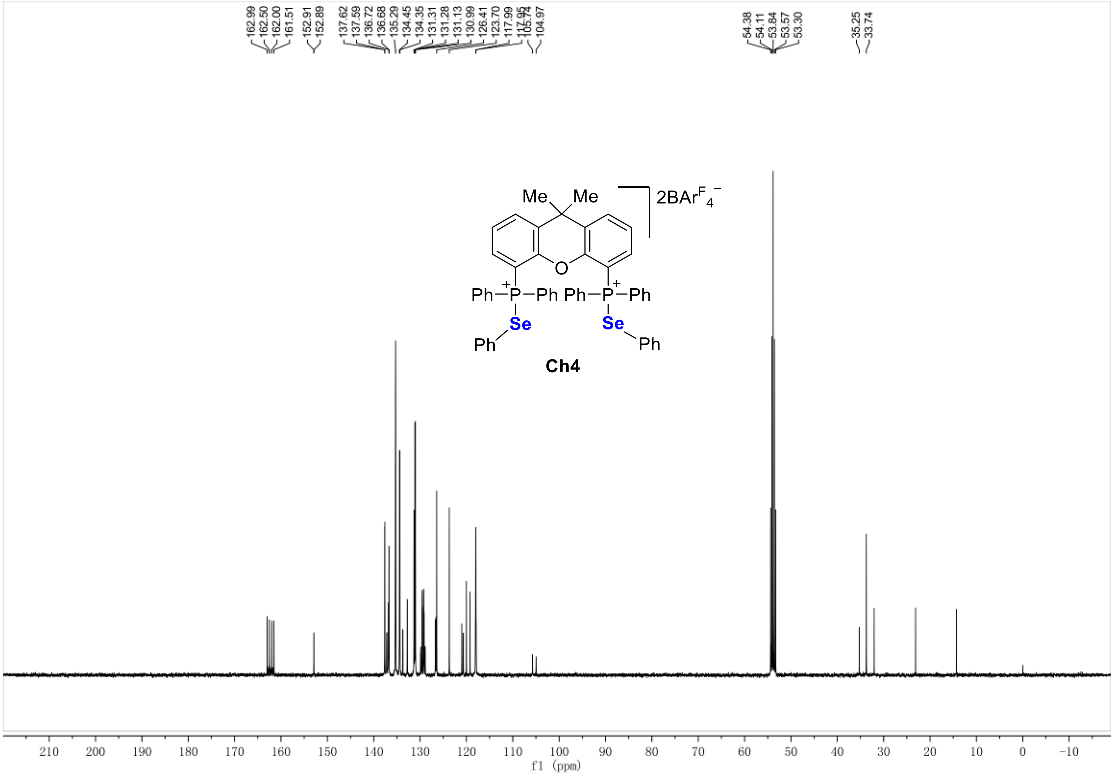

Supplementary Figure 43. ^{13}C NMR spectrum of compound Ch4 (CD_2Cl_2, 100 MHz, 298K)

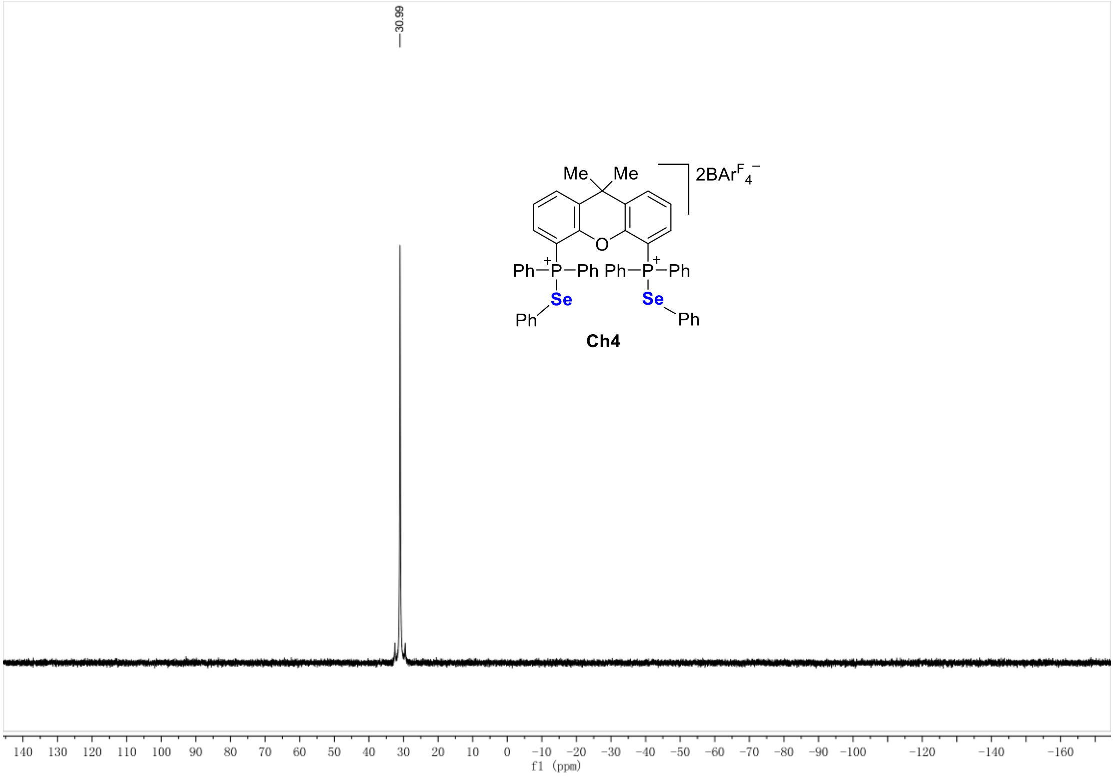

Supplementary Figure 44. ^{31}P NMR spectrum of compound Ch4 (CD_2Cl_2, 162 MHz, 298K)

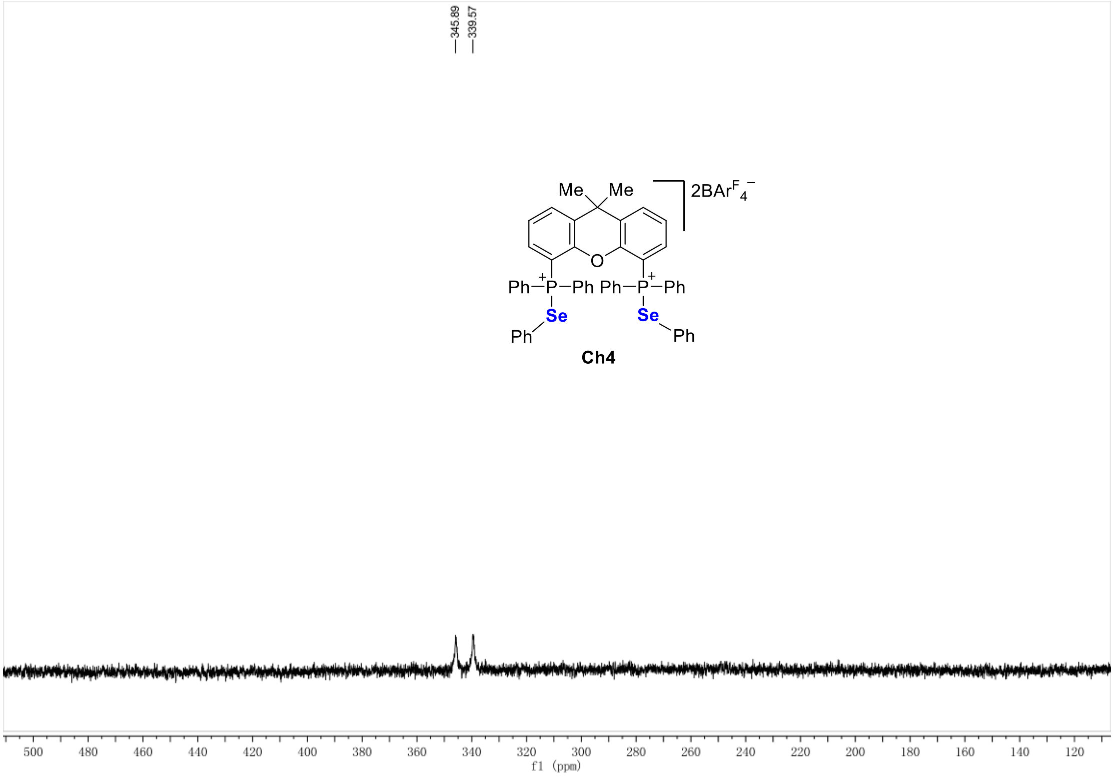

Supplementary Figure 45. ^{77}Se NMR spectrum of compound Ch4 (CD_2Cl_2, 76 MHz, 298K)

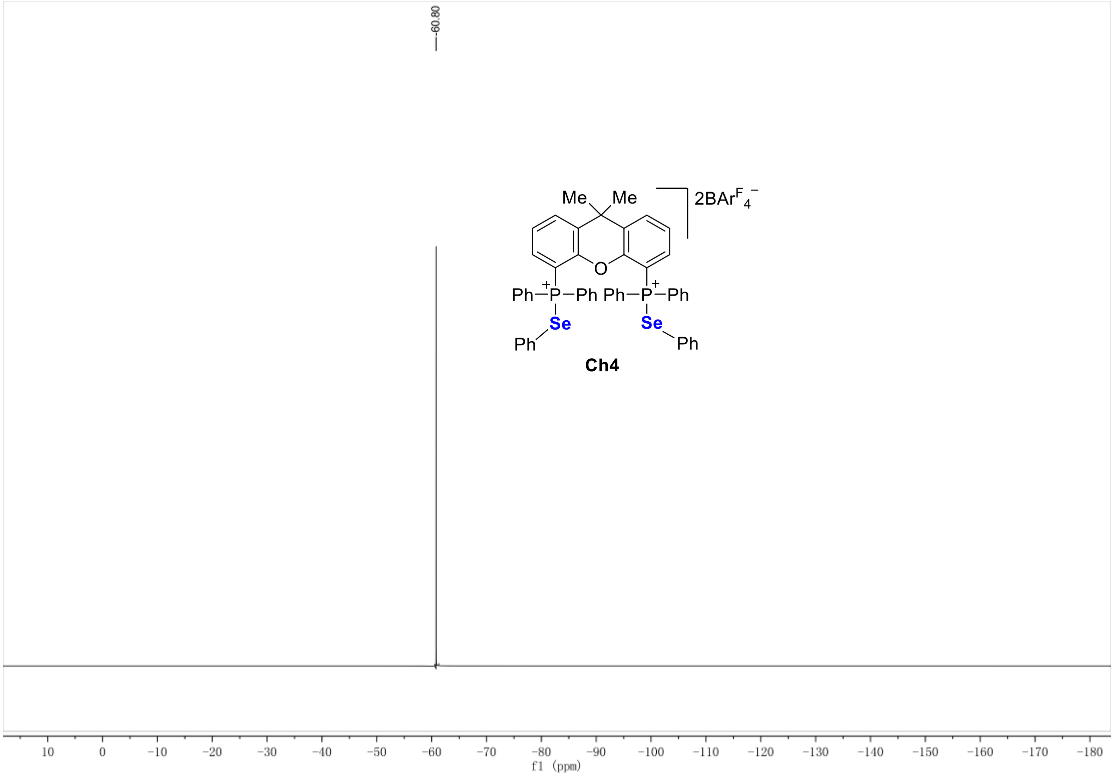

Supplementary Figure 46. ^{19}F NMR spectrum of compound Ch4 (CD_2Cl_2, 376 MHz, 298K)

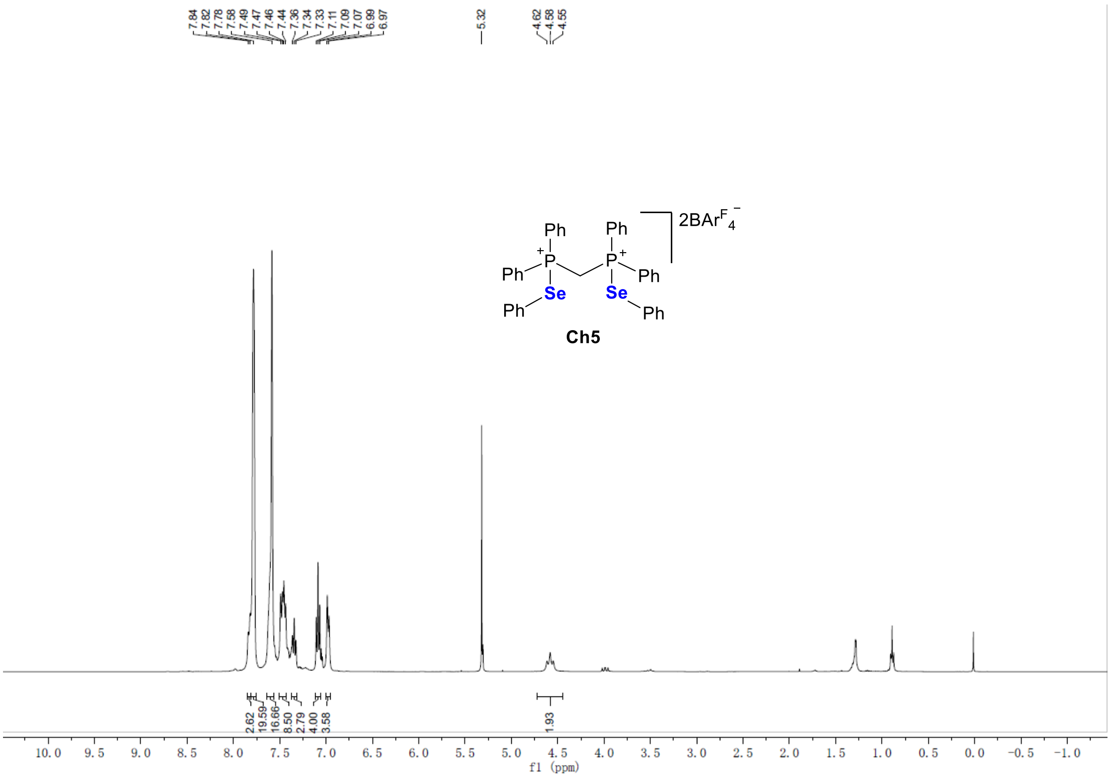

Supplementary Figure 47. ^{1}H NMR spectrum of compound Ch5 (CD_2Cl_2, 400 MHz, 298K)

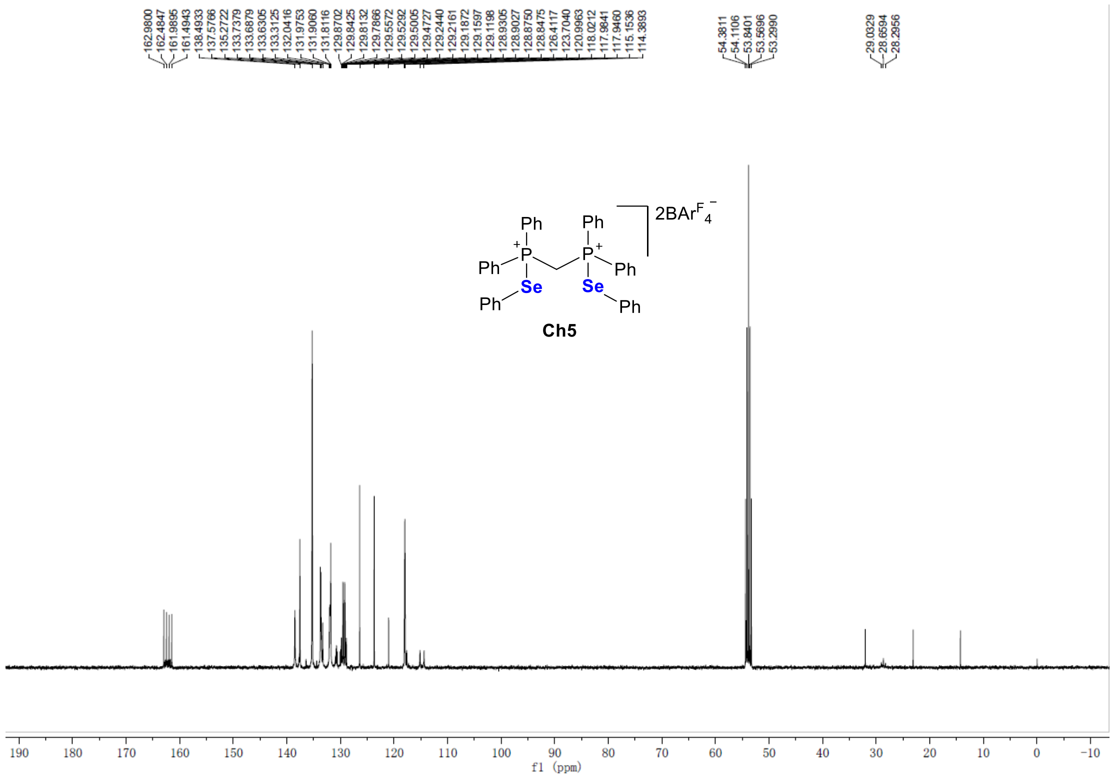

Supplementary Figure 48. ^{13}C NMR spectrum of compound Ch5 (CD_2Cl_2, 100 MHz, 298K)

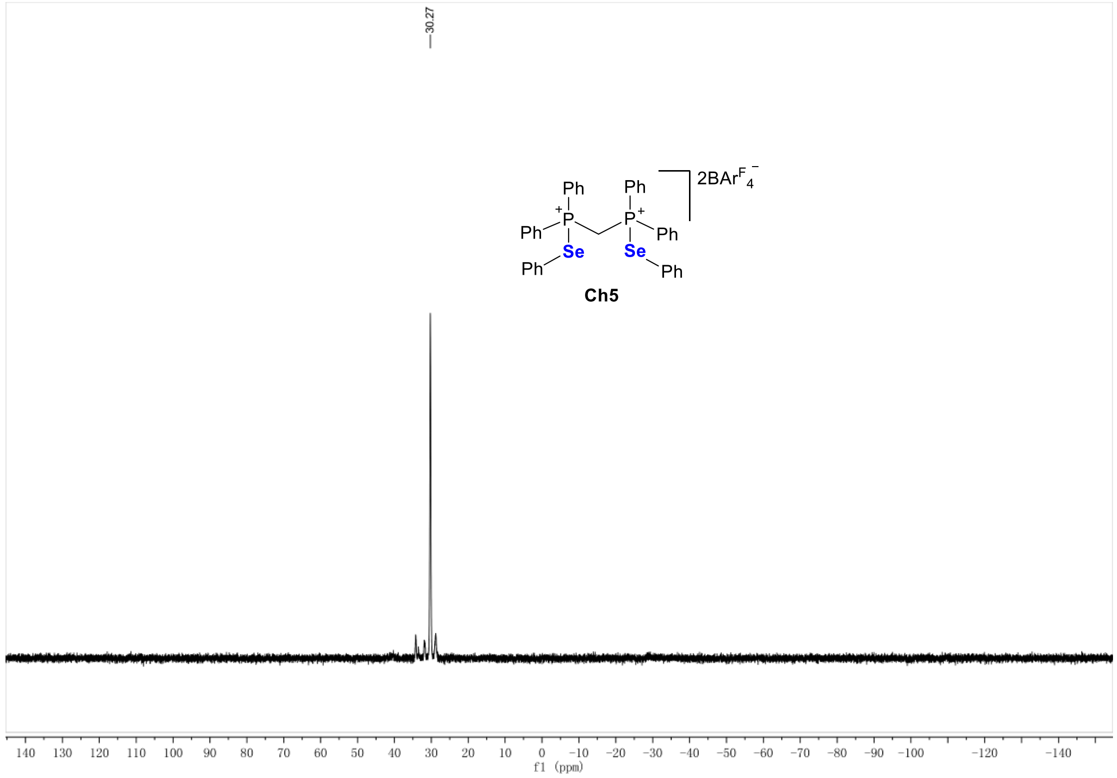

Supplementary Figure 49. ^{31}P NMR spectrum of compound Ch5 (CD_2Cl_2, 162 MHz, 298K)

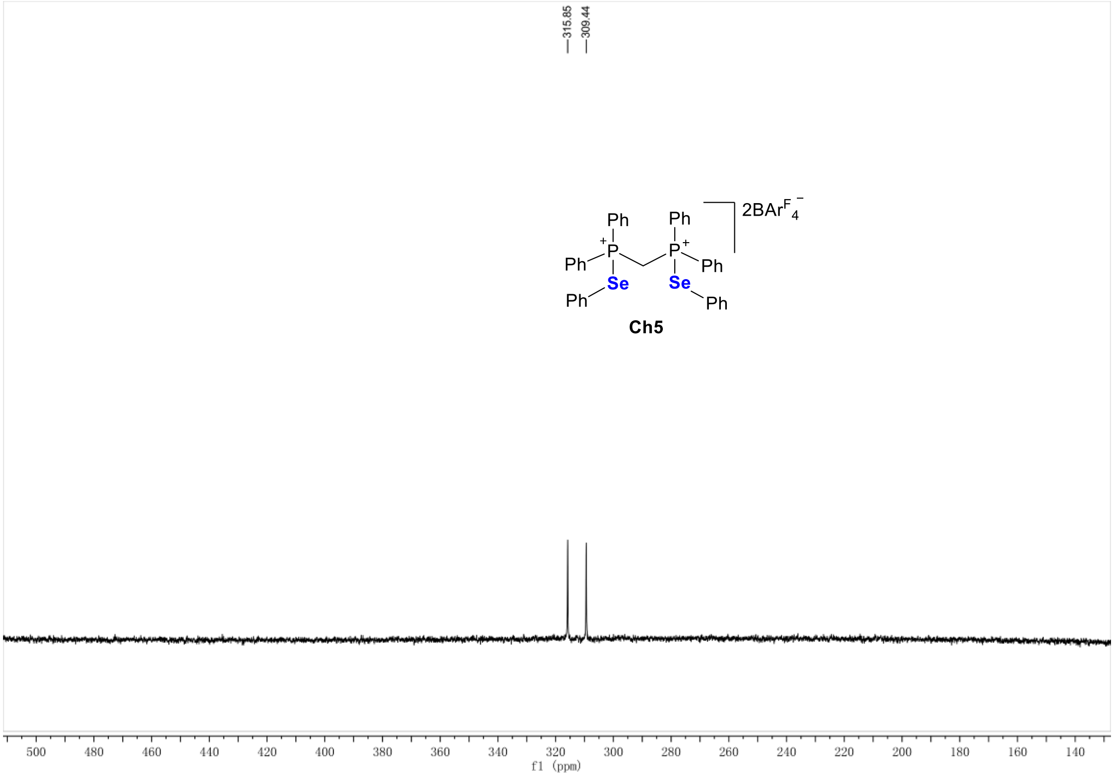

Supplementary Figure 50. ^{77}**Se NMR spectrum of compound Ch5 (**CD_2Cl_2**, 76 MHz, 298K)**

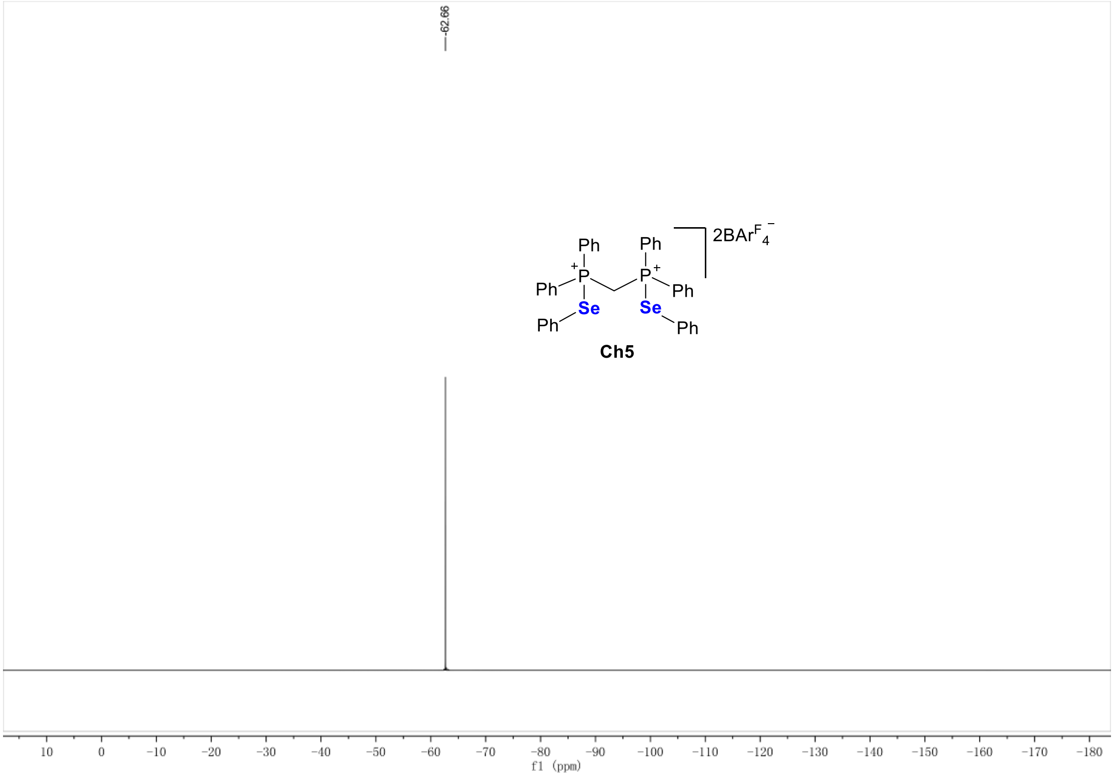

Supplementary Figure 51. ^{19}**F NMR spectrum of compound Ch5 (**CD_2Cl_2**, 376 MHz, 298K)**

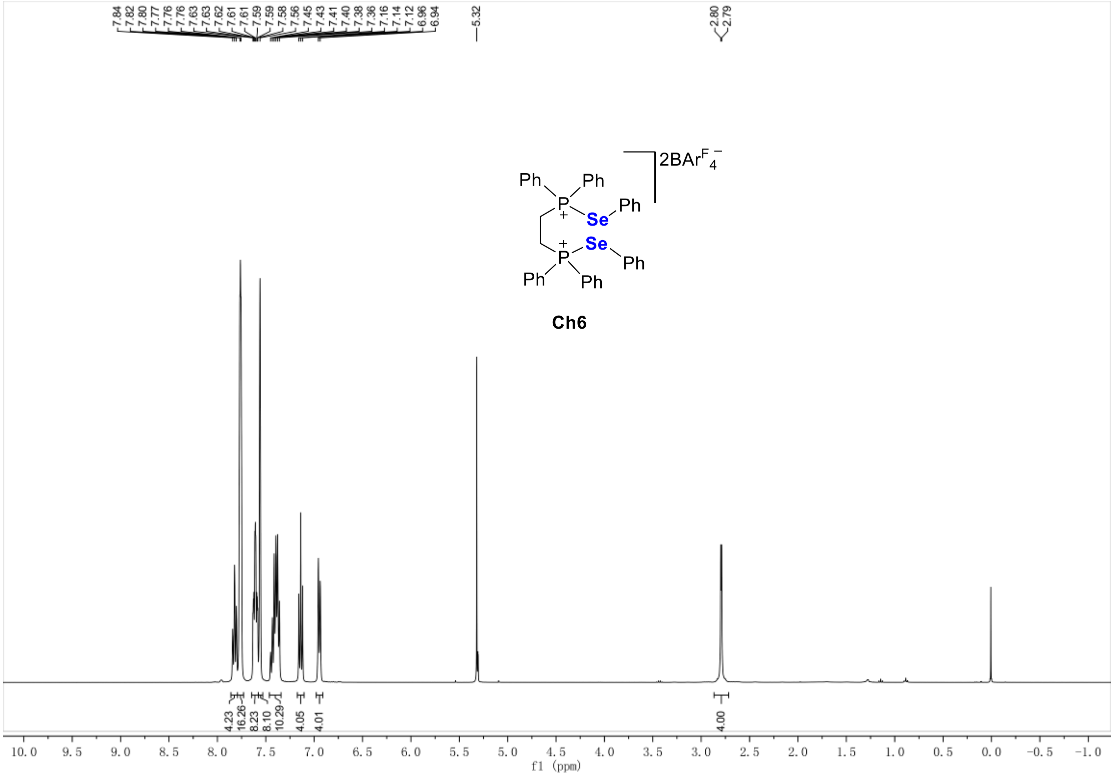

Supplementary Figure 52. ^{1}H NMR spectrum of compound Ch6 (CD_2Cl_2, 400 MHz, 298K)

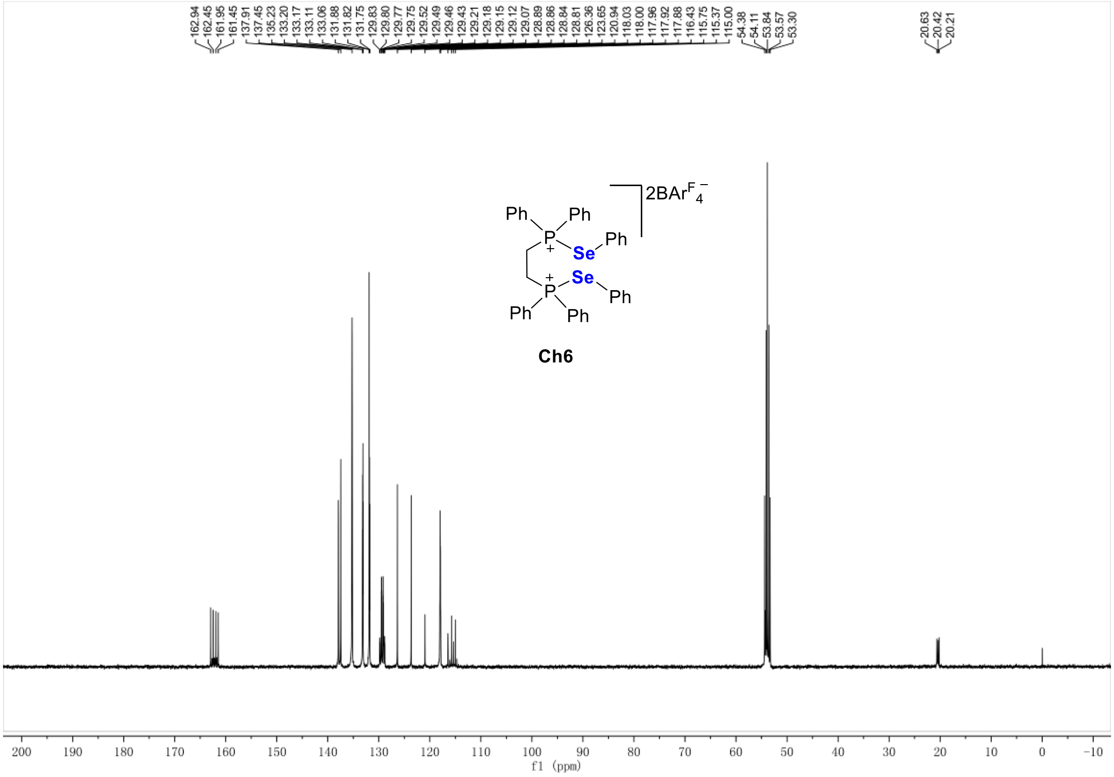

Supplementary Figure 53. ^{13}C NMR spectrum of compound Ch6 (CD_2Cl_2, 100 MHz, 298K)

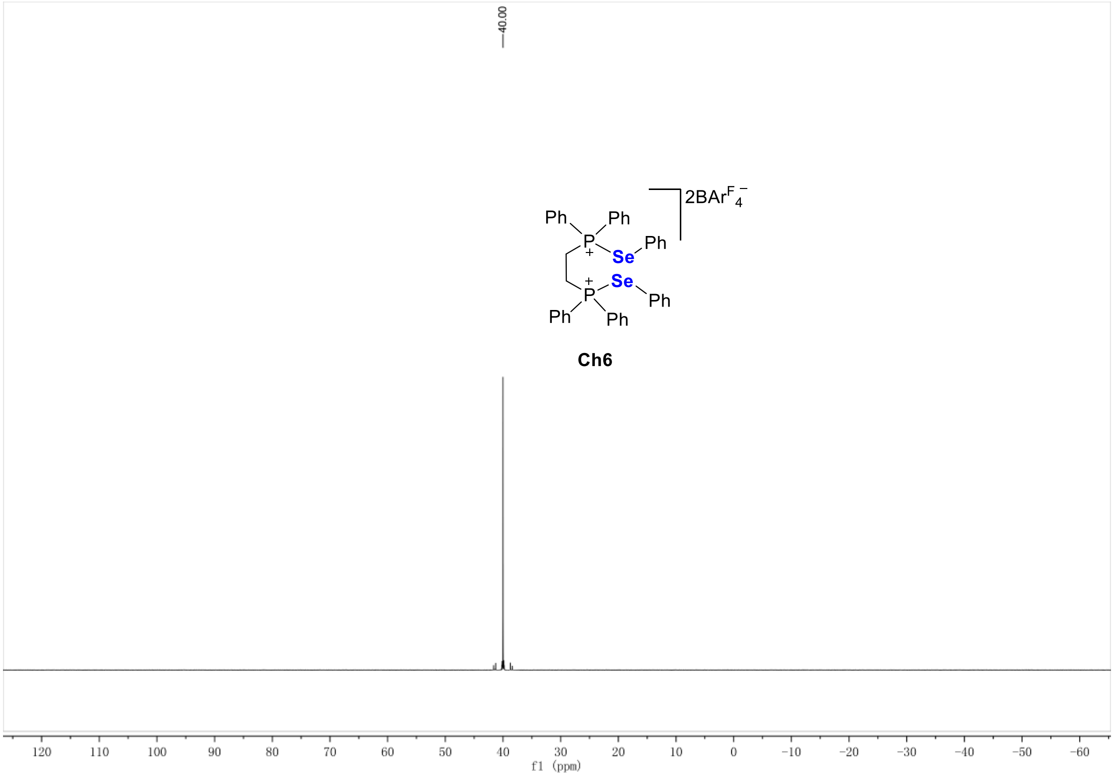

Supplementary Figure 54. ^{31}P NMR spectrum of compound Ch6 (CD_2Cl_2, 162 MHz, 298K)

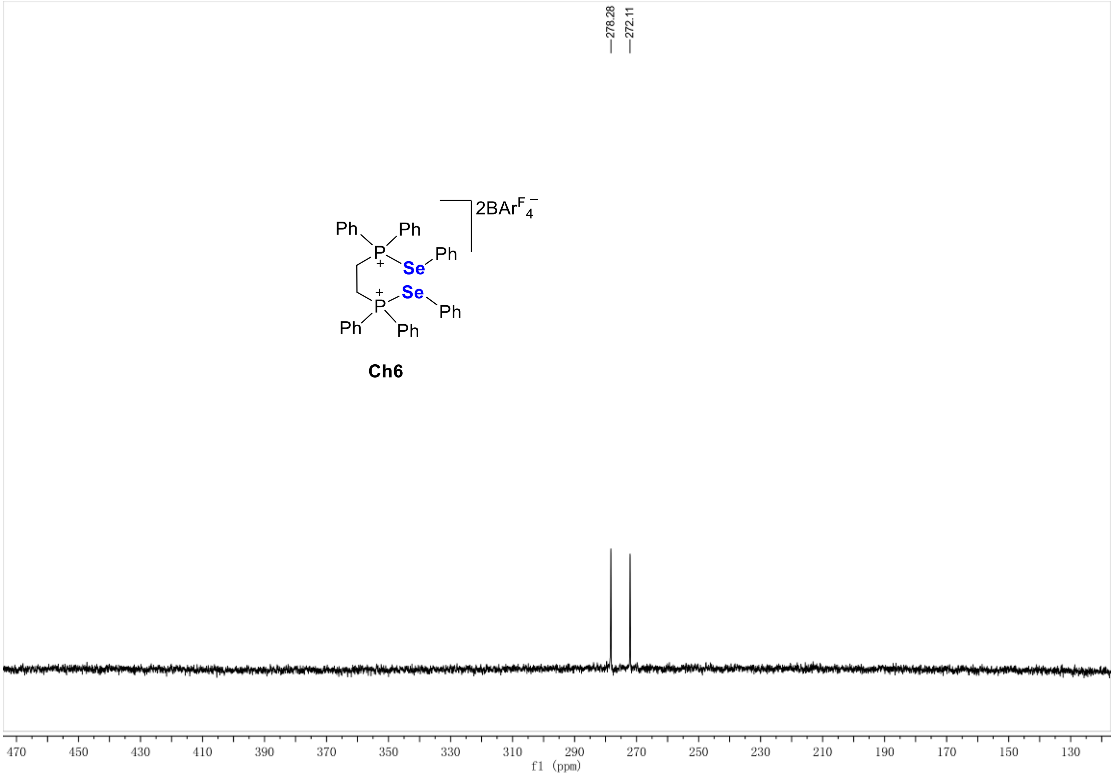

Supplementary Figure 55. ^{77}Se NMR spectrum of compound Ch6 (CD_2Cl_2, 76 MHz, 298K)

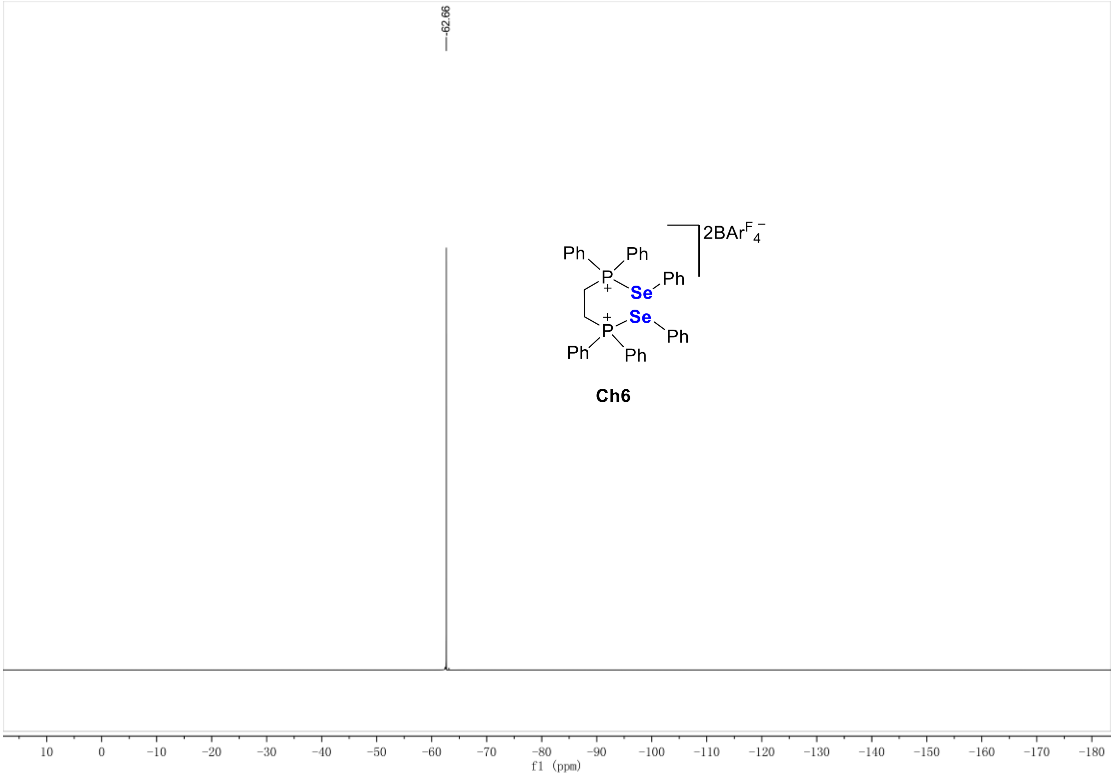

Supplementary Figure 56. ^{19}F NMR spectrum of compound Ch6 (CD_2Cl_2, 376 MHz, 298K)

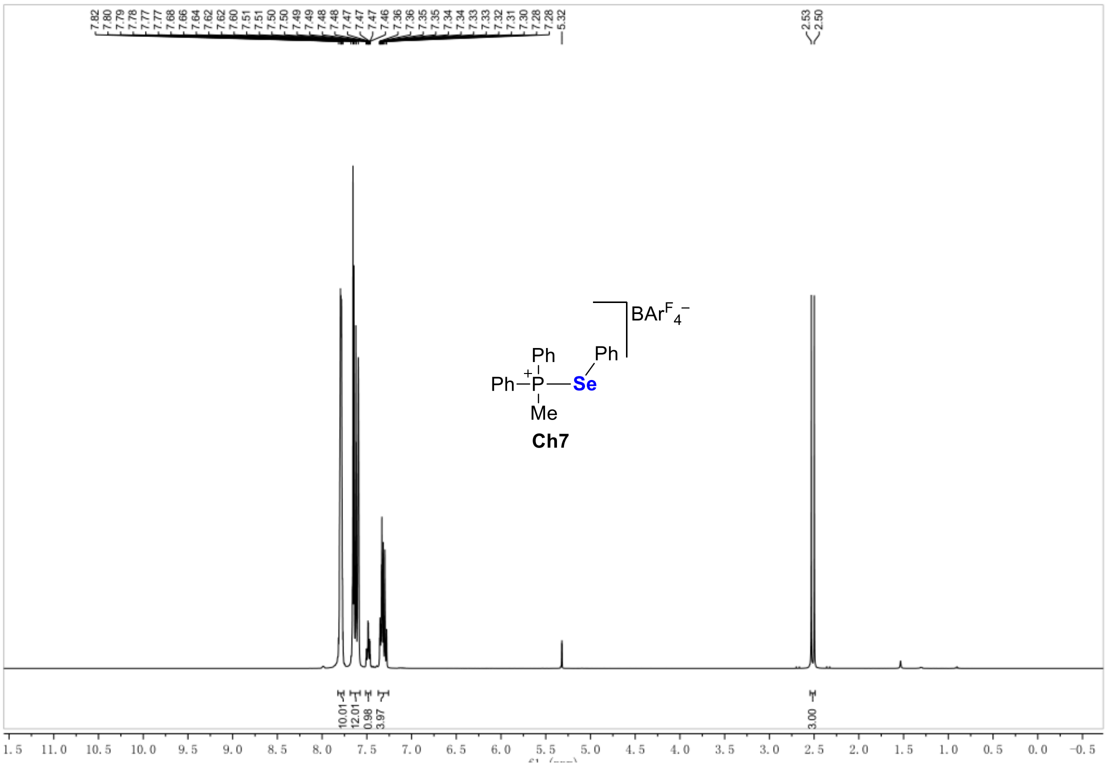

Supplementary Figure 57. ^{1}H NMR spectrum of compound Ch7 ($CDCl_3$, 400 MHz, 298K)

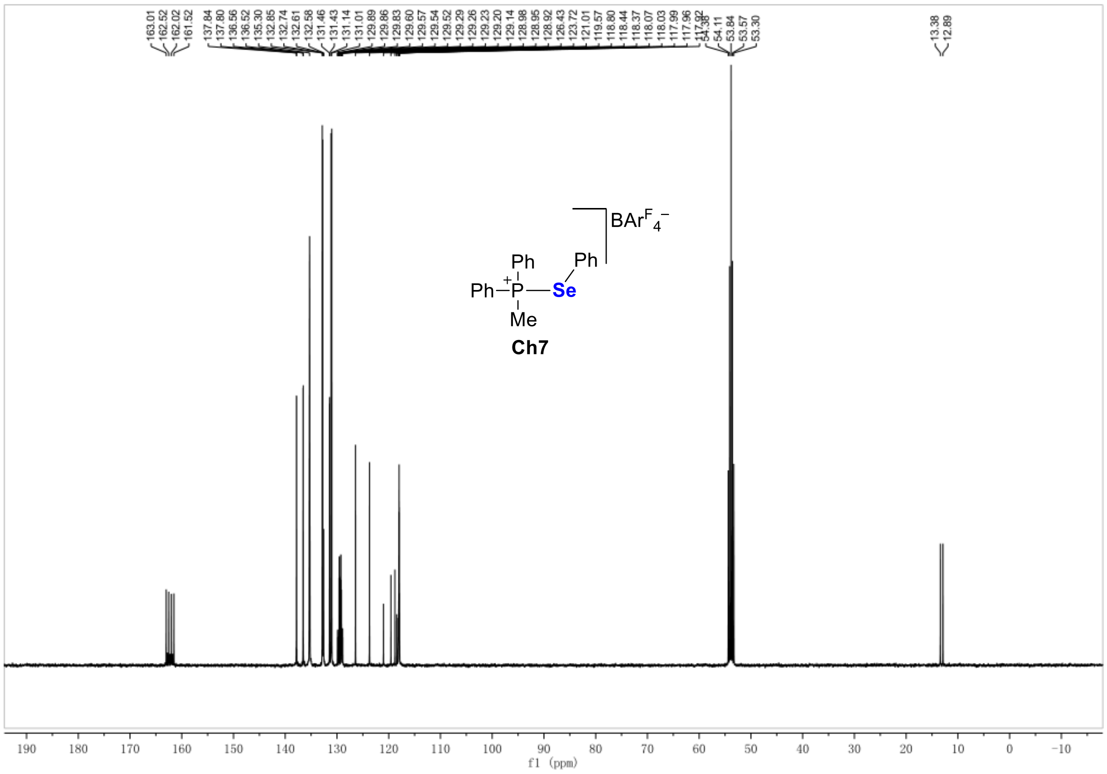

Supplementary Figure 58. ^{13}**C NMR spectrum of compound Ch7 (CDCl$_3$, 100 MHz, 298K)**

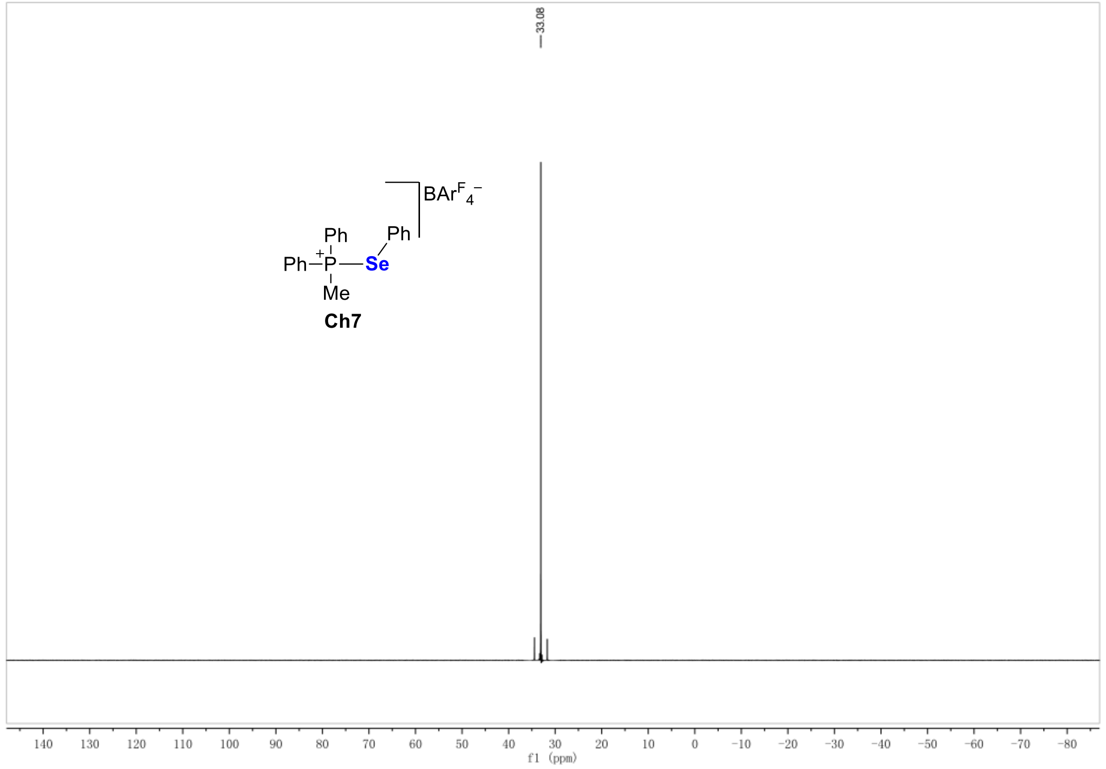

Supplementary Figure 59. ^{31}**P NMR spectrum of compound Ch7 (CD$_2$Cl$_2$, 162 MHz, 298K)**

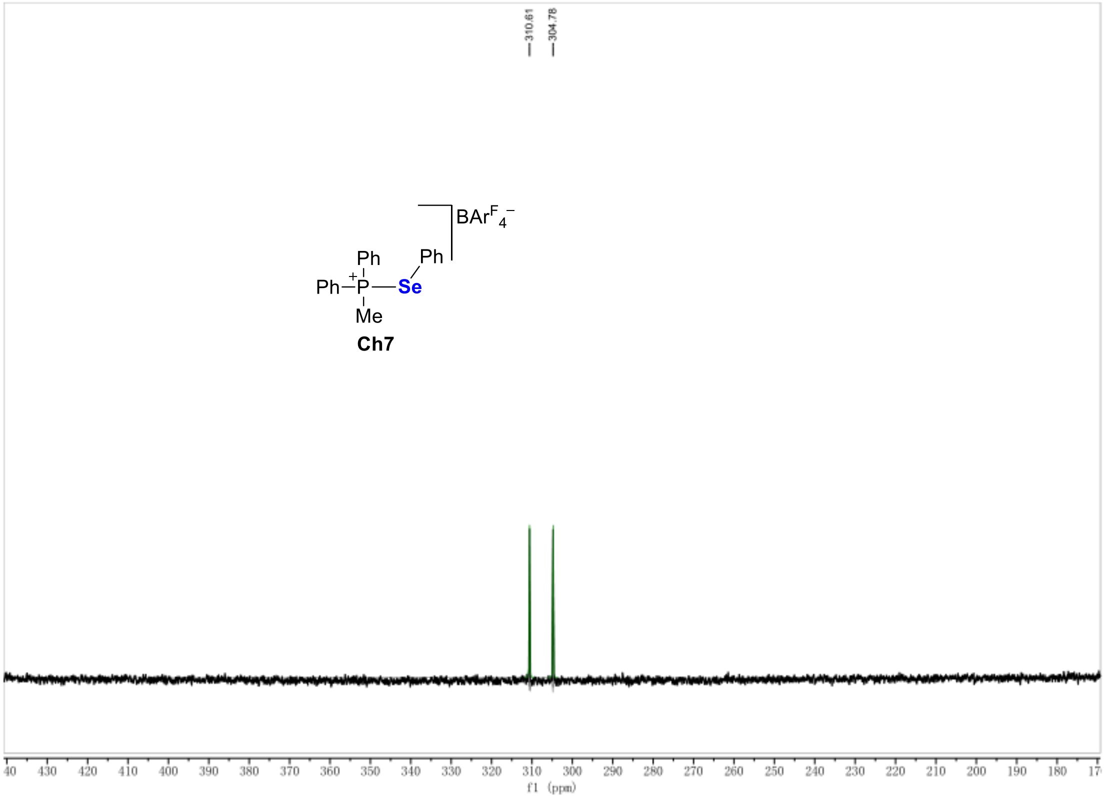

Supplementary Figure 60. ^{77}**Se NMR spectrum of compound Ch7 (**CD_2Cl_2**, 76 MHz, 298K)**

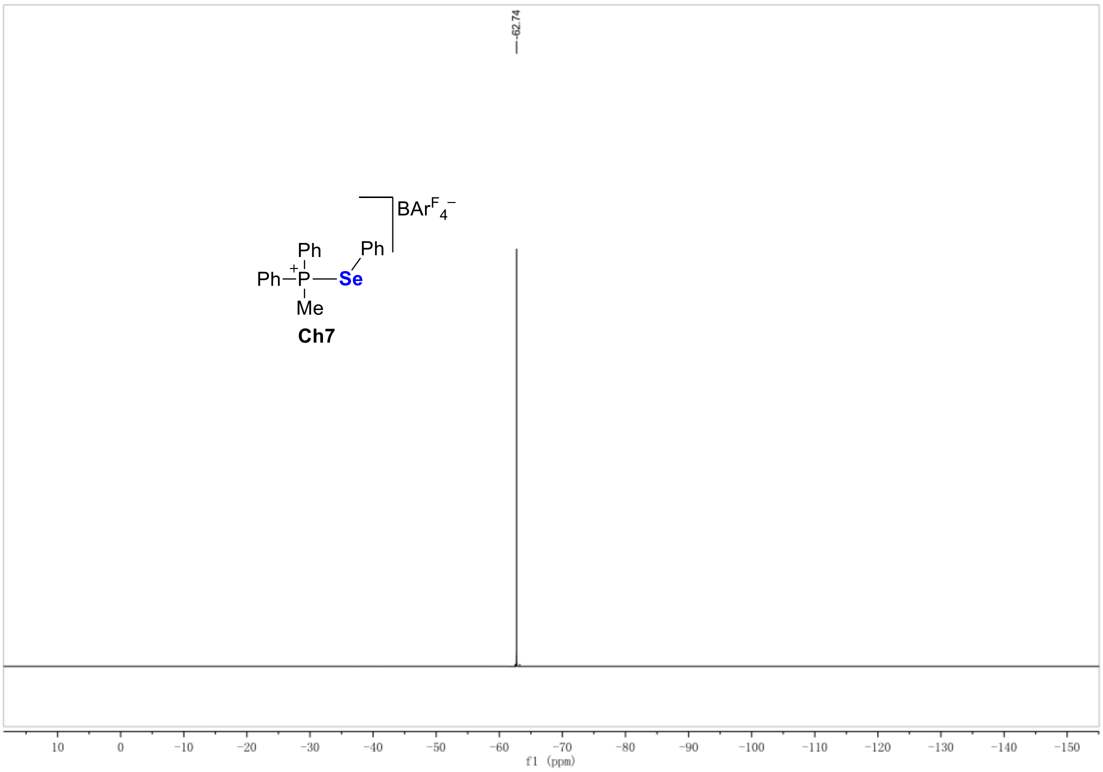

Supplementary Figure 61. ^{19}**F NMR spectrum of compound Ch7 (**CD_2Cl_2**, 376 MHz, 298K)**

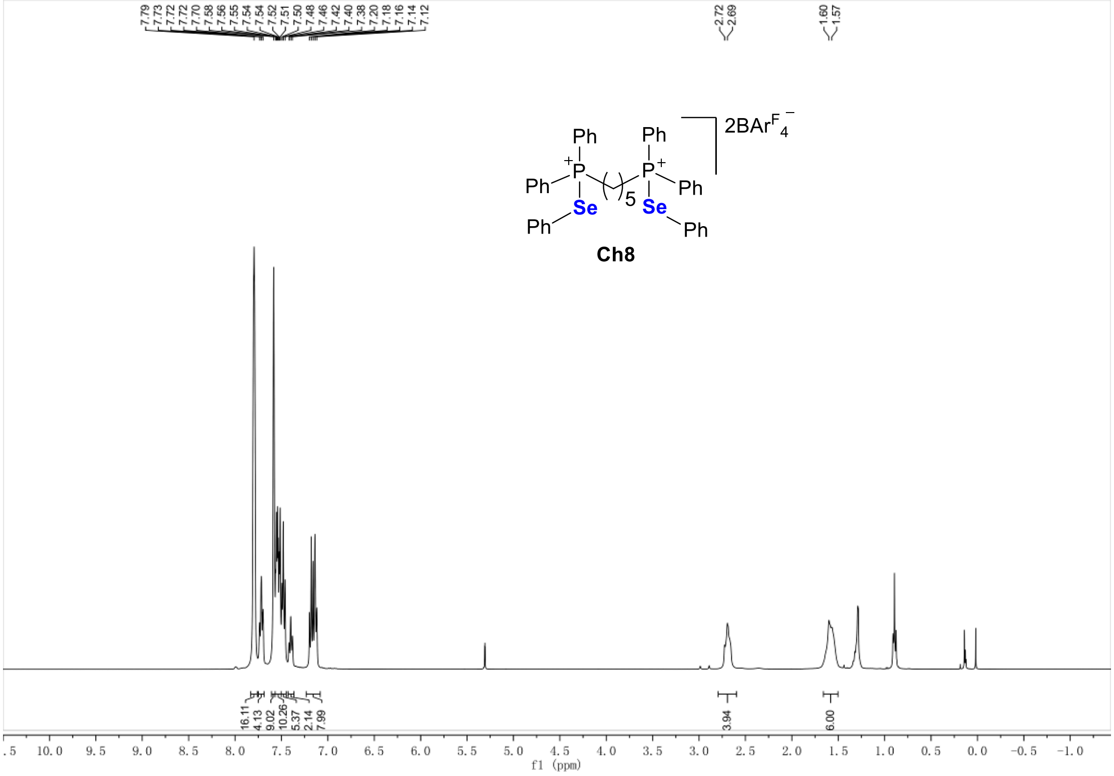

Supplementary Figure 62. ^{1}**H NMR spectrum of compound Ch8 (CD_2Cl_2, 400 MHz, 298K)**

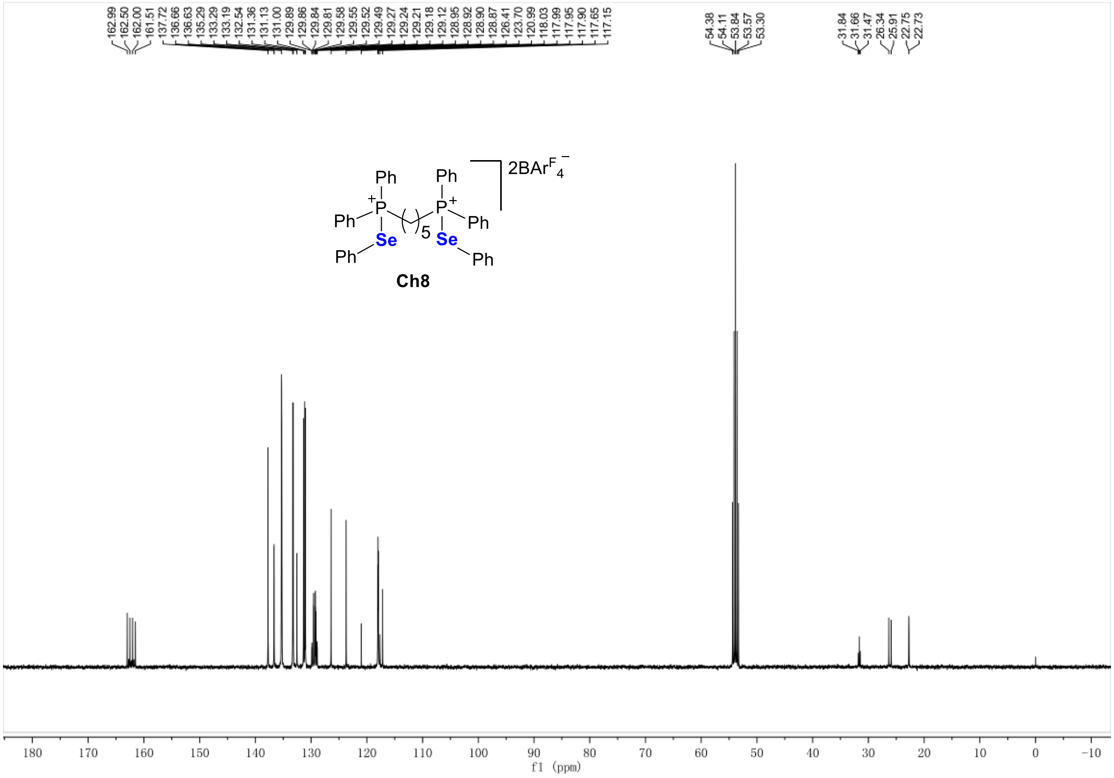

Supplementary Figure 63. ^{13}**C NMR spectrum of compound Ch8 (CD_2Cl_2, 100 MHz, 298K)**

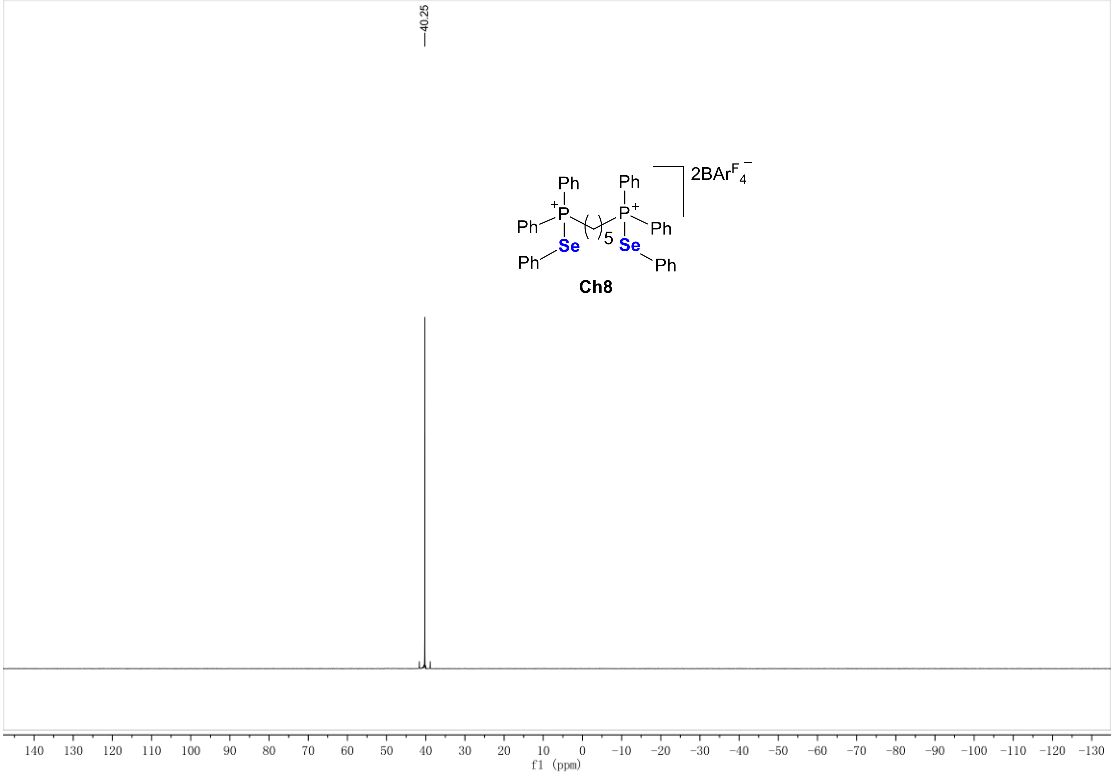

Supplementary Figure 64. ^{31}**P NMR spectrum of compound Ch8 (**CD_2Cl_2**, 162MHz, 298K)**

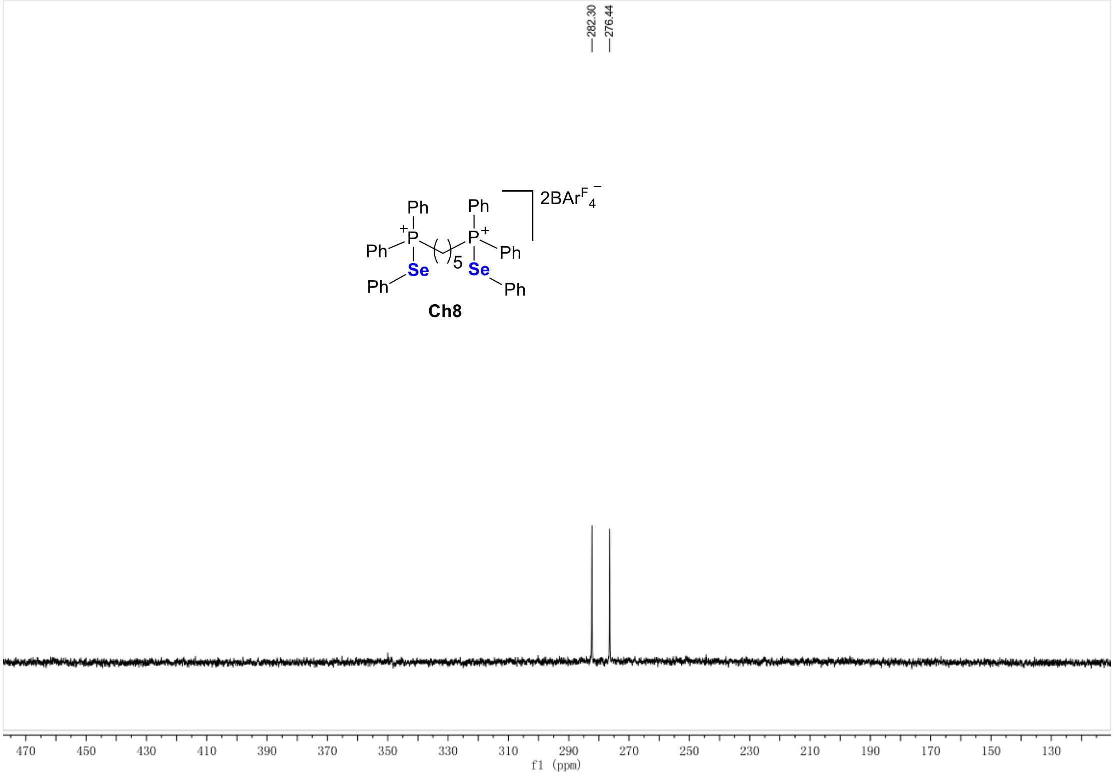

Supplementary Figure 65. ^{77}**Se NMR spectrum of compound Ch8 (**CD_2Cl_2**, 76 MHz, 298K)**

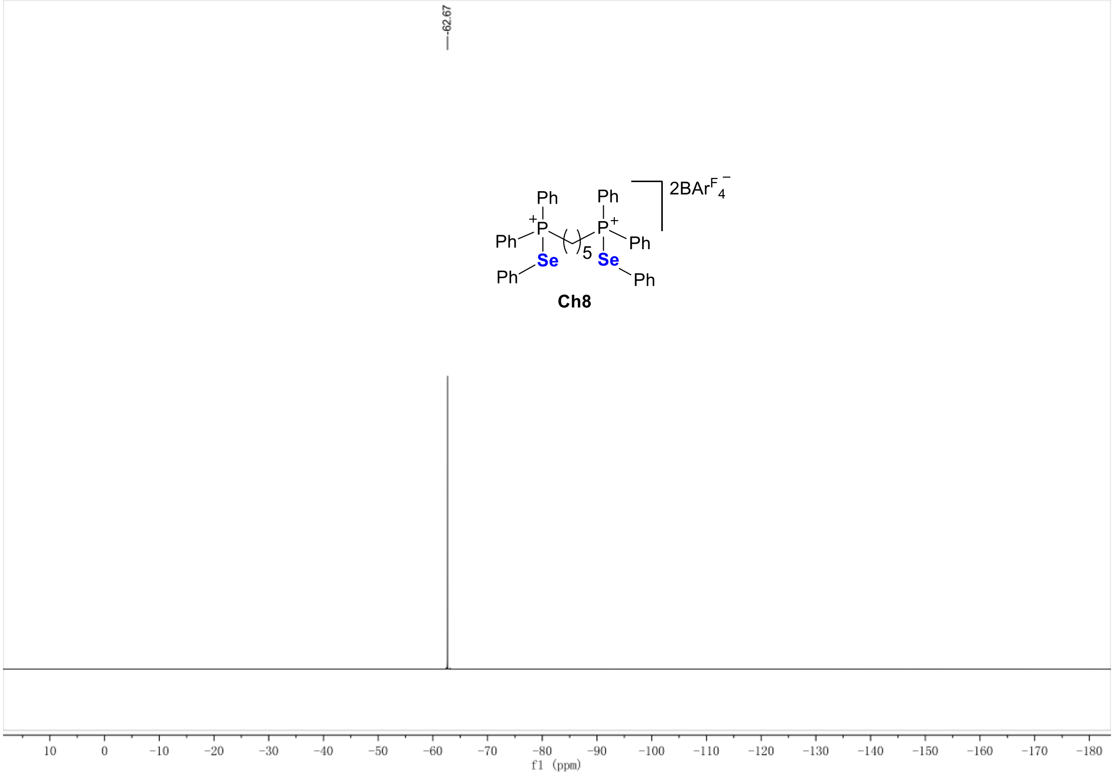

Supplementary Figure 66. ^{19}F NMR spectrum of compound Ch8 (CD_2Cl_2, 376 MHz, 298K)

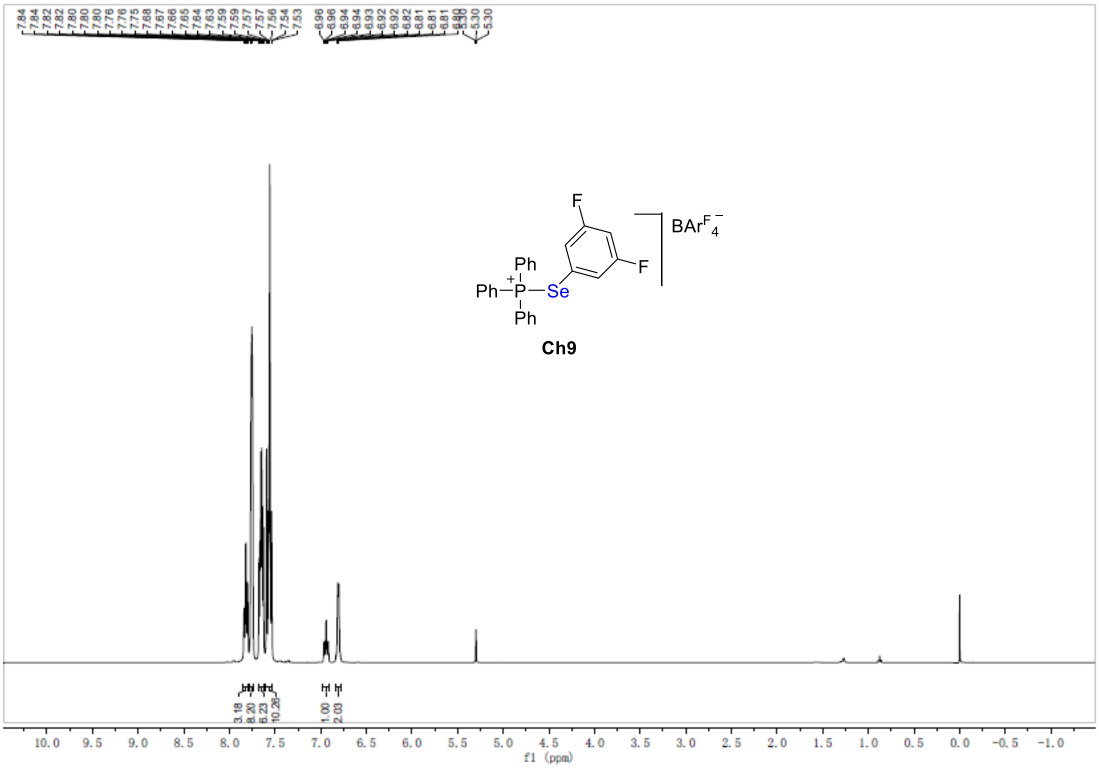

Supplementary Figure 67. ^{1}H NMR spectrum of compound Ch9 (CD_2Cl_2, 400 MHz, 298K)

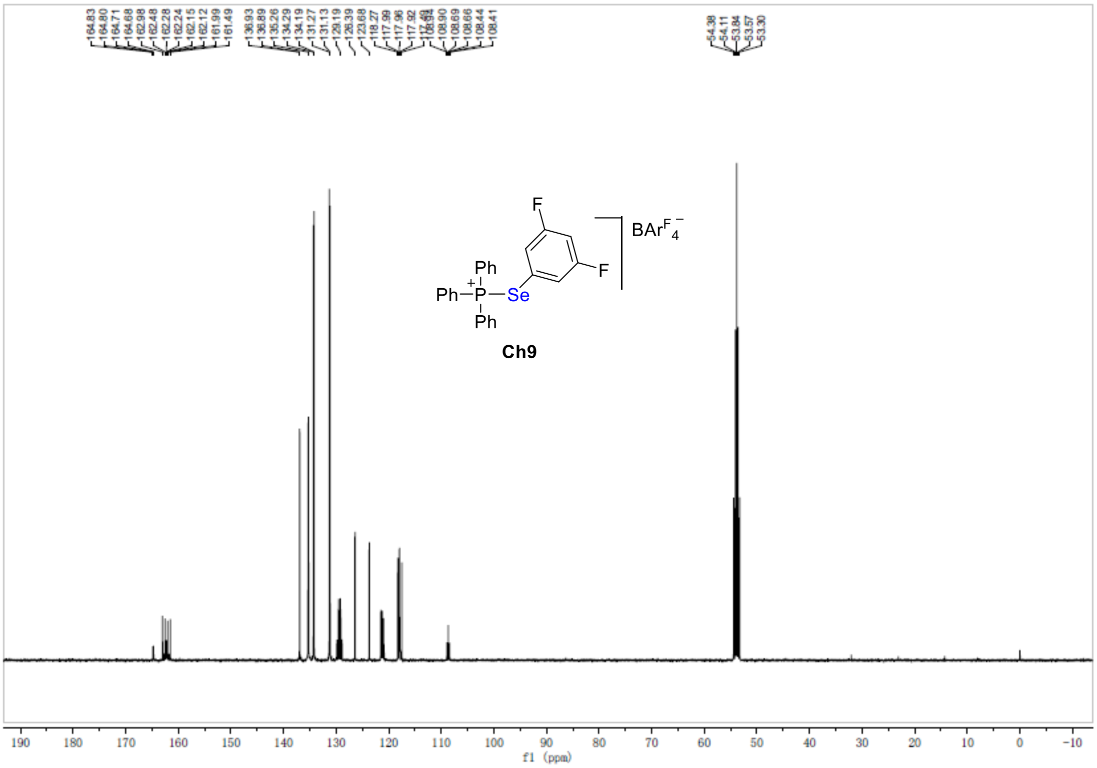

Supplementary Figure 68. ^{13}C NMR spectrum of compound Ch9 (CD_2Cl_2, 100 MHz, 298K)

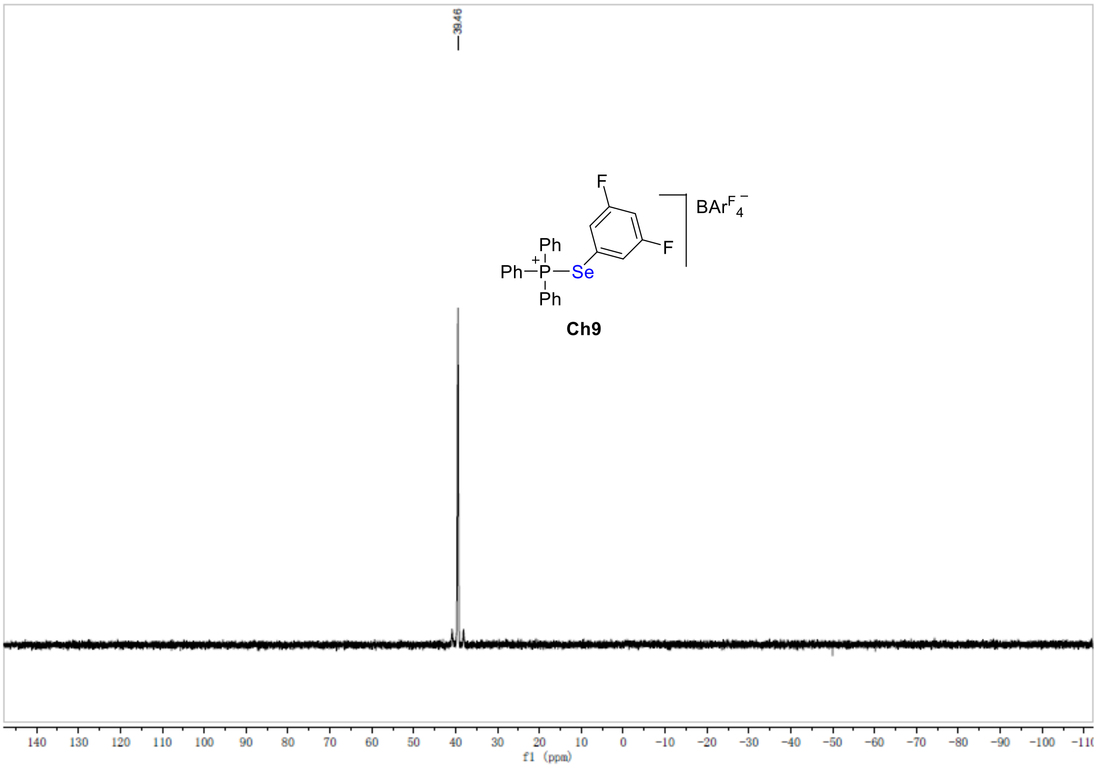

Supplementary Figure 69. ^{31}P NMR spectrum of compound Ch9 (CD_2Cl_2, 162MHz, 298K)

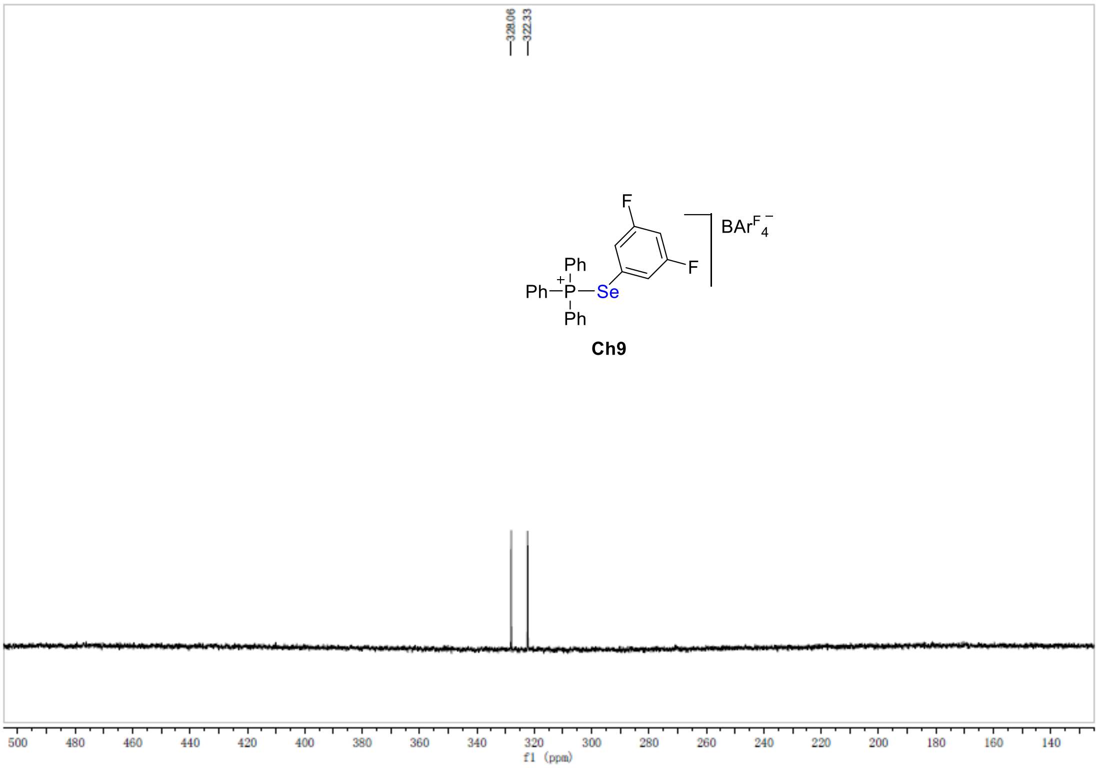

Supplementary Figure 70. ^{77}**Se NMR spectrum of compound Ch9 (**CD_2Cl_2**, 76 MHz, 298K)**

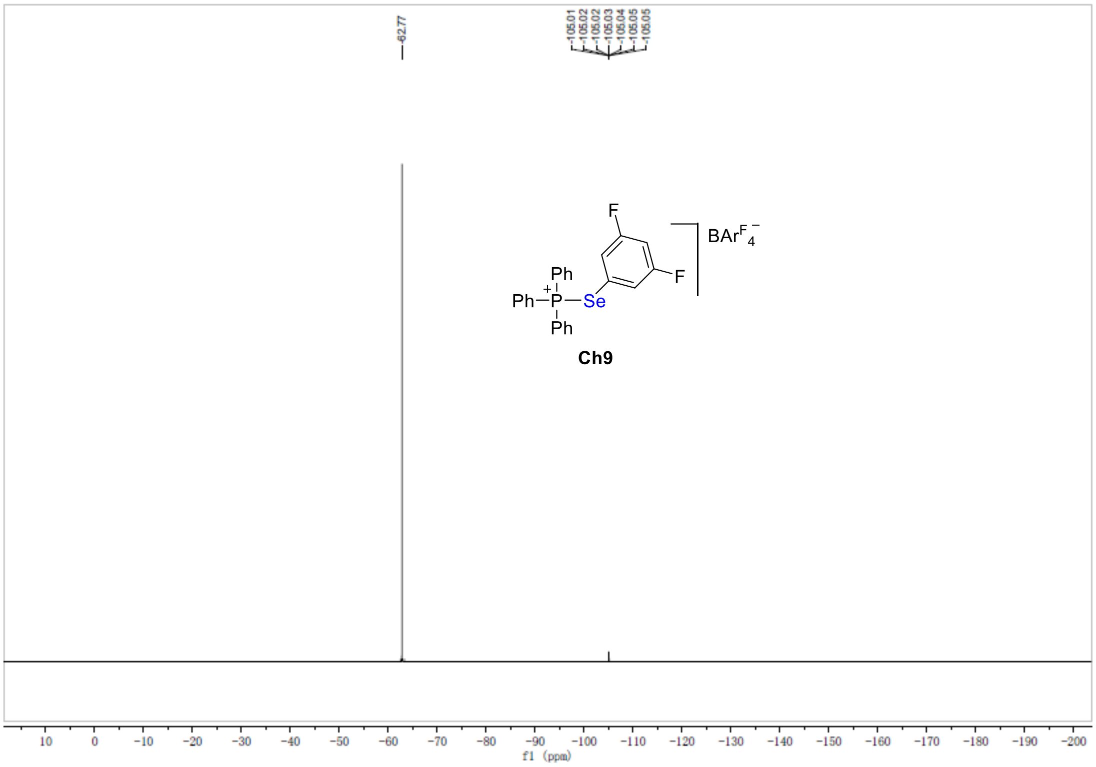

Supplementary Figure 71. ^{19}**F NMR spectrum of compound Ch9 (**CD_2Cl_2**, 376 MHz, 298K)**

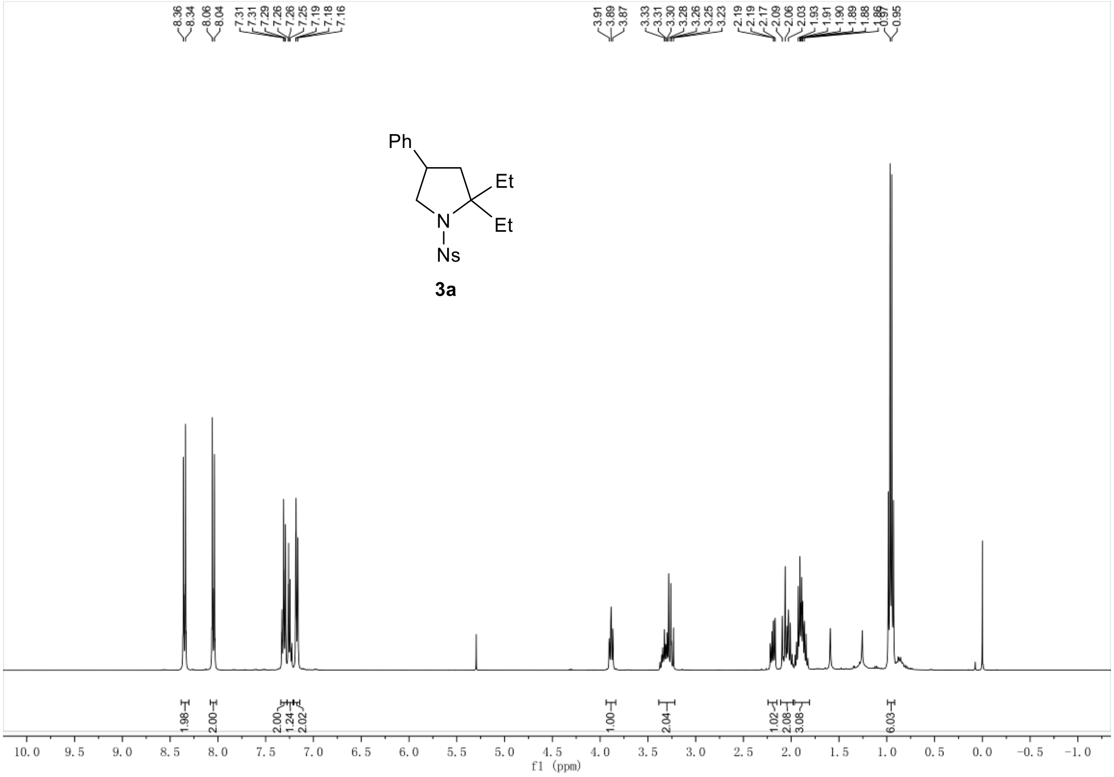

Supplementary Figure 72. ^{1}H NMR spectrum of compound 3a ($CDCl_3$, 400 MHz, 298K)

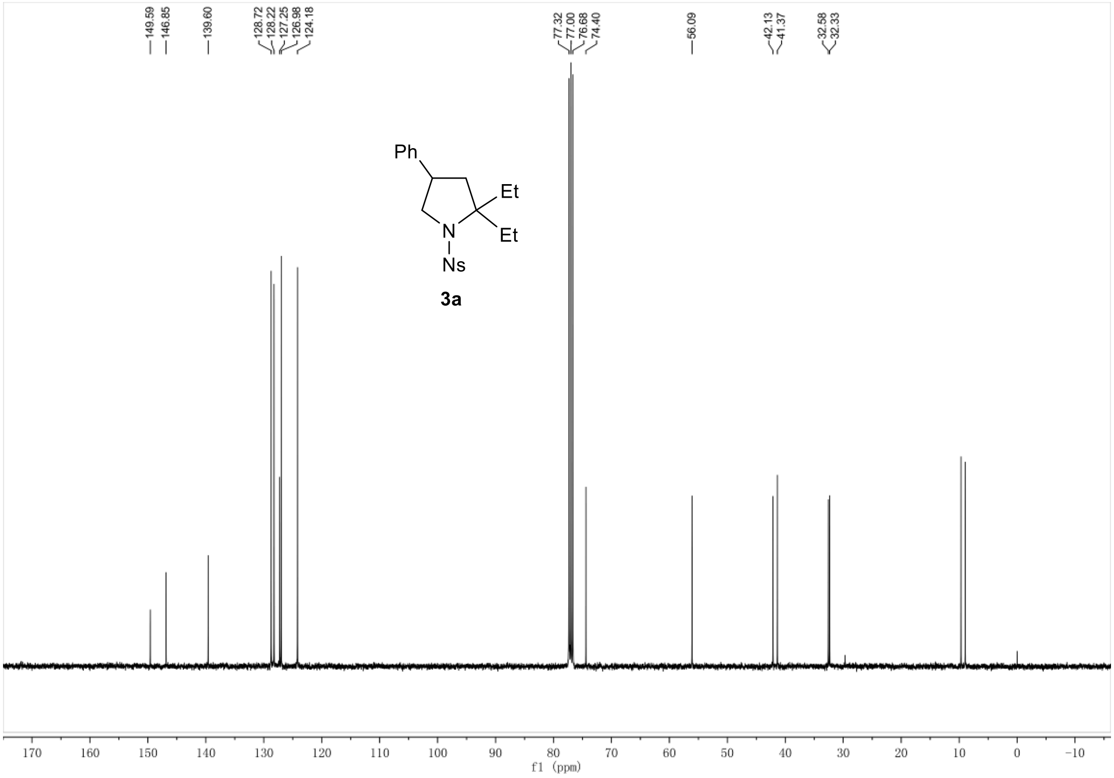

Supplementary Figure 73. ^{13}C NMR spectrum of compound 3a ($CDCl_3$, 100 MHz, 298K)

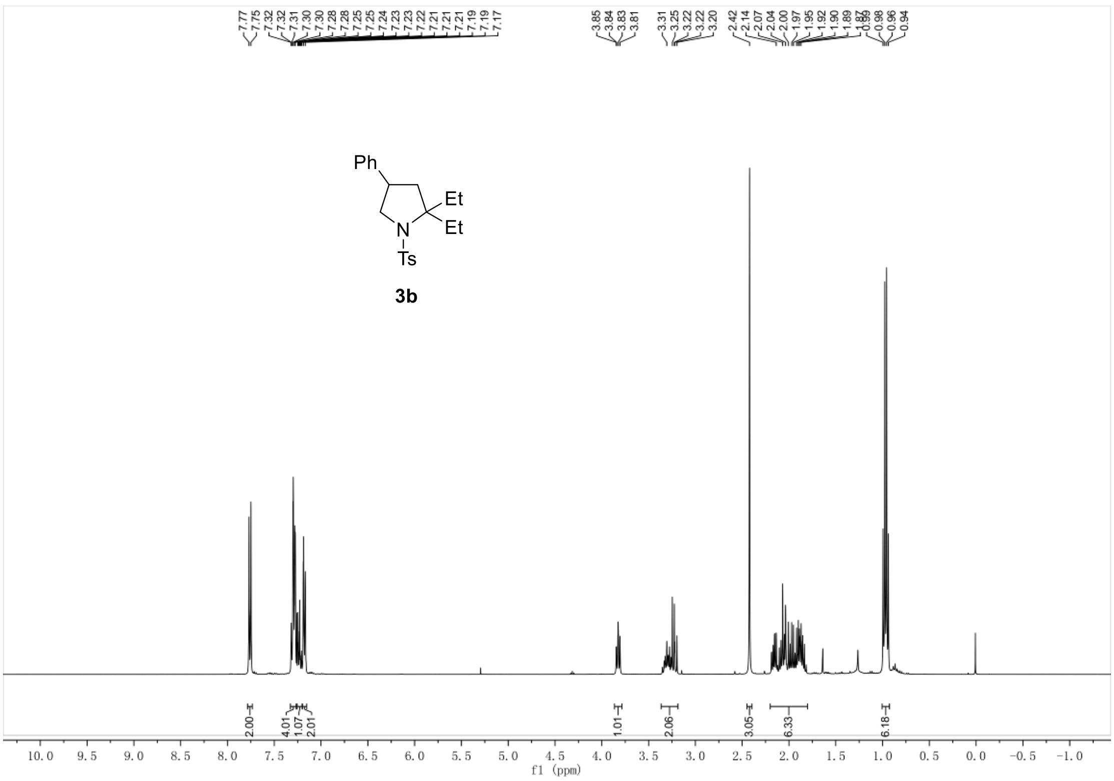

Supplementary Figure 74. ^{1}H NMR spectrum of compound 3b ($CDCl_3$, 400 MHz, 298K)

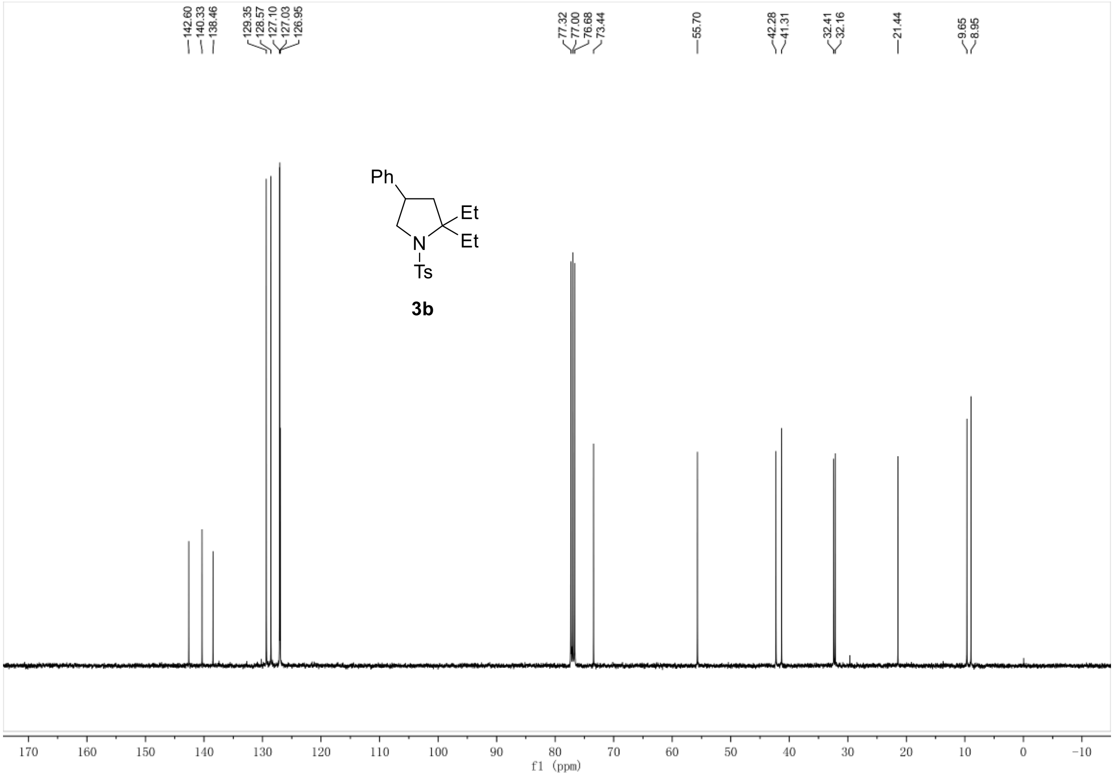

Supplementary Figure 75. ^{13}C NMR spectrum of compound 3b ($CDCl_3$, 100 MHz, 298K)

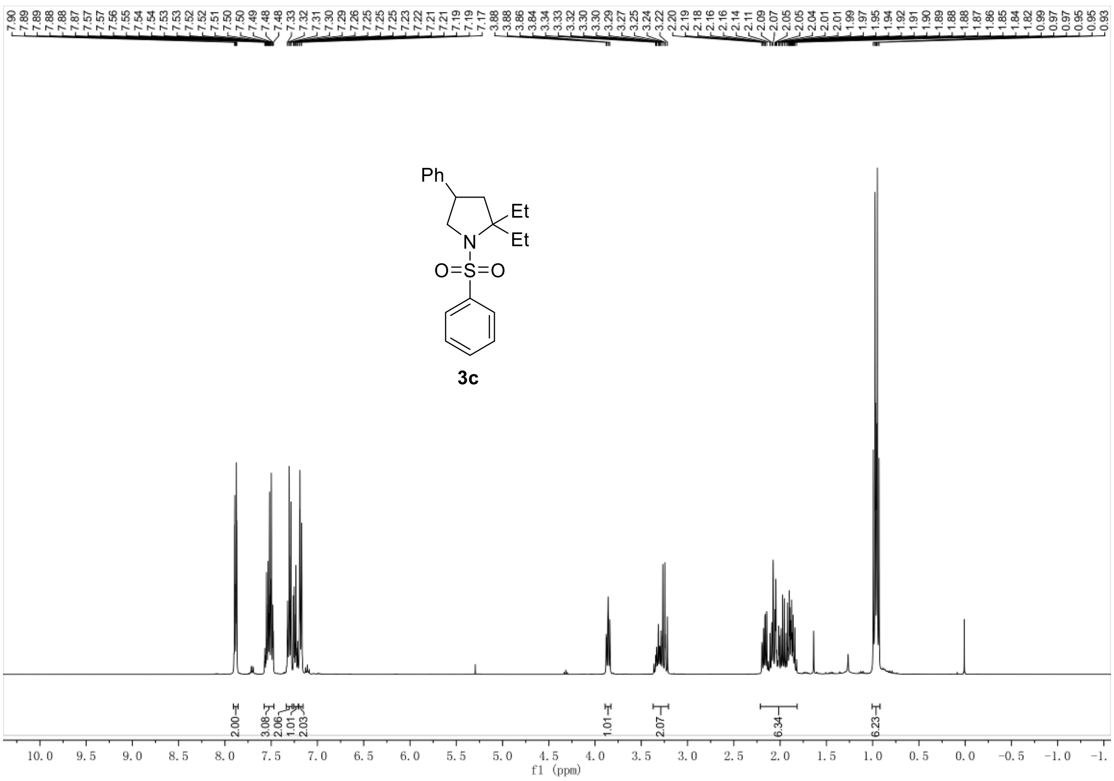

Supplementary Figure 76. ^{1}H NMR spectrum of compound 3c ($CDCl_3$, 400 MHz, 298K)

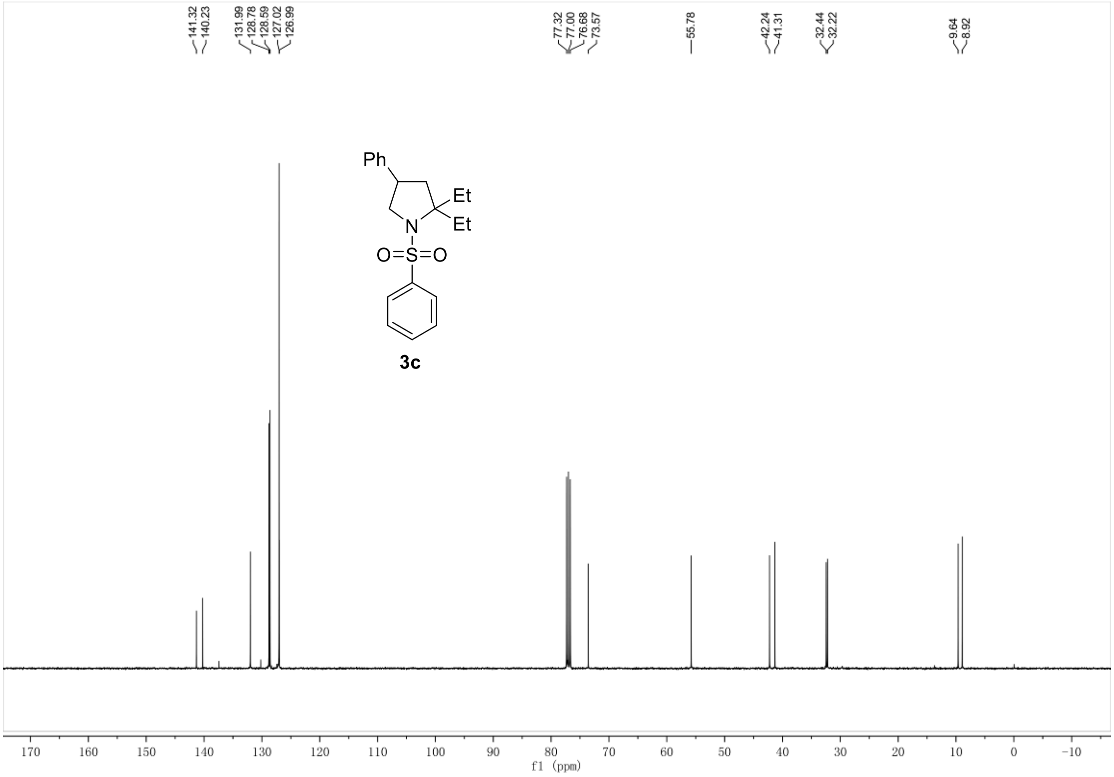

Supplementary Figure 77. ^{13}C NMR spectrum of compound 3c ($CDCl_3$, 100 MHz, 298K)

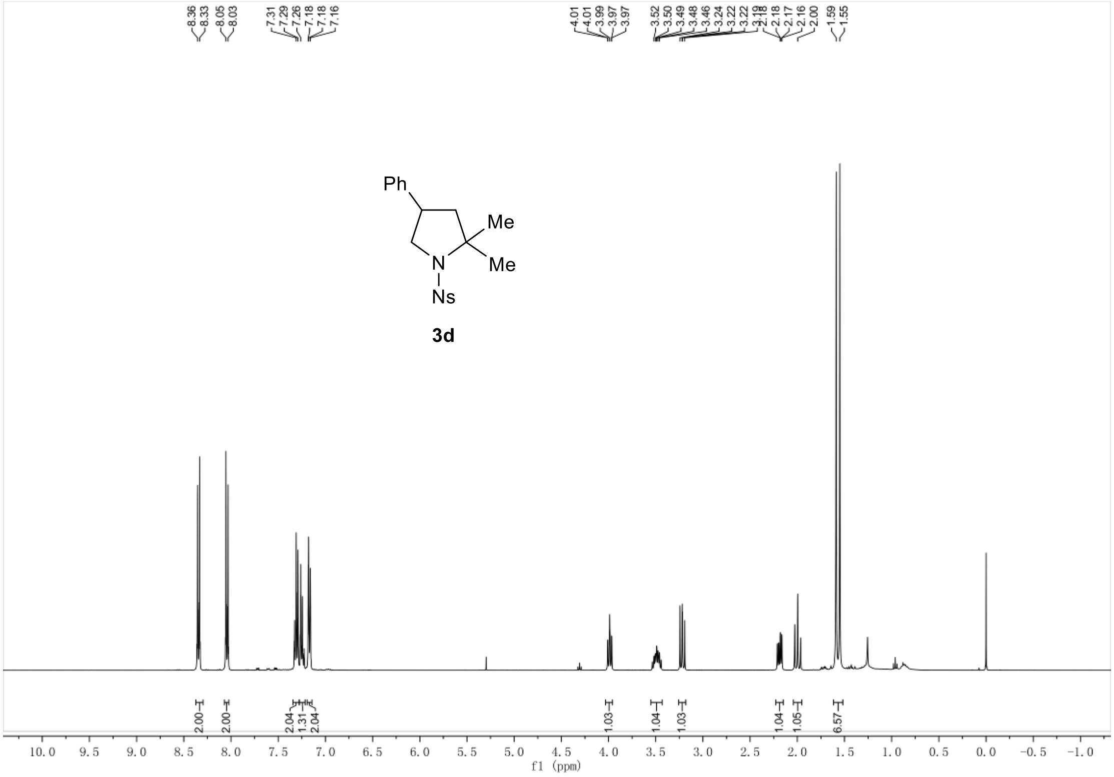

Supplementary Figure 78. ^{1}H NMR spectrum of compound 3d ($CDCl_3$, 400 MHz, 298K)

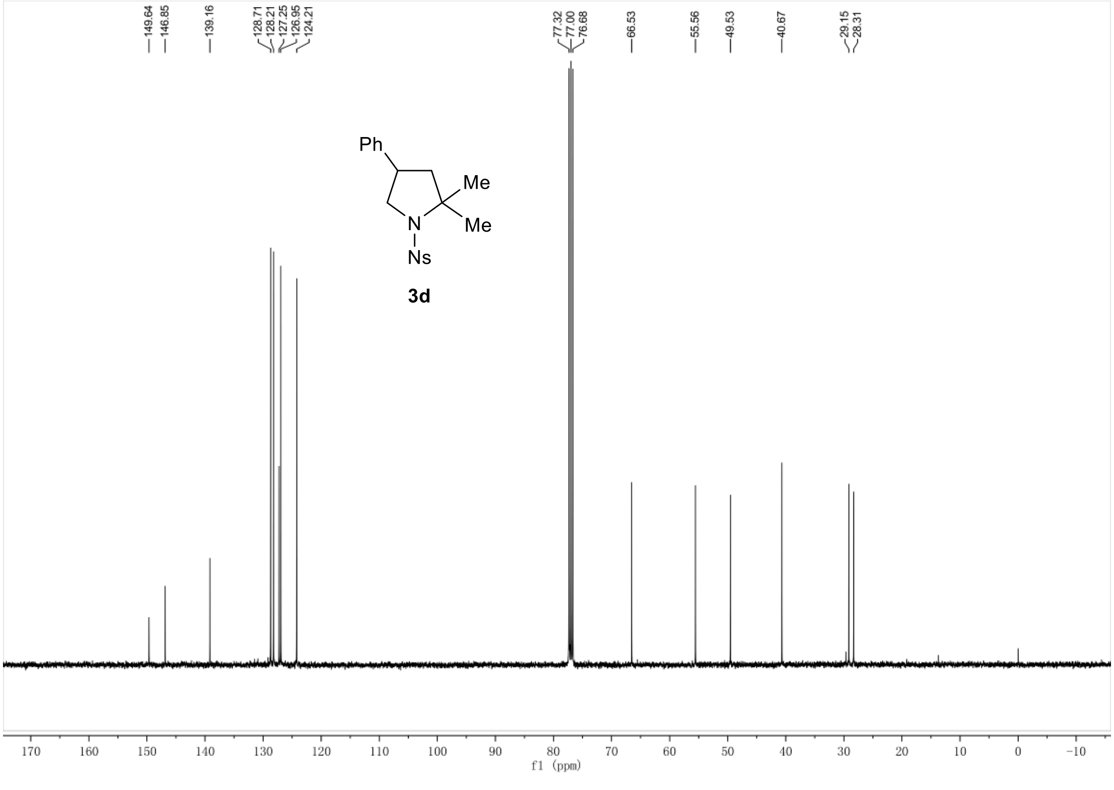

Supplementary Figure 79. ^{13}C NMR spectrum of compound 3d ($CDCl_3$, 100 MHz, 298K)

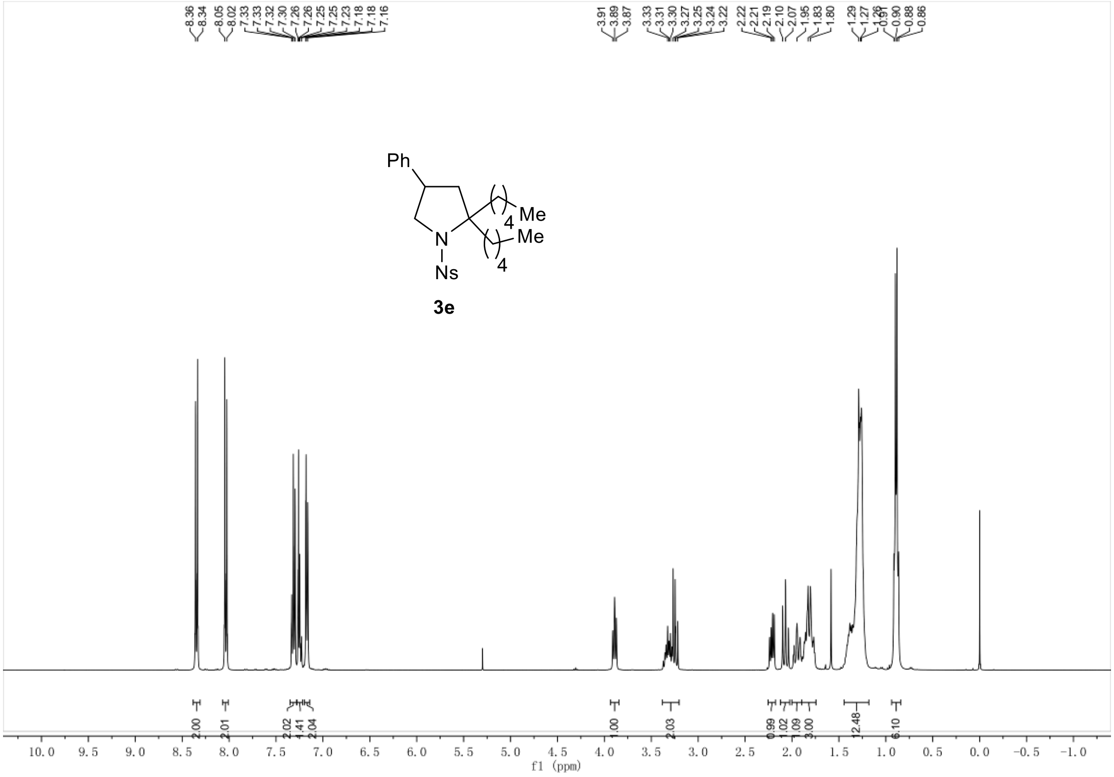

Supplementary Figure 80. ^{1}H NMR spectrum of compound 3e ($CDCl_3$, 400 MHz, 298K)

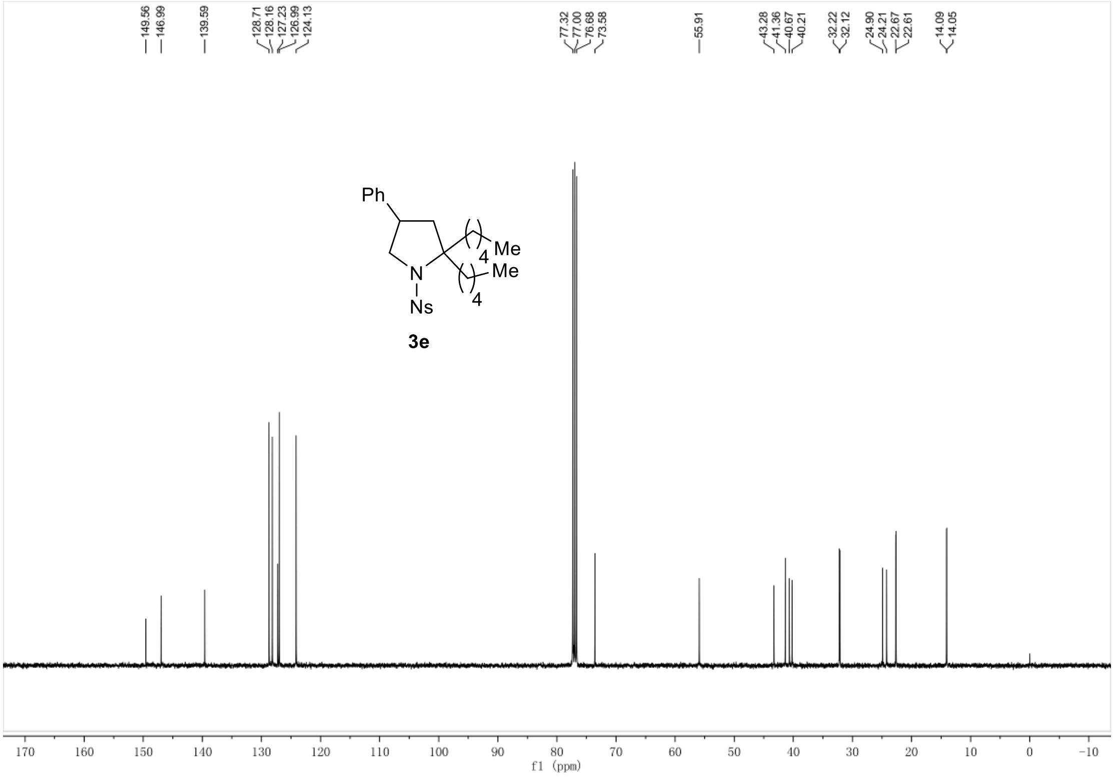

Supplementary Figure 81. ^{13}C NMR spectrum of compound 3e ($CDCl_3$, 100 MHz, 298K)

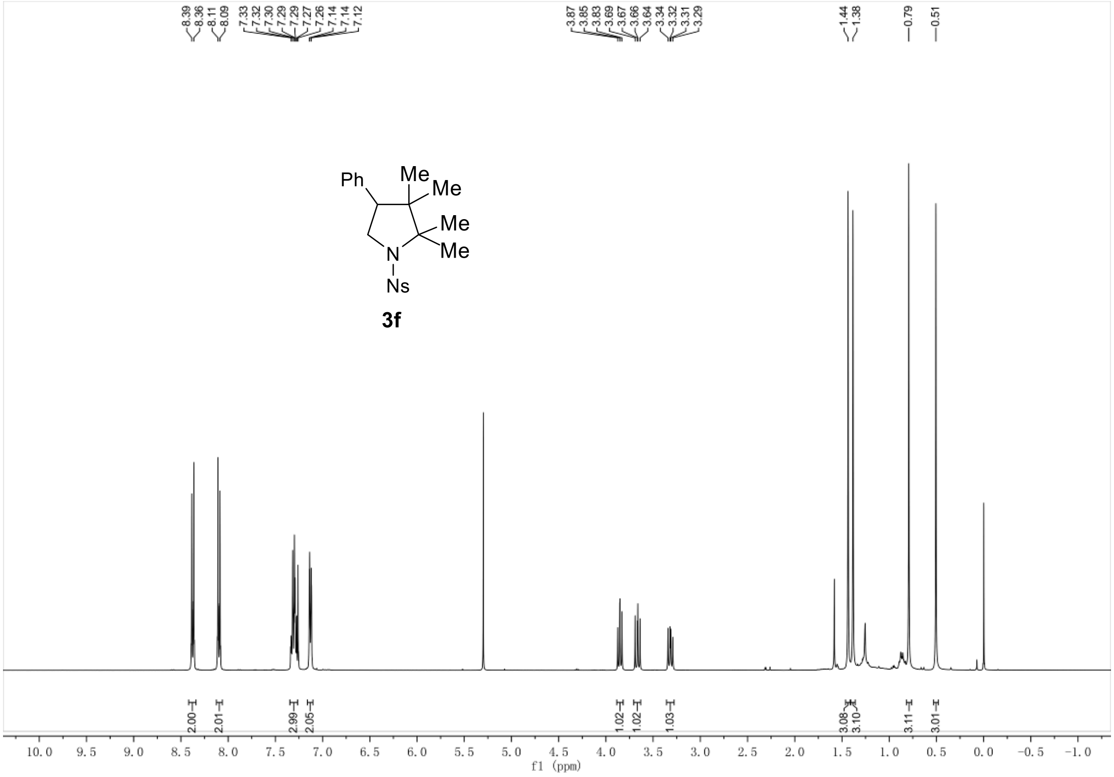

Supplementary Figure 82. ^{1}H NMR spectrum of compound 3f (CDCl$_3$, 400 MHz, 298K)

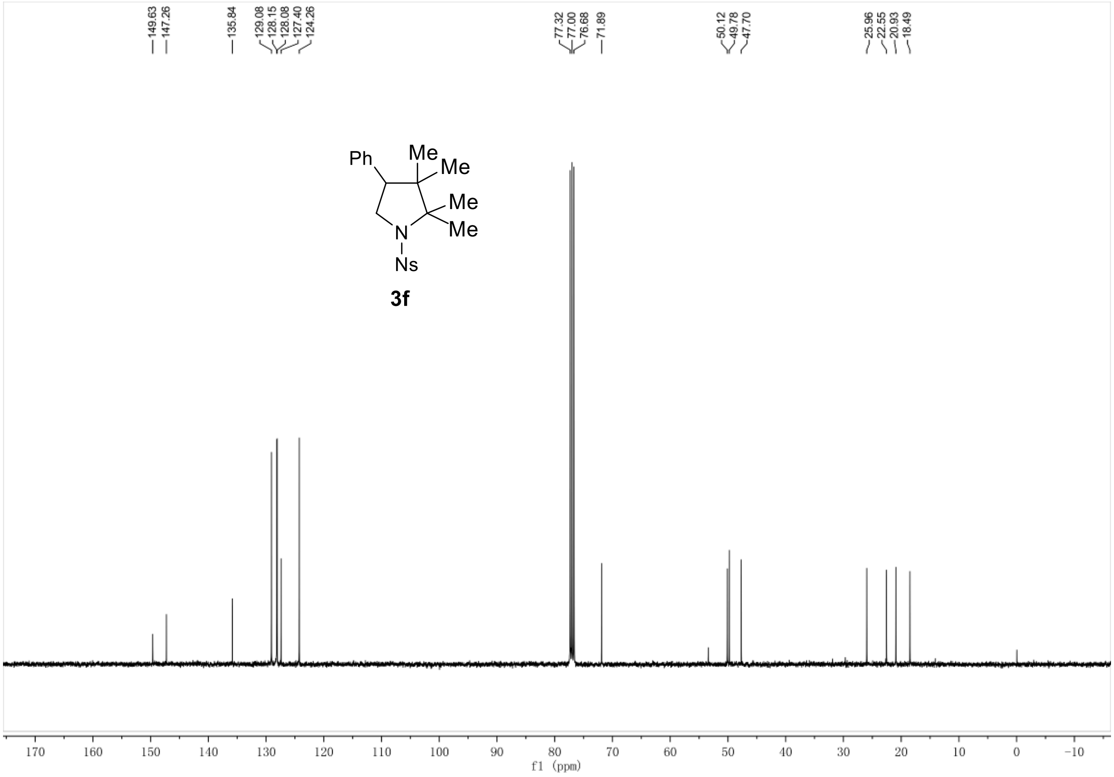

Supplementary Figure 83. ^{13}C NMR spectrum of compound 3f (CDCl$_3$, 100 MHz, 298K)

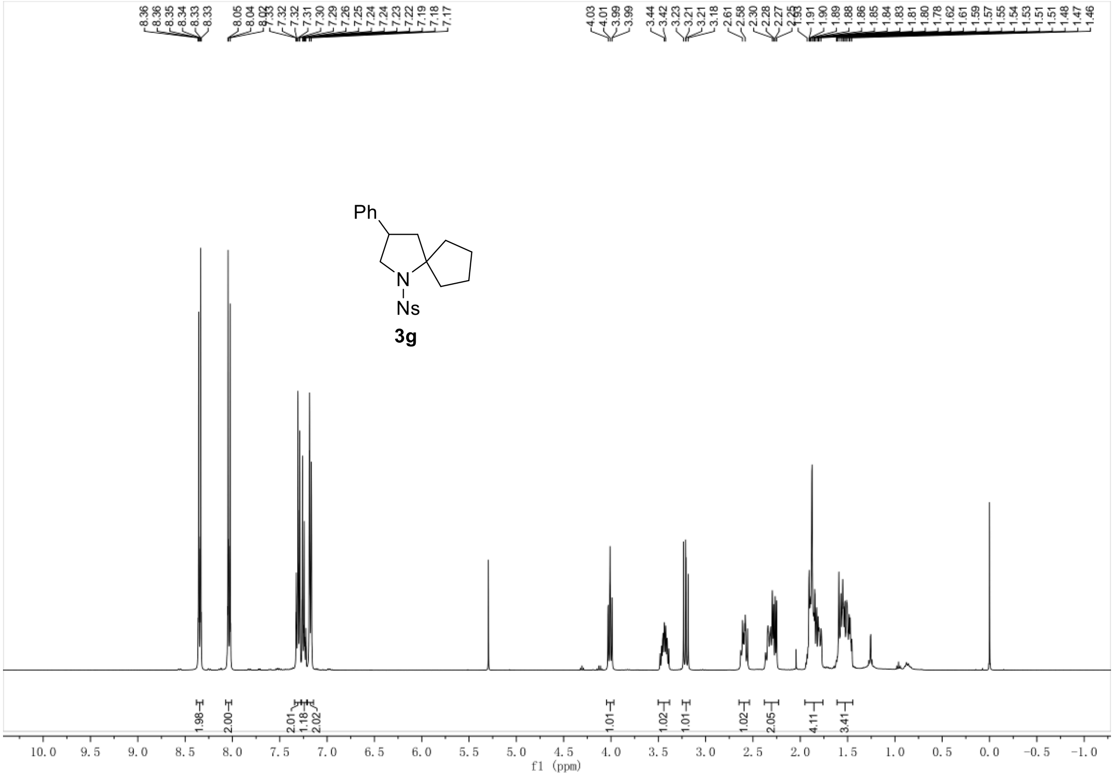

Supplementary Figure 84. ¹H NMR spectrum of compound 3g (CDCl₃, 400 MHz, 298K)

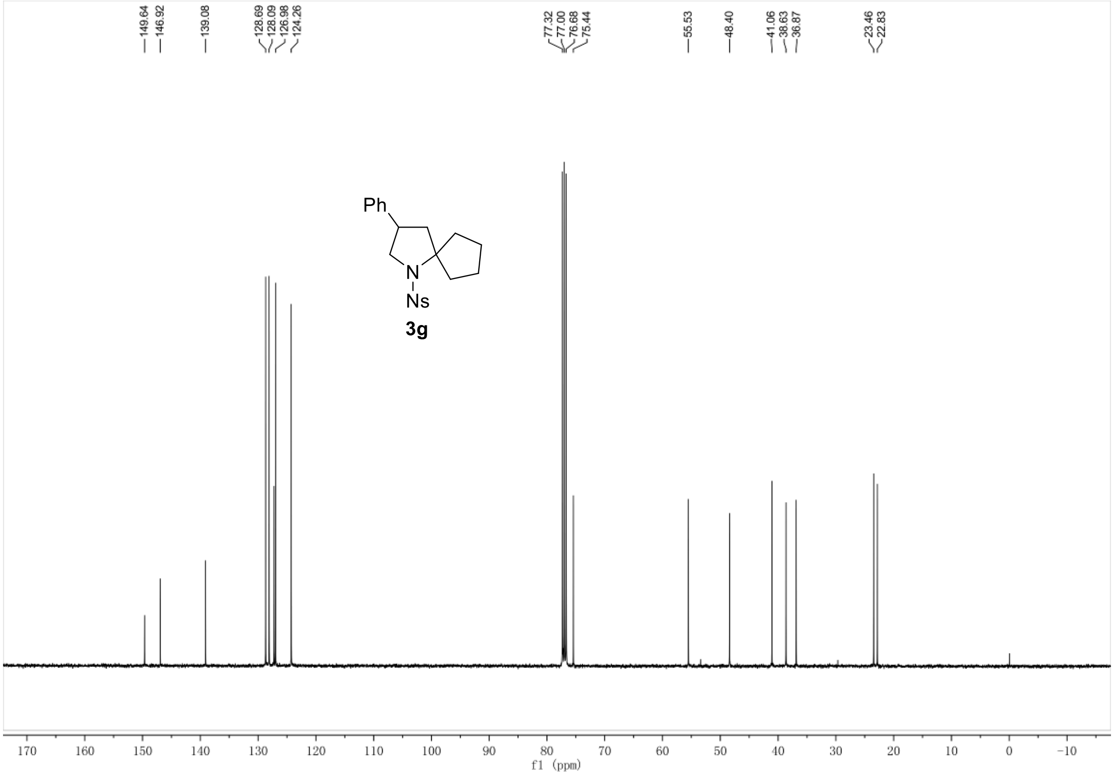

Supplementary Figure 85. ¹³C NMR spectrum of compound 3g (CDCl₃, 100 MHz, 298K)

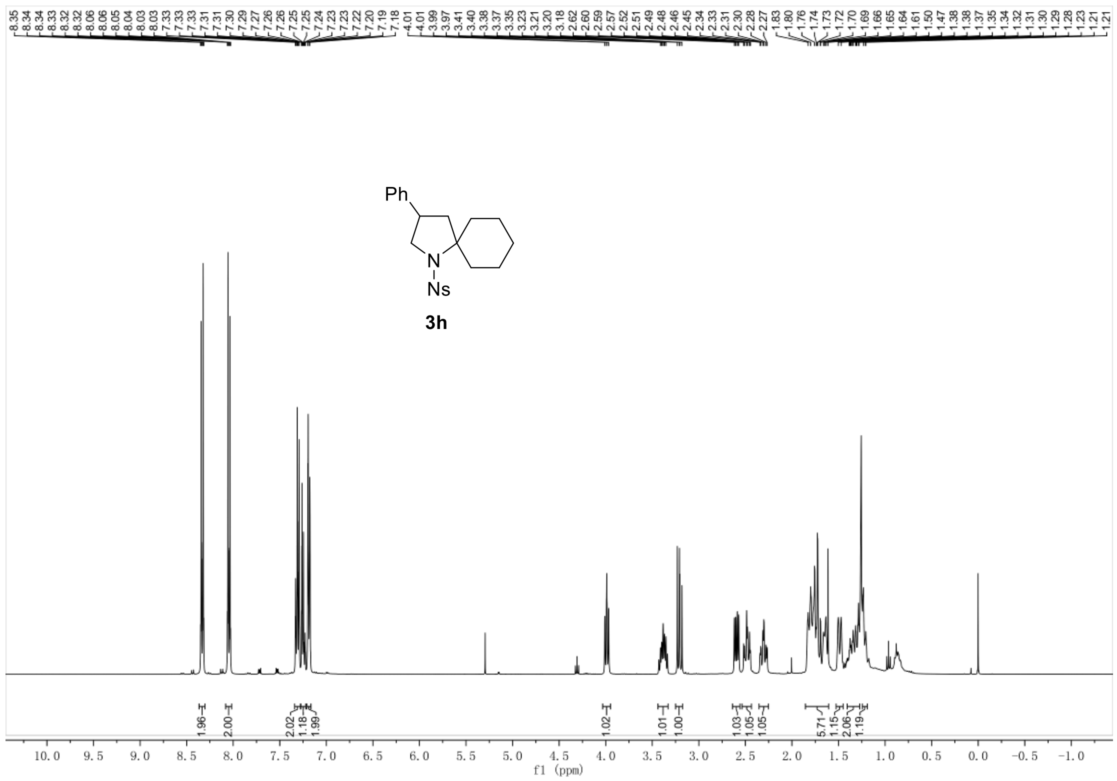

Supplementary Figure 86. ^{1}H NMR spectrum of compound 3h ($CDCl_3$, 400 MHz, 298K)

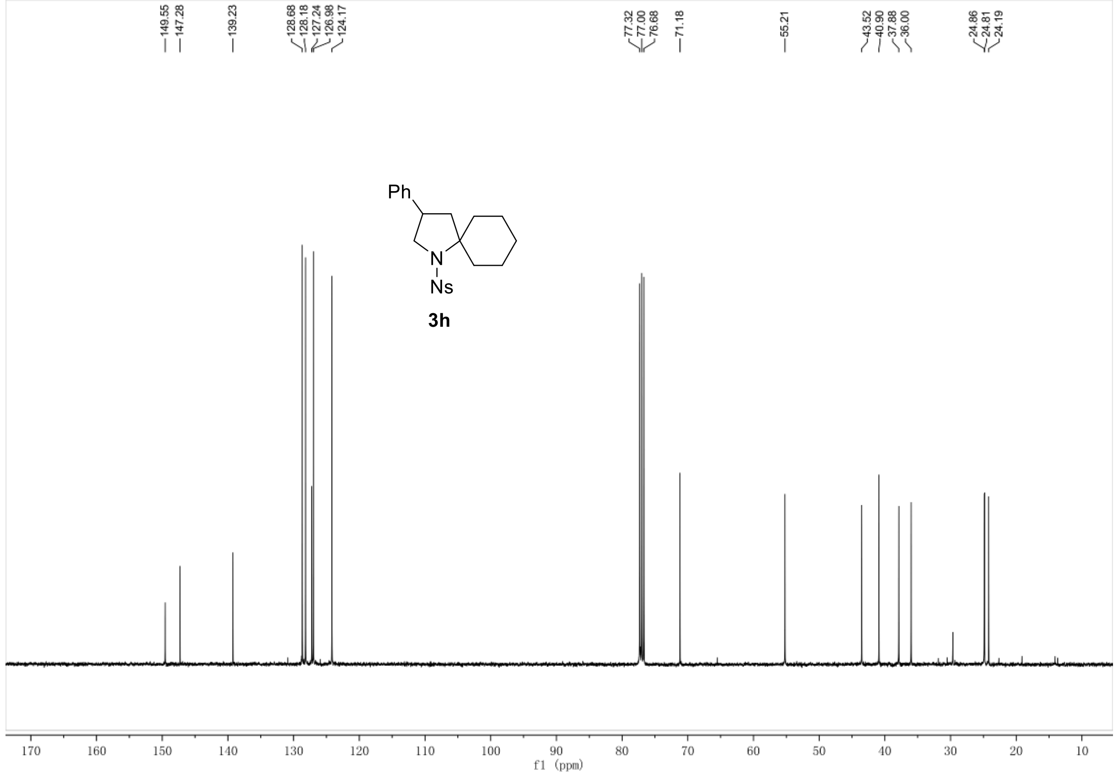

Supplementary Figure 87. ^{13}C NMR spectrum of compound 3h ($CDCl_3$, 100 MHz, 298K)

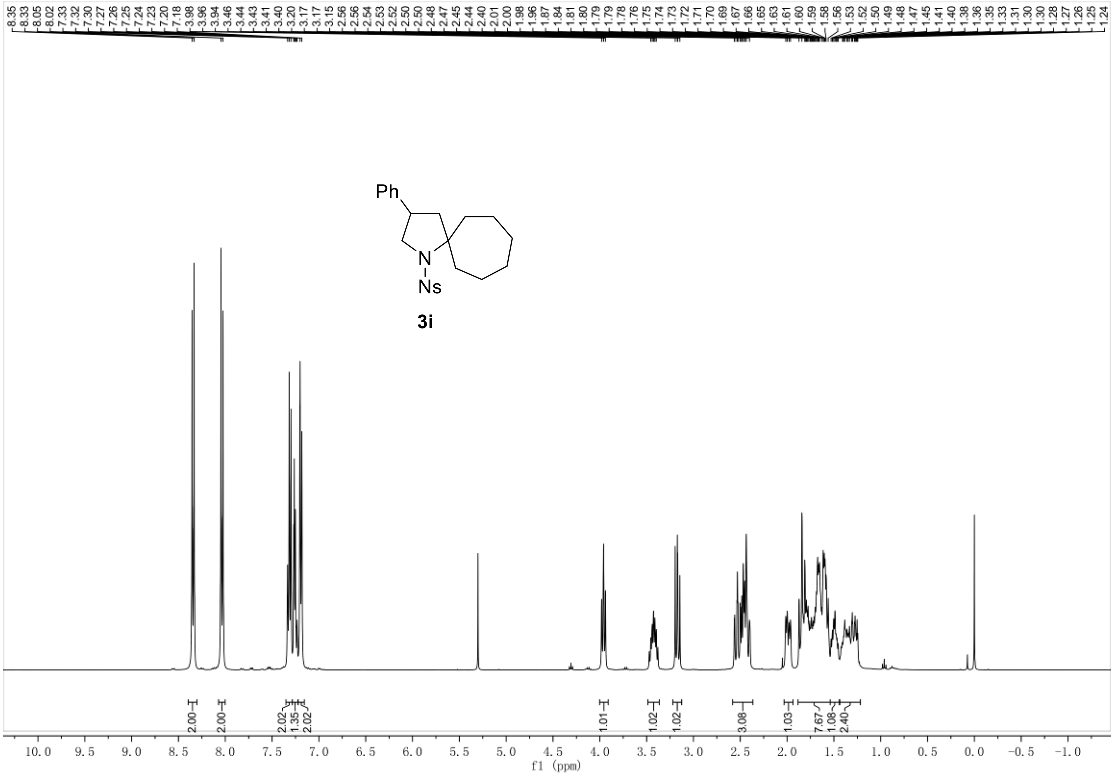

Supplementary Figure 88. **^{1}H NMR spectrum of compound 3i ($CDCl_3$, 400 MHz, 298K)**

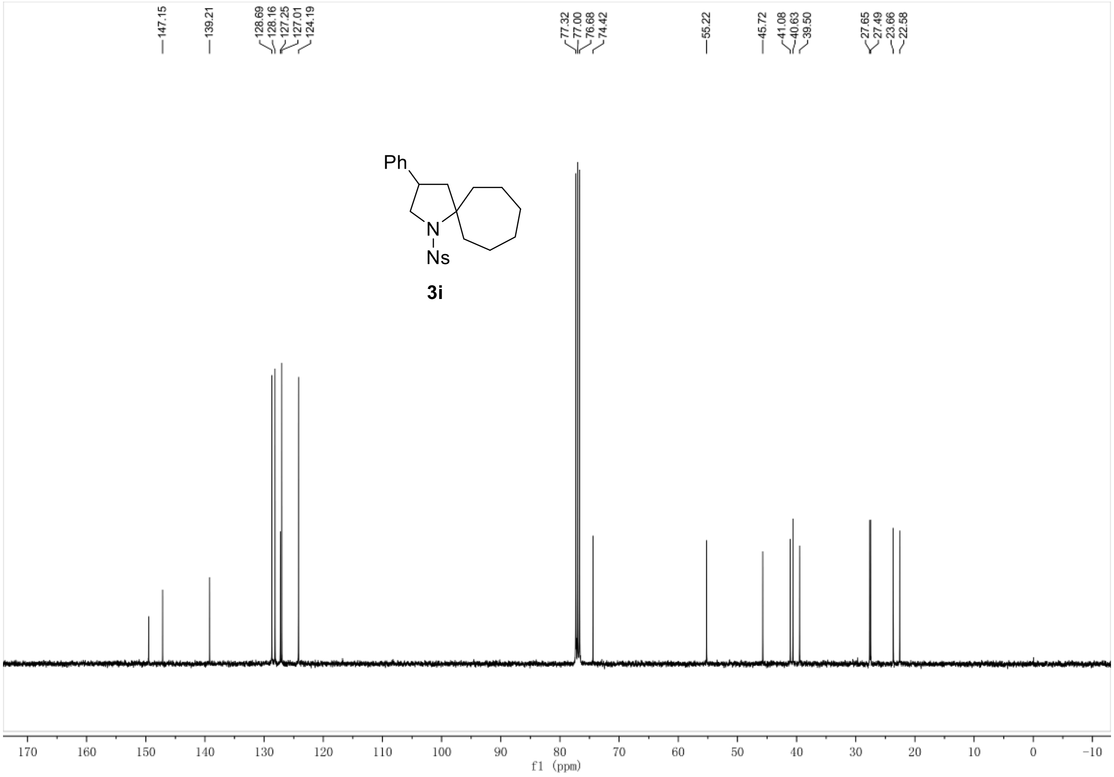

Supplementary Figure 89. **^{13}C NMR spectrum of compound 3i ($CDCl_3$, 100 MHz, 298K)**

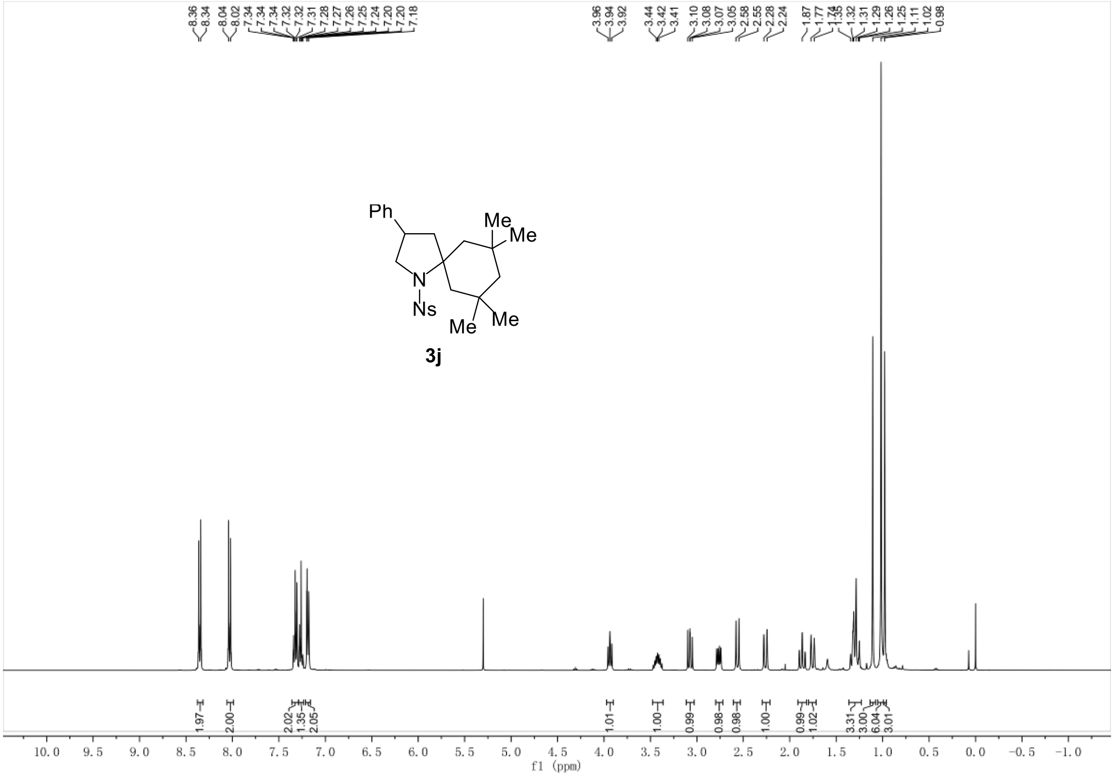

Supplementary Figure 90. ^{1}H NMR spectrum of compound 3j ($CDCl_3$, 400 MHz, 298K)

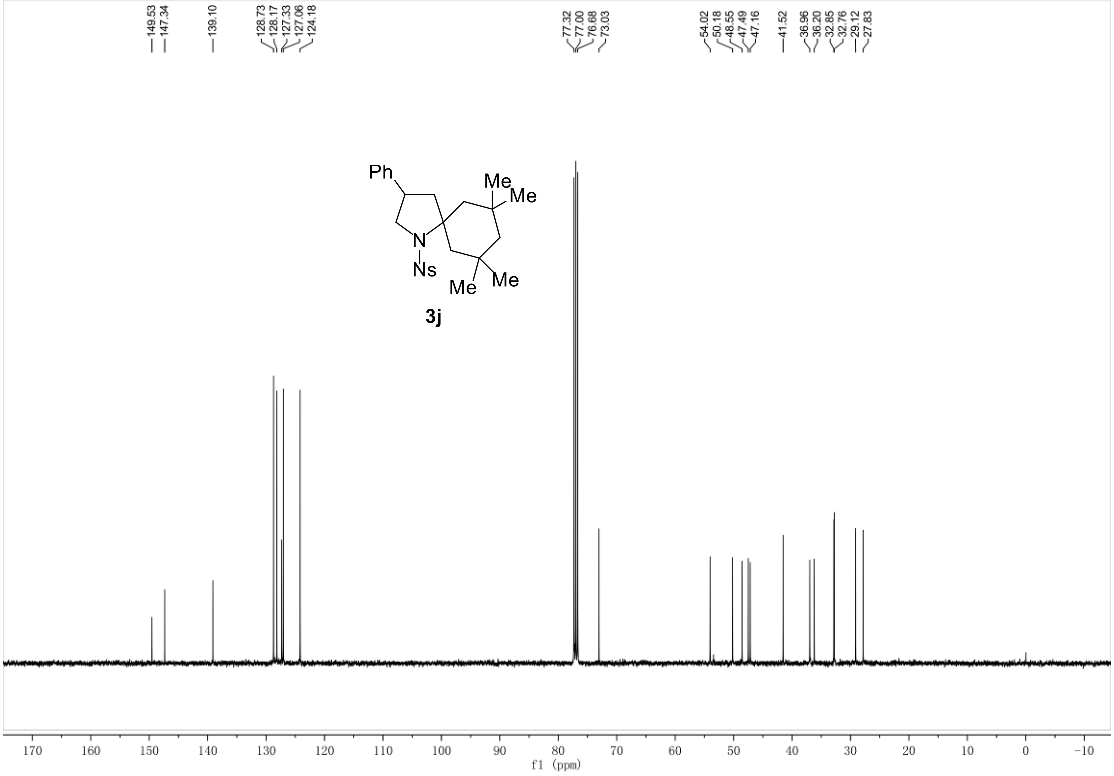

Supplementary Figure 91. ^{13}C NMR spectrum of compound 3j ($CDCl_3$, 100 MHz, 298K)

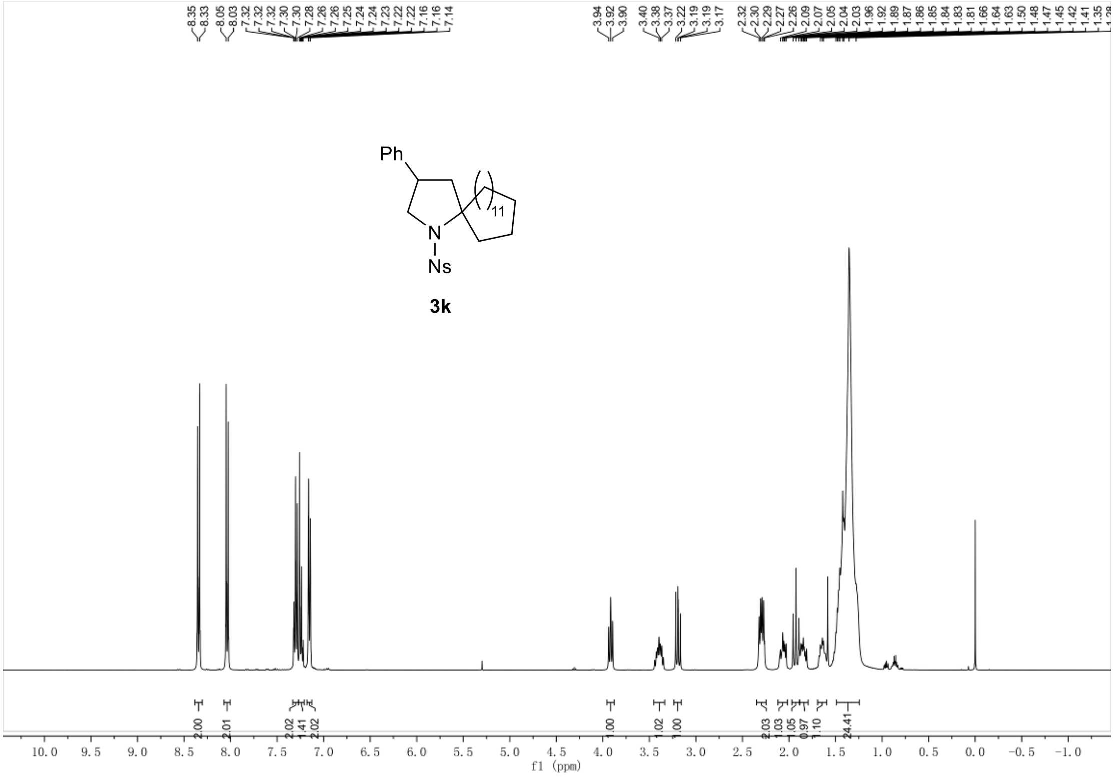

Supplementary Figure 92. ^{1}H NMR spectrum of compound 3k ($CDCl_3$, 400 MHz, 298K)

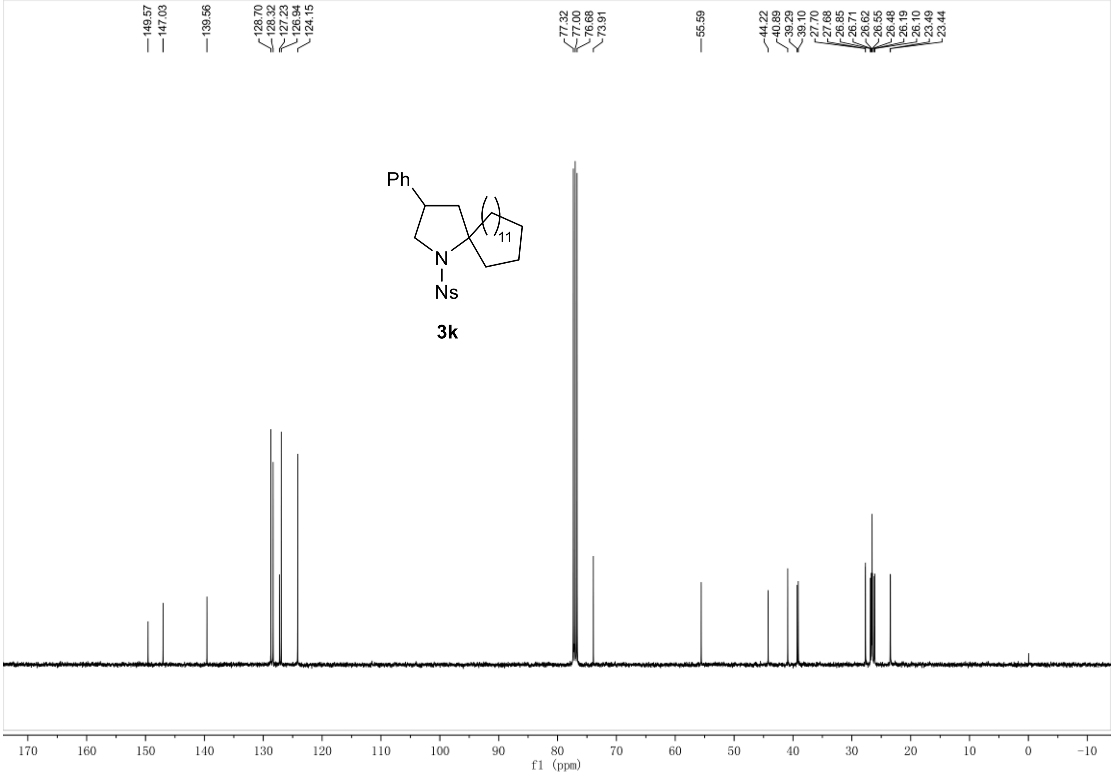

Supplementary Figure 93. ^{13}C NMR spectrum of compound 3k ($CDCl_3$, 100 MHz, 298K)

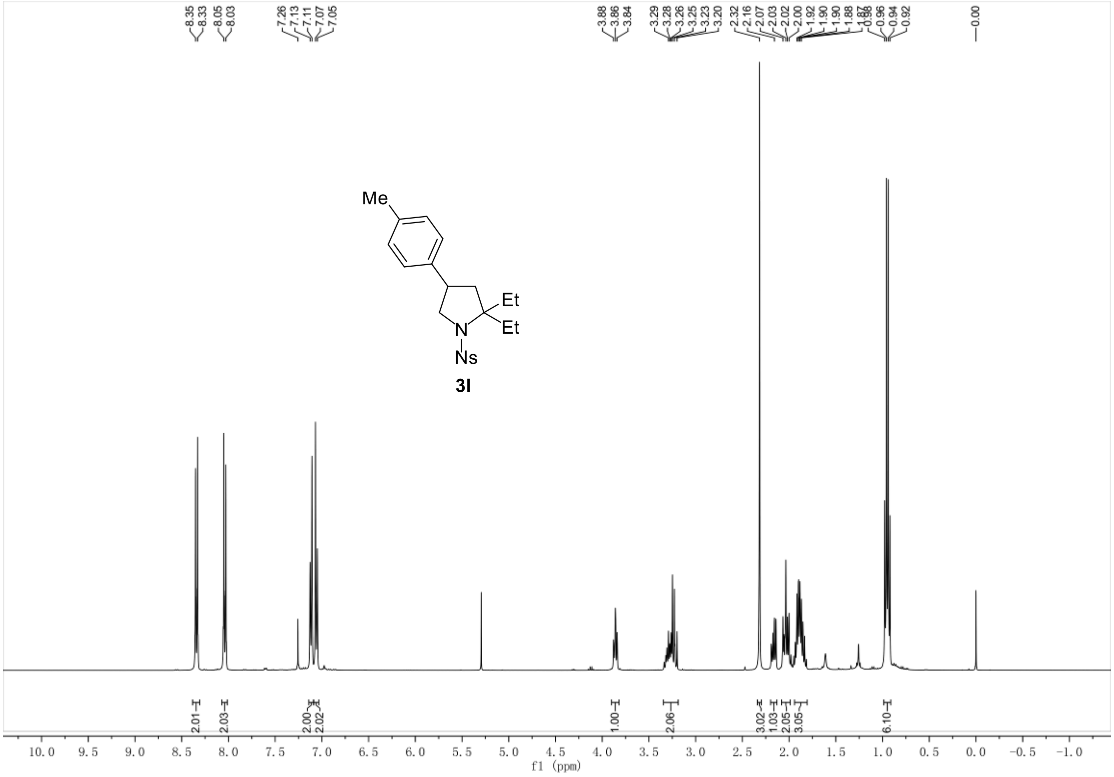

Supplementary Figure 94. ^{1}H NMR spectrum of compound 3l ($CDCl_3$, 400 MHz, 298K)

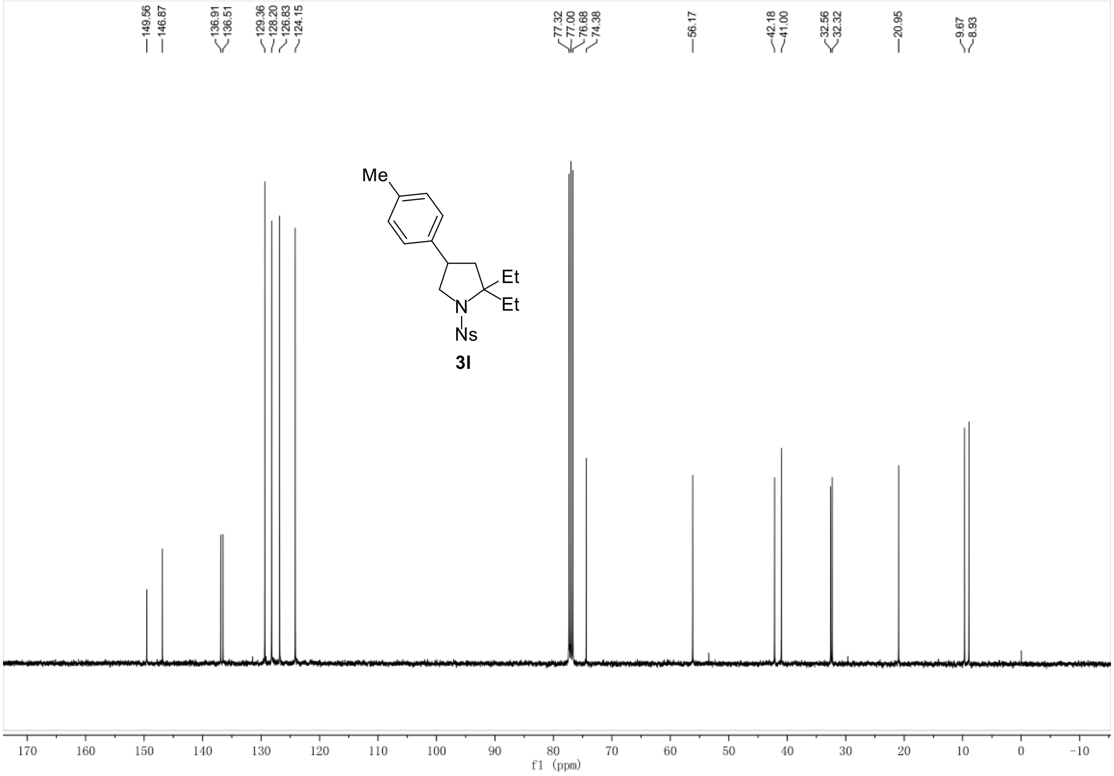

Supplementary Figure 95. ^{13}C NMR spectrum of compound 3l ($CDCl_3$, 100 MHz, 298K)

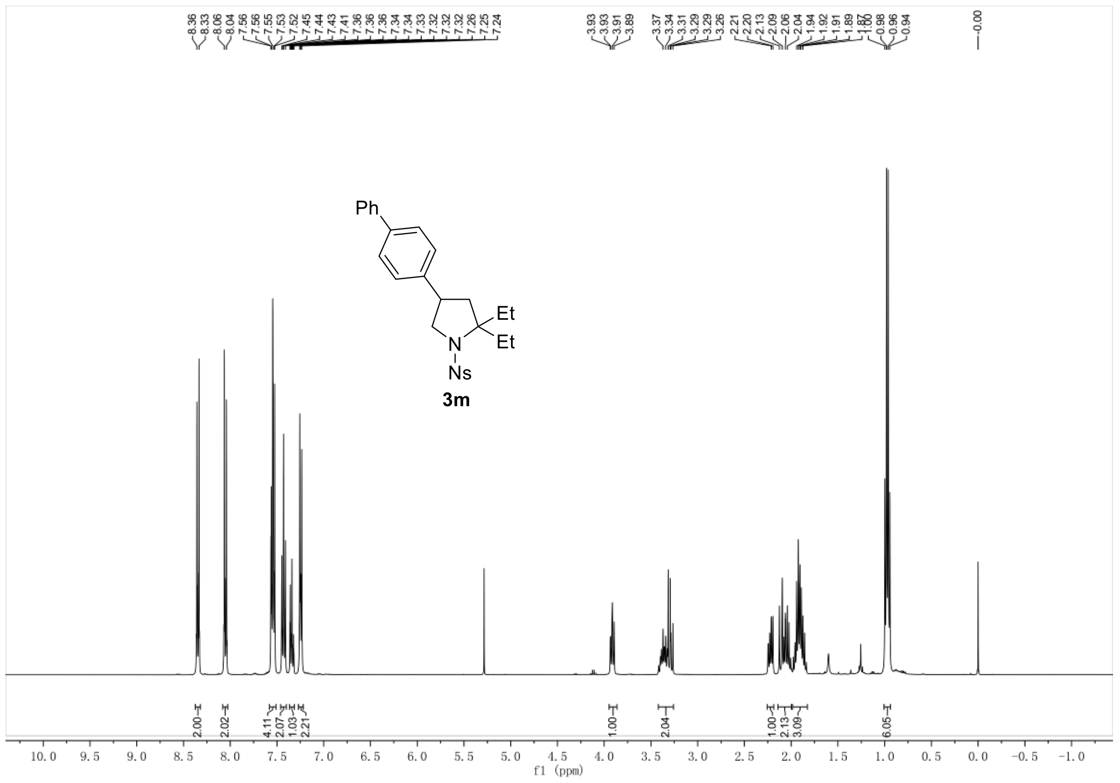

Supplementary Figure 96. ^{1}H NMR spectrum of compound 3m ($CDCl_3$, 400 MHz, 298K)

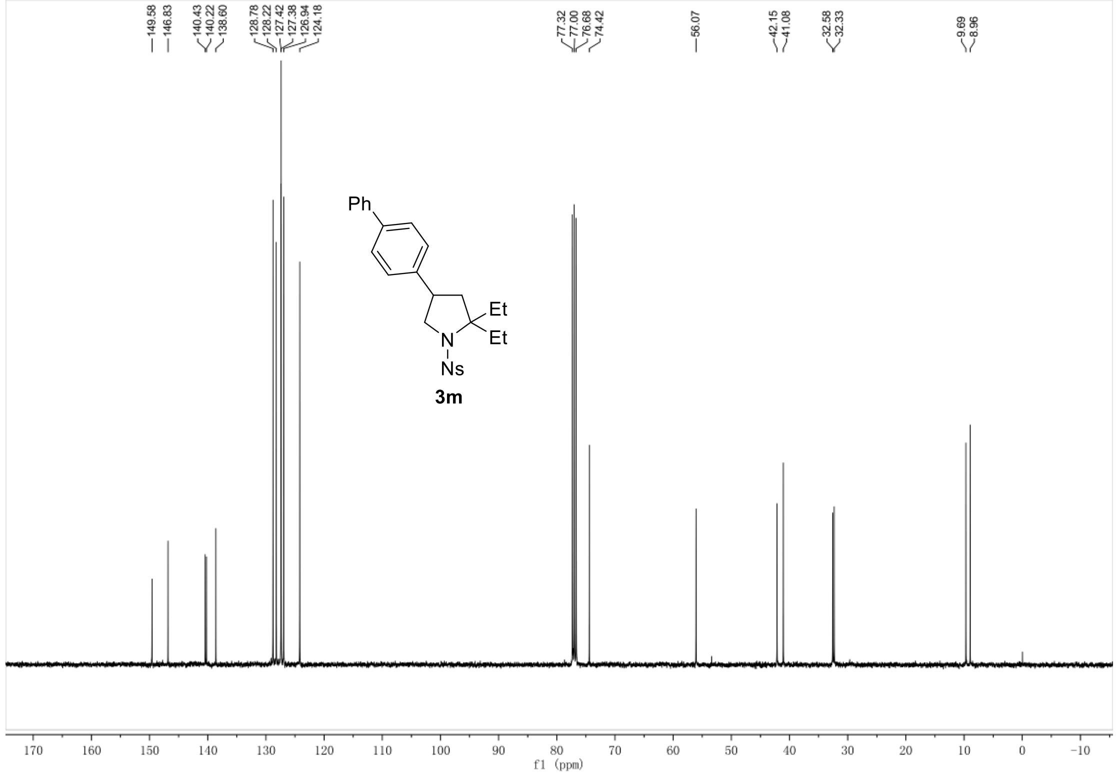

Supplementary Figure 97. ^{13}C NMR spectrum of compound 3m ($CDCl_3$, 100 MHz, 298K)

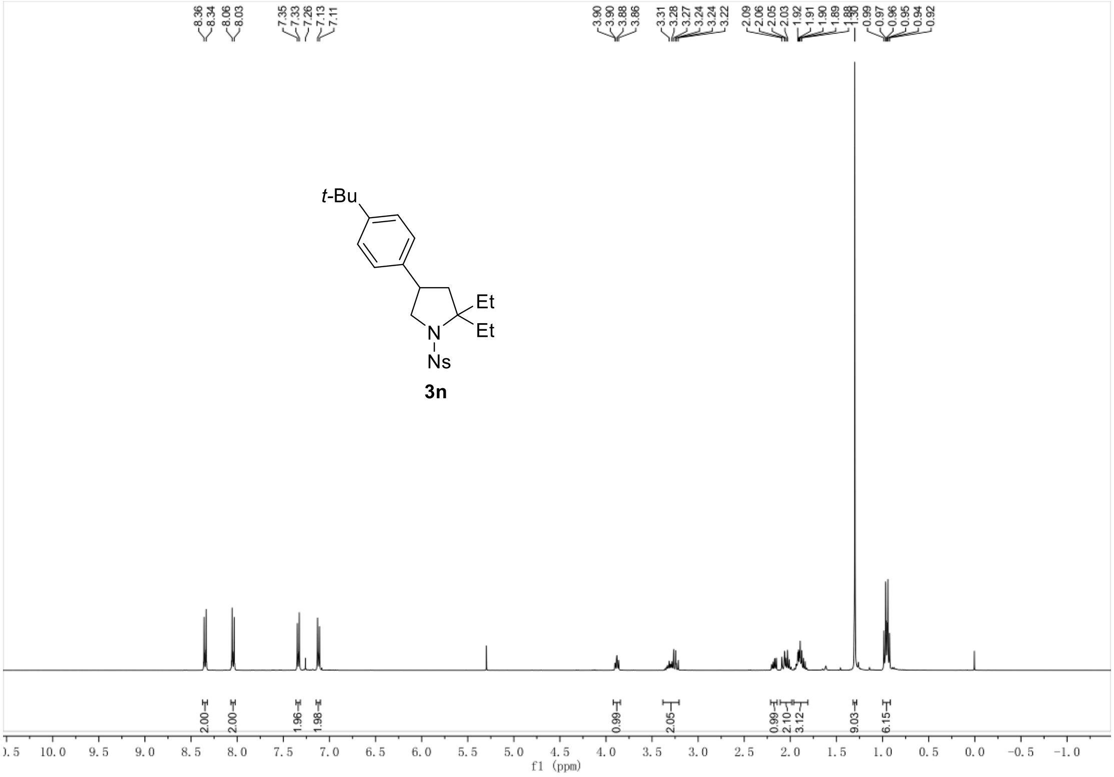

Supplementary Figure 98. ^{1}H NMR spectrum of compound 3n (CDCl$_3$, 400 MHz, 298K)

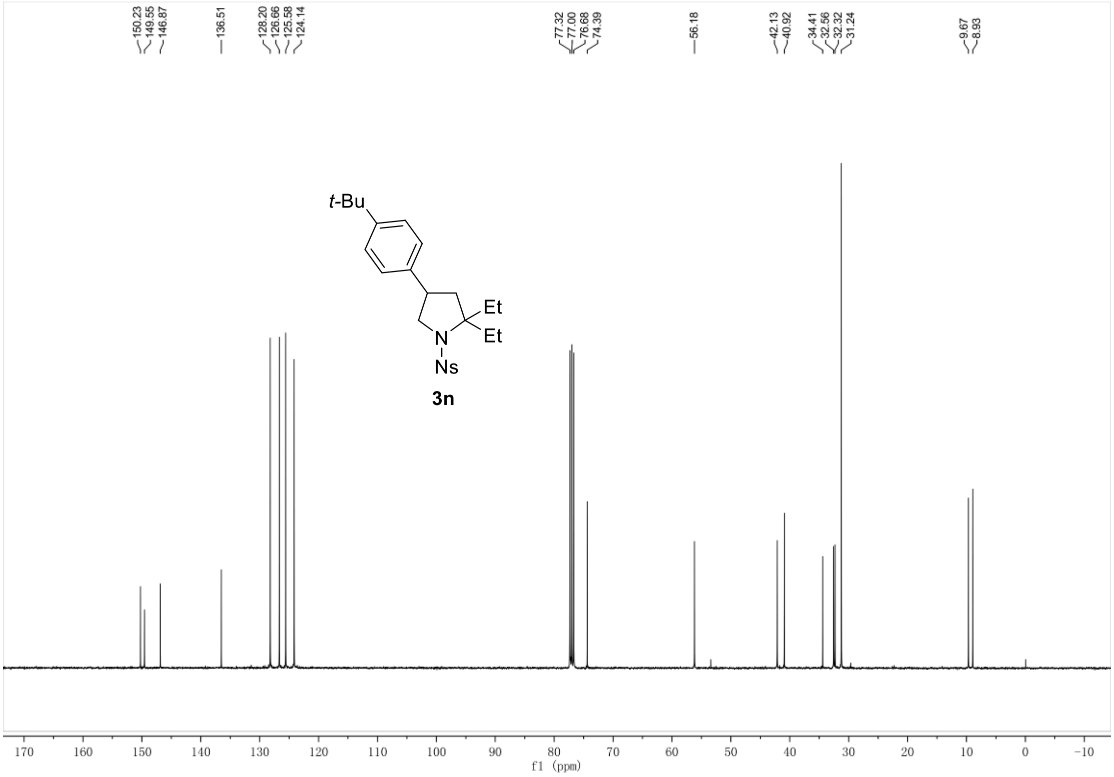

Supplementary Figure 99. ^{13}C NMR spectrum of compound 3n (CDCl$_3$, 100 MHz, 298K)

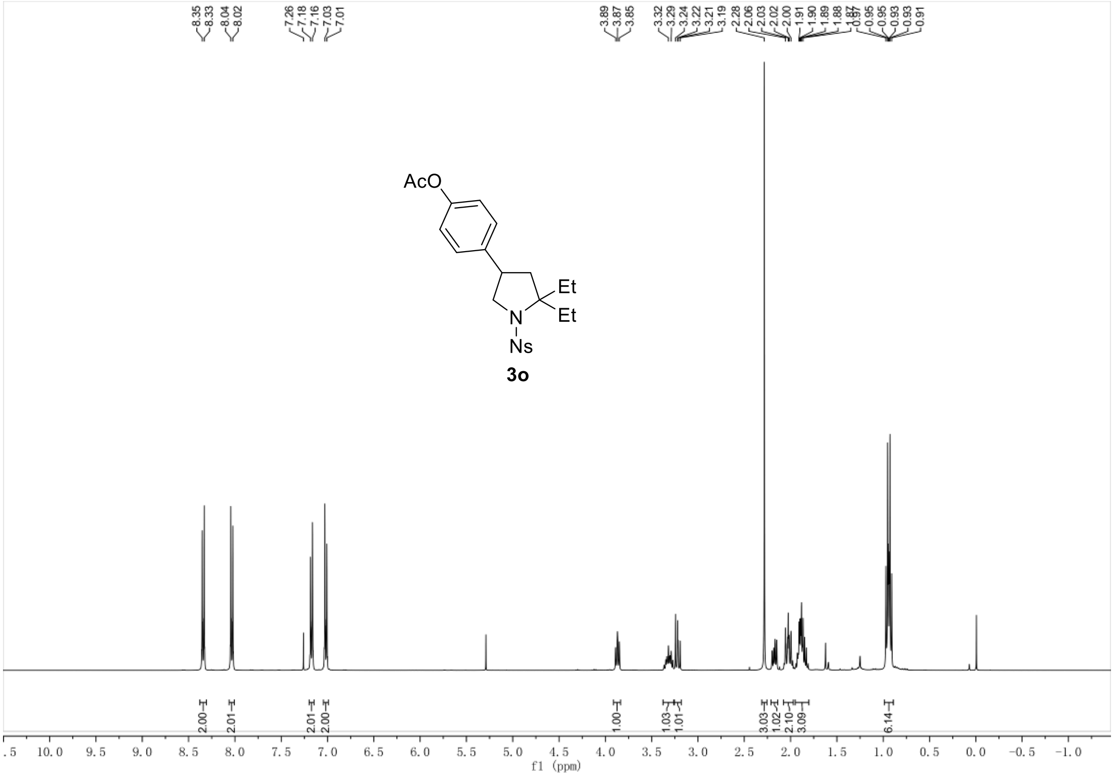

Supplementary Figure 100. ^{1}H NMR spectrum of compound 3o ($CDCl_3$, 400 MHz, 298K)

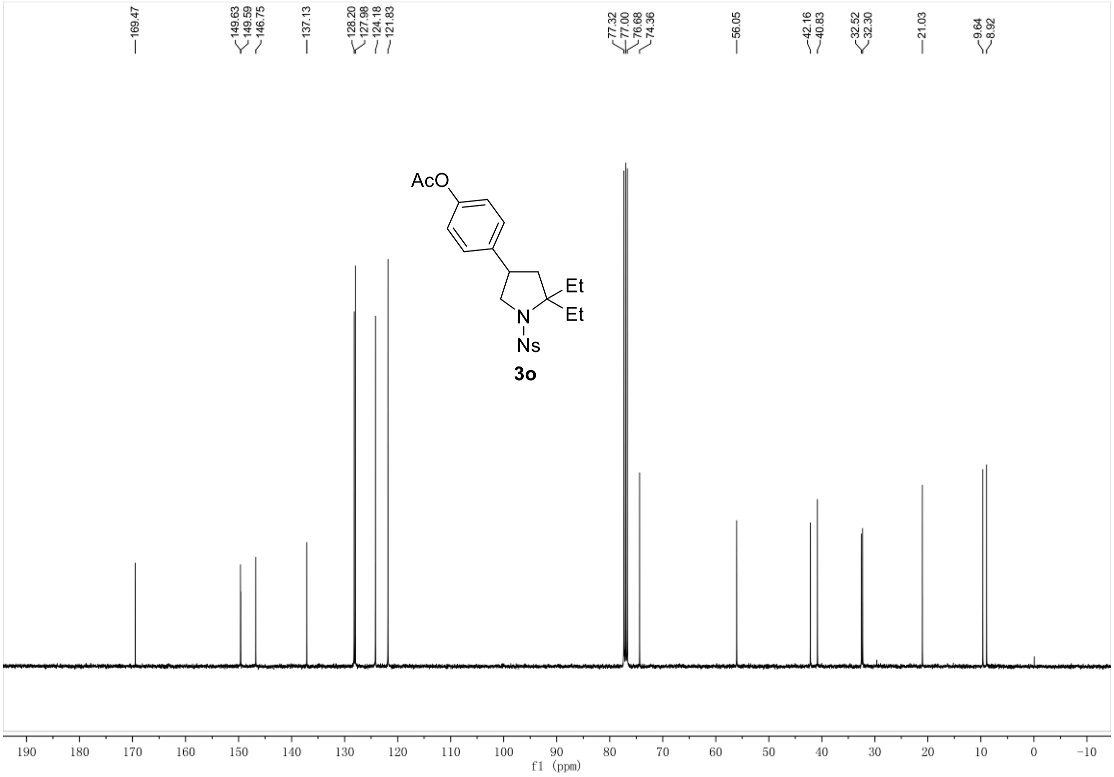

Supplementary Figure 101. ^{13}C NMR spectrum of compound 3o ($CDCl_3$, 100 MHz, 298K)

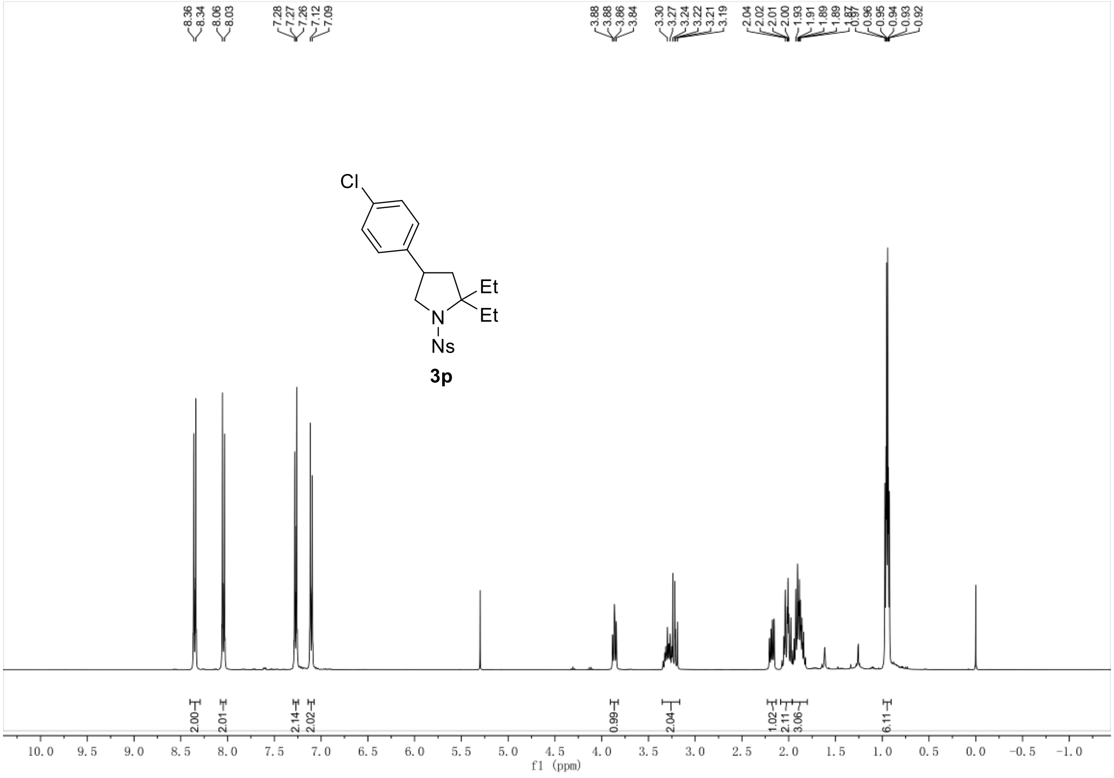

Supplementary Figure 102. ^{1}H NMR spectrum of compound 3p ($CDCl_3$, 400 MHz, 298K)

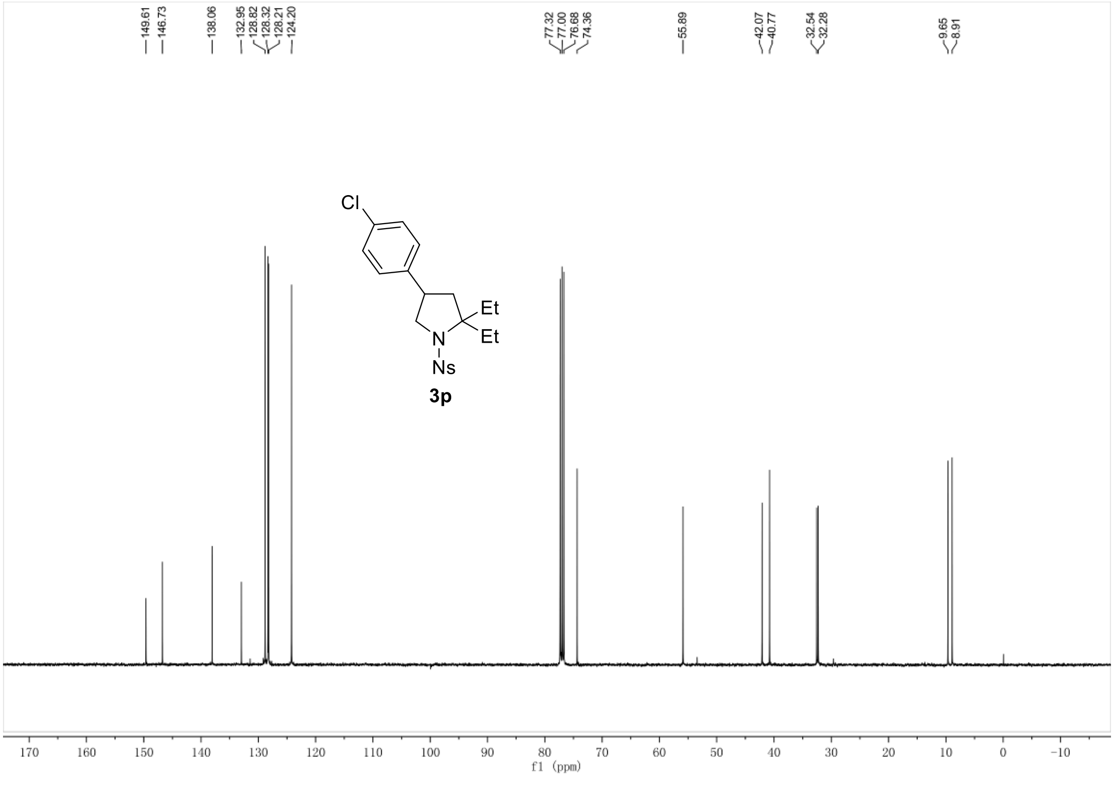

Supplementary Figure 103. ^{13}C NMR spectrum of compound 3p ($CDCl_3$, 100 MHz, 298K)

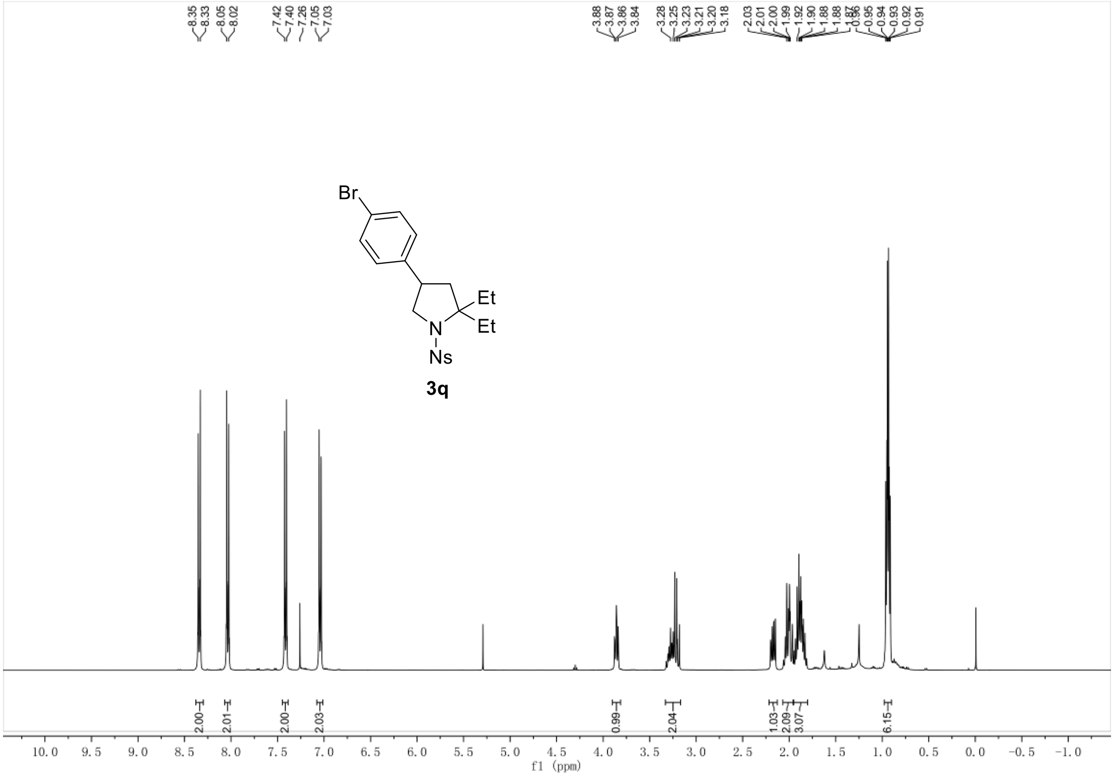

Supplementary Figure 104. ^{1}H NMR spectrum of compound 3q (CDCl$_3$, 400 MHz, 298K)

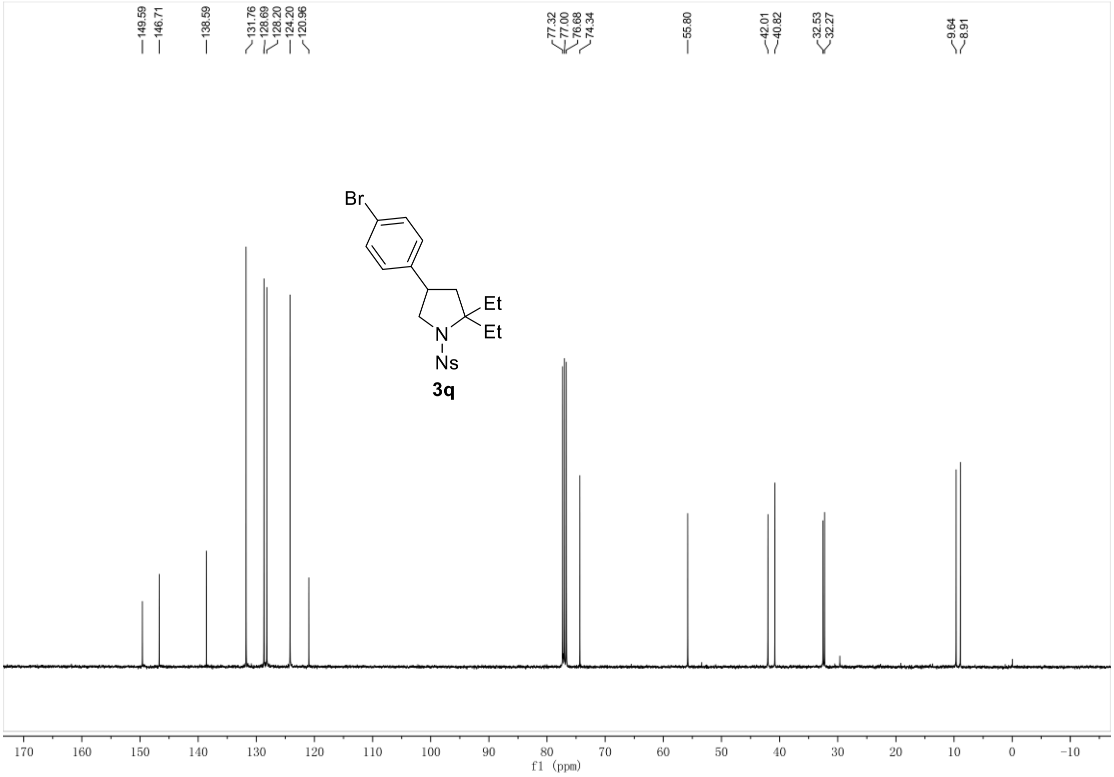

Supplementary Figure 105. ^{13}C NMR spectrum of compound 3q (CDCl$_3$, 100 MHz, 298K)

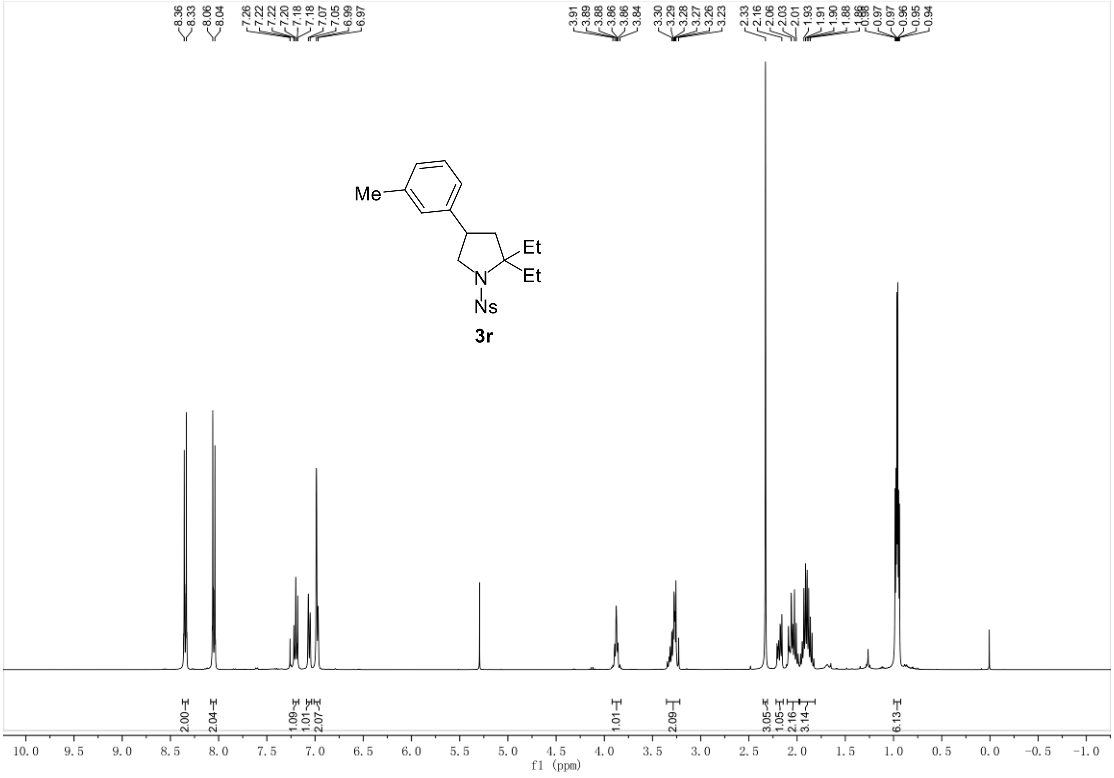

Supplementary Figure 106. ^{1}H NMR spectrum of compound 3r ($CDCl_3$, 400 MHz, 298K)

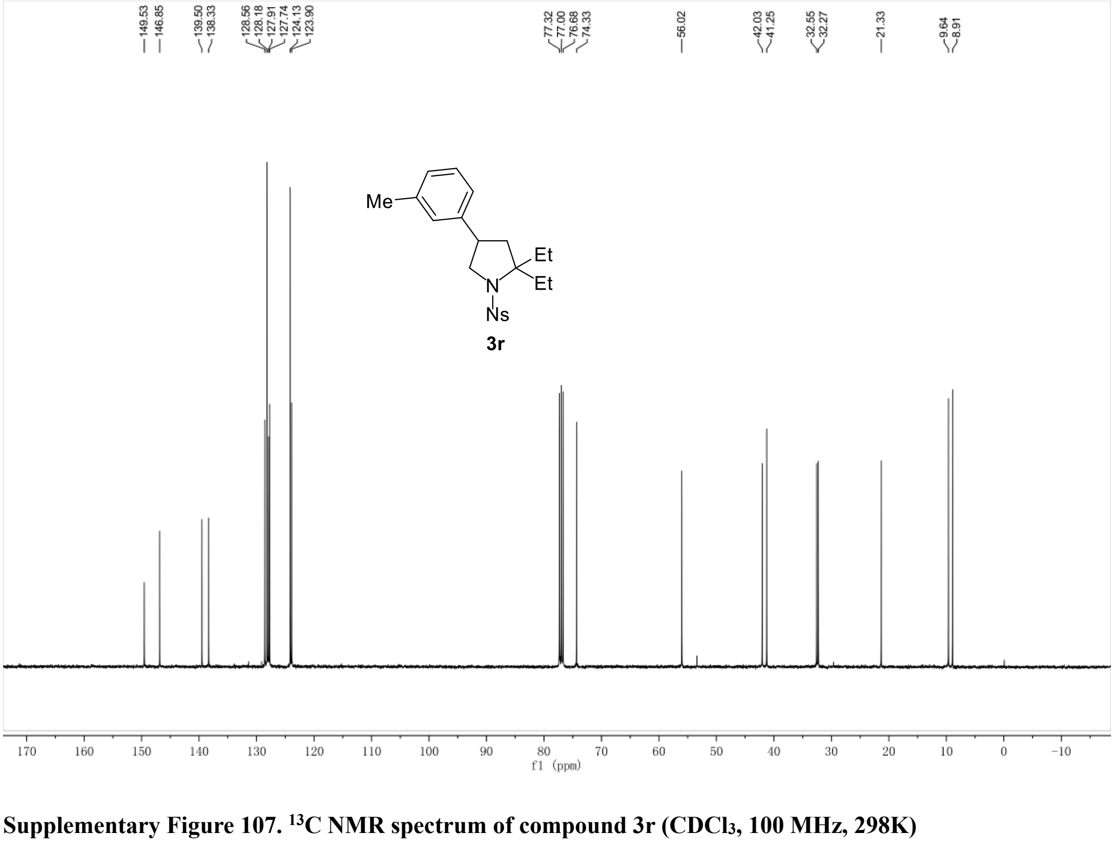

Supplementary Figure 107. ^{13}C NMR spectrum of compound 3r ($CDCl_3$, 100 MHz, 298K)

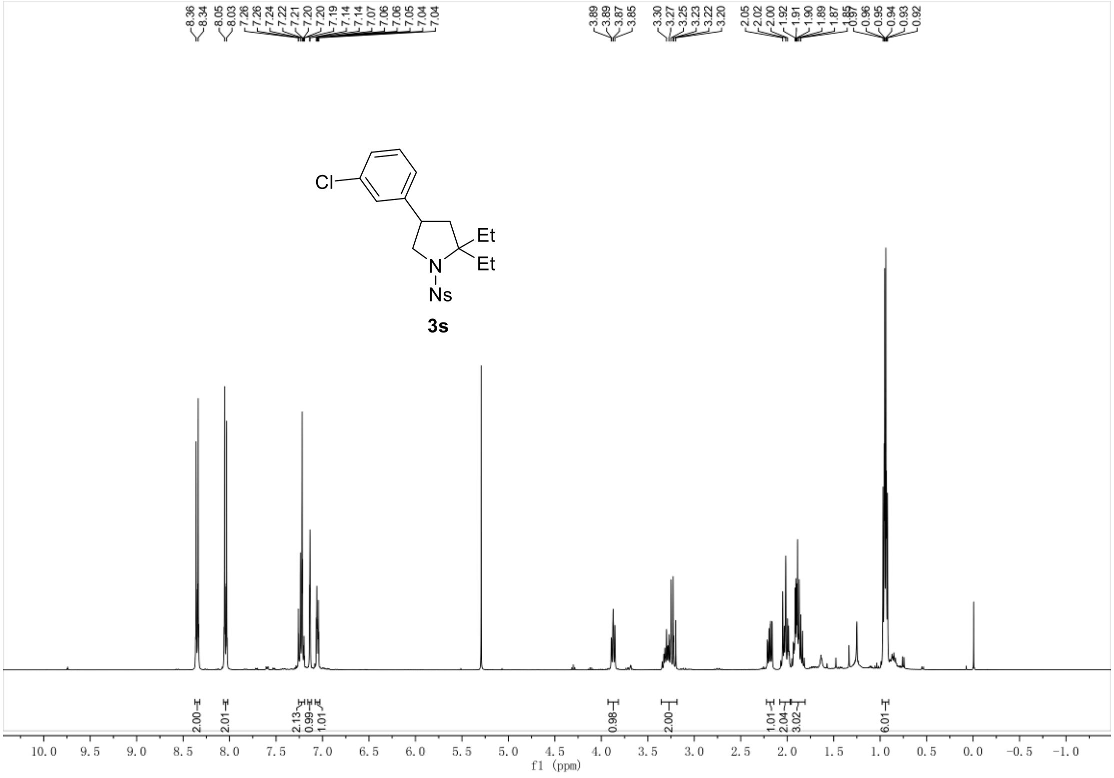

Supplementary Figure 108. ^{1}H NMR spectrum of compound 3s ($CDCl_3$, 400 MHz, 298K)

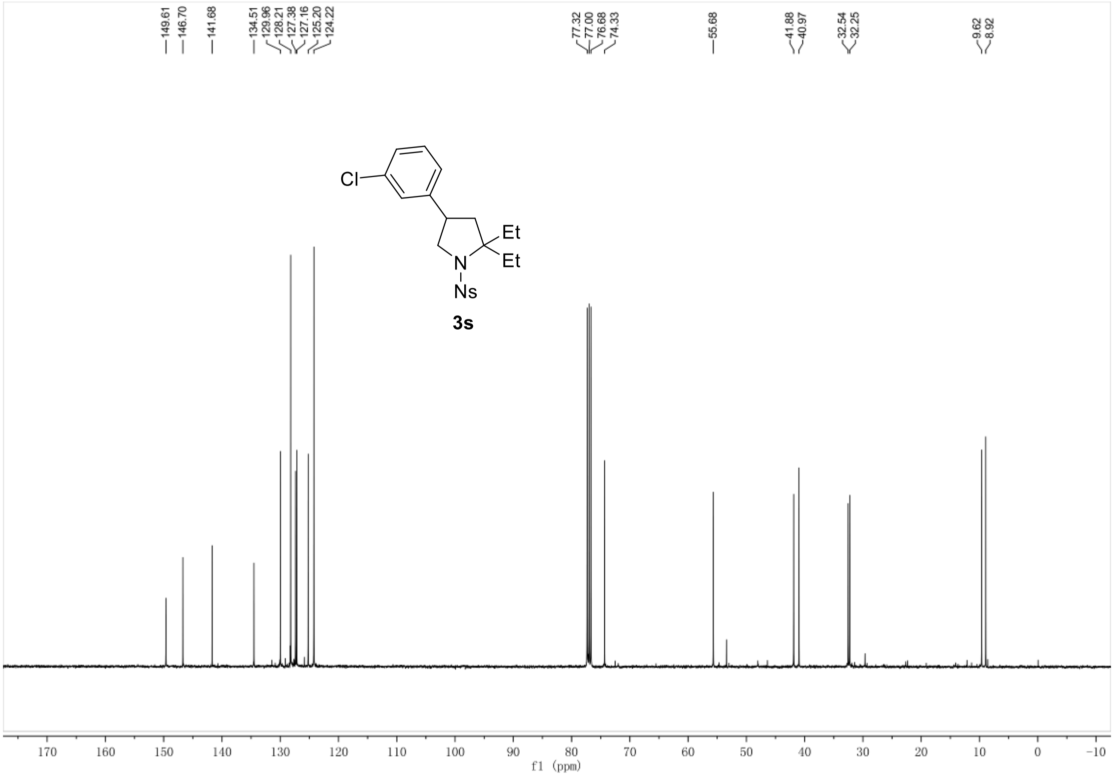

Supplementary Figure 109. ^{13}C NMR spectrum of compound 3s ($CDCl_3$, 100 MHz, 298K)

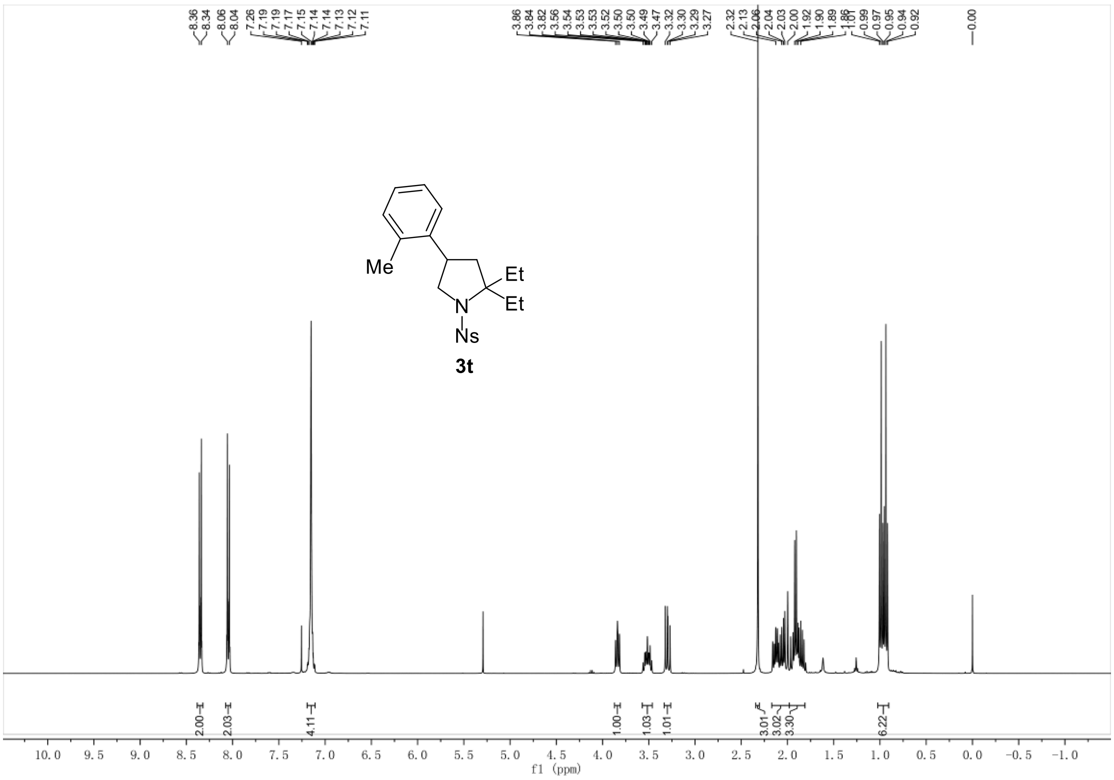

Supplementary Figure 110. ^{1}H NMR spectrum of compound 3t ($CDCl_3$, 400 MHz, 298K)

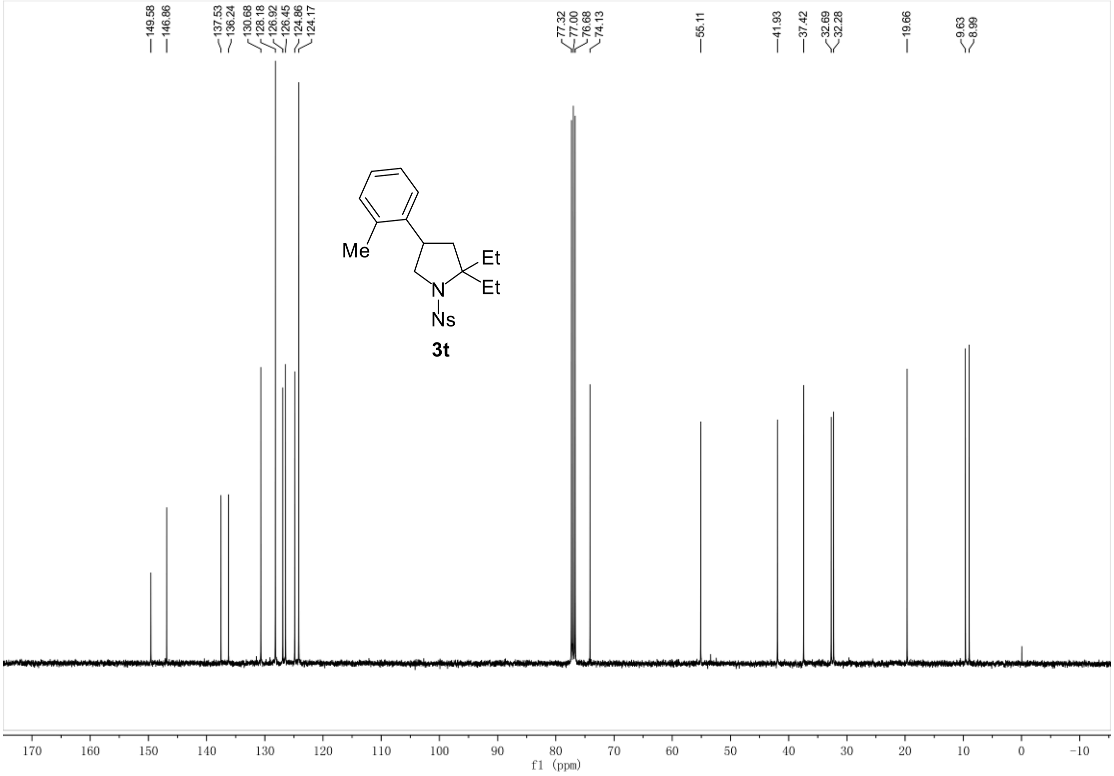

Supplementary Figure 111. ^{13}C NMR spectrum of compound 3t ($CDCl_3$, 100 MHz, 298K)

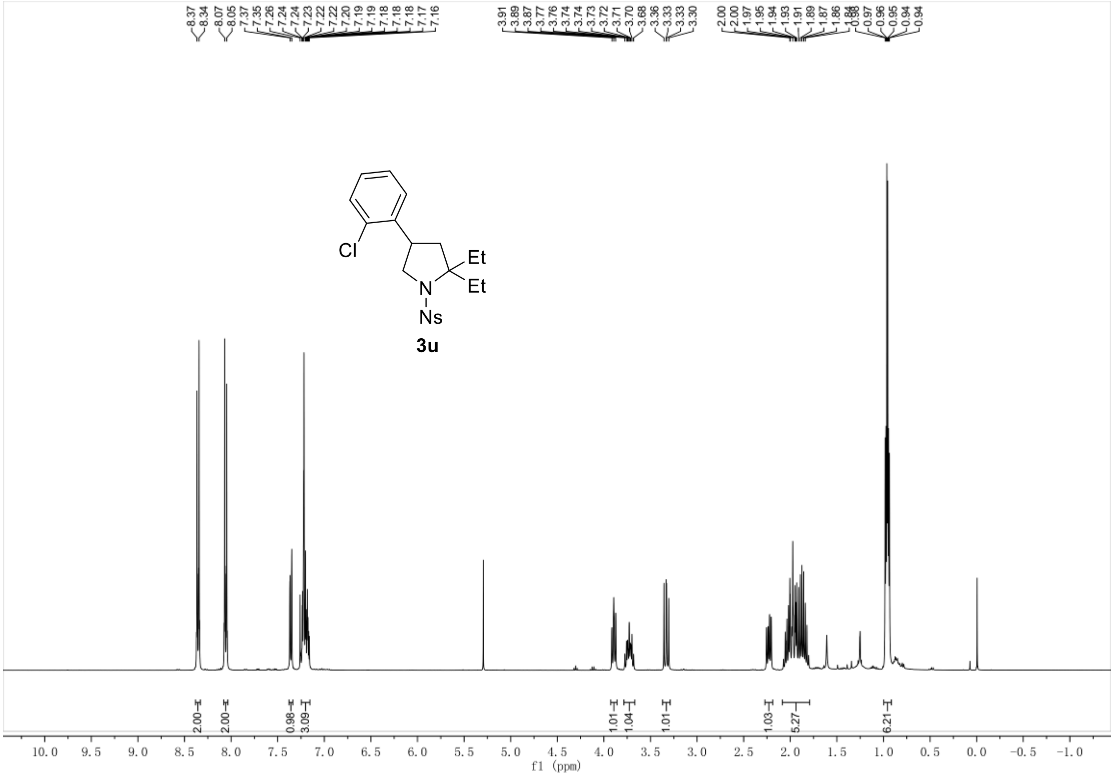

Supplementary Figure 112. ^{1}H NMR spectrum of compound 3u ($CDCl_3$, 400 MHz, 298K)

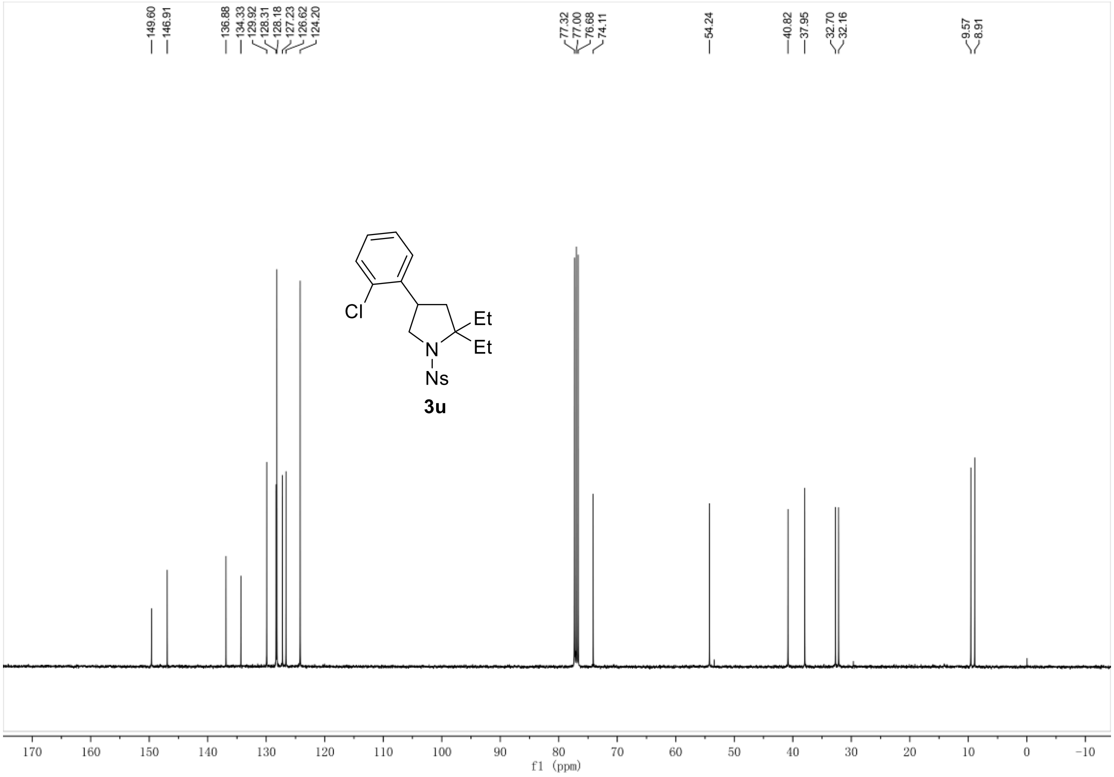

Supplementary Figure 113. ^{13}C NMR spectrum of compound 3u ($CDCl_3$, 100 MHz, 298K)

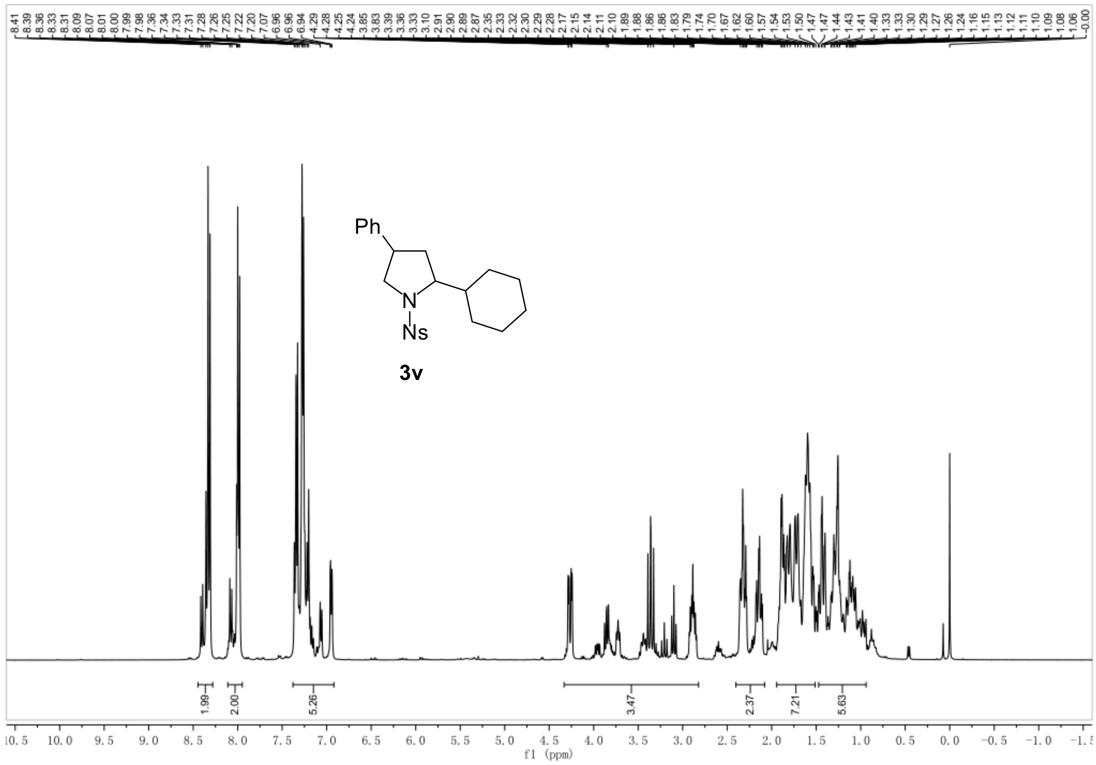

Supplementary Figure 114. ^{1}H NMR spectrum of compound 3v (CDCl$_3$, 400 MHz, 298K)

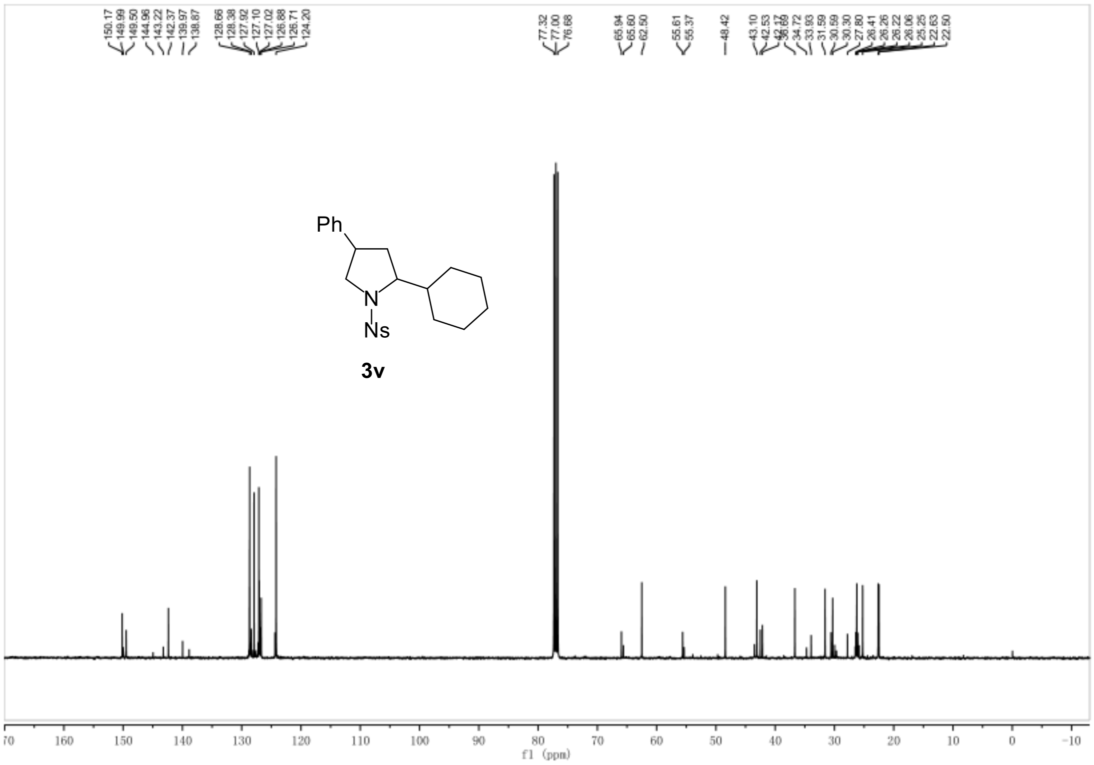

Supplementary Figure 115. ^{13}C NMR spectrum of compound 3v (CDCl$_3$, 100 MHz, 298K)

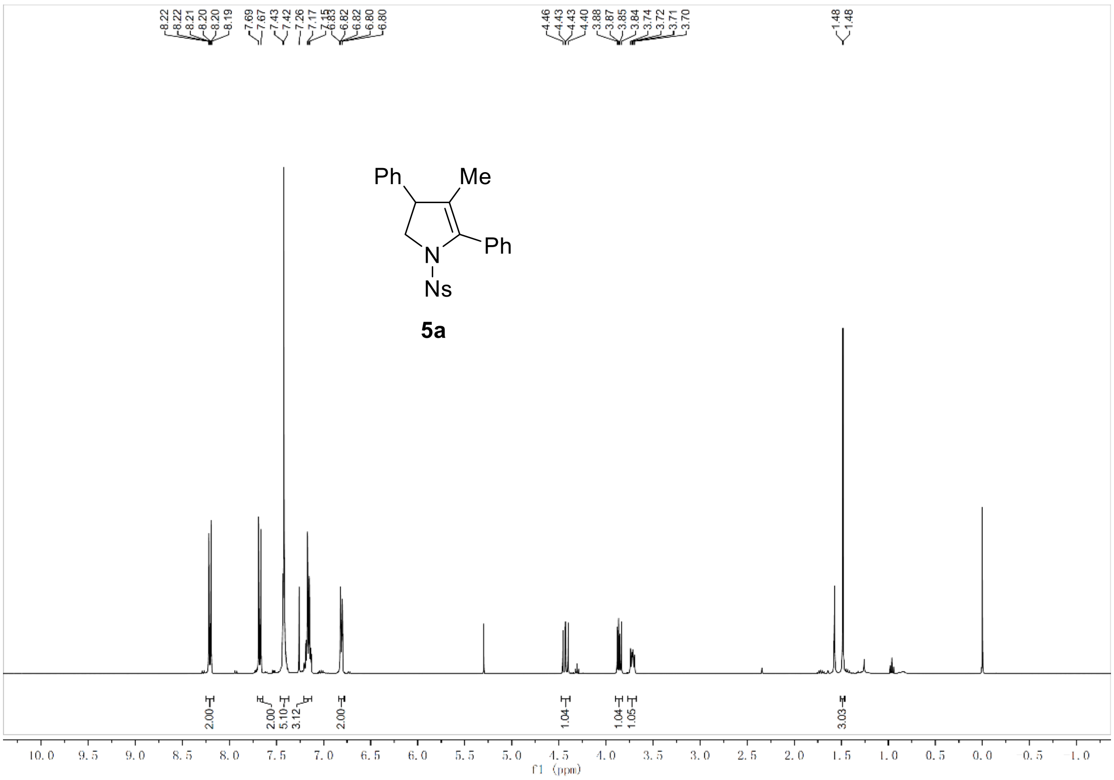

Supplementary Figure 116. ^{1}H NMR spectrum of compound 5a ($CDCl_3$, 400 MHz, 298K)

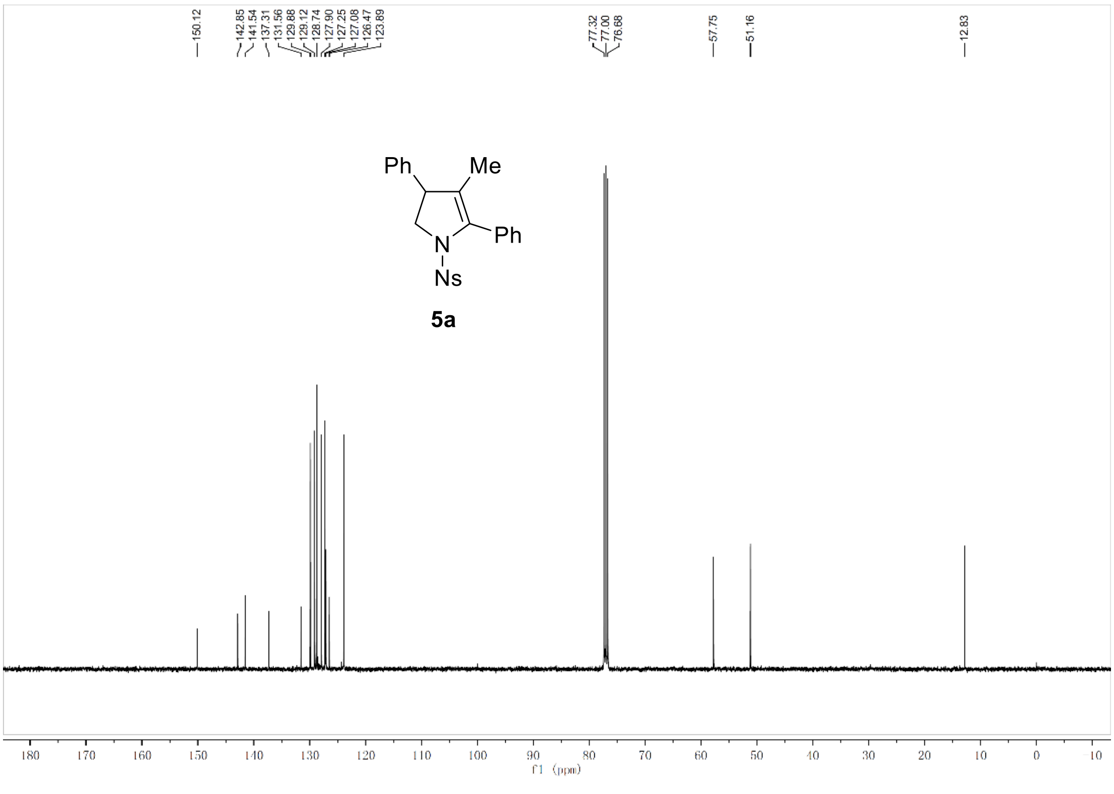

Supplementary Figure 117. ^{13}C NMR spectrum of compound 5a ($CDCl_3$, 100 MHz, 298K)

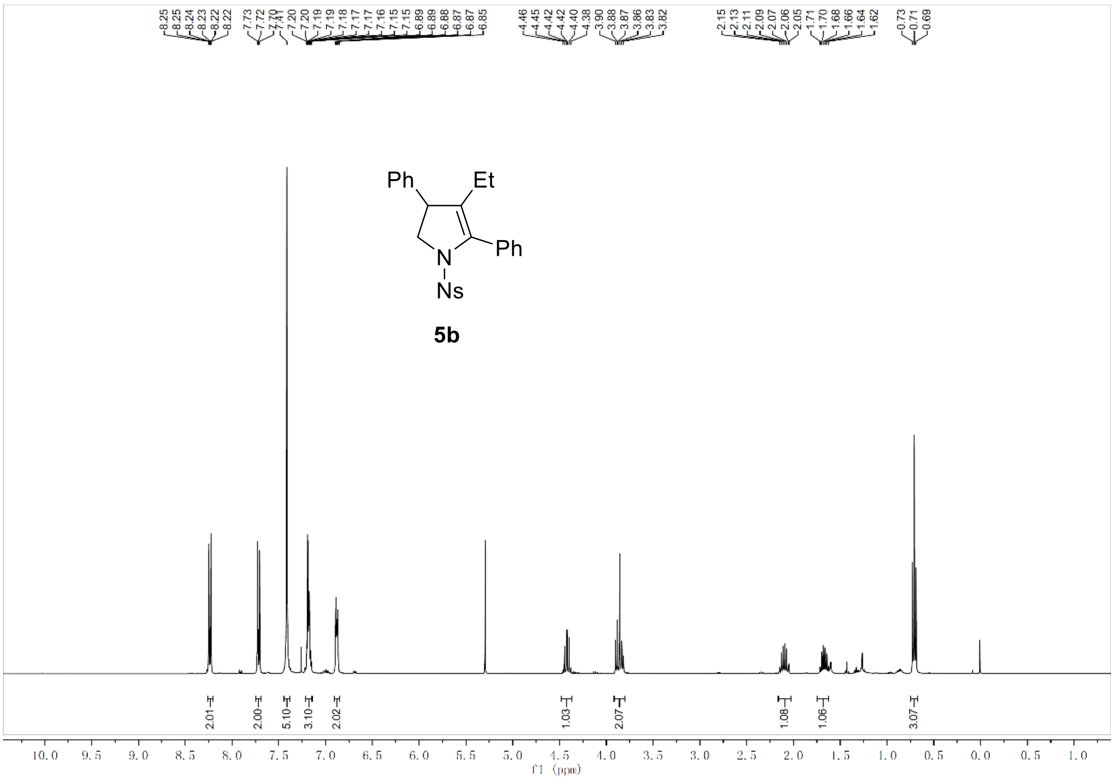

Supplementary Figure 118. ^{1}H NMR spectrum of compound 5b (CDCl$_3$, 400 MHz, 298K)

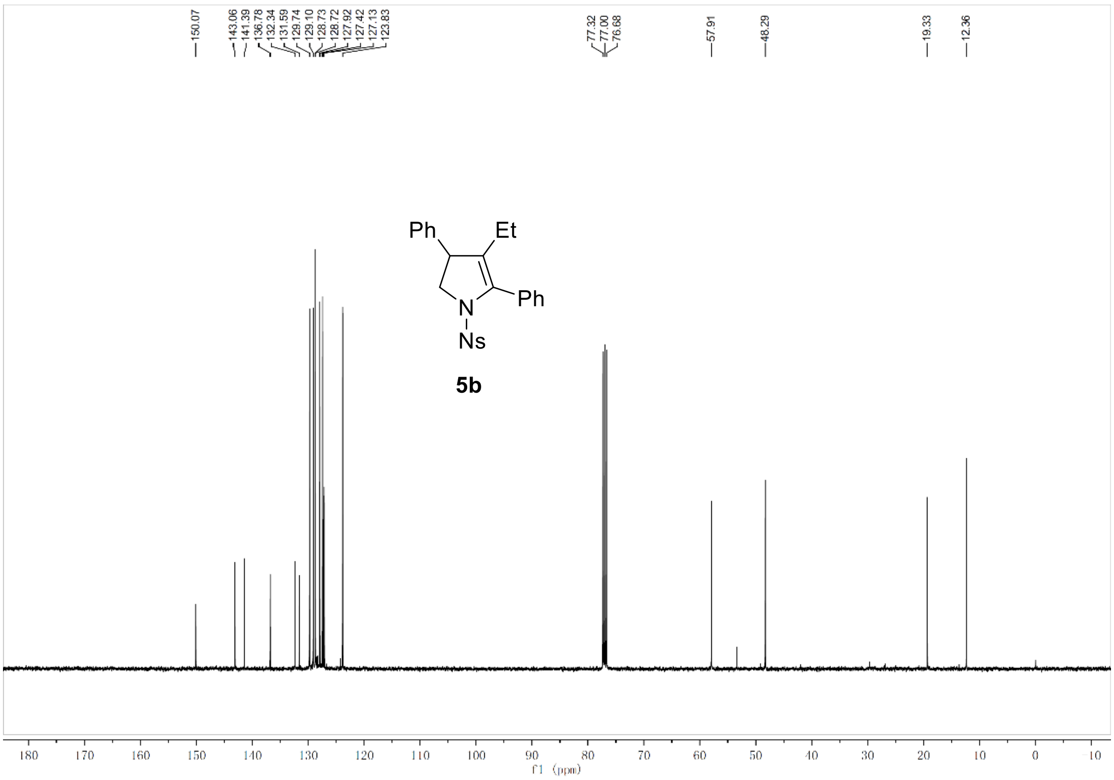

Supplementary Figure 119. ^{13}C NMR spectrum of compound 5b (CDCl$_3$, 100 MHz, 298K)

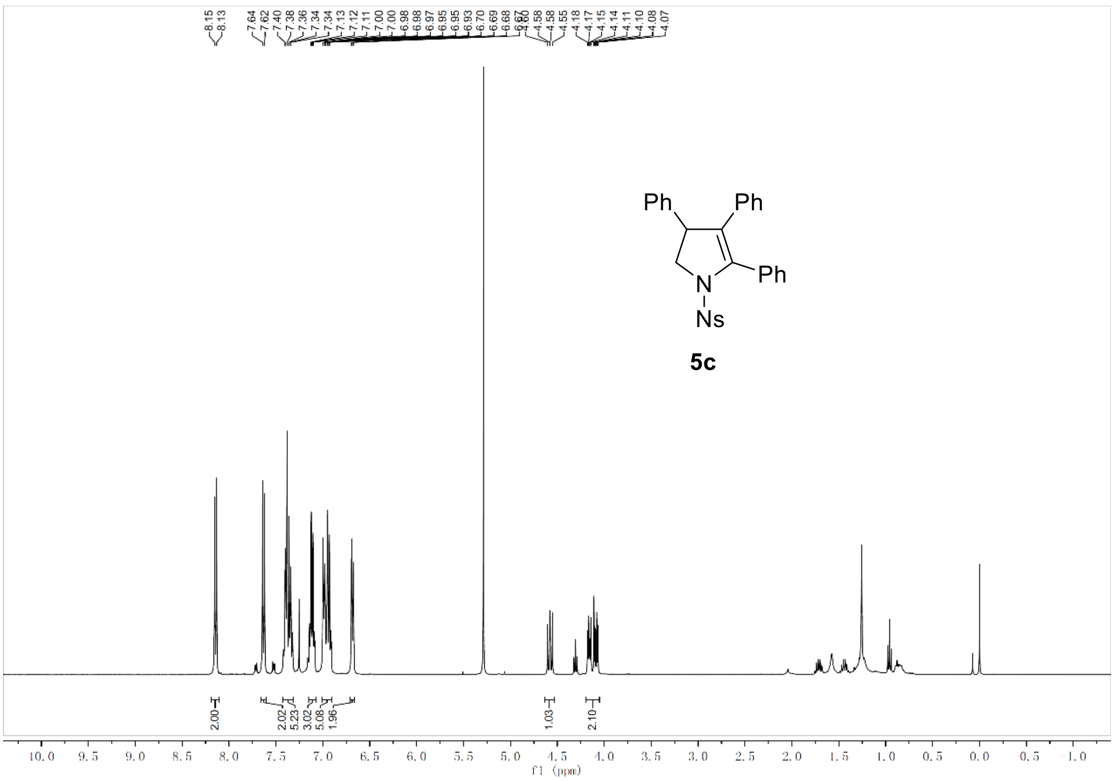

Supplementary Figure 120. ^{1}H NMR spectrum of compound 5c ($CDCl_3$, 400 MHz, 298K)

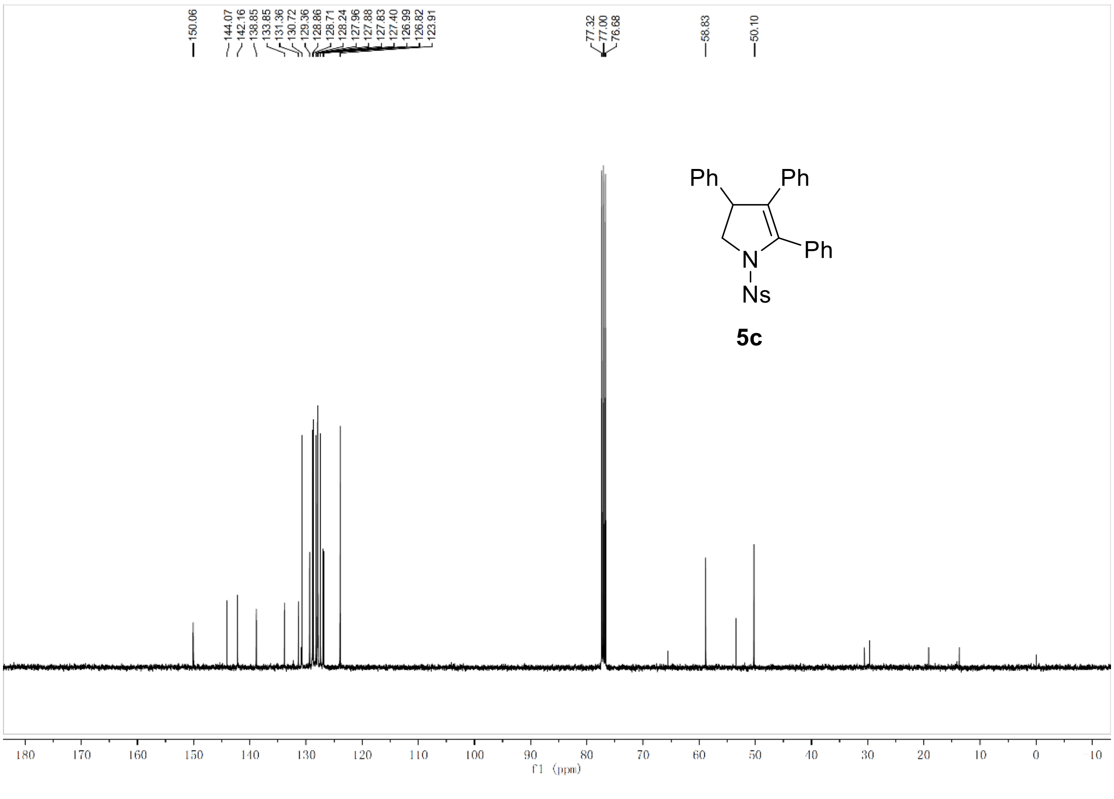

Supplementary Figure 121. ^{13}C NMR spectrum of compound 5c ($CDCl_3$, 100 MHz, 298K)

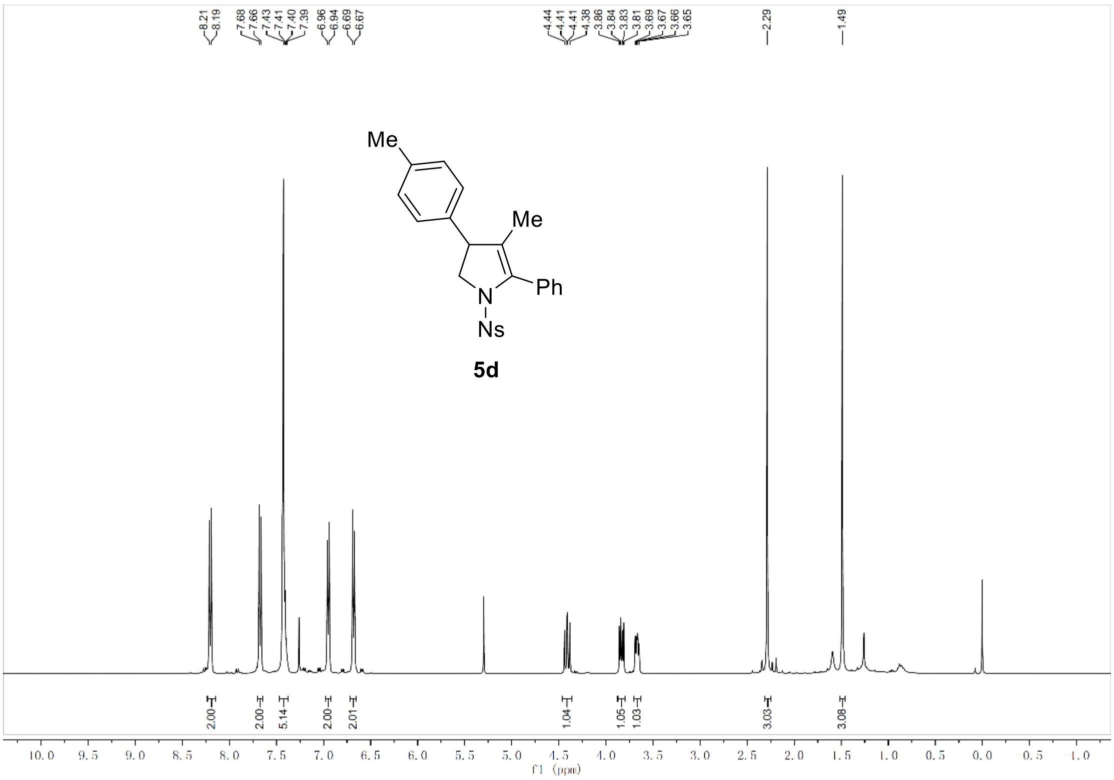

Supplementary Figure 122. ^{1}H NMR spectrum of compound 5d ($CDCl_3$, 400 MHz, 298K)

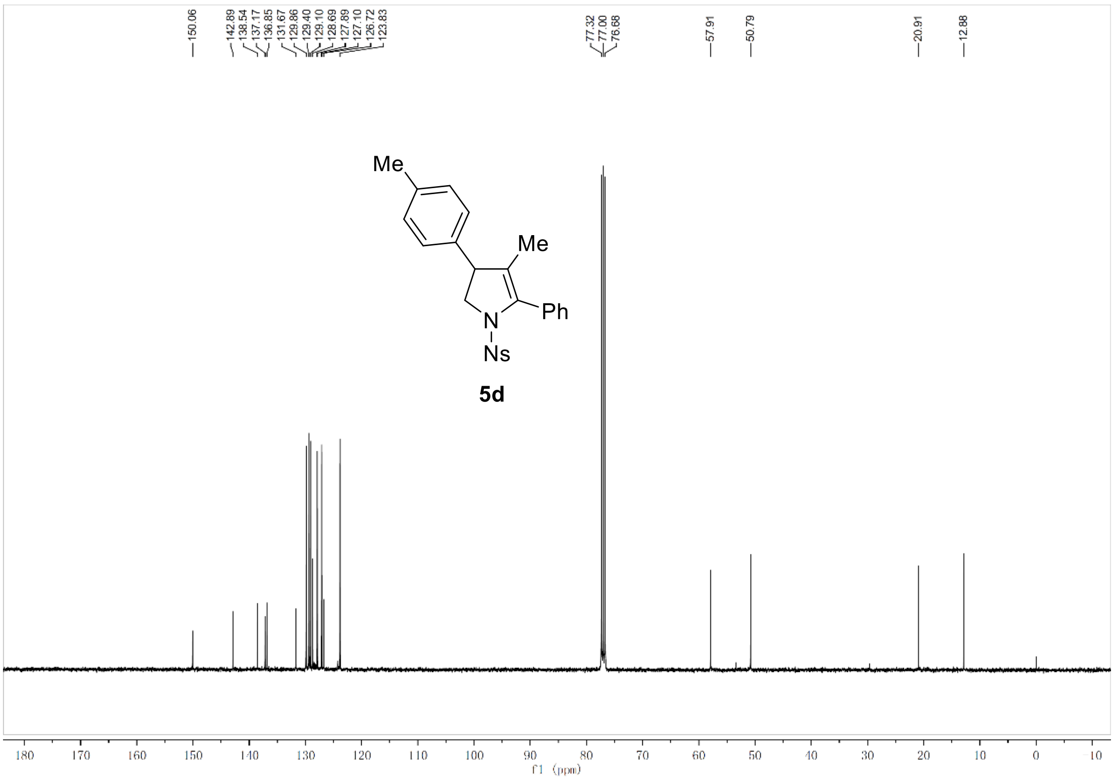

Supplementary Figure 123. ^{13}C NMR spectrum of compound 5d ($CDCl_3$, 100 MHz, 298K)

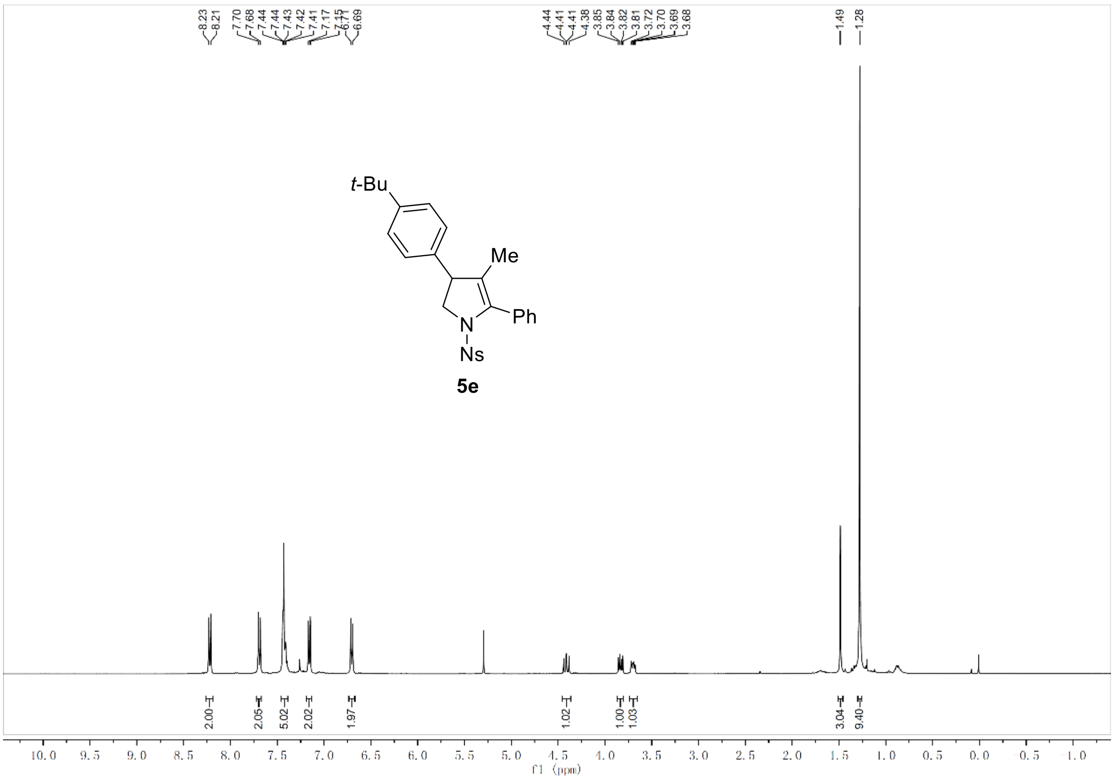

Supplementary Figure 124. ^{1}H NMR spectrum of compound 5e ($CDCl_3$, 400 MHz, 298K)

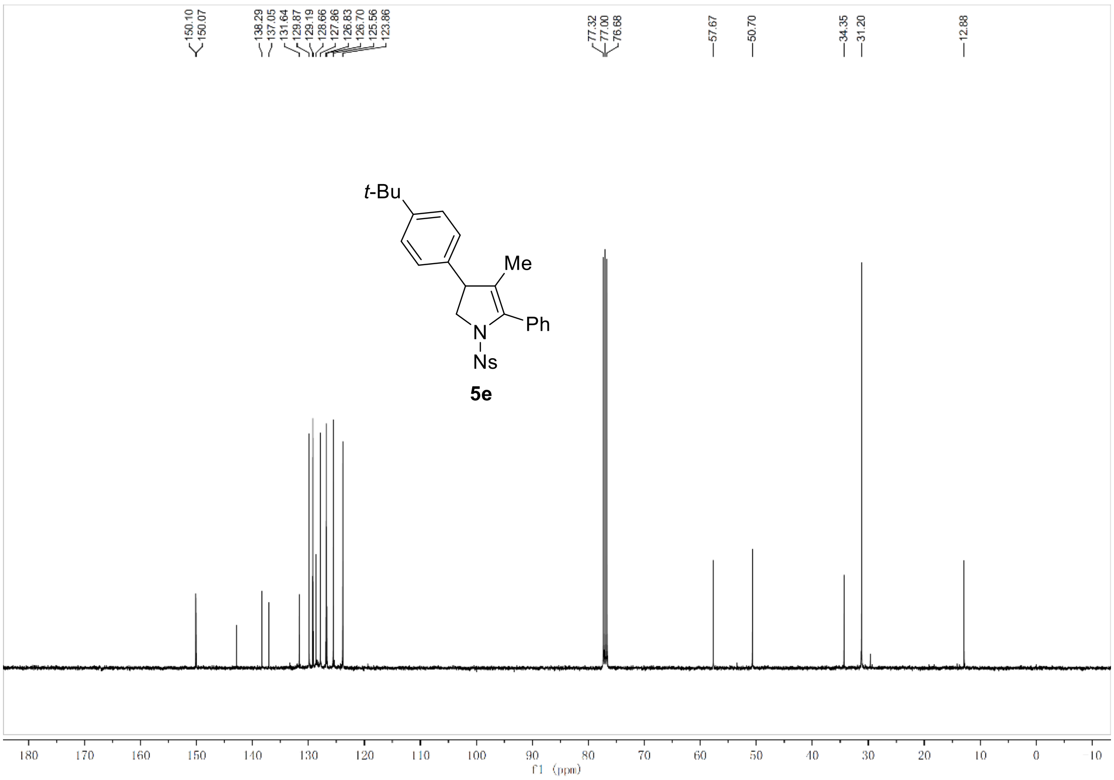

Supplementary Figure 125. ^{13}C NMR spectrum of compound 5e ($CDCl_3$, 100 MHz, 298K)

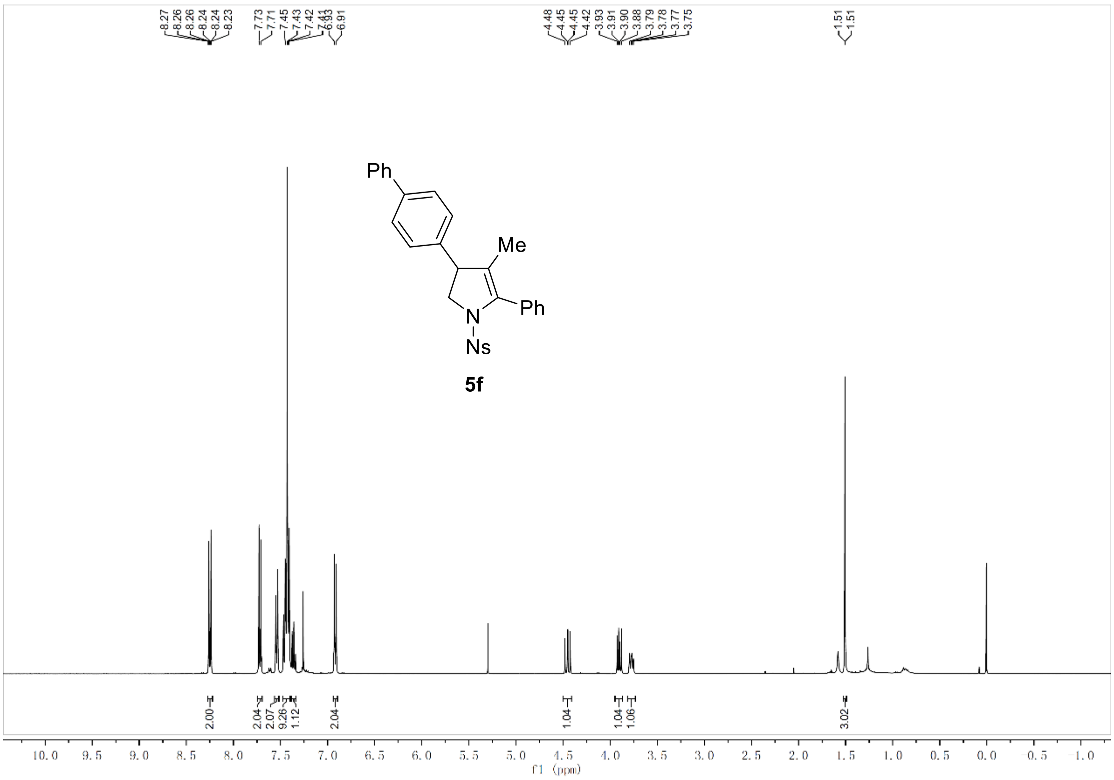

Supplementary Figure 126. ^{1}H NMR spectrum of compound 5f (CDCl$_3$, 400 MHz, 298K)

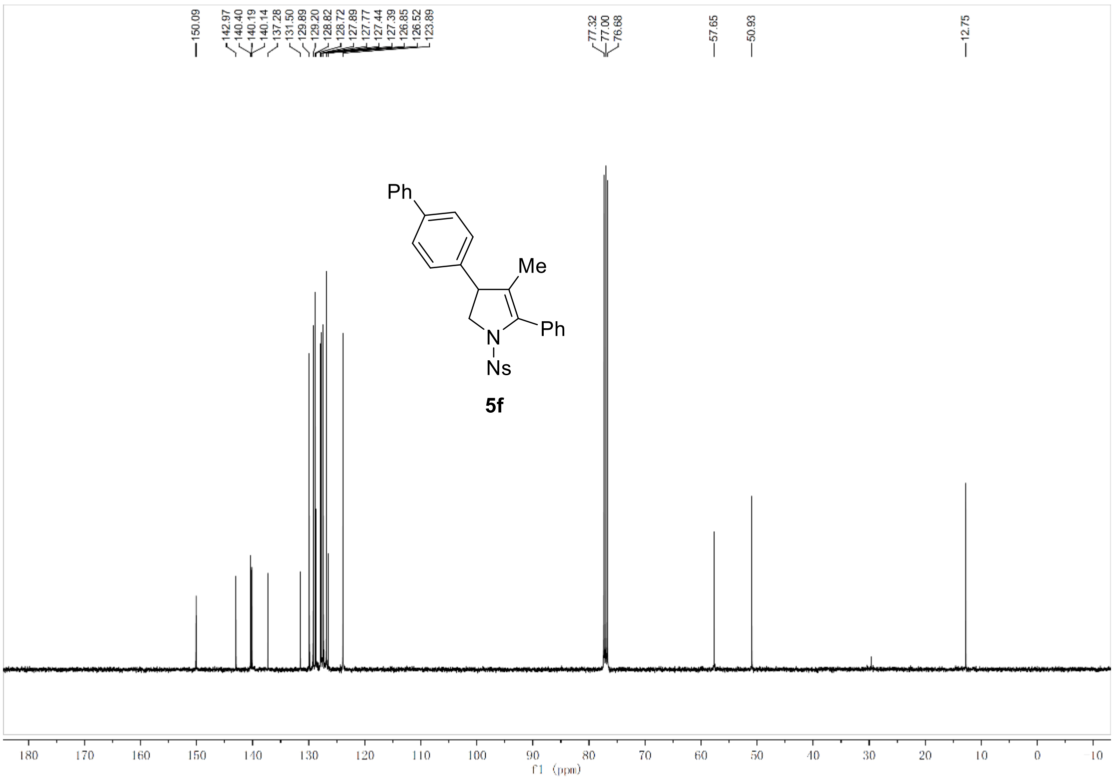

Supplementary Figure 127. ^{13}C NMR spectrum of compound 5f (CDCl$_3$, 100 MHz, 298K)

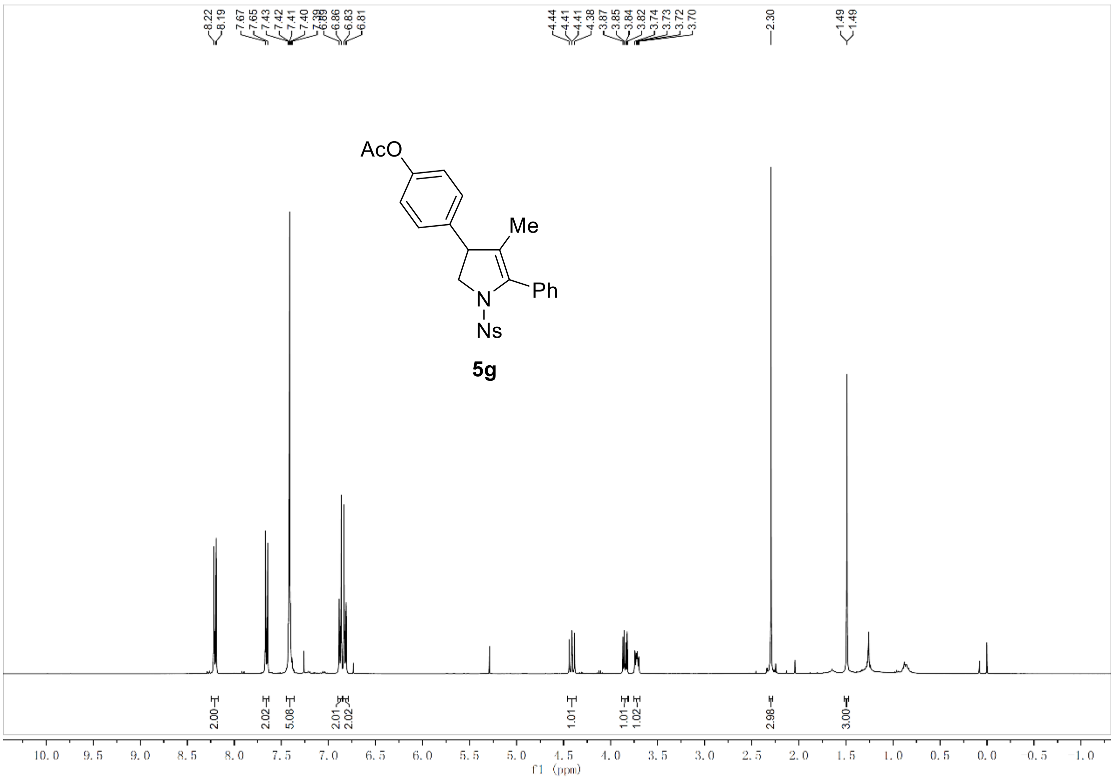

Supplementary Figure 128. ^{1}H NMR spectrum of compound 5g (CDCl$_3$, 400 MHz, 298K)

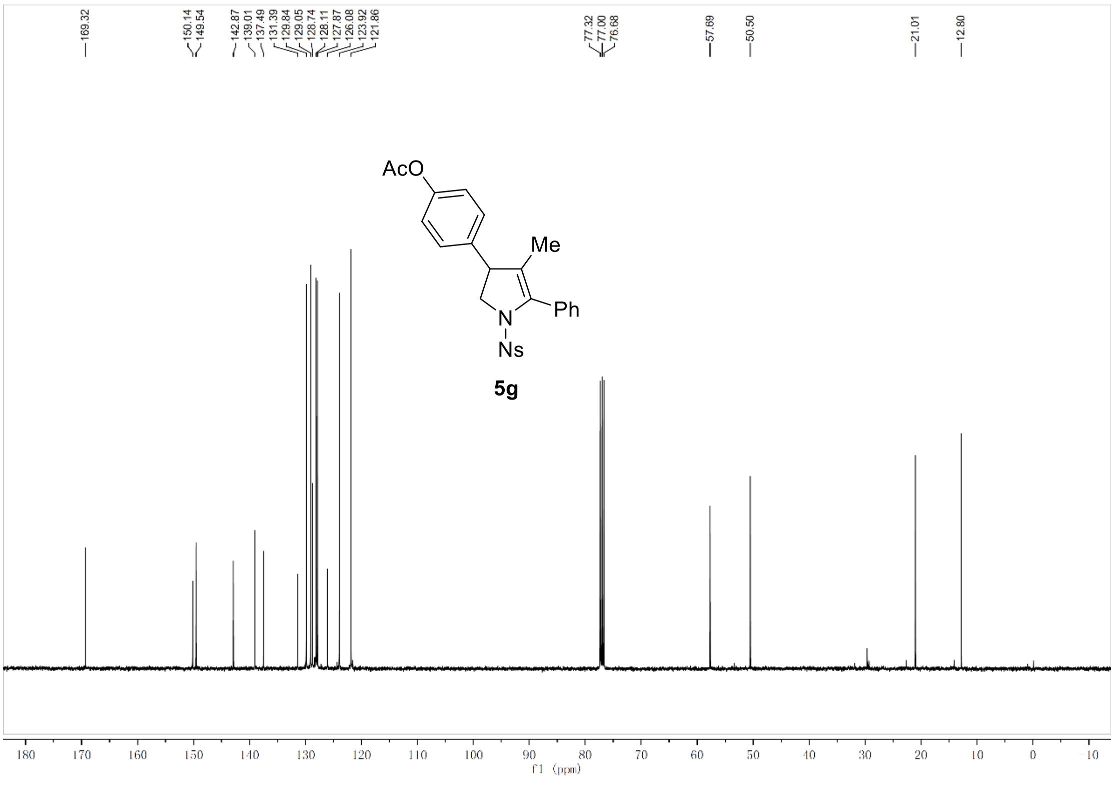

Supplementary Figure 129. ^{13}C NMR spectrum of compound 5g (CDCl$_3$, 100 MHz, 298K)

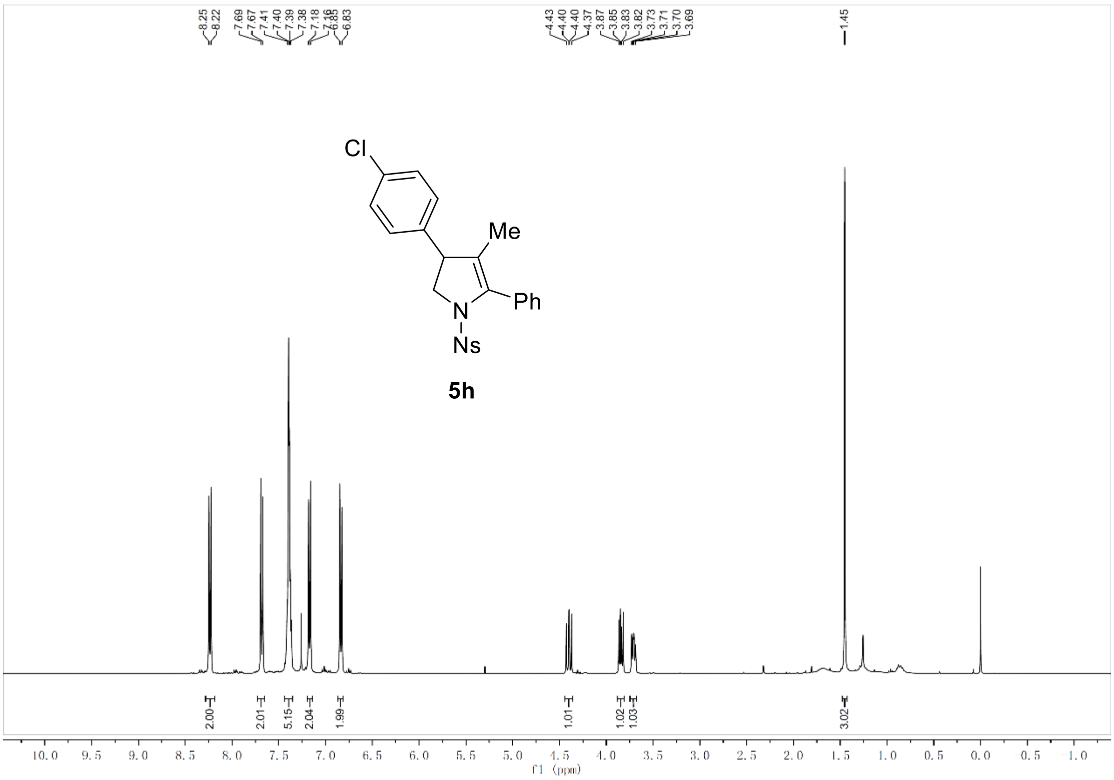

Supplementary Figure 130. ¹H NMR spectrum of compound 5h ($CDCl_3$, 400 MHz, 298K)

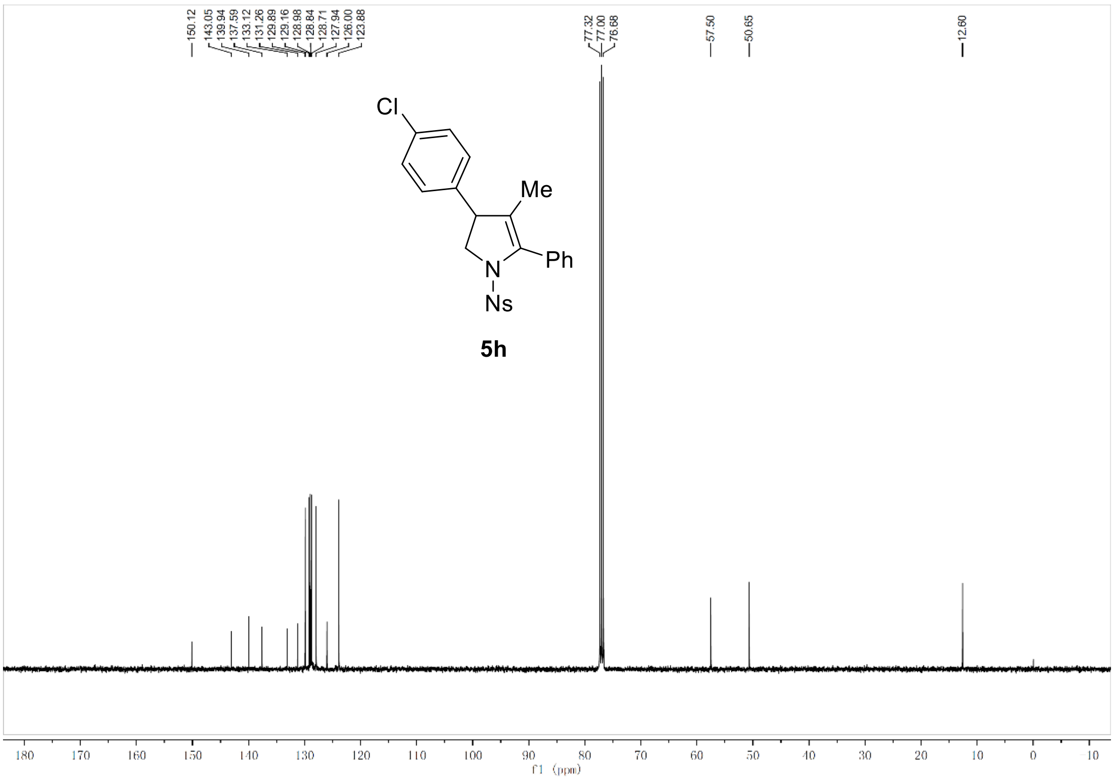

Supplementary Figure 131. ¹³C NMR spectrum of compound 5h ($CDCl_3$, 100 MHz, 298K)

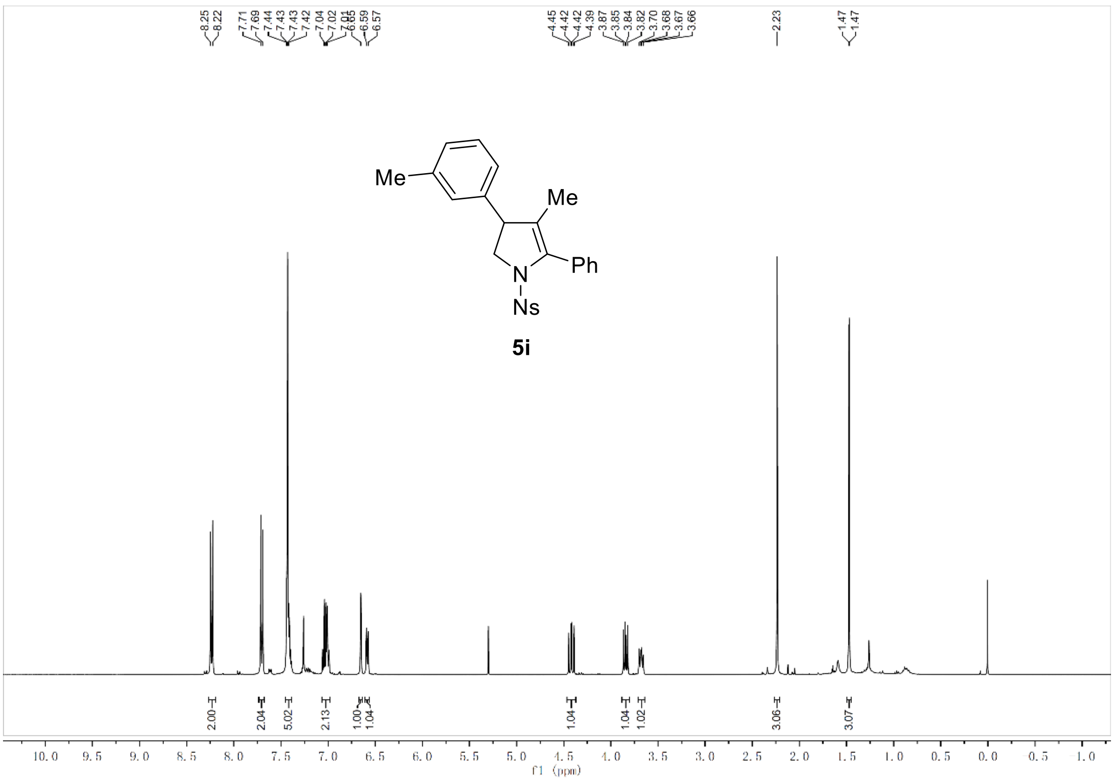

Supplementary Figure 132. ^{1}H NMR spectrum of compound 5i ($CDCl_3$, 400 MHz, 298K)

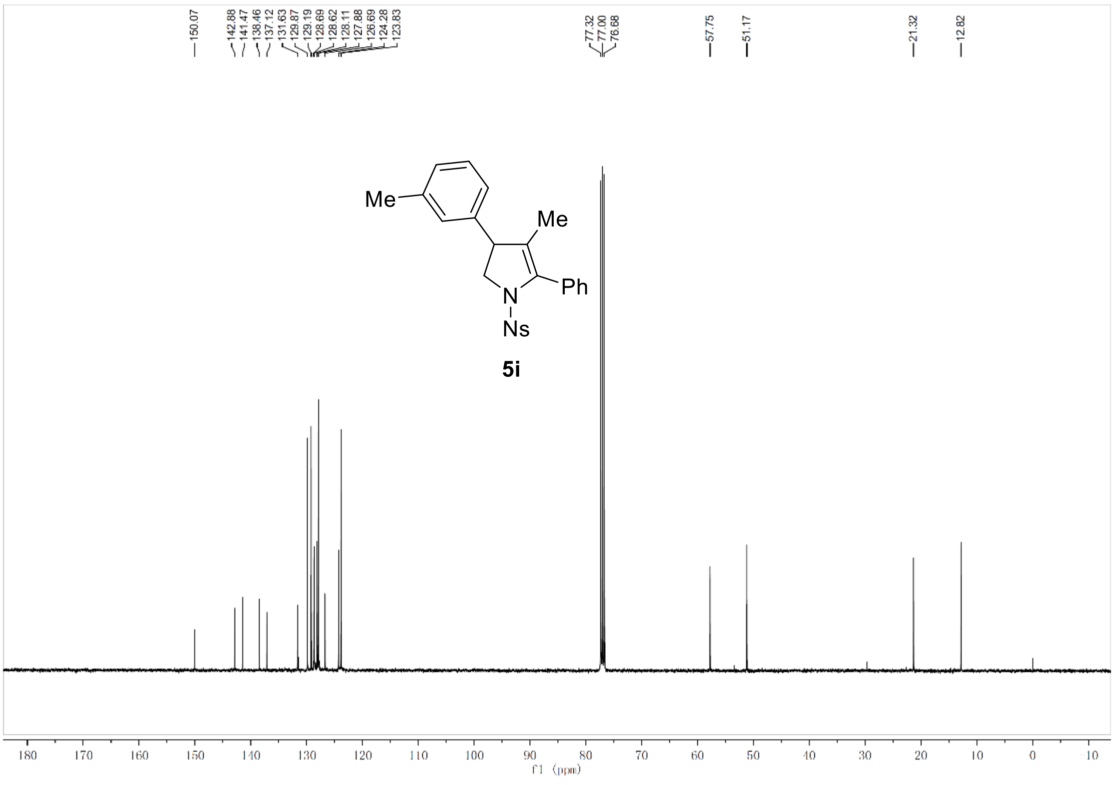

Supplementary Figure 133. ^{13}C NMR spectrum of compound 5i ($CDCl_3$, 100 MHz, 298K)

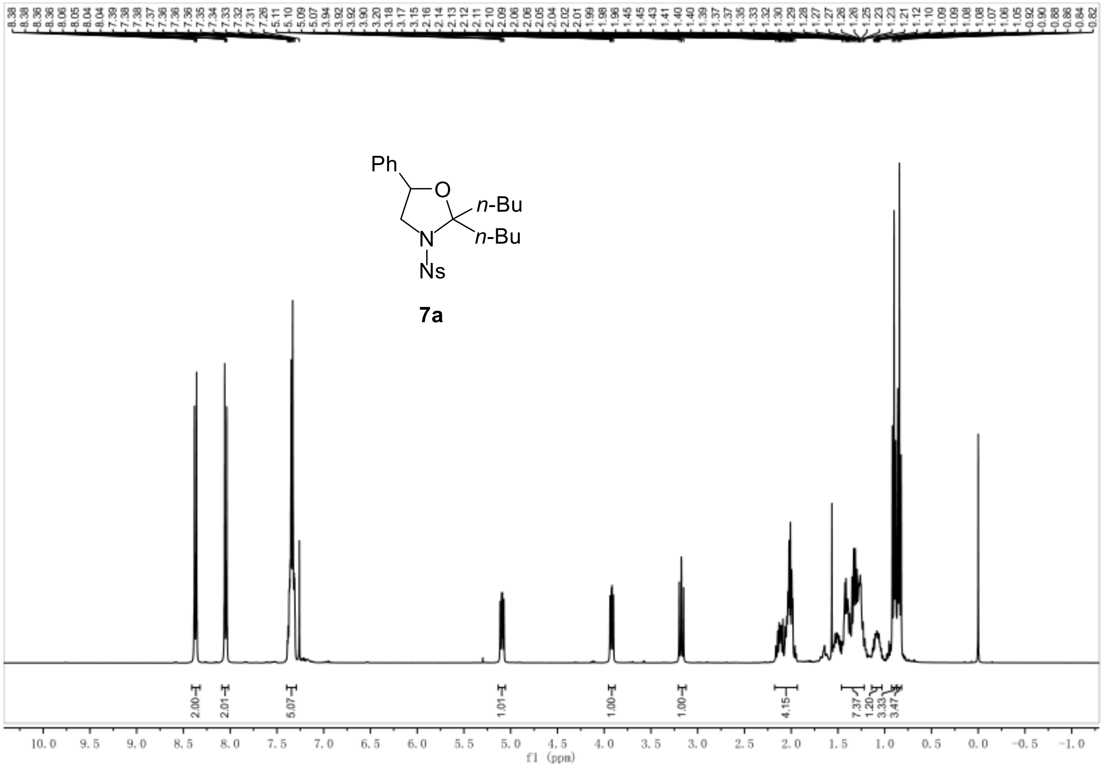

Supplementary Figure 134. ^{1}H NMR spectrum of compound 7a ($CDCl_3$, 400 MHz, 298K)

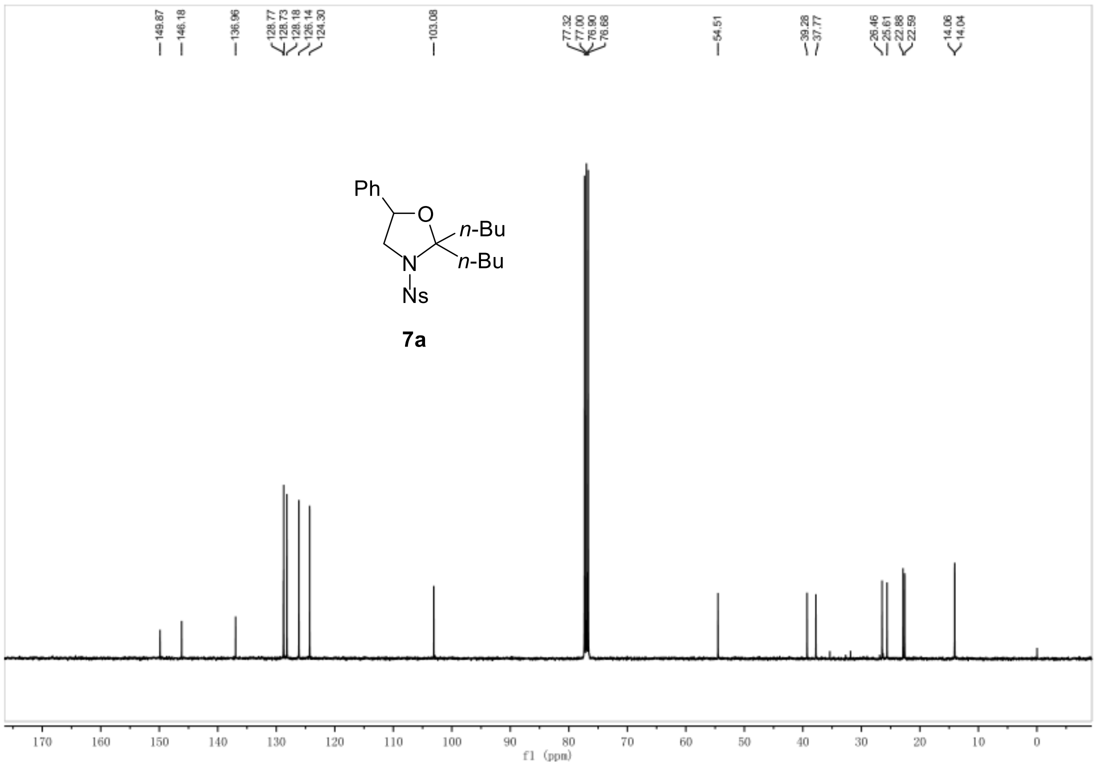

Supplementary Figure 135. ^{13}C NMR spectrum of compound 7a ($CDCl_3$, 100 MHz, 298K)

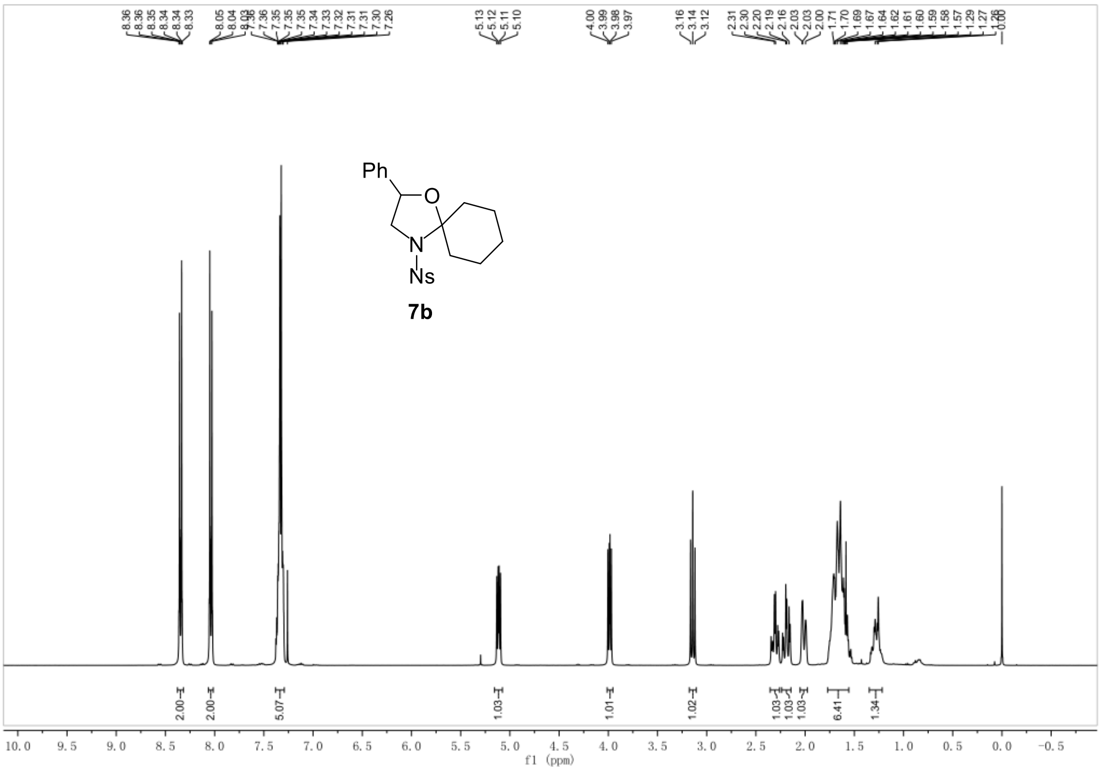

Supplementary Figure 136. ^{1}H NMR spectrum of compound 7b ($CDCl_3$, 400 MHz, 298K)

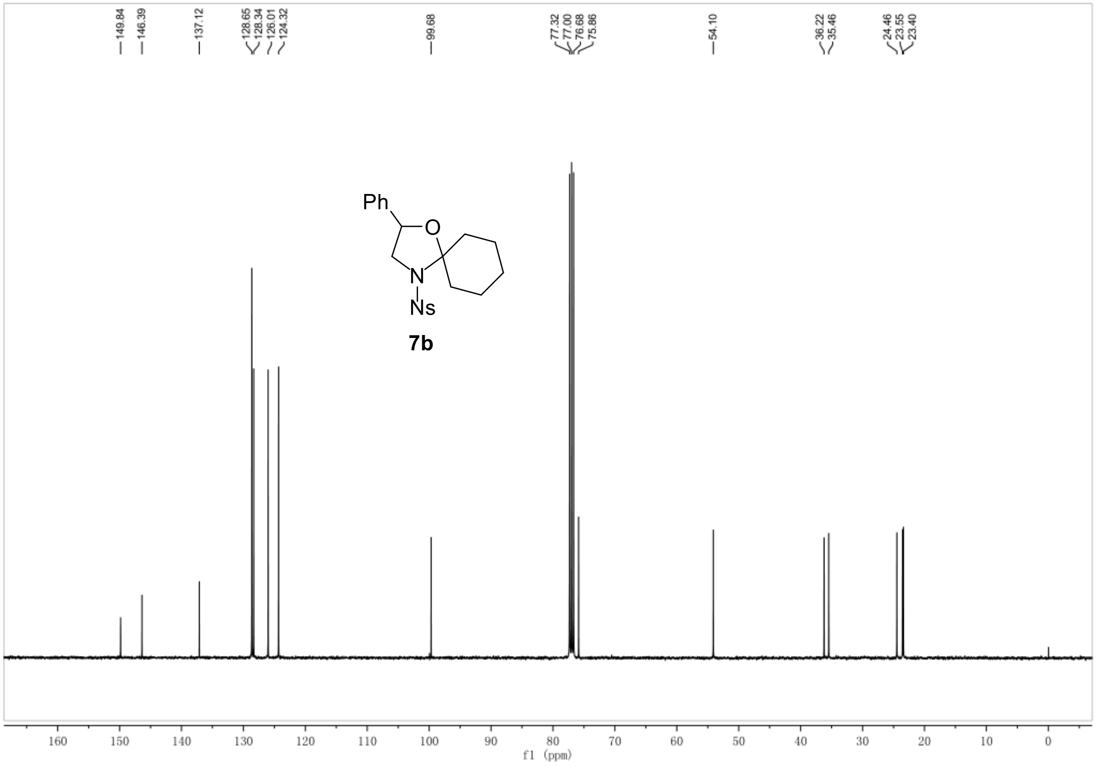

Supplementary Figure 137. ^{13}C NMR spectrum of compound 7b ($CDCl_3$, 100 MHz, 298K)

11. Supplementary References

[1] Forfar, L. C., Green, M., Haddow, M. F., Hussein, S., Lynam, J. M., Slattery, J. M., Russell, C. A. Evidence for a S_N2-type pathway in the exchange of phosphines at a $[PhSe]^+$ centre. *Dalton Trans.* **44**, 110–118 (2015).

[2] Zhao, Y. & Truhlar, D. G. The M06 suite of density functionals for main group thermochemistry, thermochemical kinetics, noncovalent interactions, excited states, and transition elements: two new functionals and systematic testing of four M06-class functionals and 12 other functionals. *Theor. Chem. Acc.* **120**, 215-241 (2008).

[3] Grimme, S., Antony, J., Ehrlich, S. & Krieg, H. A consistent and accurate *ab initio* parametrization of density functional dispersion correction (DFT-D) for the 94 elements H-Pu. *J. Chem. Phys.* **132**, 154104 (2010).

[4] Frisch, M. J. et al. Gaussian 09, Revision D.01; Gaussian, Inc.: Wallingford, CT (2013).

[5] Tomasi, J., Mennucci, B. & Cammi, R. Quantum Mechanical Continuum Solvation Models. *Chem. Rev*. **105**, 2999-3093 (2005).

[6] Bader, R. F. W. A quantum theory of molecular structure and its applications. *Chem. Rev.* **91**, 893-928 (1991).

[7] Keith, T. A. AIMAll Version 08.11.06, aim.tkgristmill.com (2008).

[8] Lu, T. & Chen, F.-W. Multiwfn: A multifunctional wavefunction analyzer. *J. Comput. Chem.* **33**, 580-592 (2012).

[9] Reed, A. E., Weinstock, R. B. & Weinhold, F. Natural population analysis. *J. Chem. Phys*. **83**, 735-746 (1985).

[10] Reed, A. E., Curtiss, L. A. & Weinhold, F. Intermolecular interactions from a natural bond orbital, donor-acceptor viewpoint. *Chem. Rev*. **88**, 899-926 (1988).
